# Needles in fungal haystacks: Discovery of a putative a-factor pheromone and a unique mating strategy in the Leotiomycetes

**DOI:** 10.1371/journal.pone.0292619

**Published:** 2023-10-12

**Authors:** Andi M. Wilson, Martin P. A. Coetzee, Michael J. Wingfield, Brenda D. Wingfield

**Affiliations:** Department of Biochemistry, Genetics & Microbiology, Forestry & Agricultural Biotechnology Institute (FABI), University of Pretoria, Pretoria, South Africa; Friedrich Schiller University, GERMANY

## Abstract

The Leotiomycetes is a hugely diverse group of fungi, accommodating a wide variety of important plant and animal pathogens, ericoid mycorrhizal fungi, as well as producers of antibiotics. Despite their importance, the genetics of these fungi remain relatively understudied, particularly as they don’t include model taxa. For example, sexual reproduction and the genetic mechanisms that underly this process are poorly understood in the Leotiomycetes. We exploited publicly available genomic and transcriptomic resources to identify genes of the mating-type locus and pheromone response pathway in an effort to characterize the mating strategies and behaviors of 124 Leotiomycete species. Our analyses identified a putative a-factor mating pheromone in these species. This significant finding represents the first identification of this gene in Pezizomycotina species outside of the Sordariomycetes. A unique mating strategy was also discovered in *Lachnellula* species that appear to have lost the need for the primary MAT1-1-1 protein. Ancestral state reconstruction enabled the identification of numerous transitions between homothallism and heterothallism in the Leotiomycetes and suggests a heterothallic ancestor for this group. This comprehensive catalog of mating-related genes from such a large group of fungi provides a rich resource from which in-depth, functional studies can be conducted in these economically and ecologically important species.

## Introduction

Studying model species has played an essential role in biological experimentation. These species have been used for the functional characterization of genes involved in development [1], aging [2], and various diseases [3]. In fungi, studies using model species have enabled in- depth research into the production of complex tissue types, cell-to-cell communication and response to the environment (reviewed in [4]). These discoveries have facilitated an improved understanding of multicellularity, mechanisms of reproduction, and other complex processes that are more difficult to investigate in non-fungal Eukaryotes. Understandably, our knowledge regarding the genetic mechanisms that underly sexual development in filamentous fungal species relies heavily on model species such as *Neurospora crassa* [5, 6], *Podospora anserina* [7, 8], *Fusarium graminearum* [9, 10] and other Sordariomycetes in which mating behaviors have been studied, manipulated, and exploited for decades.

Sexual reproduction in the Ascomycota can be achieved via heterothallism, which involves physical interactions between two compatible partners, or via homothallism, which usually involves a single individual capable of sexual development in isolation (reviewed in [11]). However, a unique type of homothallism, termed mating type switching, occurs when individuals of one mating type, and thus compatibility, are able to switch to the alternate mating type, thus changing the isolates with which they are compatible. Mating type switching can occur uni-directionally (permanent) or bi-directionally (temporary), depending on the genetic mechanisms underlying the switch (reviewed in [12]). In species where the switch is facilitated by DNA deletion, this is uni-directional (e.g.: *Ceratocystis fimbriata* [13]), while switching events facilitated by DNA inversion are typically bi-directional (e.g.: *Sclerotinia trifoliorum* [14]). This sexual strategy allows a single isolate to produce progeny that have different mating types which are therefore compatible with one another and are thus capable of sexual reproduction.

The advent of next generation sequencing (NGS) technologies and amenable genome editing protocols is facilitating progress towards understanding sexual reproduction in non-model fungal species. Our collective understanding of the sexual cycles of filamentous ascomycete fungi has been especially enriched by these advances, with genome and transcriptome sequencing enabling the identification and comparison of many genes associated with sexual development [15–19]. Similarly, genome editing has enabled the functional characterization of these genes with respect to their roles in mating and other biological processes [20–22]. Even so, these studies have typically focused on species residing in the Sordariomycetes, and particular species in the Eurotiomycetes (e.g., *Aspergillus nidulans* [23–26]), but much less attention has been paid to the other fungal classes, such as the Leotiomycetes and Dothideomycetes.

While there are hundreds of genes that are important for sexual development in fungi [reviewed in [4]], two sets of genes are particularly well-characterized with respect to their importance in this pathway. These are the mating-type (*MAT*) genes and the genes encoding the mating pheromones and their receptors. The *MAT* genes are the master regulators of sexual reproduction and typically encode transcription factors that influence the expression of many other genes involved in mating [27, 28]. These genes are typically harbored within the *MAT* locus, which possesses genes from both *MAT* idiomorphs (i.e., *MAT1-1* and *MAT1-2*) in the case of most homothallic species. In heterothallic species, the locus contains genes from only one of the two idiomorphs, with the presence of *MAT1-1* genes conferring the MAT1-1 mating type and the presence of *MAT1-2* genes conferring the MAT1-2 mating type. In this way, the *MAT* proteins determine the mating type of the fungus, which in turn dictates mating compatibility. The pheromones and their receptors are thought to act as physiological messengers of mating type and alert individuals to the presence of compatible mating partners. As such, many heterothallic Sordariomycetes express these pheromones in a mating type-dependent manner [16, 29, 30].

Genes encoding the fungal mating pheromones were first identified in *Saccharomyces cerevisiae*, with a simple peptide pheromone expressed by the α-cell and a lipopeptide pheromone being expressed by the a-cell [31, 32]. This mating type-dependent relationship resulted in these pheromones being referred to as the α- and a-factors, even in fungi whose mating types are designated as MAT1-1 and MAT1-2. Mating pheromones and their receptors have subsequently been identified in the genomes of many filamentous fungi, especially those within the Sordariomycetes. Heterothallic fungi like *N*. *crassa* [29], *Cryphonectria parasitica* [30], *Magnaporthe grisea* [33], and *Huntiella omanensis* [16] harbor both the α- and a- factors pheromones, which are preferentially expressed in the MAT1-1 and MAT1-2 mating types, respectively. Both pheromone factors have also been identified in the genomes of numerous homothallic Sordariomycete species such as *F*. *graminearum* [34] and *Sordaria macrospora* [35], but their expression profiles are not as tightly linked to the *MAT* locus. The α-factor pheromone has also been identified from species in classes outside of the Sordariomycetes, including the Eurotiomycetes [36, 37] and Leotiomycetes [38]. In contrast, a gene encoding the a-factor pheromone has never been found in any filamentous ascomycete fungi outside of the Sordariomycetes.

The Leotiomycetes is an incredibly diverse class of fungi [39]. It includes important plant pathogens such as *Botrytis cinerea* [40], *Rhynchosporium secalis* [41], *Oidium neolycopersici* [42] and *Hymenoscyphus fraxineus* [43] as well as ericoid mycorrhizal fungi such as *Hyaloscypha* species [44] and *Gamarada debralockiae* [45]. The group also includes mammalian pathogens such *Pseudogymnoascus destructans* [46], and those that produce important compounds such as antibiotics (*Ascocoryne sarcoides*, [47]) and antifungals (*Glarea lozoyensis*, [48]). Some of these fungi have well-documented and regularly observed sexual cycles, including those that produce cleistothecia, such as *Erysiphe pisi* [49] and those that produce apothecia in the case of *B*. *cinerea* [50]. Yet, the mechanisms underpinning sexual reproduction have not been investigated in most Leotiomycete species, and in some cases sexual structures have never been seen [39].

The many important impacts of meiosis on the life cycles of fungi demand a greater number of studies to fully understand the capacity of these fungi to sexually reproduce. Undergoing meiosis allows for the production of genetic diversity as well as the purging of deleterious alleles, which may allow for an increased rate of adaptation to changing environmental conditions [51]. Additionally, various fungi rely on the sexual cycle to produce the spores that act as the inoculum for infection, enable short- and long-distance spread, and/or act as the over-wintering structures [52–55]. Thus, understanding whether species are capable of sexual reproduction and if so, whether they require a mating partner, directly impacts epidemiological models, and is therefore an important consideration when establishing potential control strategies in the case of pathogenic fungi [52–55]. Furthermore, the ability to control sexual reproduction in biotechnologically-important fungi aids in strain development in these species [56, 57].

The motivation for this study lay in the recognition that the Leotiomycetes do not include model species that have been utilized to elucidate the genetics underpinning sexual reproduction and that little is known regarding this process in these fungi. We thus used comparative genomics tools and mined 124 publicly available genomes for genes encoding the mating-type proteins as well as the mating pheromones and their cognate receptors. The emerging information was then used to trace transitions between heterothallism and homothallism in the class. This is the first study, to our knowledge, to systematically catalog and compare these genes in the Leotiomycetes and it provides a rich resource from which in-depth, functional studies can be conducted in these non-model fungi.

## Methods

### Data used

A total of 136 genomes for isolates of fungi residing in the Leotiomycetes were downloaded from the National Centre for Biotechnology Information (NCBI) in December 2021. Of these, nine were excluded from further analysis due to their poor assembly statistics (S1 File). A further three were excluded because they had been incorrectly labelled (S1 File). The 124 genomes that were retained represented three orders, 22 families and 53 genera of the Leotiomycetes as well as a number of *incertae sedis* species whose taxonomic placement within the Leotiomycetes was not clear (S1 Table). Additionally, RNA-seq datasets for seven species were downloaded from the sequence read archive (SRA) at the NCBI (S1 Table).

### Genome annotation

Many of the genomes that were used had associated GFF files that were also downloaded (S1 Table) and used for downstream applications. The online version of the genome annotator AUGUSTUS [58] was used to predict genes in the genomes for which annotation files were not available. The default settings and the *B*. *cinerea* gene models were used for gene prediction.

Numerous genes involved in mating (as detailed below) were found to have incorrect gene models in the downstream applications. This was true of the annotations downloaded from NCBI as well as the annotations predicted by AUGUSTUS. It was possible to identify these errors because the encoded proteins did not harbor the correct functional domains, or the genes possessed an unexpected number of introns. Additionally, some genes, particularly those encoding the two mating pheromones were not annotated at all. In these cases, we produced alignments using homologs from close relatives to determine whether an appropriate model could be manually produced. Additionally, when available, we mapped RNAseq reads to confirm intron-exon boundaries.

DNA- and RNA-based alignments and mappings were produced in CLC Main Workbench V22.0 and CLC Genomics Workbench V22.0, respectively. The DNA-based alignments, using the gene sequences from related species, were generated using the *Create Alignment* function in the *Alignments and Trees* module, with default settings. The RNA-based mappings, using trimmed RNA-seq reads, were produced using the *Map Reads to Contigs* function in the *De Novo Sequencing* module with default settings, a minimum similarity fraction of 0.8, and a minimum length fraction of 0.5. The alignments and mappings were manually inspected, curated, and used for the annotation of the gene regions in question.

Throughout this paper, we have described genes that were not present and/or intact as deleted, missing, truncated, fragmented, or that they could not be annotated. The terms deleted and missing referred to genes which could not be found within the genome at all, even when using tBLASTn and synteny-based analyses. These genes have been lost from the genome entirely. Genes that were described as truncated or fragmented were genes whose 5’ or 3’ regions could be identified using BLAST analyses but that did not encode for the full-length proteins. Lastly, genes that could not be annotated typically referred to genes where partial regions could be identified using BLAST but there had been sufficient degeneration or pseudogenization of the gene so that no suitable gene and CDS annotations could be made.

### *MAT* gene identification and sexual strategy determination

Five *MAT* genes are commonly associated with Leotiomycete species; *MAT1-1-1*, *MAT1-1-3*, *MAT1-1-5*, *MAT1-2-1* and *MAT1-2-10* (previously *MAT1-2-4*, but revised in [59]). Various BLASTn and tBLASTn analyses were used to identify these genes in our dataset, using gene and protein sequences downloaded from NCBI, respectively (S2 Table). In instances where no mating-type sequences were identified (E-value > 0.05), the gene and/or protein sequences from APN2 and SLA2 were used as BLAST queries to identify the *MAT* locus (S2 Table). These two genes are commonly associated with the *MAT* locus in Pezizomycotina species, often flanking the locus, and do not evolve as fast as the *MAT* genes. This makes the *APN2* and *SLA2* genes ideal candidates for BLAST-based analyses.

The gene content of the *MAT* loci was used to predict the sexual strategy of each species. Species were labelled as heterothallic if genes from only a single idiomorph were present in the representative genome. In contrast, when genes from both idiomorphs were present, the species were labelled as homothallic or undetermined. Species were classified as homothallic when genes from both idiomorphs were identified within a single locus. Many of the species that were categorized as undetermined typically harbored truncated MAT1-1-1 or MAT1-2-1 genes, making it difficult to predict whether they were capable of sexual reproduction at all. Alternatively, these undetermined species possessed genes from both idiomorphs, but that were assembled into different loci.

While the majority of homothallic species harbored genes suggestive of primary homothallism, a few species exhibited evidence for mating type switching, a form of secondary homothallism. In these species, it was possible to identify the genetic signals of switching, including the presence of identical repeats and/or fragments of the *MAT1-1-1* gene flanking the *MAT1-2*-associated genes.

### α-Factor pheromone identification

The α-factor pheromone is typically the easiest of the two mating pheromones to identify as it encodes a protein with a recognizable and unique structure. The full length, pro-pheromone protein harbors a signal peptide at the N-terminal and thus begins with a hydrophobic region. This is followed by repeats of a conserved 9–14 amino acid sequence representing the mature pheromone. These conserved repeats are typically preceded by an STE13 recognition sequence (XA, XP, or repeating units of XAXA, XPXP, or XAXP) and followed by the KEX1/2 recognition sites (KR, RR, or KK).

The α-factor pheromone was identified using a variety of BLAST-based analyses, including tBLASTn and BLASTn queries using *S*. *sclerotiorum* and *B*. *cinerea* ppg1 gene and protein sequences (S2 Table). This approach typically allowed for the identification of the α-factor pheromone from at least a single representative per family. The family-specific representative sequence was subsequently used to identify the α-factor pheromones from the remaining species in that genus/family. For example, the *S*. *sclerotiorum* PPG1 protein sequence was used to identify the α-factor from the *Drepanopeziza brunnea* genome and this sequence was subsequently used to identify the α-factor from the other species in the family Drepanopezizaceae.

In a number of species, using the α-factor pheromone sequence in BLAST analysis did not allow for the α-factor pheromone to be identified. As a consequence, we employed a microsynteny approach as described by Wilson *et al* [60]. In this case, genes flanking the α-factor pheromone in closely related species were used as BLAST queries. When these genes were found adjacent to one another, the intergenic region was searched manually for the presence of α-factor-like gene sequences.

The repeats within the predicted α-factor proteins were manually annotated, based on the conserved motifs listed above. Repeats were annotated in between recognizable STE13 and KEX1/2 recognition sites (S1 Fig). The first residue following the XA or XP site was designated as the first residue of the repeat. The final residue of the repeat was more difficult to define and was typically done on a case-by-case basis. In general, the final residue was the one that directly preceded the KEX1/2 recognition site. However, in a number of cases, additional residues were present between the KEX1/2 recognition site and what was thought to be the mature repeat. In these cases, we chose to annotate repeats that would result in the length of the repeat being consistent within the putative amino acid sequence (S1 Fig).

### a-Factor pheromone identification

The a-factor pheromone has typically been more difficult to identify in the genomes of filamentous fungi, even amongst close relatives. The gene encodes a very short protein (<100 aa) and only has a single strictly conserved character- a C-terminal CAAX or CPAX domain (where C = cysteine, A = any aliphatic residue, P = any polar residue and X = any residue). This domain is not exclusive to the a-factor pheromone and thus the identification of this gene requires additional evidence from other data.

Methods similar to those used to identify the α-factor pheromone were used in an attempt to identify the a-factor pheromone. BLAST-dependent analyses were conducted using the a-factor pheromones from more than 40 Sordariomycetes and yeasts (S2 File) [38, 60–62]. Microsynteny-based approaches, as described above, were also utilized. Neither of these methods yielded usable results, likely due to the vast phylogenetic distance between the query species and the Leotiomycetes.

BLAST-independent analyses were subsequently conducted. Firstly, the CDS annotations from reasonably well-annotated genomes (*B*. *cinerea*, *B*. *sinoalli*, *S*. *sclerotiorum*, *M*. *fructicola*, *R*. *commune*, *A*. *resinae*, and *P*. *destructans*) were extracted and translated into predicted protein sequences. These lists were manually filtered to retain only proteins ≤ 100 aa and harboring a terminal CAAX or CPAX domain (S3 File). These lists were then used as local BLASTn queries against the genomes of close relatives to determine whether the gene was conserved within and beyond genus borders. This method was based on the method utilized by Srikant *et al* [62] to identify the a-factor in the Saccharomycotina.

The final step of the BLAST-independent analyses was one to determine whether the potential a-factor genes were expressed and furthermore, if they were expressed in a mating type-dependent manner. These analyses were done on three heterothallic species, *M*. *fructicola*, *B*. *cinerea*, and *A*. *resinae* (S1 Table). Raw RNAseq reads were trimmed using the default settings in the *Trim reads* function in the *Prepare Sequencing Data* module in CLC Genomics V22.0. These reads were subsequently mapped to the genome and CDS annotations using the *RNA-Seq Analysis* function in the *RNA-Seq and Small RNA Analysis* module in the same program. Default settings were used, except that the minimum similarity fraction was set to 0.8 and the minimum length fraction was set to 0.5. Expression was calculated as an RPKM value.

### Pheromone receptor identification

The two pheromone receptor genes, *ste2* (α-factor receptor) and *ste3* (a-factor receptor), were identified using BLASTn and tBLASTn analyses, using *B*. *cinerea* and *S*. *sclerotiorum* gene and protein sequences (S2 Table). Once identified, the CDS annotations of these genes were translated. These proteins were then annotated using Phobius (https://phobius.sbc.su.se/; transmembrane domains) and the NCBI Conserved Domain Search (https://www.ncbi.nlm.nih.gov/Structure/cdd/wrpsb.cgi; functional domains). In some cases, the functional domains were not detectable and/or an incorrect number of transmembrane domains (i.e.: ≠7) were annotated. These genes were subjected to manual re-annotation as described above, using DNA and RNA alignment in an effort to produce proteins with the correct domains.

### Phylogenomic analyses and ancestral state reconstruction

The phylogenomic analyses were conducted in a manner comparable to other recent studies using large genomic datasets from diverse fungi [63–65]. Single copy orthologs were identified in the genomes included in this study using BUSCO (Benchmarking Universal Single-Copy Orthologs) v. 4.0.6 [66, 67]. For this purpose, the *leotiomycetes_odb10* database was used. Orthologs that were common to all taxa studied were included in the phylogenomic analyses below. Amino acid sequences were aligned within each data file with MAFFT v. 7.407 [68, 69] using the default settings. Poorly aligned sequence regions were removed with TrimAl v. 1.2 using the gappy mode [70] and data matrices with alignments of less than 200 characters were removed from the collection.

Phylogenetic analyses were conducted on a concatenated dataset as well as on individual gene sequences. FASconCAT-G v1.0 was used to generate a concatenated dataset (a supermatrix) as well as a file defining the partitions. Maximum likelihood analyses were performed with IQTree v.2.2 [71, 72] to construct a phylogenetic tree from the supermatrix, as well as to generate individual gene trees. Evolutionary models were selected for each partition in the supermatrix and for each gene matrix using ModelFinder [73] within IQTree and incorporated in the ML analyses. Nodal support values were determined with the ultra-fast bootstrap (UFBoot) and the Shimodaira-Hasegawa-like approximate likelihood-ratio test (SH-aLRT) [74, 75] in all ML analyses with 1000 replicates. The most likely tree, which was obtained from the supermatrix, was rooted with *A*. *pellizariae* as the outgroup taxon. A supertree was generated from all gene trees obtained above. This was done using ASTRAL version 5.7.8 [76] with default settings and nodal support values were determined by calculating posterior probabilities. The tree was rooted with the outgroup.

Ancestral state reconstruction of mating systems was conducted to assess their evolution over the phylogeny of the species considered. A Bayesian approach was followed by employing MrBayes Ancestral States with R (MBASR) [77]. The phylogenetic tree obtained above from the supermatrix served as backbone onto which the character states were mapped. Character states were encoded in terms of thallism (homothallic = 0, heterothallic = 1, unknown = ?) and treated as unordered discreet characters. The number of samples was set to 1 000 (i.e. 100 000 generations in the Markov Chain Monte Carlo run).

**Fig 1:**
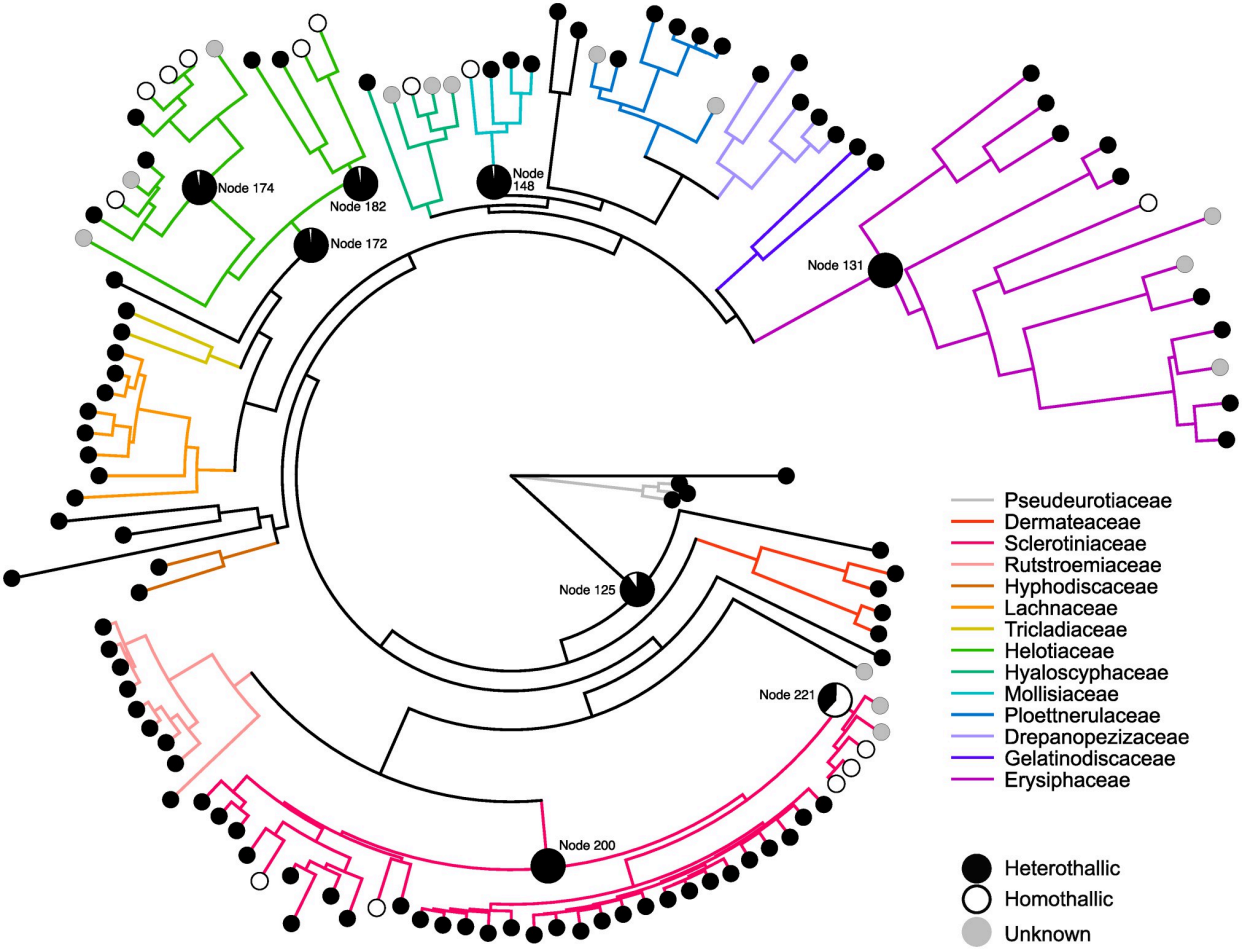
Ancestral state reconstruction of the Leotiomycete mating strategies. There is widespread heterothallism throughout the class, with 96 of the 124 species being classified as heterothallic, as indicated by black circles. Only 15 species were classified as homothallic, as indicated by white circles, with 10 of these harboring *MAT* loci indicative of primary homothallism and five of them possessing *MAT* loci suggestive of mating type switching. Ancestral state reconstruction strongly suggests a heterothallic ancestor for most genera, families and the entire class, as indicated by the circles at internal nodes within the phylogeny. The black and white sections within these circles show the likelihood of the ancestor represented at this node being heterothallic or homothallic, respectively. Further information regarding the sexual strategy of the ancestor of the Sclerotiniaceae is provided in the Discussion. Taxa for which it was not possible to determine a mating strategy are indicated by grey circles. An identical phylogeny with the taxa names can be found in the Supplementary data (S3 Fig).

The phylogeny presented in Fig 1 was generated using the tree based on the supermatrix and was visualized in R Studio v. 2022.12.0. The tree was plotted using the *ape* and *phytools* packages, and the *plotTree* function [78, 79]. Character state statistics from the ancestral state reconstruction analyses were mapped onto important nodes within the phylogeny (S4 File). The final tree was edited using Affinity Designer v 1.10.

## Results

### Phylogenomic analyses

Of the 3 234 BUSCO (Benchmarking Universal Single-Copy Orthologs) genes in the Leotiomycete database, 76 were present as complete, single copy orthologs in all 124 genomes included in this study. Of these, 72 gene alignments possessed more than 200 characters and were subsequently used for the phylogenomic analyses. The phylogenies produced using the concatenated dataset and the individual gene trees had similar topologies with minor variation (S2 Fig). The topology of the trees was congruent with a recent multigene phylogeny produced for the Leotiomycetes using 3 156 genes from 49 species in a concatenated dataset [39].

### A novel *MAT* gene identified in the Leotiomycetes

Intact *MAT* loci were identified and annotated from 104 of genomes considered in this study. Most of these genomes harbored genes that have been previously described from the *MAT* loci of the Leotiomycetes, including *MAT1-1-1*, *MAT1-1-3*, *MAT1-1-5*, *MAT1-1-2* and *MAT1-2-10* (S1 Table). In addition to these, a novel *MAT1-1*-associated gene was discovered from the *MAT* loci of species belonging to the genus *Rhynchosporium* (Ploettnerulaceae, Helotiales).

The novel gene is short, only 330 bp, and is made up of a CDS with two exons interrupted by an intron and encodes a protein of only 90 aa (S5 File). No homologs were identified in other species within the Ploettnerulaceae, suggesting a very recent origin of this gene. BLASTp and tBLASTn analyses against the non-redundant databases at the NCBI did not identify any previously described homologs. The protein contained no identifiable conserved domains and no evidence for a signal peptide or transmembrane domain according to the NCBI Conserved Domain search, Expasy ProtScale or Phobius, respectively. A proposed name for this gene is *MAT1-1-13* following the Pezizomycotina *MAT* nomenclature system [59].

### *MAT* locus restructuring and evidence for transitions between heterothallism and homothallism

Sexual reproduction in the Leotiomycetes was found to include heterothallism, primary homothallism and mating type switching (Fig 1, S3 Fig and S1 Table). Genes from only a single idiomorph were identified in 96 of genomes included in this study, indicative of heterothallism. An additional genome, harboring genes from a single idiomorph, was also identified, but it was not possible to determine whether the genome represented a heterothallic species or an isolate that had undergone mating type switching.

In the remaining genomes, genes associated with both the *MAT1-1* and *MAT1-2* idiomorphs were identified. Of these, 10 had *MAT* locus structures that were suggestive of primary homothallism while a further five harbored *MAT* loci that were indicative of mating type switching [80]. While the final 13 genomes also possessed both *MAT1-1* and *MAT1-2*-associated genes, it was not possible to predict the sexual strategy of these isolates (S6 and S7 Files). In 10 cases, the *MAT1-1-1* and/or *MAT1-2-1* genes were truncated and thus it was not clear whether the species were capable of sexual reproduction at all. In three cases, the *MAT1-1* and *MAT1-2*-associated genes were present on separate contigs and there was a possibility that the genomes represented mixed cultures, making it difficult to determine whether the isolates should be classified as heterothallic or homothallic.

Given the widespread occurrence of heterothallism throughout the Leotiomycetes, ancestral state reconstruction strongly suggested a heterothallic ancestor for this class. This was also true for most genera within the Leotiomycetes, with numerous transitions towards homothallism occurring independently within certain families (Fig 1 and S3 Fig). Families in which these transitions have occurred are discussed in more detail below, including descriptions of the transitions between these sexual strategies as well as the associated *MAT* locus restructuring events.

#### The Sclerotiniaceae

**Fig 2:**
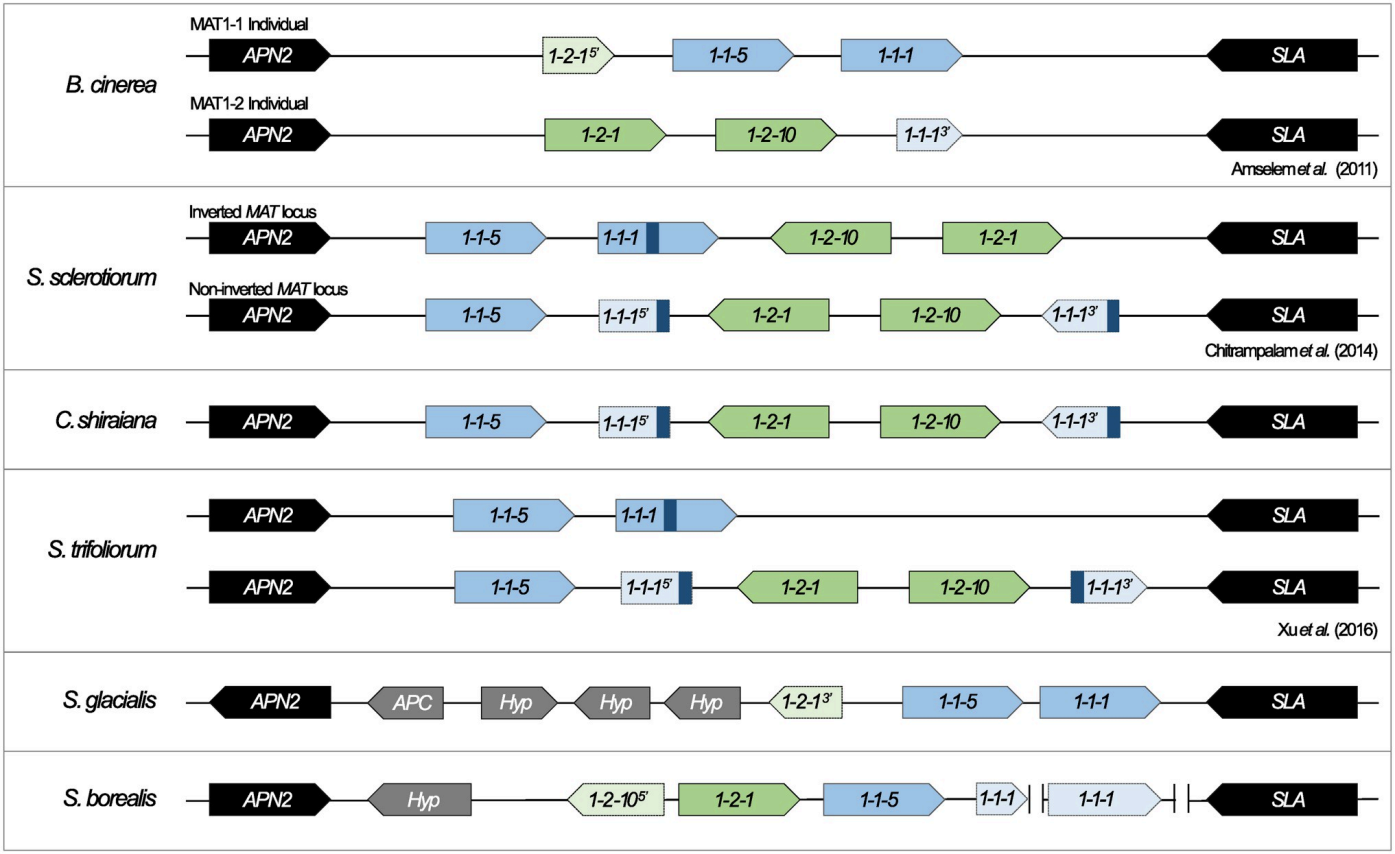
The *MAT* locus structure variety within the Sclerotiniaceae. *Botrytis cinerea* is heterothallic and thus individual isolates possess one of two different *MAT* idiomorphs- *MAT1-1* and *MAT1-2*. *Sclerotinia sclerotiorum* and *Ciboria shiraiana* both undergo mating type switching via an inversion at the *MAT* locus. This is facilitated by two 250bp identical, inverted repeats within the *MAT1-1-1* gene (indicated in dark blue). Both the inverted and non-inverted *MAT* locus structures are indicated for *S*. *sclerotiorum*. In *S*. *sclerotiorum*, this inversion results in the production of two compatible mating partners and thus allows for sexual reproduction. This is classified as a type of homothallism as sexually compatible isolates are generated from a single starting culture. *Sclerotinia trifoliorum* undergoes unidirectional mating type switching via DNA loss at the *MAT* locus. This process is also facilitated by two identical, direct repeats (indicated in dark blue). The loci of *S*. *glacialis* and *S*. *borealis* are unique, making it more challenging to assign a sexual strategy to these species. The fact that the *MAT1-1* idiomorph possesses a *MAT1-2-1* fragment in *S*. *glacialis* may be indicative of heterothallism, as this structure is reminiscent of the *MAT1-1* idiomorph of *B*. *cinerea*. The *MAT* locus of *S*. *borealis* is indicative of either primary homothallism or some kind of mating type switching, due to the presence of both *MAT1-1* and *MAT1-2*-associated genes. However, the fragmented nature of the locus made it difficult to determine which sexual strategy it employs. The *MAT1-1*-associated genes are indicated in blue, while the *MAT1-2*-associated genes are indicated in green. Lightly colored genes with dotted outlines are truncated, the region that is present is indicated by the 5’ or 3’ notation after the gene name. Eg: In *S*. *glacialis*, the 3’ region of the *MAT1-2-1* gene is present, while the 5’ region of this gene is missing. APN2: AP endonuclease, APC: anaphase promoting complex protein, Hyp: Hypothetical protein, SLA: cytoskeleton assembly control protein. These gene diagrams are not drawn to scale, but instead aim to illustrate the similarities and differences between the locus structures of these species.

In the 36 Sclerotiniaceae species investigated, heterothallism was found to dominate, with 25 of these species having been described as heterothallic or having a *MAT* locus structure consistent with heterothallic behavior (Figs 1 and 2 and S1 Table). There are a few notable exceptions, including *Sclerotinia sclerotiorum* [80], which undergoes mating type switching via an inversion at the *MAT* locus, as well as *Sclerotinia trifoliorum* [14, 81] and *Monilinia vaccinii-corymbosi* [82], which undergo mating type switching via DNA loss at this locus.

The *MAT* locus structure of *Ciboria shiraiana* suggests that this species undergoes mating type switching via inversion similar to *S*. *sclerotiorum* (Fig 2). The isolate that was used for genome sequencing harbored intact *MAT1-1-5*, *MAT1-2-1* and *MAT1-2-10* genes. The *MAT1-2-1* and *MAT1-2-10* genes were flanked by two fragments of the *MAT1-1-1* gene. Two inverted but identical repeats (250 bp) were present within the two *MAT1-1-1* fragments and would likely facilitate an inversion event, allowing for the generation of an intact *MAT1-1-1* gene.

The *MAT* loci of *Sclerotinia glacialis* and *Sclerotinia borealis* exhibited unique gene structures, which may be indicative of mating type switching or heterothallism (Fig 2). In *S*. *glacialis*, the locus harbors intact *MAT1-1-1* and *MAT1-1-5* genes, but no *MAT1-2-10* gene. Additionally, while there is a sequence homologous to the 3’ region of the *S*. *sclerotiorum MAT1-2-1* gene, the 5’ region of this gene is missing in *S*. *glacialis*. This is reminiscent of the *B*. *cinerea MAT1-1* idiomorph, which possesses *MAT1-1-1* and *MAT1-1-5* and a short fragment of the *MAT1-2-1* gene and thus might point to heterothallism [83].

The *S*. *borealis MAT* locus was not fully assembled but exists as three separate fragments. The largest fragment possessed the *APN2* gene, a hypothetical protein, a truncated version of *MAT1-2-10*, and intact homologs of *MAT1-2-1* and *MAT1-1-5* (Fig 2). The *MAT1-1-1* gene is separated across two contigs and could not be manually annotated to form a full-length protein. Lastly, the *SLA2* gene is on a separate contig. Given the presence of *MAT1-1* and *MAT1-2*-associated genes, it is likely that this species exhibits some form of homothallism- but whether it is primary homothallism or a type of mating type switching was not clear.

#### The Mollisiaceae

There were three genomes available from the genus *Phialocephala* (sexual state: *Mollisia*). Two of these, *Phialocephala* sp. D728 and *Phialocephala subalpina*, had *MAT* loci characteristic of heterothallic fungi, possessing the *MAT1-1* (*MAT1-1-1* and *MAT1-1-3*) and *MAT1-2* (*MAT1-2-1*) idiomorphs, respectively. These *MAT* loci were also flanked by *SLA2* and *APN2*. However, a third species, *Phialocephala scopiformis*, harbored the *MAT1-1-1*, *MAT1-1-3* and *MAT1-2-1* genes, which, in contrast, were associated with a SAM-Mtase gene [84]. This indicated that *P*. *scopiformis* is homothallic and suggested a transition towards homothallism in this genus as well as in the family.

#### The Helotiaceae

In total, 14 Helotiaceae species were used in this study, five of which were confirmed to be heterothallic and six of which were thought to be homothallic (S1 Table). It was difficult to determine the sexual strategy of *Hymenotorrendiella dingleyae*, as the *MAT1-1*-associated genes were flanked by *APN2* and *SLA2* while the *MAT1-2-1* gene was present elsewhere in the genome. This might be indicative of homothallism, but could also mean that the genome was generated from a mixed culture of MAT1-1 and MAT1-2 individual(s). Additionally, it was impossible to determine the sexual strategy of *Hymenoscyphus linearis* and *Hymenoscyphus herbarum*. The genome of *H*. *linearis* encodes an intact *MAT1-1-1* gene but both the *MAT1-1-3* and *MAT1-2-1* genes were truncated. In contrast, *H*. *herbarum* contains an intact *MAT1-1-3* and *MAT1-2-1* genes, but no *MAT1-1-1* gene. It is thus unclear whether these species are capable of sexual reproduction- given the absence of typically essential mating type genes.

**Fig 3:**
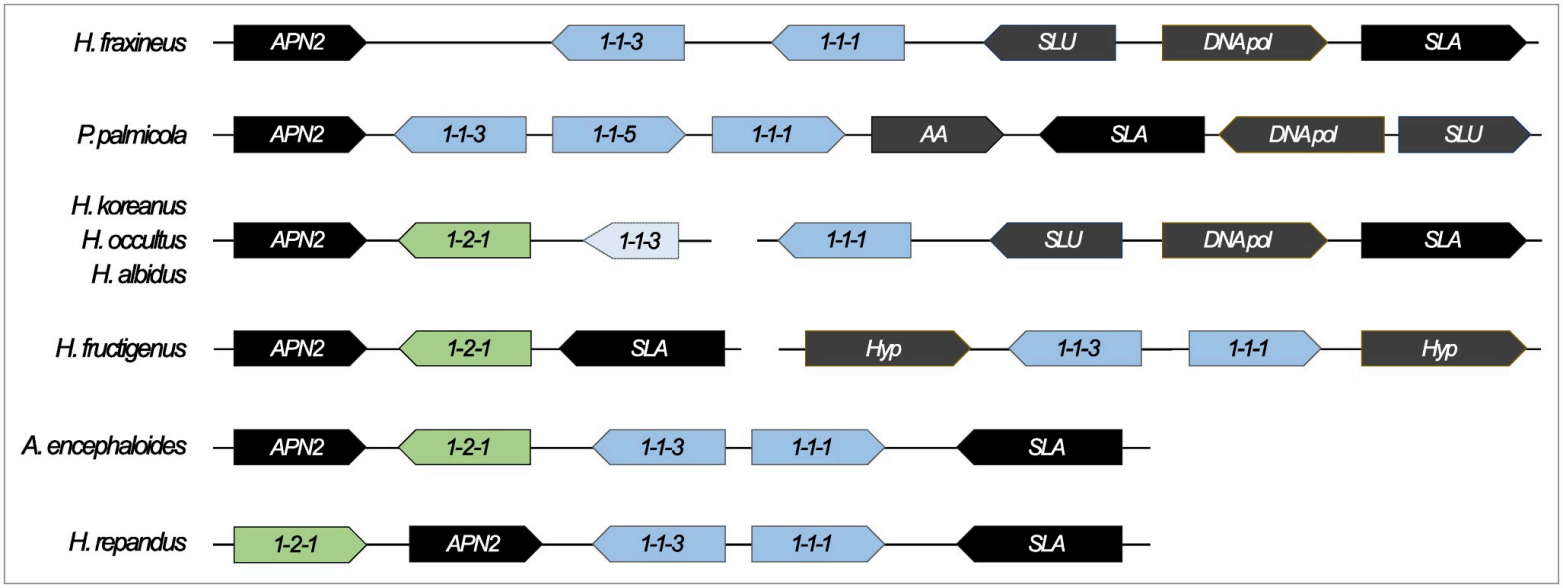
The *MAT* locus structure variety within the Helotiaceae. *Hymenoscyphus fraxineus* and *Pirottaea palmicola* are heterothallic and the representative genomes used in this study possess genes associated only with the *MAT1-1* idiomorph. The remaining species illustrated here are all homothallic, but exhibit different *MAT* locus structures which enable homothallism and thus suggest multiple, independent transitions towards self-fertility. *Hymenoscyphus koreanus*, *Hymenoscyphus occultus*, *and Hymenoscyphus albidus* possess the *MAT1-1-1* and *MAT1-2-1* genes at what is likely a single locus in the genome. However, in all three genomes, the *APN2* and *MAT1-2-1* genes are found on the end of one contig, while the *MAT1-1-1*, *SLU*, *DNA pol* and *SLA* genes are found on the end of a second contig. *Amylocarpus encephaloides* and *Hymenoscyphus repandus* both harbour the *MAT1-1* and *MAT1-2*-associated genes at a single, intact locus in their genomes. In contrast, *Hymenoscyphus fructigenus* possesses two unlinked *MAT* loci, one encoding the *MAT1-2-1* gene and the other encoding the *MAT1-1-1* and *MAT1-1-3* genes. The *MAT1-1*-associated genes are indicated in blue, while the *MAT1-2*-associated genes are indicated in green. APN2: AP endonuclease, Hyp: Hypothetical protein, SLA: cytoskeleton assembly control protein, SLU: pre-mRNA splice factor SLU7-like protein, DNA pol: DNA polymerase, AA: amino acid transporter protein. These gene diagrams are not drawn to scale, but instead aim to illustrate the similarities and differences between the locus structures of these species.

MAT1-1 isolates of the heterothallic species in the Helotiaceae either harbored *MAT1-1-1* and *MAT1-1-3* [e.g. *H*. *fraxineus*, [85]] or *MAT1-1-1*, *MAT1-1-3* and *MAT1-1-5* (e.g. *Pirottaea palmicola*, Fig 3). There also appears to have been at least three independent transitions toward homothallism in this family (Fig 1). The first transition, which likely occurred in the ancestor of *Hymenoscyphus koreanus*, *Hymenoscyphus occultus* and *Hymenoscyphus albidus*, probably occurred due to an unequal crossing over event which brought together the *MAT1-1-1* and *MAT1-2-1* genes together in what is likely a single *MAT* locus (Fig 3). The *MAT1-1-3* gene has been pseudogenised and thus, while a region homologous to the *H*. *fraxineus MAT1-1-3* gene is present at the *MAT* locus of all three homothallic species, it does not code for a putative intact MAT1-1-3 protein sequence. *Hymenoscyphus fructigenus* is also likely homothallic, harboring two unlinked *MAT* loci (Fig 3). While the *MAT1-2-1* gene is associated with the *SLA2* and *APN2* genes as expected, the *MAT1-1-1* and *MAT1-1-3* genes are present elsewhere in the genome, flanked by hypothetical and unknown proteins.

*Amylocarpus encephaloides* also possessed a *MAT* locus structure suggestive of homothallism, harboring the *MAT1-1-1*, *MAT1-1-3* and *MAT1-2-1* genes at a single locus flanked by *SLA2* and *APN2* (Fig 3). The closest relative to *A*. *encephaloides* in accordance to the phylogeny generated for this study, *Hymenoscyphus repandus*, is also homothallic, but exhibits a different *MAT* locus structure from that of *A*. *encephaloides*. *H*. *repandus* possesses the same three *MAT* genes as *A*. *encephaloides*, but while the *MAT1-1-1* and *MAT1-1-3* genes are flanked by the *SLA2* and *APN2* genes, the *MAT1-2-1* gene was found directly upstream of *APN2* (Fig 3).

### A unique mating strategy in *Lachnellula*

**Fig 4:**
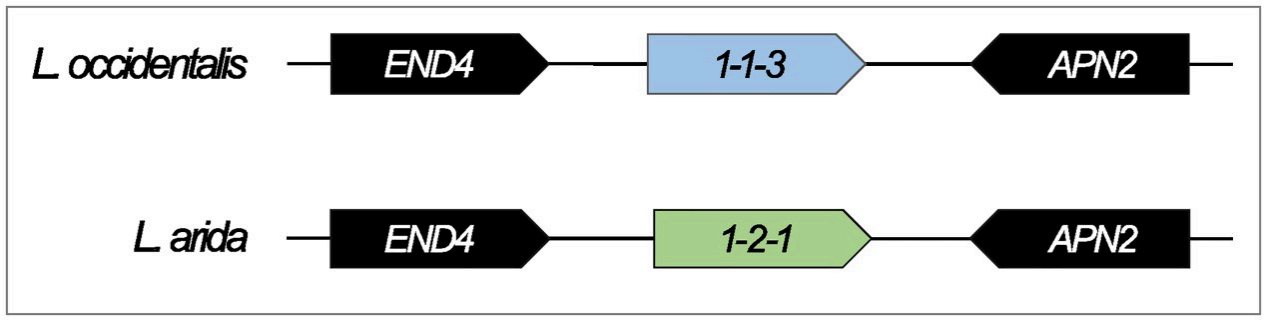
The structure of the *MAT1-1* and *MAT1-2* idiomorphs in *Lachnellula* species. Based on the content of the *MAT* idiomorph in various *Lachnenulla* species, a novel sexual strategy was described. While the MAT1-2 isolates (*L*. *arida*) harbor the *MAT1-2-1* gene as expected, the MAT1-1 isolates (*L*. *subtilissima*, *L*. *occidentalis*, *L*. *willkommii*, *L*. *cervina*, *L*. *suecica)* possessed only the *MAT1-1-3* gene, suggesting that sexual reproduction takes place between compatible mating partners in the absence of the *MAT1-1-1* gene in these species.

Species of *Lachnellula* appeared to exhibit a unique heterothallic-type mating strategy that has not been described previously. Rather than MAT1-1 isolates possessing the *MAT1-1-1* and secondary *MAT1-1* genes, these isolates harbored only the *MAT1-1-3* gene (Fig 4). While a region of the *MAT1-1* locus in these isolates is homologous to the *Cudoniella acicularis MAT1-1-1* gene, an intact gene was not predicted and could not be manually identified using BLAST- or alignment-based analyses. The MAT1-2 isolates possessed the *MAT1-2-1* gene (Fig 4). Both the predicted MAT1-1-3 and MAT1-2-1 proteins possessed the expected HMG domain (cd01389).

### An α-factor pheromone is present in most Leotiomycetes

**Fig 5:**
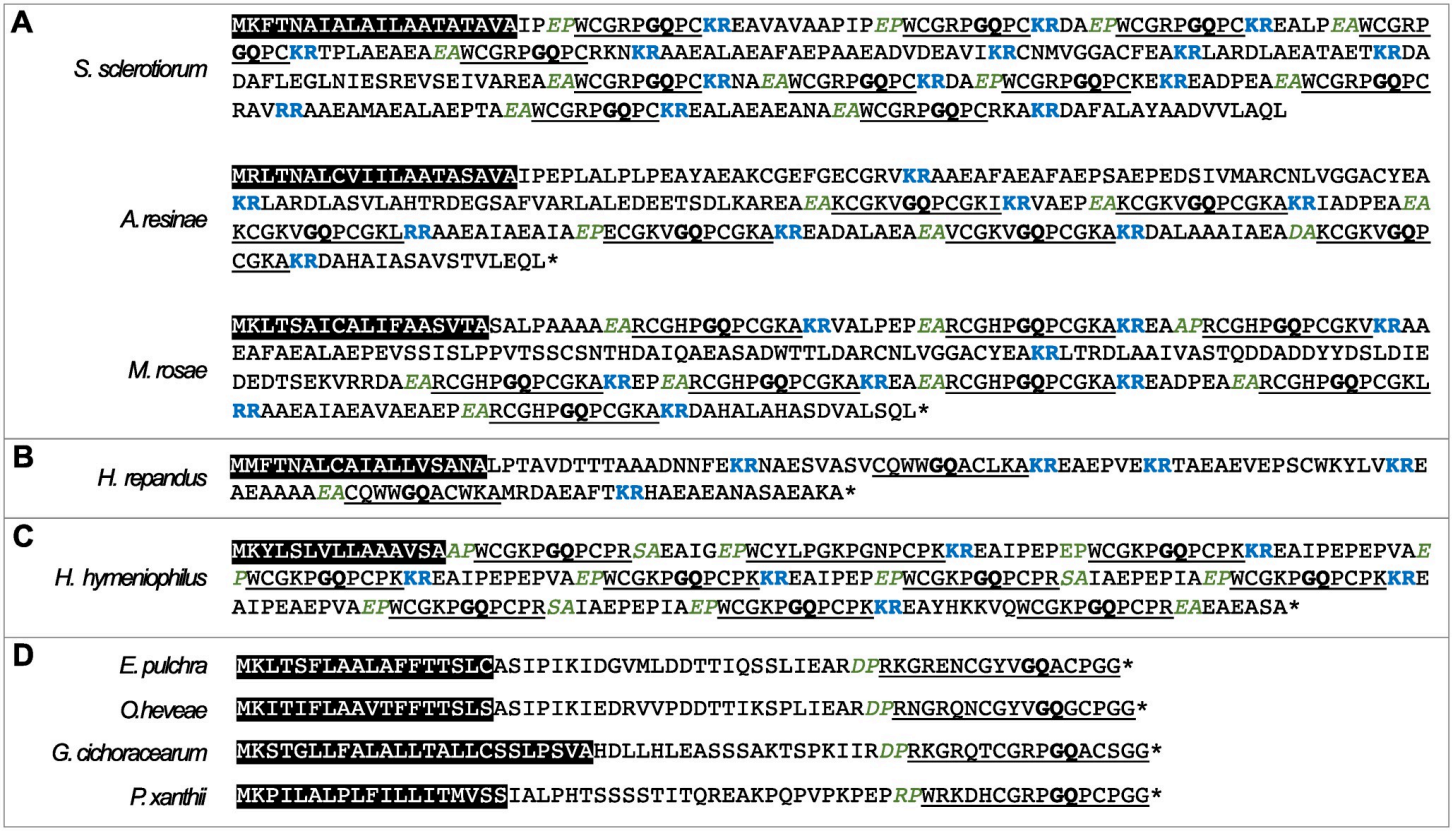
Structural variation of α-factor pheromones in the Leotiomycetes. The predicted signal peptide (white text, black background), STE13 processing sites (green text, italics), KEX1/2 processing sites (blue text, bold), putative mature repeats (underlined), and β-turn dipeptides (bold, underlined) are annotated on each pro-pheromone sequence. A) The α-factor pheromones from a variety of Leotiomycetes that exhibit the typical structure of this mating pheromone. These species all have pheromones with repeating units of the mature pheromone within clearly defined STE13 and KEX1/2 processing sites. B) It was difficult to distinguish the mature repeats in the *H*. *repandus* α-factor pheromone, because the STE13 and KEX1/2 sites did not directly flank putative repeats. Instead, the GQ motif was used to annotate putative mature repeats. C) The α-factor pheromone from *H*. *hymeniophilus* appears to rely more heavily on STE13 processing, as KEX1/2 processing sites were not present after every repeat. D) The α-factor pheromones from selected Erysiphaceae species that exhibit an atypical structure of this mating pheromone. This gene produced a protein with a single mature pheromone sequence and no KEX1/2 processing sites.

Putative α-factor pheromone genes were identified in 115 of the 124 species included in this study (S3 Table, S8 and S9 Files). Of those, 104 were recognizable as typical α-factor pheromones, with KEX1/2 and STE13 processing sites and multiple repeats of a mature pheromone peptide (Fig 5). These pheromones varied in length from 89 aa with only 2 mature repeats (*Oidiodendron maius*) to 446 aa with 18 mature repeats (*Botrytis fragariae*). Additionally, the vast majority of mature repeats had either a GQ or PG dipeptide, a sequence that facilitates the β-turn necessary for the functioning of this pheromone in *F*. *oxysporum* and *S*. *cerevisiae*, respectively [86].

Two species harbored α-factor pheromone genes which, while recognizable, were divergent in terms of sequence and/or structure (Fig 5). One of these species, *Hymenoscyphus repandus*, had a signal peptide and recognizable KEX1/2 and STE13 processing sites but the mature pheromone peptide sequences were difficult to distinguish. There were two GQ dipeptides that were used to identify the putative mature peptides, but in both cases, the processing sites weren’t proximal to the repeats. Additionally, *Hyphodiscus hymeniophilus* exhibited characteristics associated with the α-factor pheromone, however, fewer KEX1/2 processing sites were present than expected. Instead, putative STE13 sites were found after numerous repeats, suggesting that this species might rely more heavily on STE13-mediated processing.

Nine species accommodated in the Erysiphaceae possessed divergent α-factor pheromones (Fig 5). This divergent gene, identified using the pheromone’s flanking genes from *Blumeriella jaapii*, encoded for a putative protein that was structurally different to the other α-factor pheromones in that it did not harbor multiple repeats of a mature pheromone peptide or recognizable KEX1/2 and STE13 processing sites. However, each of the nine homologs encoded proteins harboring a signal peptide, a single potential mature peptide containing a GQ dipeptide, and a putative STE13 processing site (DP, VP, GP or EP). It is unclear whether this protein represents a pre-protein that requires further processing, analogous to the typical α-factor pheromone, or if this is the mature pheromone factor. Neither a typical nor an atypical α-factor could be identified from the other five Erysiphaceae species.

### A putative a-factor pheromone discovered in the Leotiomycetes

A number of potential a-factor pheromones were identified in the genomes of *B*. *cinerea* (4 genes), *Botrytis sinoalli* (5 genes), *S*. *sclerotiorum* (11 genes), *Monilinia fructicola* (12 genes), *Rhynchosporium commune* (5 genes), *Amorphotheca resinae* (2 genes), and *P*. *destructans* (2 genes). These genes each encoded small proteins (≤100 amino acids) that terminated in a CAAX or CPAX domain (S3 File). These proteins were subsequently used as queries in tBLASTn searches against the genomes of closely related species. For example, the four genes from *B*. *cinerea* and five genes in *B*. *sinoalli* were used to identify homologs from the genomes of the remaining *Botrytis*/*Botryotinia* species.

In the *B*. *cinerea* comparisons, only three of the proteins were consistently found in the other *Botrytis* genomes, while in the *M*. *fructicola* comparisons, only two proteins were consistently present in the other *Monilinia* genomes. Similar results were obtained for the other species comparisons. When these proteins were used as tBLASTn queries against the genomes of more distantly related species, it was apparent that the most promising candidate gene was *B*. *cinerea* BCIN_13g03330. Homologs of this gene were found to be present in at least 111 of the species included in this study (S3 Table, S10 File). While this gene was not annotated in most other genomes, a common characteristic of pheromone genes, it was detected and annotated manually.

The putative a-factor pheromone ranged from 45 (*Antarctomyces pellizariae*) to 64 (*Rhynchosporium agropyri*) amino acids in length and most frequently terminated in a CTVM domain, although CVVM, CIVM, CTIL, CSIM, CSVM were also present in a few species (Table 1, S10 File). Further evidence supporting the view that this protein is the a-factor pheromone was the serine (S) and threonine (T) rich N-terminal of the protein, which is also common in the a-factor pheromones of many of the Sordariomycetes (Table 1). Additionally, a glycine-tyrosine (GY) motif was present near the C-terminal of 87 of the Leotiomycete putative a-factors, residues that are conserved across in the Sordariomycete a-factors as well.

**Table 1:** The putative Leotiomycete a-factor pro-pheromone compared with various Sordariomycete a-factor pheromones. The serine and threonine rich regions are indicated in grey and italics, the glycine-tyrosine motif is indicated in white text and highlighted in black and the conserved CPAX/CAAX domain is underlined.

Sordariomycetes	*C*. *globosum*	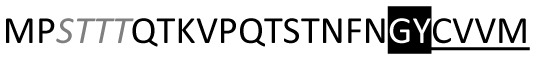
	*P*. *anserina*	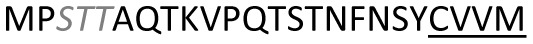
	*N*. *crassa*	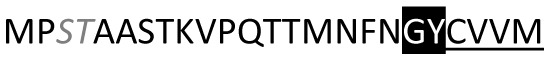
	*S*. *macrospora*	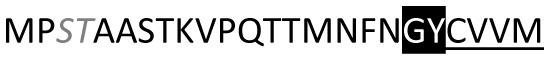
	*G*. *zeae*	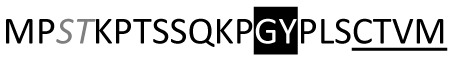
	*H*. *abstrusa*	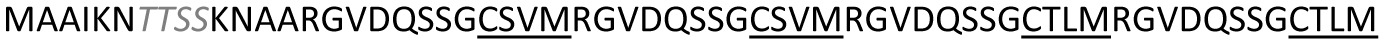
	*F*. *oxysporum*	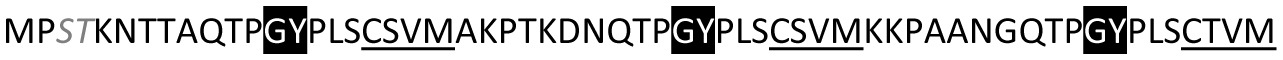
Leotiomycetes	*B*. *cinerea*	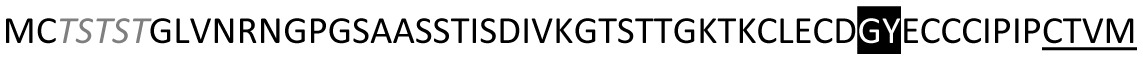
	*C*. *homoeocarpa*	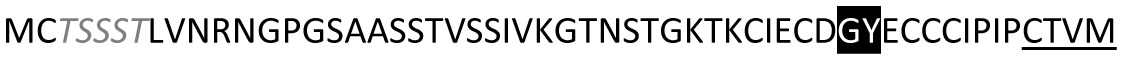
	*R*. *agropyri*	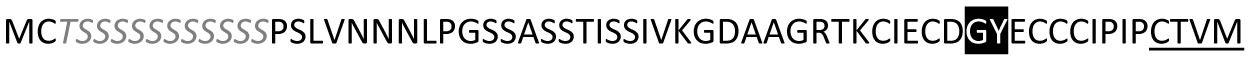
	*M*. *coronaria*	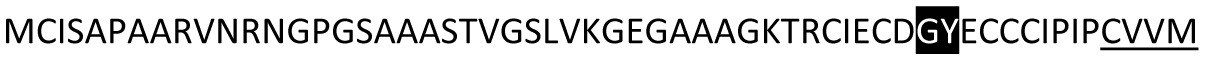
	*A*. *macrosclerotiorum*	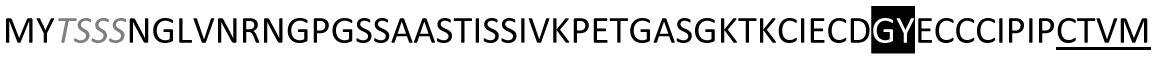
	*B*. *hordei*	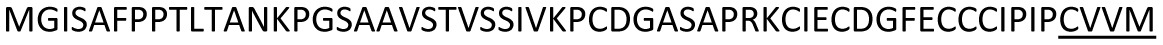
	*H*. *fructigenus*	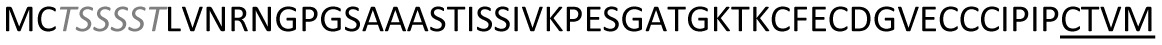
	*L*. *hyalina*	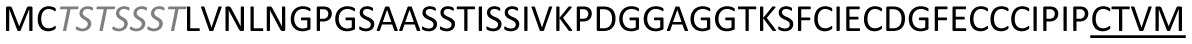

### Diversity of the α- and a-factor pheromones

There was low inter-species, intra-family diversity for the α-factor pheromone. In many of the families considered here, species harbored identical copies of the mature repeat of the α-factor pheromone. In the Sclerotiniaceae (n = 36), 31 species possessed the mature repeat WCGRPGQPC and only nine of the species had additional unique repeats within the same pro-pheromone (S3 Table). Similar patterns were also identified in the Helotiaceae, where the most common repeats were CKYVGQGCW and CKYAGQGCW. Additionally, species of the Ploettnerulaceae, Drepanopezizaceae, and Mollisiaceae (n = 14) had a number of repeats in common, with RCGHPGQPCG and RCGYPGQPCG occurring in seven and five species, respectively. Lachnaceae species (n = 8) also possessed many repeats in common with one another, with seven harboring the RCGARGQGCW repeat.

In contrast to the conservation seen in most other families, Hyaloscyphaceae species (n = 5), tended to possess repeats unique to each species. This was typically due to the first and last amino acids of the repeats, which were highly variable. Additionally, in families that were represented by a single species, the species frequently possessed unique α-factor pheromone repeats. Examples include *Chlorencoelia torta* (Cenangiaceae), *Venustampulla echinocandica* (Pleuroascaceae), and *A*. *pellizariae* (Thelebolaceae).

Higher levels of diversity were observed when the putative a-factor pro-pheromone was considered. In general, the C-terminal of the a-factor pro-proteins tended to be conserved within families, while the N-terminal frequently exhibited higher levels of divergence (S4 Fig). In contrast, species of the Sclerotiniaceae and the Rutstroemiaceae harbored highly conserved a-factor pheromones, with 19 of the 20 *Botrytis* species and four of the six *Monilinia* species possessing identical a-factor pheromones (S5 Fig).

### Pheromone expression is not mating type-dependent in three Leotiomycete species

RNAseq datasets from three heterothallic species, *M*. *fructicola*, *B*. *cinerea*, and *A*. *resinae* were used to determine whether the two putative mating pheromones were expressed in a mating type-dependent manner in the Leotiomycetes (S4 Table). Both MAT1-1 and MAT1-2 datasets were available for *B*. *cinerea* and *M*. *fructicola*, while a MAT1-1 dataset was available for *A*. *resinae*. There was no evidence for MAT1-1 exclusive expression of the α-factor nor MAT1-2 exclusive expression of the putative a-factor in these three species.

In *B*. *cinerea*, both mating pheromones were expressed by the individual MAT1-1 isolate as well as in mixed mating type material made up of sclerotia, apothecium primorida, and/or ascospores (S4 Table). The putative a-factor pheromone was expressed at much higher levels than the α-factor pheromone in all four datasets. The α-factor was expressed at its highest in the apothecium primordia (RPKM = 11.45) and its lowest in the sclerotia (RPKM = 1.47). In contrast, the putative a-factor was expressed at its highest in the sclerotia (RPKM = 198.08) and its lowest in the ascospores (RPKM = 46.28).

In *M*. *fructicola*, expression of the α-factor pheromone was very low (RPKM < 1.00), regardless of mating type and tissue type (S4 Table). The expression of the *M*. *fructicola* putative a-factor was undetectable (RPKM = 0.00). Lastly, the MAT1-1 isolate of *A*. *resinae* expressed the α-factor at a very low level (RPKM < 1.00) while the putative a-factor was more highly expressed (RPKM = 19.58).

The *MAT* genes were also expressed at very low levels in all three species (S4 Table), suggesting that the conditions under which these isolates were grown prior to RNA extraction were not necessarily conducive to the expression of mating-related genes.

### Pheromone receptors

Genes encoding both the α-factor receptor (*ste2*) and a-factor receptor (*ste3*) were identified in the majority of the species included in this study. Furthermore, most of these genes encoded proteins with the expected seven transmembrane domains as well as the STE2 (pfam02116) and STE3 (pfam02076) functional domains. There were a number of exceptions that are worth noting, where the gene encoding either of the two receptors has been disrupted and the putative protein is not predicted to contain the expected domains or has been entirely deleted from the genome.

#### The α-factor receptor (STE2)

A total of 117 species harbored an intact *ste2* gene that encoded proteins with seven predicted transmembrane domains. In *H*. *koreanus*, *H*. *occultus*, *Hymenoscyphus scutula*, *H*. *repandus*, *Pezicula neosporulosa*, and *Cudoniella acicularis*, functional seven transmembrane domain-encoding *ste2* genes could not be annotated. This was despite the fact that these species do encode detectable α-factor pheromones. In both *H*. *koreanus* and *H*. *occultus*, a gene encoding a STE2-domain protein is present, but the encoded proteins only had six predicted transmembrane domains (S6 Fig). In *H*. *scutula*, the 5’ region of the *ste2* gene was not identifiable and the remaining region of the gene does not encode a protein with seven transmembrane domains. The α-factor pheromone receptor was entirely deleted in *H*. *repandus* and could not be annotated in *C*. *acicularis*. Lastly, a gene encoding this receptor could not be annotated in *Calycina marina*, a species that also does not possess a gene encoding the α-factor pheromone.

#### The a-factor receptor (STE3)

A total of 112 species possessed *ste3* genes that encoded seven-transmembrane domain proteins. In a further nine species, *ste3* gene regions were identified using various BLAST searches, but functional protein-encoding genes could not be annotated for various reasons. For example, there were in frame, premature stop codons in the STE3-encoding genes of *Monilinia fructigena*, *Rutstroemia* sp. NJR-2017a BBW, *Neobulgaria alba*, and *A*. *sarcoides*. In *Rutstroemia* sp. NJR-2017a WRK4, *Rutstroemia* sp. NJR-2017a BVV2, *V*. *echinocandica* and *C*. *acicularis*, too many transmembrane domains were predicted in each of the encoded proteins. Furthermore, a *ste3* gene could not be annotated in *A*. *pellizariae* due to a lack of sequence similarity. Finally, the *ste3* gene was entirely deleted in three species; *Hyaloscypha variabilis*, *Hyaloscypha hepaticicola* and *Hymenoscyphus fructigenus*. Interestingly, of all 12 species that do not code for functional STE3 proteins, only *N*. *alba* did not harbor an a-factor pheromone gene.

## Discussion

In this study, we exploited a large number of publicly available genomic and transcriptomic resources in an effort to elucidate and characterize the mating strategies and behaviors of more than 100 Leotiomycete species. This led to the discovery of a unique mating strategy in the *Lachnellula* and the first identification of a putative Pezizomycotina a-factor mating pheromone outside of the Sordariomycetes. It also revealed the presence of numerous transitions between homothallism and heterothallism in the Leotiomycetes.

Transitions between heterothallism and homothallism are well documented across both the Ascomycota [17, 87–89] and the Basidiomycota [90, 91]. By elucidating the *MAT* loci in the majority of the species considered here, it was possible to identify numerous, independent transitions from heterothallism to homothallism in the Leotiomycetes. Furthermore, this provided evidence to support a heterothallic ancestor for this important Class of fungi. The evolution towards self-fertility is a common pattern in the Ascomycota, where heterothallism is believed to be the ancestral state of numerous clades, including *Neurospora* [89], *Aspergillus* [92], the Mycosphaerellales [88], and various budding yeasts [93]. Given the potential benefits of outcrossing and the costs associated with continuous inbreeding, this shift towards homothallism may have significant consequences in terms of species longevity.

It has been hypothesized that the ancestor of the Sclerotiniaceae was homothallic [83]. Heterothallism was by far the most common sexual strategy in this family and thus, in contrast to previously published work, our ancestral state reconstruction suggested that the ancestor of the Sclerotiniaceae was heterothallic. However, a homothallic ancestor is well-supported by the structure of the *MAT* loci of the heterothallic species in this family, including those specifically accommodated in the *Botrytis* and *Monilinia* genera [83, 94], which typically have *MAT* genes fragments in their idiomorphs. This structure most likely evolved from a homothallic ancestor that harbored all four *MAT* genes and subsequently underwent DNA loss at this locus to produce two compatible idiomorphs [83]. Thus, despite the predominance of heterothallism in this group of fungi, it is far more likely that the Sclerotiniaceae evolved from a homothallic ancestor. This example emphasizes the need to consider the structure of the *MAT* locus in addition to the gene content of the locus when assigning sexual strategies and investigating the evolution of sexual behaviors in fungi.

One of the difficulties associated with predicting mating behavior from genome data is that mixed mating type cultures are sometimes sequenced. In these cases, individuals of both the MAT1-1 and MAT1-2 mating type are included and the genome sequence will thus possess genes associated with both mating types [15]. This can lead to the incorrect conclusion that a species is homothallic when it is in fact heterothallic. Under these circumstances, one of the idiomorphs is typically assembled as usual and is flanked by genes that are associated with the *MAT* locus. The second idiomorph is then usually assembled on its own contig, possessing only the genes from this idiomorph and no flanking genes. This was the case for a number of the genomes considered in the present study and it highlights the importance of conducting genome sequencing on isolates representing single genotypes.

It is possible to use raw sequence reads to predict whether genomes harboring multiple *MAT* genes assembled into individual contigs represent single or mixed isolates. By mapping these reads to the genome assembly, regions where SNPs are present at a frequency higher than the average error rate of the sequencing technology used can be identified. When more SNPs than expected are identified in these mappings, the representative genome would most likely have been generated from a mixed isolate. In contrast, if few SNPs are present, the genome was most likely produced from a single isolate. Unfortunately, such raw data were not available for the specific isolates used for genome sequencing in the species for which this would have been useful.

One of the most surprising results of this study was the identification of a novel mating strategy in species of *Lachnellula*. Both the MAT1-1 and MAT1-2 mating types were represented in the dataset, but while the MAT1-2 isolates harbored the *MAT1-2-1* gene as expected, the MAT1-1 isolates possessed only the secondary *MAT1-1-3* gene and thus lacked the primary *MAT1-1-1* gene. This implies that both mating partners encoded only HMG domain proteins and that the interaction between these two proteins may be sufficient for sexual development in this genus, where sexual structures (apothecia) are known to occur [95]. While this system may be unique in the Pezizomycotina, a similar mating system is known in the Mucorales (Zygomycota) and possibly in the Microsporidia [96, 97]. In these early diverging fungi, the two compatible *MAT* idiomorphs each harbor an allelic HMG protein. Furthermore, HMG proteins are known to be important transcription factors, with a network of HMG proteins controlling the sexual cycle in Sordariomycete species such as *P*. *anserina* [98]. Given the importance of HMG proteins in such a diverse assemblage of fungi, it is possible that there has been a rewiring of the genetic pathways that control sexual development in the *Lachnellula* species. This would lead to a dependence on HMG proteins and the subsequent loss of the MAT1-1-1 α-box protein. Unfortunately, there is a lack of research specifically focusing on sexual reproduction in *Lachnellula* species and additional mating studies need to be conducted to determine whether these partners are indeed compatible.

We were able to identify a putative a-factor pheromone in the Leotiomycetes; the first time this class of pheromone has been identified from non-Sordariomycete Pezizomycotina. The presence of other genes from the pheromone response pathway and widespread heterothallism in the Leotiomycetes suggested that the a-factor pheromone would also be present. However, while efforts have been made to identify the a-factor pheromone in other non-Sordariomycete classes, it has not previously been discovered [36, 99]. Using BLAST-independent methods, we were able to identify a protein that shares a number of characteristics with known Sordariomycete a-factor pheromones, including a serine and threonine rich N-terminal, a conserved glycine-tyrosine motif as well as a C-terminal CAAX/CPAX domain.

The function of the gene that has been identified as the putative a-factor pheromone in the Leotiomycetes is unclear. The fact that components of the pheromone response pathway, including the α-factor pheromone and both pheromone receptors (STE2 and STE3), are conserved across the Ascomycota, and specifically within the Leotiomycetes, suggests that the putative a-factor pheromone is involved in mating. This would be in a manner comparable to the role of the a-factor pheromone in the Sordariomycetes. However, there are a number of lines of evidence that strongly suggest that this is not the case.

The most notable line of evidence suggesting that the identified a-factor gene is not involved in mating in the Leotiomycetes, is the fact that the two pheromones were not expressed in a mating type-dependent manner in the three distantly related heterothallic species investigated here. While these three species may not be representative of all the Leotiomycetes, this does indicate that, at least in certain species, these proteins do not act in the same manner as their Sordariomycete homologs, which are expressed strictly according to mating type to facilitate compatible mating partner interactions. Furthermore, functional characterization studies in *S*. *sclerotiorum* show that the expression of the α-factor gene is significantly altered in *ΔMAT1-1-1*, *ΔMAT1-1-5*, *ΔMAT1-2-1*, and *ΔMAT1-2-10* mutants, further suggesting a mating type-independent control of this pheromone [100]. In the absence of mating type-dependent expression, it is unclear how these peptides would be capable of aiding opposite mating type partner recognition and attraction.

The high level of intra-family conservation of the α-factor pheromone as well as the high level of conservation of the a-factor in the Sclerotiniaceae and Rutstroemiaceae also provides evidence against the utility of these proteins as mating pheromones in the classical sense. This contradicts the hypothesis that the pheromones act as signaling molecules that aim to specifically attract opposite mating partners of the same species [101]. The fact that so many species share identical mature α-factor repeats would make it impossible for individuals to distinguish suitable mating partners from individuals of a different species. A similar conundrum was acknowledged by Martin et al (2011), who noted that pheromone proteins were highly conserved within the *Fusarium fujikuroi* species complex (previously the *Gibberella fujikuroi* species complex) [38]. While there may be some post-fertilization hybridization barriers in these species, such that the pheromones would not be the only factor involved in species discrimination, this level of protein conservation at both genus and family level is surprising in proteins that are generally thought to act as partner attractants. To the best of our knowledge, this is unique amongst the Ascomycota.

The widespread occurrence and conservation of the two pheromones throughout the Leotiomycetes, implies that they play important roles in the lifecycles of these species. In other fungi, pheromones and their receptors have been co-opted for new functions, both related and unrelated to mating. For example, in *N*. *crassa*, the a-factor pheromone is thought to act as a conglutination factor that cements hyphae together during the production of the ascoma [102]. Additionally, a-factor *N*. *crassa* mutants exhibit defects associated with vegetative growth, suggesting that the pheromone may also play an unknown role in filamentous differentiation [102]. More recent research has shown that *Fusarium oxysporum* also utilizes components of the pheromone response pathway for a variety of non-mating functions [103, 104]. This form of functional evolution may explain why the pheromone response pathway is conserved in Leotiomycetes, despite the fact that it may not operate as a partner signaling pathway.

There are limitations to the method used in this study to identify this putative pheromone factor. Firstly, our BLAST-independent method relied on the availability of well-annotated genomes, because the predicted protein lists that were filtered to identify the putative a-factor pheromone proteins were produced directly from the CDS annotations. These annotations are the result of *in silico*-based predictions and may not be fully accurate. However, to overcome this limitation, the annotations from seven genomes were used to create as comprehensive a list as possible from which to filter out non-target proteins. Secondly, short genes, such as pheromones, are often not annotated during standard annotation pipelines. Thus, while genes encoding proteins as short as 35 aa were annotated in, for example, the *S*. *sclerotiorum* genome, it is possible that shorter proteins may have been missed prior to filtering. A BLAST- and annotation-independent method is currently being developed and aims to solve these limitations for future studies.

The identification of numerous, complex genes across the Leotiomycetes illustrated the lack of high-quality gene models in many publicly available genomes. This was particularly true in species for which limited non-genomic NGS data are available (i.e.: RNA-seq data) and was specifically evident when the genes encoding the two pheromone receptors were considered. Both receptors typically have numerous intron/exon boundaries and produce proteins with a very strict structure/function relationship. The structure of these proteins contributes directly to their functioning. The extracellular N-terminal, transmembrane domains, and intracellular C-terminal are essential for signal recognition, anchoring of the receptors, and transmitting the intracellular signal, respectively. This strong relationship made identifying misannotated genes possible as each encoded protein could be assessed *in silico* for these structural domains. Thus, while currently available genome annotation pipelines are useful for the identification of the genomic location of a particular gene, it is often necessary to manually curate the annotations, using well-characterized gene sequences from closely related species or transcriptomic data.

## Conclusions

The Leotiomycetes accommodates an incredibly diverse group of fungi, including well-known plant and animal pathogens as well as species important for various biotechnological applications. Sexual reproduction in this group of fungi is poorly understood. However, recent advances in sequencing technologies made it possible in this study to compare the genomes of 124 Leotiomycete species to determine whether key genes involved in sexual reproduction were present and conserved. This led to the identification of a putative a-factor pheromone, the discovery of a unique mating strategy, and evidence supporting heterothallism as the ancestral mating strategy of these fungi. Other than these important new insights, this study provides a rich database from which future functional studies can be conducted on these ecologically and economically relevant fungi.

## Supporting information

S1 Fig Determining the length of the mature repeat in the α-factor pheromone using *Hymenoscyphus occultus* as an example. (PPTX)

S2 Fig Comparison between the maximum likelihood tree generated using a concatenated supermatrix of 72 genes and the maximum likelihood supertree generated using the 72 individual gene regions combined using ASTRAL. (PPTX)

S3 Fig Ancestral state reconstruction of the Leotiomycete mating strategies. (PPTX)

S4 Fig The diversity of the Leotiomycete a-factor pheromone in the Ploettnerulaceae, Drepanopezizaceae, Mollisiaceae, Erysiphaceae, Hyaloscyphaceae, Helotiaceae and Lachnaceae. (PPTX)

S5 Fig The conservation of the Sclerotiniaceae and Rutstroemiaceae a-factor pheromones. (PPTX)

S6 Fig Gene alignments and RNAseq mappings of the *ste2* gene from *Hymenoscyphus* species. (PPTX)

S1 Table Species included in this study, with NCBI Genome and RNAseq accession numbers. (XLSX)

S2 Table NCBI accession numbers for the various gene and protein sequences used in BLASTn and tBLASTn analyses. (XLSX)

S3 Table Presence and absence of both pheromone factors and additional functional information of the α-factor pheromone. (XLSX)

S4 Table Expression of housekeeping genes, *MAT* genes, pheromone genes and pheromone receptor genes. (XLSX)

S1 File Genomes that were excluded from the analyses. (PDF)

S2 File A pheromone sequences from a variety of Sordariomycetes & yeasts. (PDF)

S3 File CAAX/CpAX proteins (<100aa) from *B*. *cinerea*, *B*. *sinoalli*, *S*. *sclerotiorum*, *M*. *fructicola*, *R*. *commune*, *A*. *resinae* and *P*. *destructans*. (PDF)

S4 File Character state statistics from the ancestral state reconstruction analyses. (PDF)

S5 File *MAT1-1-13* sequences from *Rhynchosporium* species. (PDF)

S6 File Species that were classified as “undetermined” with regards to their sexual strategy. (PDF)

S7 File Explanations regarding the sexual strategy determinations in species with both *MAT1-1-* and *MAT1-2*-associated genes. (PDF)

S8 File Alpha pheromone sequences for all Leotiomycetes (GBK). (PDF)

S9 File Alpha pheromone sequences for all Leotiomycetes (FASTA). (PDF)

S10 File A pheromone sequences for all Leotiomycetes. (PDF)
