# Supplementary material for: Needles in fungal haystacks: Discovery of a putative a-factor pheromone and a unique mating strategy in the Leotiomycetes
Source: PLoS One. 2023 Oct 12;18(10):e0292619. doi: 10.1371/journal.pone.0292619 (PMC10569646; doi:10.1371/journal.pone.0292619)
Supplement: S1 Fig — (PPTX) [file pone.0292619.s001.pptx]

## Slide 1
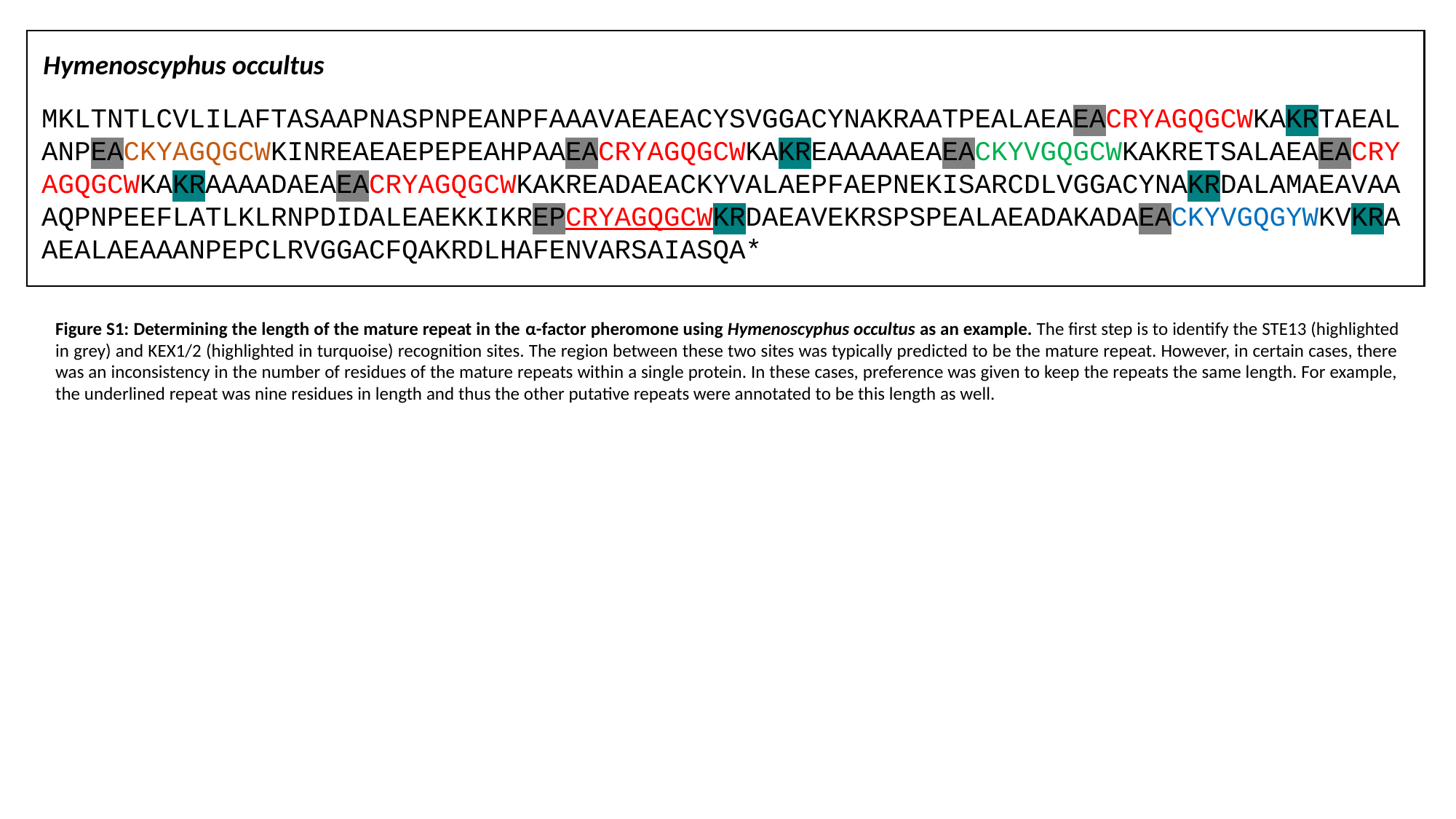

Hymenoscyphus occultus
MKLTNTLCVLILAFTASAAPNASPNPEANPFAAAVAEAEACYSVGGACYNAKRAATPEALAEAEACRYAGQGCWKAKRTAEALANPEACKYAGQGCWKINREAEAEPEPEAHPAAEACRYAGQGCWKAKREAAAAAEAEACKYVGQGCWKAKRETSALAEAEACRYAGQGCWKAKRAAAADAEAEACRYAGQGCWKAKREADAEACKYVALAEPFAEPNEKISARCDLVGGACYNAKRDALAMAEAVAAAQPNPEEFLATLKLRNPDIDALEAEKKIKREPCRYAGQGCWKRDAEAVEKRSPSPEALAEADAKADAEACKYVGQGYWKVKRAAEALAEAAANPEPCLRVGGACFQAKRDLHAFENVARSAIASQA*
Figure S1: Determining the length of the mature repeat in the α-factor pheromone using Hymenoscyphus occultus as an example. The first step is to identify the STE13 (highlighted in grey) and KEX1/2 (highlighted in turquoise) recognition sites. The region between these two sites was typically predicted to be the mature repeat. However, in certain cases, there was an inconsistency in the number of residues of the mature repeats within a single protein. In these cases, preference was given to keep the repeats the same length. For example, the underlined repeat was nine residues in length and thus the other putative repeats were annotated to be this length as well.
