# Supplementary material for: Needles in fungal haystacks: Discovery of a putative a-factor pheromone and a unique mating strategy in the Leotiomycetes
Source: PLoS One. 2023 Oct 12;18(10):e0292619. doi: 10.1371/journal.pone.0292619 (PMC10569646; doi:10.1371/journal.pone.0292619)
Supplement: S2 Fig — (PPTX) [file pone.0292619.s002.pptx]

## Slide 1
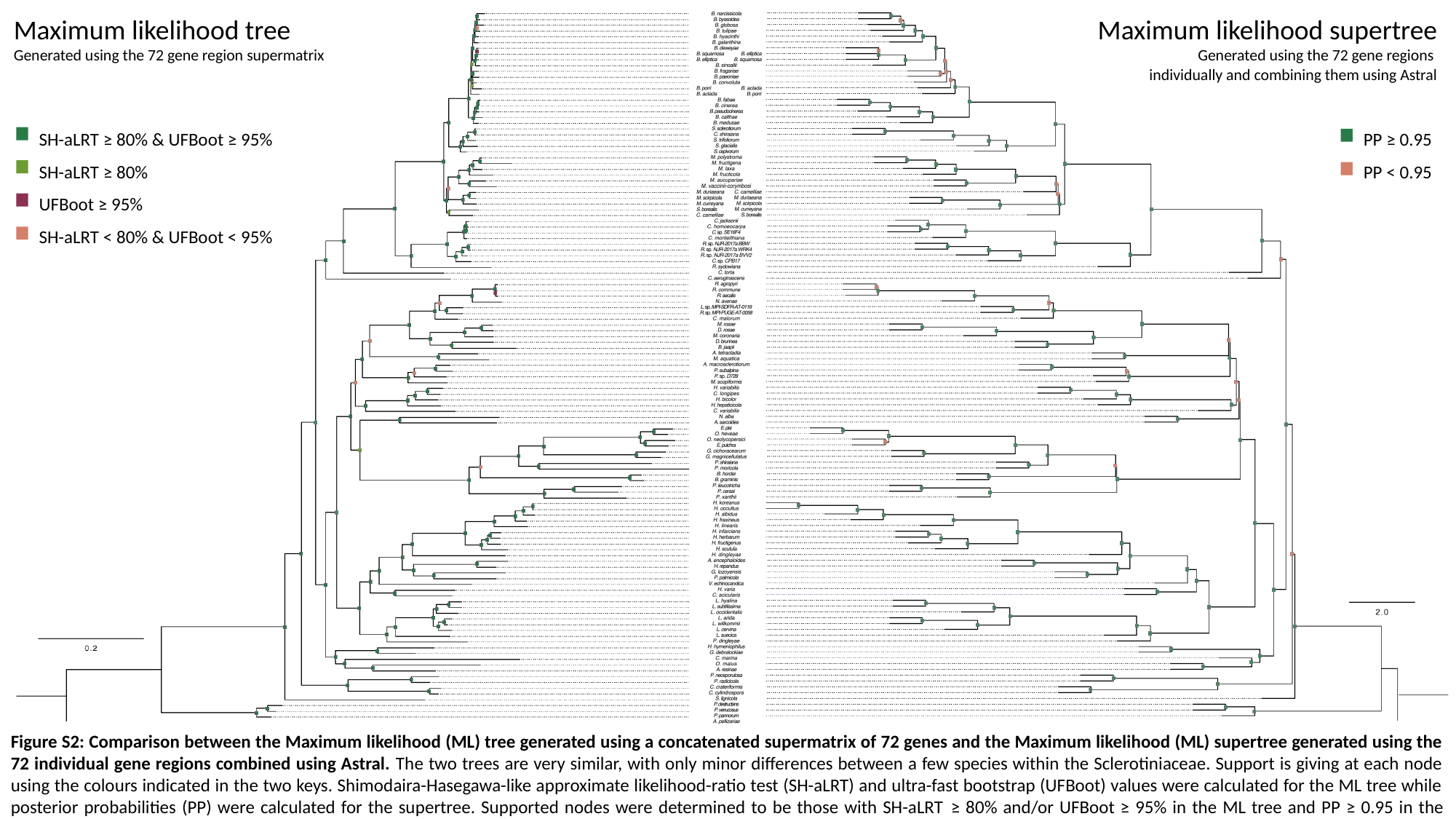

Maximum likelihood tree
Generated using the 72 gene region supermatrix
Maximum likelihood supertree
Generated using the 72 gene regions
individually and combining them using Astral
SH-aLRT ≥ 80% & UFBoot ≥ 95%
SH-aLRT ≥ 80%
UFBoot ≥ 95%
SH-aLRT < 80% & UFBoot < 95%
PP ≥ 0.95
PP < 0.95
Figure S2: Comparison between the Maximum likelihood (ML) tree generated using a concatenated supermatrix of 72 genes and the Maximum likelihood (ML) supertree generated using the 72 individual gene regions combined using Astral. The two trees are very similar, with only minor differences between a few species within the Sclerotiniaceae. Support is giving at each node using the colours indicated in the two keys. Shimodaira-Hasegawa-like approximate likelihood-ratio test (SH-aLRT) and ultra-fast bootstrap (UFBoot) values were calculated for the ML tree while posterior probabilities (PP) were calculated for the supertree. Supported nodes were determined to be those with SH-aLRT ≥ 80% and/or UFBoot ≥ 95% in the ML tree and PP ≥ 0.95 in the supertree. The ML tree generated using the 72 gene region supermatrix was used for the ancestral state reconstruction and is visualized in Figures 1 and S3.
