# Supplementary material for: Needles in fungal haystacks: Discovery of a putative a-factor pheromone and a unique mating strategy in the Leotiomycetes
Source: PLoS One. 2023 Oct 12;18(10):e0292619. doi: 10.1371/journal.pone.0292619 (PMC10569646; doi:10.1371/journal.pone.0292619)
Supplement: S3 Fig — (PPTX) [file pone.0292619.s003.pptx]

## Slide 1
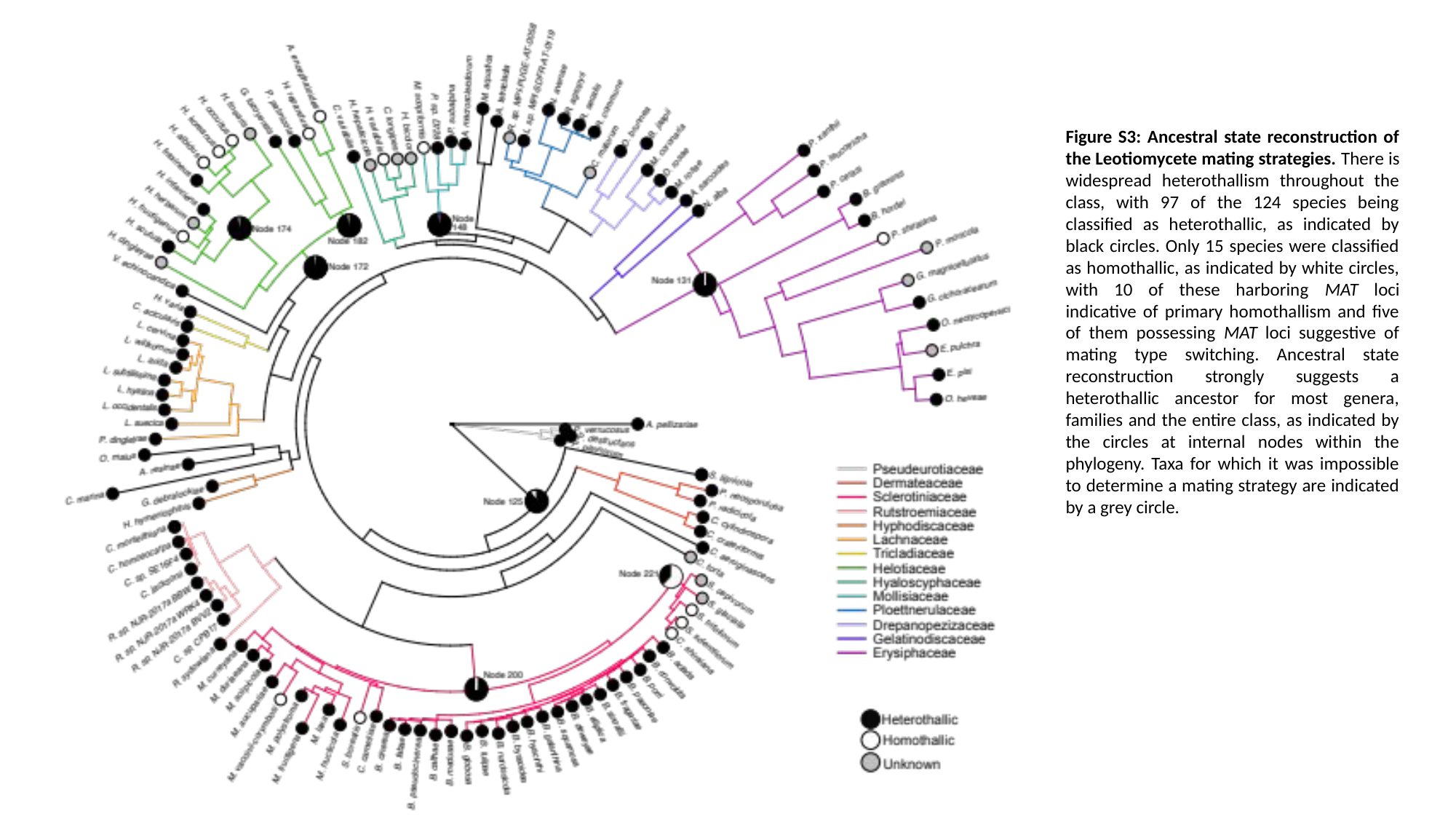

Figure S3: Ancestral state reconstruction of the Leotiomycete mating strategies. There is widespread heterothallism throughout the class, with 97 of the 124 species being classified as heterothallic, as indicated by black circles. Only 15 species were classified as homothallic, as indicated by white circles, with 10 of these harboring MAT loci indicative of primary homothallism and five of them possessing MAT loci suggestive of mating type switching. Ancestral state reconstruction strongly suggests a heterothallic ancestor for most genera, families and the entire class, as indicated by the circles at internal nodes within the phylogeny. Taxa for which it was impossible to determine a mating strategy are indicated by a grey circle.
