# Supplementary material for: Needles in fungal haystacks: Discovery of a putative a-factor pheromone and a unique mating strategy in the Leotiomycetes
Source: PLoS One. 2023 Oct 12;18(10):e0292619. doi: 10.1371/journal.pone.0292619 (PMC10569646; doi:10.1371/journal.pone.0292619)
Supplement: S4 Fig — (PPTX) [file pone.0292619.s004.pptx]

## Slide 1
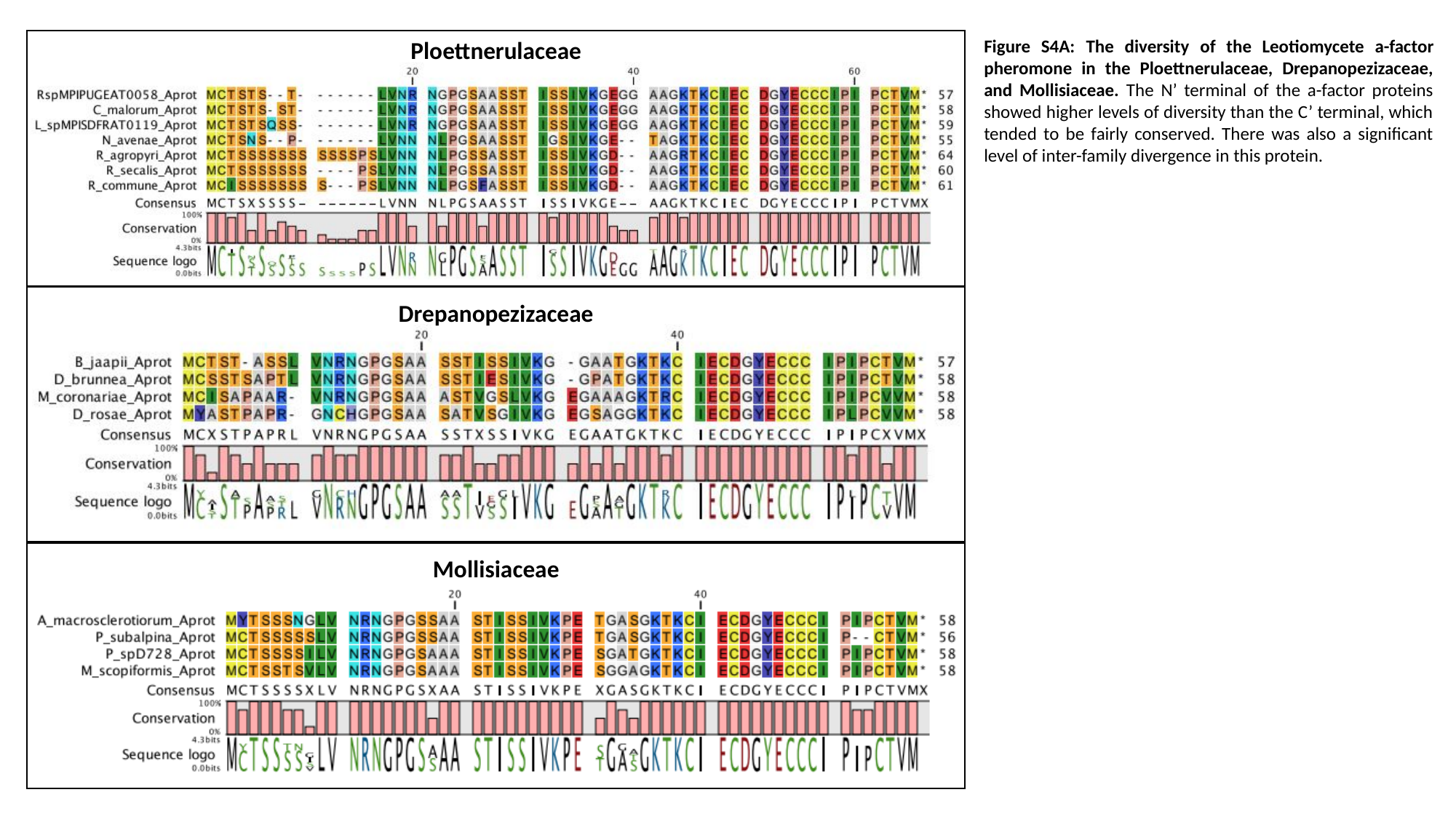

Figure S4A: The diversity of the Leotiomycete a-factor pheromone in the Ploettnerulaceae, Drepanopezizaceae, and Mollisiaceae. The N’ terminal of the a-factor proteins showed higher levels of diversity than the C’ terminal, which tended to be fairly conserved. There was also a significant level of inter-family divergence in this protein.
Ploettnerulaceae
Drepanopezizaceae
Mollisiaceae

## Slide 2
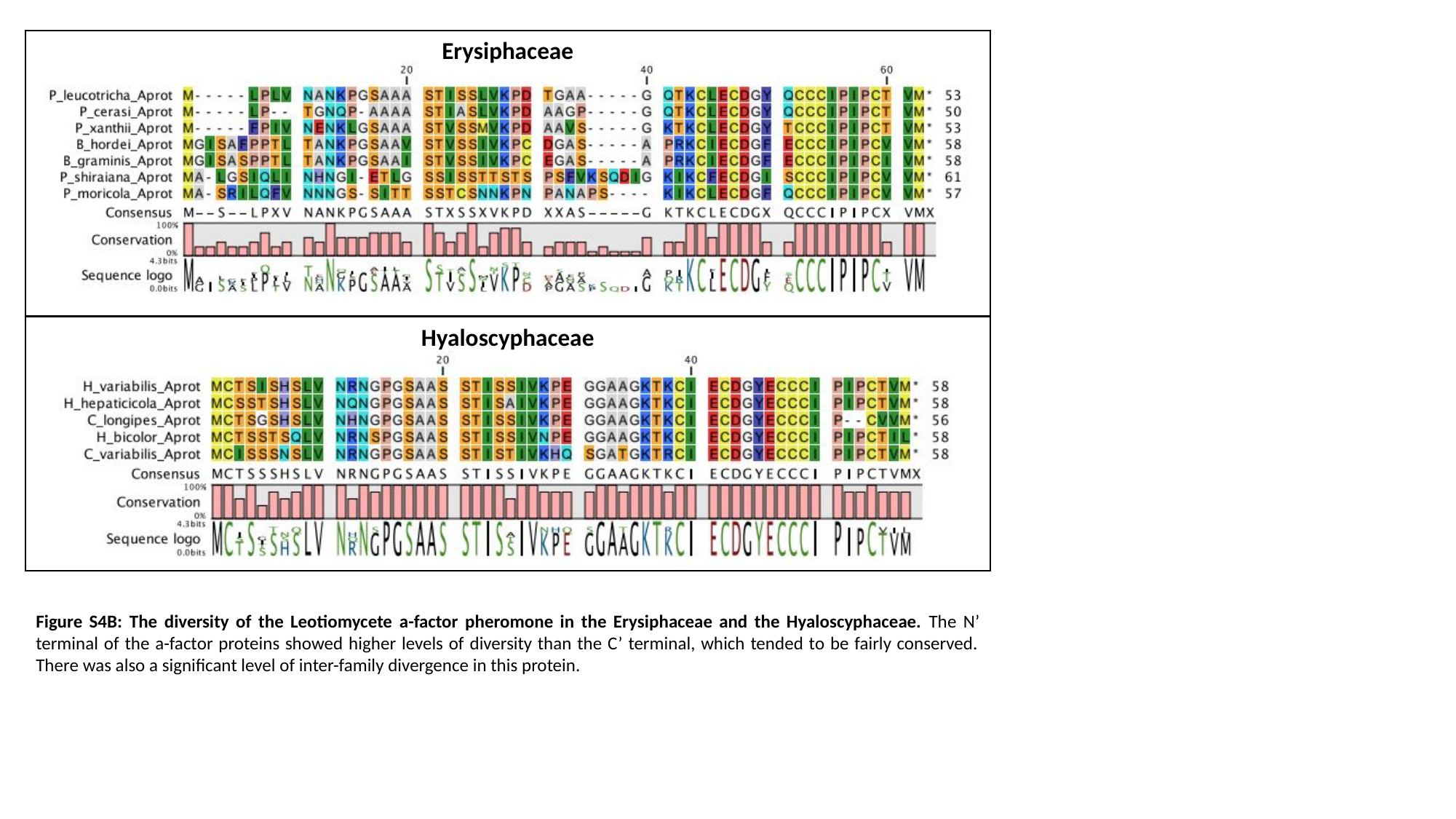

Erysiphaceae
Hyaloscyphaceae
Figure S4B: The diversity of the Leotiomycete a-factor pheromone in the Erysiphaceae and the Hyaloscyphaceae. The N’ terminal of the a-factor proteins showed higher levels of diversity than the C’ terminal, which tended to be fairly conserved. There was also a significant level of inter-family divergence in this protein.

## Slide 3
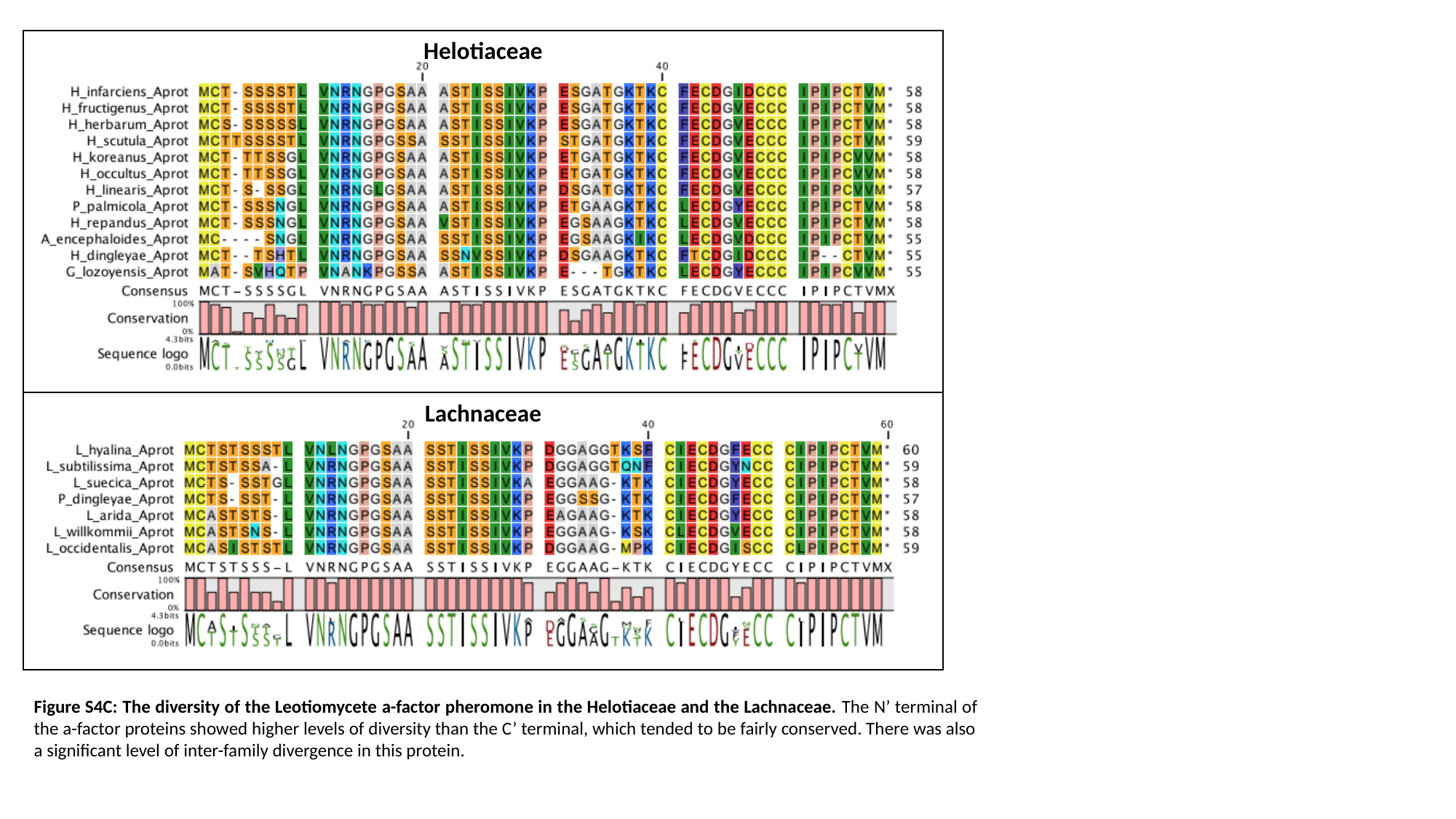

Helotiaceae
Lachnaceae
Figure S4C: The diversity of the Leotiomycete a-factor pheromone in the Helotiaceae and the Lachnaceae. The N’ terminal of the a-factor proteins showed higher levels of diversity than the C’ terminal, which tended to be fairly conserved. There was also a significant level of inter-family divergence in this protein.
