# Supplementary material for: Needles in fungal haystacks: Discovery of a putative a-factor pheromone and a unique mating strategy in the Leotiomycetes
Source: PLoS One. 2023 Oct 12;18(10):e0292619. doi: 10.1371/journal.pone.0292619 (PMC10569646; doi:10.1371/journal.pone.0292619)
Supplement: S5 Fig — (PPTX) [file pone.0292619.s005.pptx]

## Slide 1
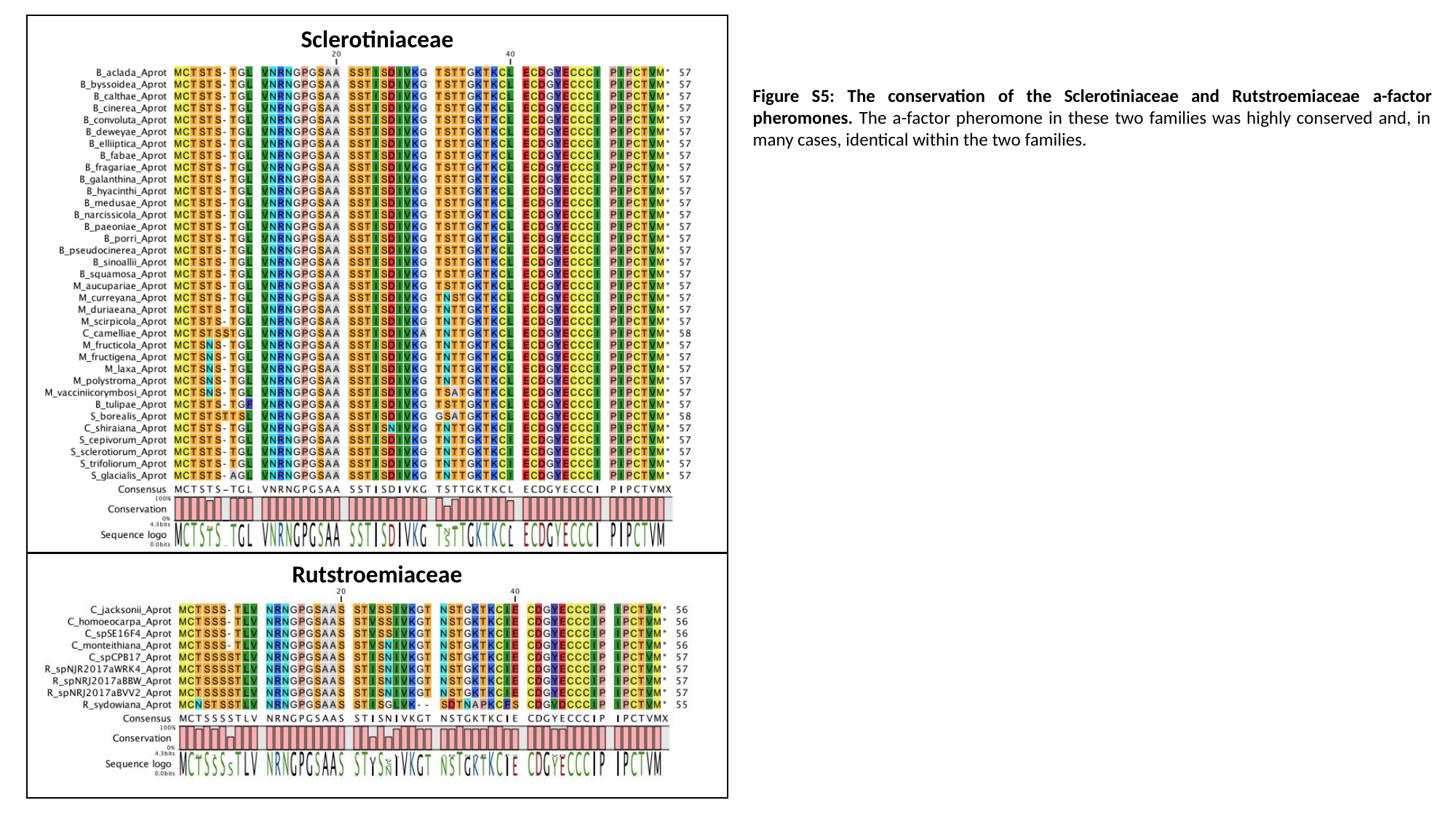

Sclerotiniaceae
Figure S5: The conservation of the Sclerotiniaceae and Rutstroemiaceae a-factor pheromones. The a-factor pheromone in these two families was highly conserved and, in many cases, identical within the two families.
Rutstroemiaceae
