# Supplementary material for: Needles in fungal haystacks: Discovery of a putative a-factor pheromone and a unique mating strategy in the Leotiomycetes
Source: PLoS One. 2023 Oct 12;18(10):e0292619. doi: 10.1371/journal.pone.0292619 (PMC10569646; doi:10.1371/journal.pone.0292619)
Supplement: S6 Fig — (PPTX) [file pone.0292619.s006.pptx]

## Slide 1
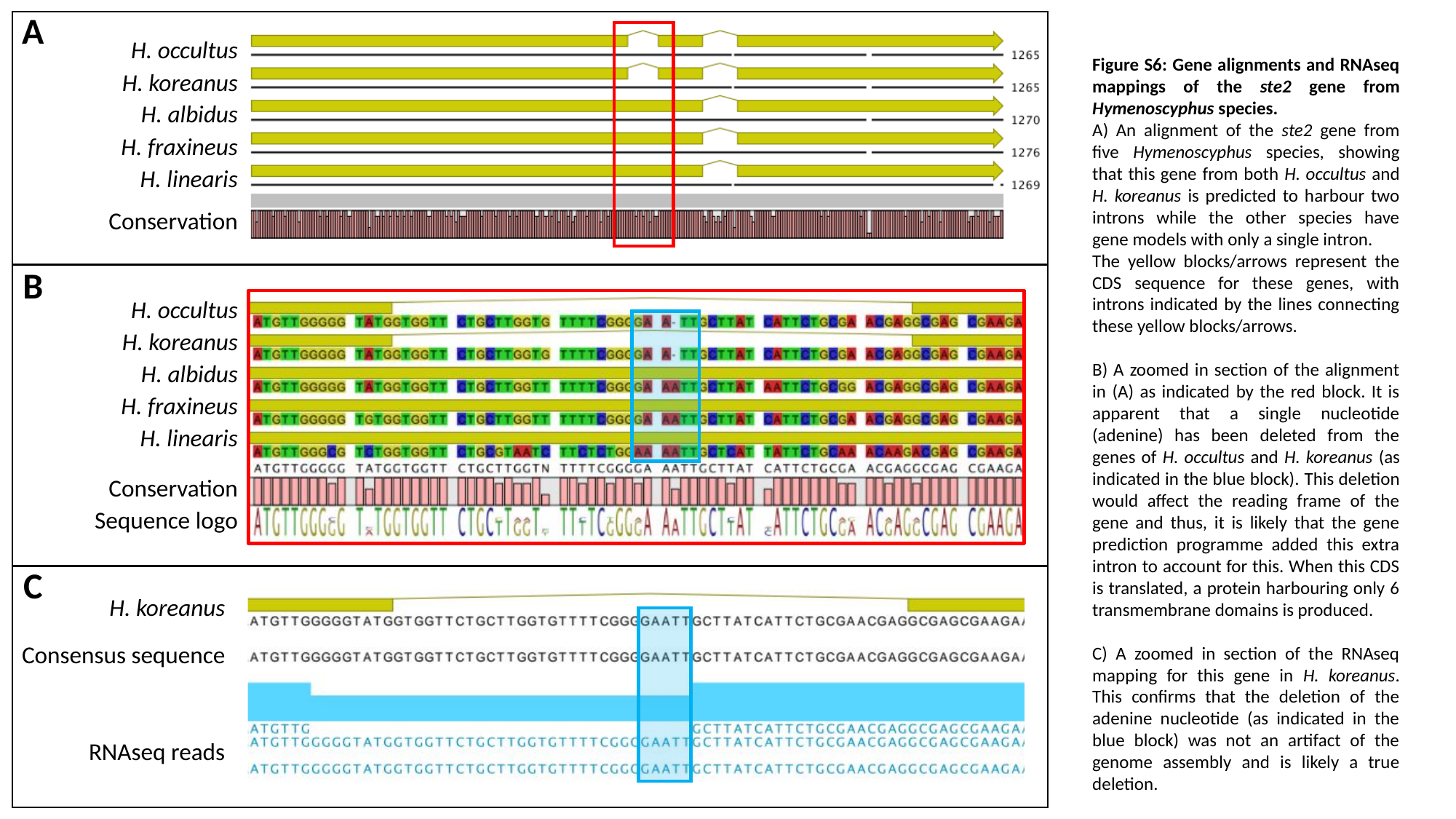

A
H. occultus
H. koreanus
H. albidus
H. fraxineus
H. linearis
Conservation
H. occultus
H. koreanus
H. albidus
H. fraxineus
H. linearis
Conservation
Sequence logo
H. koreanus
Consensus sequence
RNAseq reads
Figure S6: Gene alignments and RNAseq mappings of the ste2 gene from Hymenoscyphus species.
A) An alignment of the ste2 gene from five Hymenoscyphus species, showing that this gene from both H. occultus and H. koreanus is predicted to harbour two introns while the other species have gene models with only a single intron.
The yellow blocks/arrows represent the CDS sequence for these genes, with introns indicated by the lines connecting these yellow blocks/arrows.
B) A zoomed in section of the alignment in (A) as indicated by the red block. It is apparent that a single nucleotide (adenine) has been deleted from the genes of H. occultus and H. koreanus (as indicated in the blue block). This deletion would affect the reading frame of the gene and thus, it is likely that the gene prediction programme added this extra intron to account for this. When this CDS is translated, a protein harbouring only 6 transmembrane domains is produced.
C) A zoomed in section of the RNAseq mapping for this gene in H. koreanus. This confirms that the deletion of the adenine nucleotide (as indicated in the blue block) was not an artifact of the genome assembly and is likely a true deletion.
B
C
