# Supplementary material for: Needles in fungal haystacks: Discovery of a putative a-factor pheromone and a unique mating strategy in the Leotiomycetes
Source: PLoS One. 2023 Oct 12;18(10):e0292619. doi: 10.1371/journal.pone.0292619 (PMC10569646; doi:10.1371/journal.pone.0292619)
Supplement: S1 File — (PDF) [file pone.0292619.s011.pdf]

Supplementary File S1: Genomes that were excluded from the analyses

A. Genomes that were excluded due to the fragmented nature of the genome.

It was thus difficult to acquire intact genes and loci from these genomes and there was thus limited confidence regarding the presence and absence of certain genes.

1. *Ploettnerulaceae sp. D365*
2. *Lanzia echinophila*
3. *Cadophora sp. M221*
4. *Cadophora sp. DSE1049*
5. *Arachnopeziza araneosa*
6. *Hyphodiscus sp. D1413*
7. *Hymenoscyphus salicellus*
8. *Lachnum nothofagi*
9. *Aotearoamyces nothofagi*

B. Genomes that were excluded because they are not the species that they were labelled.

Preliminary phylogenomic analyses using the methods described in Section 4.7 suggested that these genomes did not belong to the Leotiomycetes. Follow up BLAST analyses using the ITS, TEF and BT regions of these genomes as queries against the NBCI NR database confirmed that they were not the expected species.

1. *Erysiphe necator*
2. *Hymenoscyphus laetus*
3. *Tetracladium sp. T11_2_10C*
