# Supplementary material for: Needles in fungal haystacks: Discovery of a putative a-factor pheromone and a unique mating strategy in the Leotiomycetes
Source: PLoS One. 2023 Oct 12;18(10):e0292619. doi: 10.1371/journal.pone.0292619 (PMC10569646; doi:10.1371/journal.pone.0292619)
Supplement: S2 File — (PDF) [file pone.0292619.s012.pdf]

#Additional File S2: A pheromone sequences from a variety of Sordariomycetes & Yeasts

>Chaetomium_globosum_NT165976_AFactor
MPSTTTQTKVPQTSTNFNGYCVVM*

>Podospora_anserina_AAV90629_AFactor
MPSTTAQTKVPQTSTNFNSYCVVM*

>Neurospora_crassa_XP011394832_AFactor
MPSTAASTKVPQTTMNFNGYCVVM*

>Sordaria_macrospora_AJ249863_AFactor
MPSTAASTKVPQTTMNFNGYCVVM*

>Gibberella_zeae_AACM02000166_AFactor
MPSTKPTSSQKPGYPLSCTVM*

>Candida_glabrata_CR380949_AFactor
MQPTIEATQKDNTQEKRDNYIVKGFFWSPDCVIA*

>Kluyveromyces_delphensis_AY181250_AFactor
MEPAQATQKDNSQDKKDNYVWRGRFWYPECVVV*

>Kluyveromyces_lactis_NC006041_AFactor
MQPTQQASQNESAENKENWIIPGFVWVPQCVVA*

>Kluyveromyces_waltii_AADM01000052_AFactor
MQPIAQATQNDSSDNKDNWIHKGLAWDPQCVIA*

>Saccharomyces_bayanus(1)_AACG02000017_AFactor
MQPITTVSAAPKDKTSTEKKDNYIIKGVFWDPACIIV*

>Saccharomyces_bayanus(2)_CAI4050096.1_AFactor
MQPVATVSAQASQKDKSSEKKDNYIIKGLFWDPACVIA*

>Saccharomyces_castelii(2)_AACF01000002_AFactor
MQPSAQASQKDNTAENKDNYIVKGLFWDPACVIA*

>Saccharomyces_castellii(1)_XP003677458_AFactor
MQPTTQATHKDNSAEKQDNYIVKGLFWDPACVIA*

>Saccharomyces_kluyveri(1)_AACE03000009_AFactor
MQPKSNATQKDSAENKDNWIIEGLAWNPQCVII*

>Saccharomyces_kluyveri(2)_AACE03000009_AFactor
MKAATHATQKGSTEDKENWIIKGFAWDPQCIIA*

>Colletotrichum_asianum_AFactor
MIPCFELKIDLYTATDPSPSTQSNFGHSSHTSSATISACVVM*

>Colletotrichum_camelliae_AFactor
MIPCSELKVDINTAKDPSPSTQSNFGHSSHTSSATISACVVM*

>Colletotrichum_caudatum_AFactor
MNSAPVCCEIKTMLKSSTGEAAALAQTNTNQGDYGGATILGCVIL*

>Colletotrichum_cereale_AFactor
MDSVPAYCEIKMKPKPGTGEAAIPTQTNTSPGDFGGATMLGCVIL*

>Colletotrichum_eremochloae_AFactor
MDSVPAYCEIKMKPKSGAWEAATPAQTNTNPGDYGGTTLLGCVIL*

>Colletotrichum_falcatum_AFactor
MDSVPAYYEIKMKPKSGTGEAAGTAQTNTNQGDYGGATILGCVIL*

>Colletotrichum_fructicola_AFactor
MIPCSELKIDLYTATDPSSSTQSNFGHSSHTSSATISACVVM*

>Colletotrichum_gloeosporioides_AFactor
MIPCSELKIDLYTATDPSSSTQSNFGHSSHTSSATISACVVM*

>Colletotrichum_graminicola_AFactor
MDSVPSFCEIKMKPKSGTGGGATTAQKNTNQGDYGGATILGCVIL*

>Colletotrichum_higginsianum_AFactor
MDSAPKCCKITTKSKSGAEEPTVPLQTNANPGYFGGASSHGCVIL*

>Colletotrichum_incanum_AFactor
MDSVAECCKITMKPKPGTEKPTVPTQMNTNPGDFGGATWVGCVIL*

>Colletotrichum_karsti_translation_AFactor
MKRFPDIINVDTITDPGTTTQSNVGNAYGNSTATIGGCLVM*

>Colletotrichum_navitas_AFactor
MDSVPAFCEIKMKPKSGTGEAATPAQKNTNQGDYGGATILGCVIL*

>Colletotrichum_orbiculare_AFactor
MTININYLSDPSMAPQAGIGGMTGGTTYHACVVM*

>Colletotrichum_orchidophilum_AFactor
MEAVPECCKIKLKPKSSSDQSSLAKTDAIEPGQFGTHGVTVNACVIL*

>Colletotrichum_sansevieriae_AFactor
MSPFSEAKINVPTIADPNTSAQSSVVHNYNTPTSTVGACVVM*

>Colletotrichum_shisoi_AFactor
MDSAPKCCKMTTKSKSGAEEPTVPTQTNANPGYFGGASSHGCVIL*

>Colletotrichum_sidae_AFactor
MTININYLSDPSMAPQAGIGGMTGGTTYHACVVM*

>Colletotrichum_somersetensis_AFactor
MDSVPAFCEIKTMLKSGIGEAAAPAQMNTNQGDYGGATMLGCVIL*

>Colletotrichum_spinosum_AFactor
MTININYLSDPSMAPQAGIGGMTGGTTYHACVVM*

>Colletotrichum_sublineola_AFactor
MDSVPAYCEIKMKPKSGAWEAATPAQTNTNPGDYGGTTLLGCVIL*

>Colletotrichum_tanaceti_AFactor

MESAPNFCKITTKSKSGVEEPTVPTQTNASPGYFGGASSHGCVIL*

>Colletotrichum_tofieldiae1_AFactor
MDSVAECCKITMKPKPGAEKPAVPTKTNTNPGDFGGTTWVGCVIL*

>Colletotrichum_tofieldiae2_AFactor
MDSVAECCKITMKPKPGAEKPAVPTKTNTNPGDFGGTTWVGCVIL*

>Colletotrichum_trifolii_AFactor
MTININYLSEPSMAPQAGIGGMTGGTTYHACVVM*

>Colletotrichum_truncatum_3_AFactor
MNSNSELQYSINLITKPNAAIQTNIGDGVTAGFATDTMCVVM*

>Colletotrichum_truncatum_AFactor
MNSNSELQYSINLITKPNAAIQTNIGDGVTAGFATDTMCVVM*

>Colletotrichum_zoysiae_AFactor
MDSVPACCEIKTMLRSGIGEAAAPGQTNTNQGDYGGATMLGCVIL*

>Verticillium_dahliae_AFactor
MPSYTQKNGGGHSGCSIMKNGGGHSGCSIMKNGGGHSGCSIMKNGGGHSGCSVMKNGGGHSGCAIM*

>Verticillium_tricorpus_AFactor
MPSRAQKNGGGHSGCSVMKNGGGHSGCSVMKNGGGHSGCSVMKNGGGHSGCSVMKNGGGHSGCSVM*

>Neurospora_africana_AFactor
MPSTAASTKVPQTTMNFNGYCVVM*

>Neurospora_tetrasperma_AFactor
MPSTAASTKVPQTTMNFNGYCVVM*

>Neurospora_pannonica_AFactor
MPSTAASTKVPQTTMNFNGYCVVM*

>Neurospora_crassa_AFactor
MPSTAASTKVPQTTMNFNGYCVVM*

>Neurospora_terricola_AFactor
MPSTAASTKVPQTTMNFNGYCVVM*

>Neurospora_sublineolata_AFactor
MPSTAASTKVPQTTMNFNGYCVVM*

>Neurospora_discreta_AFactor
MPSTAASTKVPQTTMNFNGYCVVM*

>Huntiella_abstrusa_AFactor(1)
MAAIKNTTSSKNAARGVDQSSGCSVMRGVDQSSGCSVMRGVDQSSGCTLMRGVDQSSGCTLM*

>Huntiella_bhutanensis_AFactor(1)
MAAIKNITSSKNAARGVDQSNPCNVMRGVDQSNPCAVMRGVDQSNPCTVM*

>Huntiella_decipiens_AFactor(1)
MAAIKNITSSKHAARGVDQSNGCSVMRGVDQSNGCTVMRGVDQSNGCTVMRGVNQSNGCTVMRGVNQS
NGCTLM*

>Huntiella_fecunda_AFactor(4)
MPSIKNHTPSTKTSGNETIQPPTSNAGRGAIQSPINPITRGVTQAPPCNVMRGVTQAPPCNVM*

>Huntiella_moniliformis_AFactor(4)
MPSIKNHTPSTKTSGNETIQPPTSNAGRGAIQSPINPITRGVTQAPPCNVMRGVTQAPPCNVM*

>Huntiella_omanensis_AFactor(1)
MAAIKNTTTSKNAARGVDQSNPCAVMRGVDQSNPCAVMRGVDQSNPCTVMRGVDQSNPCTLM*

>Huntiella_savannae_AFactor(1)
MASVKNITSSKHAARGVDQSTPCSVMRGVDQSTPCNVMRGVDQSTPCNVMRGVDQSTPCTVM*

>Huntiella_tyalla_AFactor(4)
MPSIKNHTPSTKTSGNETIQPPTSNAGRGAIQSSINPITRGVTQAPPCNVMRGVTQAPPCNVM*
