# Supplementary material for: Needles in fungal haystacks: Discovery of a putative a-factor pheromone and a unique mating strategy in the Leotiomycetes
Source: PLoS One. 2023 Oct 12;18(10):e0292619. doi: 10.1371/journal.pone.0292619 (PMC10569646; doi:10.1371/journal.pone.0292619)
Supplement: S3 File — (PDF) [file pone.0292619.s013.pdf]

#Additional File S3: CAAX/CpAX proteins (<100aa) from B. cinerea, B. sinoalli, S. sclerotiorum, M. fructicola, R. commune, A. resinae & P. destructans

>M_fructicola_VICG01000013.1_EYC84_009782
MLLFLRFLSWGFFSTVGWLGNGHFHASIISCVPNLCVIHGHDSNQYHLFMGNKFLSLPLR
PTTMCSAP*

>M_fructicola_VICG01000010.1_EYC84_008624
MRVMVQEYLAGVQEKMLARRAEDSQGLKWPMNQAGDARCLCWFQLPTFINLAYGLLSCSG
VVMLCCIVLCCVVL*

>M_fructicola_VICG01000011.1_EYC84_009873
MQDEDEDDYALAAMGISNGGGYRPSDSNQNTYTPPPPQPESQLPPLERRRASTPPPETLK
HNKAKRNRFVCVAK*

>M_fructicola_VICG01000002.1_EYC84_004913
MKIRKTLVESGQMESDADDDEEEEEEDGGGGGGDGVDVVKPCRSKRALKRLKLRSSHGKL
RLKSKRVEYGALYKCTLYCTVE*

>M_fructicola_VICG01000003.1_EYC84_005768
MPYRAIPQLKINNFPLRSLLRPPVPKIALWPSSLNSLFCPPSPLLVYHLPTSPTAYRYIT
IRSARGCLLSHYPPPKRTCPIP*

>M_fructicola_VICG01000011.1_EYC84_010293
MVFFKYLLRFVSRSWTFKTSDHVASDFCEFGVSLDDRYIVQCNLTCTTHATGRPVGIGNE
KSYPIQLSVFTKLCIDIICACNAN*

>M_fructicola_VICG01000016.1_EYC84_011744
MAQQPQKPHRSLWDREPSDVANMPPVYTQEYDNRVSPQPQSQPQPANAVKDASETQKEAI
YVQKQQNERKNNEDDVASGCCGCSVM*

>M_fructicola_VICG01000007.1_EYC84_002179
MQFIATTFIAILSAVAAASPLQSRSGLCSSALDTAQCCDVSVAGVANLNCASPSSTPTSV
ENFRSICAAGGQQASCCVLPVAGDALVCVAP*

>M_fructicola_VICG01000007.1_EYC84_002586
MPDNGITLCQYPSIRHLNHWYSTSGIYLCNFSILFLFWPFFKRIPDIPERSPRINEQQSN
YLSSASGLEIEIMNRRDAAKGVVGCTFCAVC*

>M_fructicola_VICG01000004.1_EYC84_003548
MKRKEEKTKDKKKIIIVQQGLPSILHSSPQAGTSNPHLTQIHSFHDRFGIPKREQDPCSV
PLHVTQDQPPSASQTARESMSSLHNHDAPCLPV*

>M_fructicola_VICG01000011.1_EYC84_010490
MIYKLFEPSRSFDFWDAGHLYQSRIKELIQDKDGDLETYGMVAQRELSTTRVLVHTKTLF
YFLPLISIHGWIQSMQGNVRQGKARQGKAMQCNVA*

>M_fructicola_VICG01000006.1_EYC84_000648
MIRFRMGRSRIRSSALSIFHEEINTVMRLFVYIISSSPPLLLSPPQLTIYTSILIIILAQR
HPHSHHITGHDIILIYTQGNAMQRSAAQIEKLLACLLA*

>C_cinerea_NC_037317.1_BCIN_08g01220
MPADWRTDKSGNVKGFIRRKGDPNPLNWGYKVDDKVLSHPKAAMIKTDVSKPKKDDKCKS
CEKKKKKEEKKKEKKKSAGGCIVS*

>C_cinerea_NC_037322.1_BCIN_13g03330
MCTSTSTGLVNRNGPGSAASSTISDIVKGTSTTGKTKCLECDGYECCCIPIPCTVM*

>C_cinerea_NC_037319.1_Bcgg1
MPQGYSSRDVGDPSQIKKNKQSMADLKLRRLTELNNRLREDLERERIPVSQAAKSIIQYT
SQTKDFMVPSIWGTVEKKDDPYAPQQSGGCCLVM*

>C_cinerea_NC_037315.1_Bhp3
MQFTTTTLIAILSALAVASPIEPRQNATAQQERLCTSAIDTAMCCQTTLAGVINQTCTTP
AITPINKQAFRAYCAAQGQDSSCCKTPLVGDGVICTPP*

>B_sinoallii_NW_024066037.1_EAF02_006546
MPADWRTDKSGNVKGFIRRKGDPNPMNWGYKVDDKVLSHPKAGMVKTDVSKPKKDDKCKS
CEKKKEKEKKKKEKKDSAGGCLVS*

>B_sinoallii_NW_024066059.1_EAF02_011438
MPQGYSSRDVGDPSQIKKNKQSMADLKLRRLTELNNRLREDLERERIPVSQAAKSIIQYT
SQTKDFMVPSIWGTVDKKDDPYAPQQSGGCCLVM*

>B_sinoallii_NW_024066033.1_EAF02_004193
MTTLSLEAWIWITQCAGKTCKFFAFPTNLPRLPTGNSLLAEAHEAPYRSRKSQQQSYDQH
VEKFRHAWRKKENPAAGLADGCSGICSGFRKGCPAL*

>B_sinoallii_NW_024066029.1_EAF02_001539
MTPQPSHFKTFTLSSLYRHHLKPHKLILILNLNLNQIQQLKMCTSTSTGLVNRNGPGSAA
SSTISDIVKGTSTTGKTKCLECDGYECCCIPIPCTVM*

>B_sinoallii_NW_024066033.1_EAF02_004477
MQFTITTLIAILSAIAAASPIEPRQNGTAQQERLCTSAIDTAMCCQTSLAGVINQTCSTP
AITPFTKEQFRAYCAAQGQDSSCCKTPLVGDGVICTPP*

>S_sclerotiorum_NW_001820814.1_SS1G_12764
MAYDPAMPWILWKSWQTLTDGAISSKSDCLCAAP*

>S_sclerotiorum_NW_001820822.1_SS1G_10003
MTDTNSSQTKHCEKVIVGGPPVEEINAKTFCIAA*

>S_sclerotiorum_NW_001820812.1_SS1G_13368
MGIHARCQRIKRADCALRNKSCQRSMRDAAQCVIV*

>S_sclerotiorum_NW_001820812.1_SS1G_13356
MVEKRDRLLDYAKAHNIAVPTMGAAFEGCSNCSIE*

>S_sclerotiorum_NW_001820832.1_SS1G_03496
MGHRAVVLPLHFKVSWKPAPVNPIRIMIFFSASGDVYCLLD*

>S_sclerotiorum_NW_001820831.1_SS1G_04828
MVDVEAFGWSGYCTASSKACCPAQLRHLCPTPPVGAVTVPDLAQVSSVTILSSYLCVVV*

>S_sclerotiorum_NW_001820832.1_SS1G_03509
MSSPDTPGNIEGLLRRKGDLNPMNWGFKVDDKILDHPKIGLIKTDVSKPKDDGKCKSCVK
KQEKAKKKEKNGDAGGCVVS*

>S_sclerotiorum_NW_001820822.1_SS1G_09848
MCGRCLTKQGVHIDKDFLQEANARRRSEAAEIRQSTILTLKILEQANVFVKEEKRNNRER
RRRRNTRGGMADRGKIGKQRKCVVM*

>S_sclerotiorum_NW_001820828.1_SS1G_06541
MTIASVRESLFLQTVRRRRIISALYSISVYGTKVTSRMEGLYVYCGGMGTEGIANGGQSA
ITDTQRLATKDHRPHIIVWSNKCSIY*

>S_sclerotiorum_NW_001820815.1_SS1G_12567
MPQGYSSRDVGDPSQIKKNKQSMADLKLRRLTELNNRLREDLERERIPVSQAAKSIIQYT
SQTKDFMVPSIWGTVDKKDDPYAPQQSGGCCLVM*

>S_sclerotiorum_NW_001820824.1_SS1G_09095
MAQNPQEPHRSRWDREPADVANMPPAYTQENETKPPFQPQPLPRPTYAMNDGRGGQREVV
YVQQQQVERKHNKDDIALGCCAGCAAGCCCCGCSVM*

>R_commune_FJUW01000046.1_CZT08289.1
MSAQQLIYLHPWTGRSQQLEVSPGHGPWIYDIAYCVAN*

>R_commune_FJUW01000014.1_CZS98287.1
MARENRFPKVAGYTVNTGPSNNPARYNADYDYQRNRPHRPHGGHHSSHRPHGHHSSSRKK
DPCRCVVM*

>R_commune_FJUW01000010.1_CZS95335.1
MEASCLPRHDIHPSLGTLTEEFGSIILDDRLALATASMYRGLTGEGSPGWSVGRSVDIRP
EKLRRCPLF*

>R_commune_FJUW01000051.1_CZT09580.1
MNLEINLRNSNSLTGTLKSCSIYATPRPILPSHERTPPVKLLDVAAISDSDTEIENQTDT
TSTNVNNPGCTLP*

>R_commune_FJUW01000027.1_CZT03207.1
MCGCYVSSRESKVAKSLFENVGISSIYEHLAPSNKSYIVSDWAATALHIFGLSSVQTQAE
SFRNKKAQGVPQRHRSSGCVPH*

>A_resinae_NW_020194476.1_M430DRAFT_139873
MCTATPSAPINHNGPGSAASSTISSIVKPEAGAAGKTKCLECDGFECCCIPIPCTVM*

>A_resinae_NW_020194477.1_M430DRAFT_141309
MPSGYSSRDVGDPSQVKKNKQSMADLKLRRLTELNNRLREDLERERIPVSQAAKSIITYT
NNTKDFMVPSVWGTVDKKEDPYAPQQSGGCCVVM*

>P_destructans_NW_020167544.1_GNG1
MGYASRDAADPSQIKKNKQSMADLKLRRLTELNNRLREDLERERIPVSQAARSIIAYTDT
TRDFMVPSVWGPVDKKDDPYTPQPTGGCCVVM*

>P_destructans_NW_020167532.1_VC83_05352
MARSTDPAPLRIAGIQGSGTDREAAGHSRISCKPSSSSLADFKEPAYLAINPNGRVPSIH
DPNTDLTLWESGAIIEYLIEKYDSGRDQIICPLC*
