# Supplementary material for: Needles in fungal haystacks: Discovery of a putative a-factor pheromone and a unique mating strategy in the Leotiomycetes
Source: PLoS One. 2023 Oct 12;18(10):e0292619. doi: 10.1371/journal.pone.0292619 (PMC10569646; doi:10.1371/journal.pone.0292619)
Supplement: S4 File — (PDF) [file pone.0292619.s014.pdf]

#Additional File S4: Character state statistics from the ancestral state reconstruction analyses

node125	0.09870356	0.90129644
node126	0.06089178	0.93910822
node127	0.01605149	0.98394851
node128	0.00447921	0.99552079
node129	0.00124412	0.99875588
node130	0.00047188	0.99952812
node131	0.00054733	0.99945267
node132	0.00101774	0.99898226
node133	0.00167607	0.99832393
node134	0.00161923	0.99838077
node135	0.00211741	0.99788259
node136	0.00173674	0.99826326
node137	0.00251705	0.99748295
node138	0.00124970	0.99875030
node139	0.00100684	0.99899316
node140	0.00000120	0.99999880
node141	0.00000012	0.99999988
node142	0.00207155	0.99792845
node143	0.00147530	0.99852470
node144	0.00079939	0.99920061
node145	0.00011683	0.99988317
node146	0.00285203	0.99714797
node147	0.00557889	0.99442111
node148	0.02976367	0.97023633
node149	0.00878271	0.99121729
node150	0.00060613	0.99939387
node151	0.03419888	0.96580112
node152	0.56192200	0.43807800
node153	0.65516820	0.34483180
node154	0.80625061	0.19374939
node155	0.00426565	0.99573435
node156	0.03059804	0.96940196
node157	0.03579409	0.96420591
node158	0.07675811	0.92324189
node159	0.05499074	0.94500926
node160	0.00279084	0.99720916
node161	0.00379417	0.99620583
node162	0.00038407	0.99961593
node163	0.03704705	0.96295295
node164	0.21859621	0.78140379
node165	0.00100257	0.99899743
node166	0.01282803	0.98717197
node167	0.00550039	0.99449961
node168	0.01524597	0.98475403
node169	0.00202326	0.99797674
node170	0.00607658	0.99392342
node171	0.00749340	0.99250660
node172	0.02044830	0.97955170
node173	0.02848188	0.97151812
node174	0.03987084	0.96012916
node175	0.13235476	0.86764524
node176	0.19220919	0.80779081
node177	0.96909897	0.03090103

node178	1.00000000	0.00000000
node179	0.02892198	0.97107802
node180	0.04556902	0.95443098
node181	0.03586778	0.96413222
node182	0.03688434	0.96311566
node183	0.97335281	0.02664719
node184	0.01928050	0.98071950
node185	0.07705365	0.92294635
node186	0.00199155	0.99800845
node187	0.00177555	0.99822445
node188	0.00004385	0.99995615
node189	0.00001931	0.99998069
node190	0.00000625	0.99999375
node191	0.00005045	0.99994955
node192	0.00008692	0.99991308
node193	0.00125138	0.99874862
node194	0.00399806	0.99600194
node195	0.00882037	0.99117963
node196	0.00384444	0.99615556
node197	0.00445009	0.99554991
node198	0.01352949	0.98647051
node199	0.00456928	0.99543072
node200	0.00302323	0.99697677
node201	0.01814661	0.98185339
node202	0.00004640	0.99995360
node203	0.00000102	0.99999898
node204	0.00000002	0.99999998
node205	0.00000022	0.99999978
node206	0.00000019	0.99999981
node207	0.00000015	0.99999985
node208	0.00000003	0.99999997
node209	0.00000040	0.99999960
node210	0.00000075	0.99999925
node211	0.00000069	0.99999931
node212	0.00000002	0.99999998
node213	0.00000002	0.99999998
node214	0.00000001	0.99999999
node215	0.00000104	0.99999896
node216	0.00000059	0.99999941
node217	0.00000177	0.99999823
node218	0.00000100	0.99999900
node219	0.00000027	0.99999973
node220	0.00000010	0.99999990
node221	0.61851718	0.38148282
node222	0.75943209	0.24056791
node223	0.99111504	0.00888496
node224	0.99999812	0.00000188
node225	0.00150141	0.99849859
node226	0.00058312	0.99941688
node227	0.00100906	0.99899094
node228	0.00007849	0.99992151
node229	0.00001693	0.99998307
node230	0.00001876	0.99998124
node231	0.02043251	0.97956749
node232	0.00006870	0.99993130
node233	0.00001330	0.99998670

node234	0.00785695	0.99214305
node235	0.00389952	0.99610048
node236	0.00029186	0.99970814
node237	0.00000405	0.99999595
node238	0.00000009	0.99999991
node239	0.00000000	1.00000000
node240	0.00005325	0.99994675
node241	0.00000994	0.99999006
node242	0.00000183	0.99999817
node243	0.01049598	0.98950402
node244	0.00038629	0.99961371
node245	0.00168182	0.99831818
node246	0.00100108	0.99899892
node247	0.00009975	0.99990025
