# Supplementary material for: Needles in fungal haystacks: Discovery of a putative a-factor pheromone and a unique mating strategy in the Leotiomycetes
Source: PLoS One. 2023 Oct 12;18(10):e0292619. doi: 10.1371/journal.pone.0292619 (PMC10569646; doi:10.1371/journal.pone.0292619)
Supplement: S5 File — (PDF) [file pone.0292619.s015.pdf]

#Additional File S5: MAT1-1-13 sequences from Rhynchosporium species

>R_agropyri_MAT1113gene
ATGGCGAGGCTGCTCAAAGAAGACAATCTCTATGTGCCTAGCCAAGTTATTTCTTTTGTT
CACATCCACACGGTACGAAAAGCTTCCGGATCTAGCTGCTTACTATCTCTAACATTCACA
TCTATACAGTACGAAAATTCTCCTGAGTTTTCCTTCACGGACGTCCCTGGTGGCTCGAAG
CATGTTTGCACAGCTCTCGAGGCACGCAGTATCTCTCGCAGTCAAAGAGAACGAAAGAGA
AACATAGCACATATGGCAGAAGGGTCGGAAAGGATTATCCAATATCGAGCCTGGAAGCTT
TCAAGGATTGAGCAGGGGGGAGAGGGATGA

CDS: join(1..72,130..330)

>R_agropyri_MAT1113prot
MARLLKEDNLYVPSQVISFVHIHTYENSPEFSFTDVPGGSKHVCTALEARSISRSQRERK
RNIAHMAEGSERIIQYRAWKLSRIEQGGEG*

>R_commune_MAT1113gene
ATGGCGAGGCTGCTCAAAGAAGACAATCTCTATGTGCCTAGCCAAGTTATTTCTTTTGTT
CACATCCACACGGTACGAAGAGCTTTCGGATCTAGCTGCTTATTATCTCTAACATTCACA
TCTATACAGTACGAAAATTCTCCTGAGCTTGACTTCATGGACGTCCCTGGTGGCTCGAAG
CATGTTTGCACAGCTCTCGAGGCACGCAGTATCTCTCGCAGTGAAAGAGAACGAAAGAGA
AACATAGCACATATGGCAGAAGGGTCGGAAAGGATTATCCAATATCGAGCCTGGAAGCTT
TCAAGGACTGAGCAGGGGGGAGAGGGATGA

CDS: join(1..72,130..330)

>R_commune_MAT1113prot
MARLLKEDNLYVPSQVISFVHIHTYENSPELDFMDVPGGSKHVCTALEARSISRSERERK
RNIAHMAEGSERIIQYRAWKLSRTEQGGEG*

>R_secalis_MAT1113gene
ATGGCTGGGCTGCTCAAAGAAGACAATCTCTATGTGCCTAGCCAAGTTATTTCTTTTGTT
CACATCCACACGGTACGAAAAGCTACCGGATCTAGCTGCTTACTATCTCTAACATTCACA
TCTATACAGTACGAAAATTCTCCTGAGTTTTCCTTCACGGACGTCCCTGGTGGCTCGAAG
CATGTTTGCACAGCTCTCGAGGCACGCAGTATCTCTCGCAGTCAAAGAGAACGAAAGAGA
AACATAGCACATATGGCAGAAGAATCGGAAAGGATTATCCAATATCGAGCCCGGAAGCTT
TCAAGGATTGAGCAGGGGGGAGAGGGATGA

CDS: join(1..72,130..330)

>R_secalis_MAT1113prot
MAGLLKEDNLYVPSQVISFVHIHTYENSPEFSFTDVPGGSKHVCTALEARSISRSQRERK
RNIAHMAEESERIIQYRARKLSRIEQGGEG*
