# Supplementary material for: Needles in fungal haystacks: Discovery of a putative a-factor pheromone and a unique mating strategy in the Leotiomycetes
Source: PLoS One. 2023 Oct 12;18(10):e0292619. doi: 10.1371/journal.pone.0292619 (PMC10569646; doi:10.1371/journal.pone.0292619)
Supplement: S6 File — (PDF) [file pone.0292619.s016.pdf]

Additional File S6: Species that were classified as "undetermined" with regards to their sexual strategy

1. ***Sclerotinia glacialis:*** Could be either heterothallic or mating type switching; intact *MAT1-1-1* and *MAT1-1-5* genes, a short fragment of the *MAT1-2-1* gene, and no *MAT1-2-10* gene
2. ***Sclerotium cepivorum:*** Could be either primary homothallism or mating type switching; intact *MAT1-2-4, MAT1-2-1*, and *MAT1-1-5* genes, *MAT1-1-1* gene is separated across two contigs
3. ***Chlorencoelia torta:*** Could be homothallism or heterothallism; *MAT1-1* and *MAT1-2* genes on two separate contigs
4. ***Cadophora malorum:*** Unclear if sexual reproduction is possible as *MAT1-1-1* is truncated and it's not clear if *MAT1-1-3* and *MAT1-2-1* are intact.
5. ***Erysiphe pulchra:*** Could be homothallism or heterothallism; *MAT1-1* and *MAT1-2* genes on two separate contigs
6. ***Golovinomyces magnicellulatus:*** Unclear if sexual reproduction is possible as *MAT1-1-1* is truncated, *MAT1-2-1* is intact.
7. ***Phyllactinia moricola:*** Could be homothallism or heterothallism; *MAT1-1* and *MAT1-2* genes on two separate contigs
8. ***Chalara longipes:*** Unclear if sexual reproduction is possible as *MAT1-1-1* is truncated, *MAT1-2-1* is intact.
9. ***Hyaloscypha bicolor:*** Unclear if sexual reproduction is possible as *MAT1-1-1* is truncated, *MAT1-2-1* is intact.
10. ***Hyaloscypha hepaticicola:*** Unclear if sexual reproduction is possible as *MAT1-1-1* and *MAT1-2-1* are truncated
11. ***Hymenoscyphus linearis:*** Unclear if sexual reproduction is possible as *MAT1-1-3* and *MAT1-2-1* are truncated, *MAT1-1-1* is intact.
12. ***Hymenoscyphus herbarum:*** Unclear if sexual reproduction is possible as *MAT1-1-3* and *MAT1-2-1* are present, but *MAT1-1-1* is absent
13. ***Hymenotorrendiella dingleyae:*** Could be homothallic or heterothallic; *MAT1-1* genes are flanked by APN and SLA but *MAT1-2-1* is elsewhere in the genome.
