# Supplementary material for: Needles in fungal haystacks: Discovery of a putative a-factor pheromone and a unique mating strategy in the Leotiomycetes
Source: PLoS One. 2023 Oct 12;18(10):e0292619. doi: 10.1371/journal.pone.0292619 (PMC10569646; doi:10.1371/journal.pone.0292619)
Supplement: S7 File — (PDF) [file pone.0292619.s017.pdf]

Additional File S7: Explanations regarding the sexual strategy determinations in species with both *MAT1-1-* and *MAT1-2*-associated genes

Genes associated with the *MAT1-1* and *MAT1-2* idiomorphs were identified from the genomes of numerous species in this study. While this would typically indicate that the species is capable of homothallic mating, there were four species that we could not confirm as homothallic given the fragmented nature of the *MAT* locus.

Homothallism can arise due to the presence of *MAT1-1* and *MAT1-2* genes at the same locus (eg: XX) or the presence of *MAT1-1* and *MAT1-2* at two unliked loci (eg: XX). In species where the genes are present at the same locus, the locus is typically flanked by *SLA2*, *APN2*, or other *MAT* locus-associated genes. In contrast, in species where the *MAT* genes are harboured at unlinked loci, it is often the case that one of the two idiomorphs are flanked by common *MAT* locus-associated genes while the other idiomorph is present elsewhere in the genome. When assembling a genome from a homothallic species, it is thus expected that a single *MAT* locus complete with the relevant flanking genes will be assembled within a contig or two *MAT* loci complete with the relevant flanking genes will be assembled on different contigs (see Figure below).

When the genome of a heterothallic species is sequenced and assembled from a mixed mating type isolate, both *MAT1-1* and *MAT1-2* genes will also be identified from the genome. However, because these idiomorphs will share near-identical flanking genes, the two idiomorphs are usually assembled into the genome differently. One of the idiomorphs, with its relevant flanking genes, will be assembled within a contig. The second idiomorph will subsequently be assembled in isolation, with no flanking genes because that sequence will have already been assembled into the genome (see Figure below).

Unfortunately, in numerous cases, the *MAT1-1* and *MAT1-2* genes were present on individual contigs with no flanking genes. In these cases, it is not possible to determine whether the isolates these genomes came from represent a homothallic or heterothallic species.

The genomes that harboured both *MAT1-1* and *MAT1-2* idiomorphs could thus be divided into those that were most likely homothallic and those that could have been homothallic or heterothallic (undetermined).

a) **Homothallic with a single *MAT* locus:** *Mollisia scopiformis, Pleochaeta shiraiana, Hyaloscypha variabilis, Hymenoscyphus koreanus*, Hymenoscyphus occultus*, Hymenoscyphus albidus*, Amylocarpus encephaloides, Hymenoscyphus repandus*

b) **Homothallic with two unlinked *MAT* loci:** *Hymenoscyphus fructigenus, Cudoniella acicularis*

c) **Undetermined (See Additional File S2):** *Chlorencoelia torta, Erysiphe pulchra, Phyllactinia moricola and Hymenotorrendiella dingleyae*

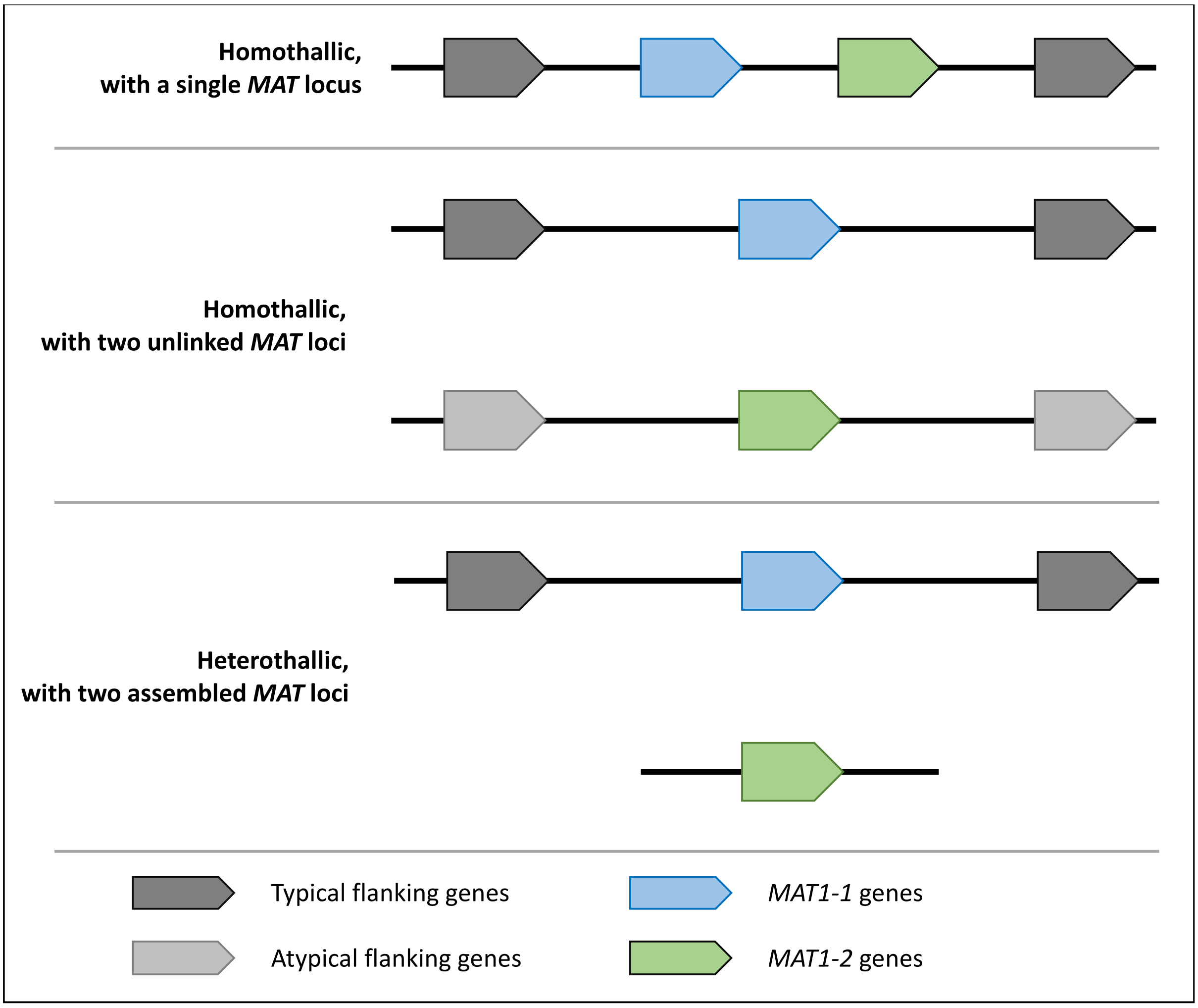

Figure: A schematic representation of the typical assembly of the *MAT* locus in the genomes of species with different sexual strategies.
