# Supplementary material for: Needles in fungal haystacks: Discovery of a putative a-factor pheromone and a unique mating strategy in the Leotiomycetes
Source: PLoS One. 2023 Oct 12;18(10):e0292619. doi: 10.1371/journal.pone.0292619 (PMC10569646; doi:10.1371/journal.pone.0292619)
Supplement: S8 File — (PDF) [file pone.0292619.s018.pdf]

```
#Additional File S8: Alpha pheromone sequences for all Leotiomycetes
(GBK)

LOCUS       A_resinae_ppg1       275 aa            linear   UNA
FEATURES             Location/Qualifiers
     KR              48..49
     KR              85..86
     AlphaRep        127..138
                     /label="AWAlpha"
     KR              139..140
     AlphaRep        147..158
                     /label="AWAlpha"
     KR              159..160
     AlphaRep        169..180
                     /label="AWAlpha"
     KR              181..182
                     /label="RR"
     AlphaRep4       195..206
                     /label="AWAlpha"
     KR              207..208
     AlphaRep3       219..230
                     /label="AWAlpha"
     KR              231..232
     AlphaRep        245..256
                     /label="AWAlpha"
     KR              257..258
ORIGIN
        1 MRLTNALCVI ILAATASAVA IPEPLALPLP EAYAEAKCGE FGECGRVKRA
AEAFAEAFAE
       61 PSAEPEDSIV MARCNLVGGA CYEAKRLARD LASVLAHTRD EGSAFVARLA
LEDEETSDLK
      121 AREAEAKCGK VGQPCGKIKR VAEPEAKCGK VGQPCGKAKR IADPEAEAKC
GKVGQPCGKL
      181 RRAAEAIAEA IAEPECGKVG QPCGKAKREA DALAEAEAVC GKVGQPCGKA
KRDALAAAIA
      241 EADAKCGKVG QPCGKAKRDA HAIASAVSTV LEQL*
//
LOCUS       A_pellizariae_ppg1       250 aa            linear   UNA
FEATURES             Location/Qualifiers
     AlphaRep        27..38
                     /label="AWAlpha"
     KR              39..40
     AlphaRep1       49..60
                     /label="AWAlpha"
     KR              61..62
     KR              79..80
     AlphaRep2       125..136
                     /label="AWAlpha"
     KR              137..138
     KR              151..152
     AlphaRep3       157..168
                     /label="AWAlpha"
     KR              169..170
     AlphaRep        181..192
                     /label="AWAlpha"
     KR              193..194
```

```
     AlphaRep4       203..214
                     /label="AWAlpha"
     KR              215..216
     KR              232..233
ORIGIN
        1 MQFATAIVVA VLAYSVNSAA TPDAAAWCRY VGQPCGKVKR DGTPESEPWC
RYVGQPCGKS
       61 KREAEAACFL PGGPCAKAKR DANALAHAVA DALPAAEAEA YYKALALRDA
QPEPAPLAGA
      121 EAEAWCRYFG QPCGKAKRSA VAFEDISEQA KRDAEAWCRY VGQPCGKAKR
AAEAVAEAEA
      181 WCRYVGQPCG KVKRGATPEA EPWCRYVGKP CGKSKRGAEA ACDASGLCGK
AKREAHAIAE
      241 AVANIIEKY*
//
LOCUS       A_tetracladia_ppg1       264 aa            linear   UNA
FEATURES             Location/Qualifiers
     KR              36..37
     AlphaRep        48..57
                     /label="AWAlpha"
     KR              60..61
     AlphaRep        70..79
                     /label="AWAlpha"
     KR              82..83
     KR              156..157
     AlphaRep        168..177
                     /label="AWAlpha"
     KR              180..181
     KR              194..195
     KR              224..225
                     /label="RR"
     KR              245..246
ORIGIN
        1 MKFASTFIAV AIAVSVSATA IPEACSVEGQ SCMAIKRHAE AIATPDPWCW
HPGQVCVKSK
       61 RTATAYAEAW CWHPGQVCVK VKRAAEAFAE AIASPDEILD ARCESSECFD
ARLKIRELAG
      121 LVALTVRDAT PEAYIESLNL RDETKLRRDA SEHIEKREAE ADAEATPWCW
HPGQVCVKTK
      181 RSCGDAGSPC DKLKRAAAAL ANAVAEPAAE PQCLNPGQIC VKERREAEAT
AKAWCMTVPG
      241 CNMAKRDAHA LAAAANEVLA MYN*
//
LOCUS       A_sarcoides_ppg1       116 aa            linear   UNA
FEATURES             Location/Qualifiers
     AlphaRep        23..30
                     /label="AWAlpha"
     KR              31..32
     AlphaRep1       43..51
                     /label="AWAlpha"
     KR              52..53
     AlphaRep1       64..72
                     /label="AWAlpha"
     KR              73..74
     AlphaRep        87..94
                     /label="AWAlpha"
```

```
     KR              95..96
     AlphaRep1       107..115
                     /label="AWAlpha"
ORIGIN
        1 MKLSHALSIF LLAATATAVA IPCGAPGQPC KREALPAPVP EPRCGAVGQP
CKREAVPEPA
       61 PGPRCGAVGQ PCKREAEPEP APEPVPCGAP GQPCKREVEP EPAPEPRCGA
VGQPC*
//
LOCUS       C_torta_ppg1       271 aa            linear   UNA
FEATURES             Location/Qualifiers
     AlphaRep        27..37
                     /label="AWAlpha"
     KR              38..39
     AlphaRep        54..64
                     /label="AWAlpha"
     KR              67..68
                     /label="RR"
     AlphaRep        83..93
                     /label="AWAlpha"
     KR              94..95
     KR              114..115
     AlphaRep        157..167
                     /label="AWAlpha"
     KR              168..169
     AlphaRep        176..186
                     /label="AWAlpha"
     KR              189..190
                     /label="RR"
     AlphaRep        203..213
                     /label="AWAlpha"
     AlphaRep        221..231
                     /label="AWAlpha"
     KR              232..233
     AlphaRep        240..250
                     /label="AWAlpha"
     KR              253..254
ORIGIN
        1 MKFTSAVCVI ILAASASAIA IPEANAQHCG RPGQPCGKRD ASPFPEPDAE
ALAQHCGRPG
       61 QPCGKVRRAA EAMAEAFAEP EPQHCGRPGQ PCGKREVEIA ERCNLVGGAC
YEAKRLARDL
      121 AGVVASTIEG DADAFYDNLN LEDRETSDKI ARDAEAQHCG RPGQPCGKRD
AEAEAQHCGR
      181 PGQPCGKLRR AAEAIAEAVA EPQHCGRPGQ PCGRDALPEP QHCGRPGQPC
GKREAEAFAQ
      241 HCGRPGQPCG KAKRNAHALA HAADVAVSQL *
//
LOCUS       C_aeruginascens_ppg1       272 aa            linear
UNA
FEATURES             Location/Qualifiers
     AlphaRep        25..33
                     /label="AWAlpha"
     KR              34..35
     AlphaRep3       52..60
                     /label="AWAlpha?"
```

```
     KR              64..65
     KR              101..102
     KR              137..138
                     /label="RR"
     AlphaRep1       143..151
                     /label="AWAlpha"
     KR              152..153
                     /label="RR"
     AlphaRep4       162..170
                     /label="AWAlpha"
     KR              174..175
                     /label="RR"
     AlphaRep1       188..196
                     /label="AWAlpha"
     KR              201..202
     AlphaRep2       215..223
                     /label="AWAlpha"
     KR              228..229
     AlphaRep1       242..250
                     /label="AWAlpha"
     KR              254..255
ORIGIN
        1 MKFATAIFVI VLAATATATA LPEPTCGRPG QGCKREPLAL PEAEADAIAI
AICGSEGQPC
       61 ARVKRAAEAF ASAVSEPEAE PVDEMLLARC NLVGGACYEA KRMTRDLATE
VAATQEDPEA
      121 FYSSLDIEDE ETSEKIRRDA EAKCGRPGQG CRRDADAEPE AICGRPGQGC
RQLRRAAEAM
      181 ANALADAKCG RPGQGCKDKA KREASALAEA EADARCGRPG QGCKDKLKRE
AVAVSIAEAV
      241 AKCGRPGQGC RKAKRDAAAL ASAAEEALRS L*
//
LOCUS       G_debralockiae_ppg1       164 aa            linear   UNA
FEATURES             Location/Qualifiers
     AlphaRep        24..32
                     /label="AWAlpha"
     KR              33..34
                     /label="RR"
     AlphaRep        47..55
                     /label="AWAlpha"
     KR              56..57
     AlphaRep1       70..78
                     /label="AWAlpha"
     KR              79..80
     AlphaRep        89..97
                     /label="AWAlpha"
     KR              98..99
     AlphaRep        112..120
                     /label="AWAlpha"
     KR              121..122
     AlphaRep        131..139
                     /label="AWAlpha"
     KR              140..141
     AlphaRep        154..162
                     /label="AWAlpha"
ORIGIN
```

```
        1 MKYLSIIVLA AAVSAIAIPA PEPTCGKPGQ PCRRNPEPVA VALPEPTCGK
PGQPCKREAE
       61 PVAVAAPEPT CGKSGQPCKR EAAPAPEPTC GKPGQPCKRE AEPIAVAAPQ
PTCGKPGQPC
      121 KRKAAPAPEP TCGKPGQPCK REAEPVAVAA PEPTCGKPGQ PCK*
//
LOCUS       H_hymeniophilus_ppg1       243 aa            linear
UNA
FEATURES             Location/Qualifiers
     AlphaRep        18..27
                     /label="AWAlpha"
     KR              51..52
     AlphaRep        61..70
                     /label="AWAlpha"
     KR              72..73
     AlphaRep        86..95
                     /label="AWAlpha"
     KR              97..98
     AlphaRep        111..120
                     /label="AWAlpha"
     KR              122..123
     AlphaRep        132..141
                     /label="AWAlpha"
     AlphaRep        155..164
                     /label="AWAlpha"
     KR              166..167
     AlphaRep        180..189
                     /label="AWAlpha"
     AlphaRep        203..212
                     /label="AWAlpha"
     KR              214..215
     AlphaRep        224..233
                     /label="AWAlpha"
ORIGIN
        1 MKYLSLVLLA AAVSAAPWCG KPGQPCPRSA EAIGEPWCYL PGKPGNPCPK
KREAIPEPEP
       61 WCGKPGQPCP KKREAIPEPE PVAEPWCGKP GQPCPKKREA IPEPEPVAEP
WCGKPGQPCP
      121 KKREAIPEPE PWCGKPGQPC PRSAIAEPEP IAEPWCGKPG QPCPKKREAI
PEAEPVAEPW
      181 CGKPGQPCPR SAIAEPEPIA EPWCGKPGQP CPKKREAYHK KVQWCGKPGQ
PCPREAEAEA
      241 SA*
//
LOCUS       M_aquatica_ppg1       259 aa            linear   UNA
FEATURES             Location/Qualifiers
     AlphaRep        27..37
                     /label="AWAlpha"
     KR              40..41
     AlphaRep        46..56
                     /label="AWAlpha"
     KR              59..60
     KR              124..125
     KR              133..134
     AlphaRep        139..149
                     /label="AWAlpha"
```

```
     KR              152..153
     AlphaRep        160..170
                     /label="AWAlpha"
     KR              173..174
     AlphaRep        181..191
                     /label="AWAlpha"
     KR              194..195
     AlphaRep        208..218
                     /label="AWAlpha"
     KR              221..222
     KR              240..241
ORIGIN
        1 MKFTTTLFAV ALATSVSAIA LPASEPQNCW RQGQICNKEK RDAEAQNCWR
QGQICNKVKR
       61 AADAFAEAVA SPDDILDARC ESAECFSARL KLRELAGLVA VTVRDAKPEA
YLASLNLRDE
      121 FEIRRDALAF QEKRAADPQN CWRQGQICNK EKREAEALAQ NCWRQGQICN
KERREADPEA
      181 QNCWRQGQIC NKLKRAAAAV AEALAEPQNC WRQGQICNKE KRDAEAEAVA
SCGEACTMAK
      241 RDASALAAAA NEILYMAN*
//
LOCUS       N_alba_ppg1       148 aa            linear   UNA
FEATURES             Location/Qualifiers
     AlphaRep        35..45
                     /label="AWAlpha"
     KR              46..47
     AlphaRep1       66..76
                     /label="AWAlpha"
     KR              77..78
     AlphaRep        97..107
                     /label="AWAlpha"
     KR              108..109
     AlphaRep2       128..138
                     /label="AWAlpha"
     KR              139..140
                     /label="RR"
ORIGIN
        1 MKFTSSLCIG IAAFAINTTA LPVANPVPAA APEPCNGWHS PGQPCKREAD
PEPIPEPEPE
       61 PEADPCLYWH SPGQPCKREA XPEPIPEPEP EPEPEACNGW HSPGQPCKRE
AAPEPIPEPE
      121 PEPEAEPCNG WYSPGQPCRR RRRSFKV*
//
LOCUS       O_maius_ppg1?        87 aa            linear   UNA
FEATURES             Location/Qualifiers
     KR              25..26
     AlphaRep1       39..50
                     /label="AWAlpha?"
     KR              55..56
     AlphaRep        71..82
                     /label="AWAlpha?"
ORIGIN
        1 MKIASSVFLA VLVIGASTAP VPEAKRNPDP VLSPIMVARY GYPTGCGAPG
EPCPKREAAP
       61 EPVAAAIAEP RYGYPKGCGA PGEPCP*
```

```
//
LOCUS       P_destructans_ppg1       272 aa            linear   UNA
FEATURES             Location/Qualifiers
     AlphaRep        27..38
                     /label="AWAlpha"
     KR              39..40
     AlphaRep1       71..82
                     /label="AWAlpha"
     KR              83..84
     KR              129..130
     AlphaRep2       137..148
                     /label="AWAlpha"
     KR              149..150
     AlphaRep3       167..178
                     /label="AWAlpha"
     KR              179..180
     AlphaRep4       189..200
                     /label="AWAlpha"
     KR              201..202
     KR              227..228
ORIGIN
        1 MKYLATLCVA ALVAGVNSAA IAAAEPFCWR LGQPCDKVKR AAEAFAEAFD
EPIAEAEAFD
       61 EPIAEAEASA FCWRPGQICE KAKRAALALA HTVADANPEA EAFFDKLAID
EAFPEPEAVA
      121 DAEIADKVKR EAEAEAFCWR PGQPCGKVKR AADAIASALA EPAPEPFCQR
PGQLCGKVKR
      181 DAEAVAEAFC WRPGQPCGKA KREANALAEA AAEALEFGGL EKEQNSKRIF
RPPHYTTTAI
      241 FPTDPRLFHH FHEEQPYDCR KVDPNCVTVE A*
//
LOCUS       P_pannorum_ppg1       234 aa            linear   UNA
FEATURES             Location/Qualifiers
     AlphaRep        29..40
                     /label="AWAlpha"
     KR              41..42
     AlphaRep1       63..74
                     /label="AWAlpha"
     KR              75..76
     AlphaRep2       83..94
                     /label="AWAlpha"
     KR              95..96
     KR              146..147
     AlphaRep3       152..163
                     /label="AWAlpha"
     KR              164..165
     AlphaRep2       182..193
                     /label="AWAlpha"
     KR              194..195
     AlphaRep2       204..215
                     /label="AWAlpha"
     KR              216..217
ORIGIN
        1 MKYLATICVA ALVAGVNSAA IAVAEPEPIC WRPDQPCGKV KRAAEAFAEA
MAEPIAEPIA
```

```
       61 EPICWRPGQQ CGKVKREAEA AAICWRPGQP CGKAKRAALA LAHAVANAQP
EAEAFFDKLA
      121 VREAFPEPEP EPEAIANAAP IDDKLKREAE AICWRPGQPC GKVKRAAEAI
ASALAKSEPE
      181 AICWRPGQPC GKAKRDAEAE AEAICWRPGQ PCGKAKREAN ALAEAAAETL
ASL*
//
LOCUS       P_verrucosus_ppg1       230 aa            linear   UNA
FEATURES             Location/Qualifiers
     AlphaRep        29..38
                     /label="AWAlpha"
     KR              41..42
     AlphaRep        59..68
                     /label="AWAlpha"
     KR              71..72
     AlphaRep        83..94
                     /label="AWAlpha"
     KR              95..96
     KR              142..143
     AlphaRep1       148..159
                     /label="AWAlpha"
     KR              160..161
     AlphaRep        178..189
                     /label="AWAlpha"
     KR              190..191
     AlphaRep        200..211
                     /label="AWAlpha"
     KR              212..213
ORIGIN
        1 MKYLANLCLA ILVVGANSAA IAVAEPEPIC WRPGQPCGKV KRAAEAFAEA
IAEPIAEPIC
       61 WRPGQPCGKV KREAEAEAEA AAICWRPGQP CGKAKRAALA LAHAVANANP
EAGAFFDKLA
      121 IREAFPEPEA VAEAEPVDEK LKREAEAICW RPGQPCGKVK RAAEAIASAL
AEAAPEPICW
      181 RPGQPCGKAK RDAEAAAEAI CWRPGQPCGK AKREANALAE AAAEALASL*
//
LOCUS       S_lignicola_ppg1       238 aa            linear   UNA
FEATURES             Location/Qualifiers
     AlphaRep        45..56
                     /label="AWAlpha"
     KR              58..59
     AlphaRep1       82..93
                     /label="AWAlpha"
     KR              95..96
     AlphaRep2       123..134
                     /label="AWAlpha"
     KR              136..137
     AlphaRep3       184..195
                     /label="AWAlpha"
     KR              197..198
     AlphaRep4       223..234
                     /label="AWAlpha"
ORIGIN
        1 MKLTSVLAFA ILAVARAPIP EPEPEPEPAP VAEPEPHPVA VAEPRCTRMG
QPCGKKAKRE
```

```
       61 AVPEPVPVPK AAPNPVAEPN PVCTRMGQAC GKKAKREPIP EPVPEPEPAA
KAEPNPVAAP
      121 EPKCTRMGQP CGKKSKRVAV AEPEPEPEPM PVPLPEPQPE PEPLPEPAPA
AKAVPNPVAE
      181 PEPKCTRMGQ PCGRKEKREP VPEPQPAPVA KAAPNPVAEP DPECTRMGQP
CGRKFRA*
//
LOCUS       V_echinocandica_ppg1       244 aa            linear
UNA
FEATURES             Location/Qualifiers
     AlphaRep        26..34
                     /label="AWAlpha"
     KR              37..38
     AlphaRep        47..55
                     /label="AWAlpha"
     KR              60..61
     AlphaRep        70..78
                     /label="AWAlpha"
     KR              83..84
     AlphaRep        99..107
                     /label="AWAlpha"
     KR              108..109
     AlphaRep        114..122
                     /label="AWAlpha"
     KR              127..128
     AlphaRep        137..145
                     /label="AWAlpha"
     KR              150..151
     AlphaRep        166..174
                     /label="AWAlpha"
     KR              175..176
     AlphaRep        181..189
                     /label="AWAlpha"
     KR              194..195
     AlphaRep        204..212
                     /label="AWAlpha"
     KR              217..218
     AlphaRep        233..241
                     /label="AWAlpha"
ORIGIN
        1 MKFTNAIAIL FLTATTINAL PSAEPCTHVG QGCWKDKREA VAEPDPCTHV
GQGCWKHKLK
       61 RLAEAEAEPC THVGQGCWKE KQKRVAEAEA EADAAAEACT HVGQGCWKRE
ADPCTHVGQG
      121 CWKHKMKRVA EAYAEPCTHV GQGCWKEKQK RVAEAEAEAD AAAEACTHVG
QGCWKREADP
      181 CTHVGQGCWK HKMKRVAEAY AEPCTHVGQG CWKEKMKRVA EAEAEAEAAA
EACTHVGQGC
      241 WKQ*
//
LOCUS       B_calthae_ppg1       359 aa            linear   UNA
FEATURES             Location/Qualifiers
     AlphaRep        25..33
                     /label="AWAlpha"
     KR              34..35
     AlphaRep        48..56
```

```
                     /label="AWAlpha"
     KR              57..58
     AlphaRep        65..73
                     /label="AWAlpha"
     KR              74..75
     AlphaRep        80..88
                     /label="AWAlpha"
     KR              89..90
     AlphaRep        97..105
                     /label="AWAlpha"
     KR              106..107
     AlphaRep        118..126
                     /label="AWAlpha"
     KR              130..131
     AlphaRep        144..152
                     /label="AWAlpha"
     KR              153..154
     KR              166..167
     KR              179..180
     AlphaRep        221..229
                     /label="AWAlpha"
     KR              230..231
     AlphaRep1       236..244
                     /label="AWAlpha"
     KR              245..246
     AlphaRep        255..263
                     /label="AWAlpha"
     KR              266..267
     AlphaRep        276..284
                     /label="AWAlpha"
     KR              288..289
     AlphaRep2       306..314
                     /label="AWAlpha"
     KR              315..316
     AlphaRep        329..337
                     /label="AWAlpha"
     KR              341..342
ORIGIN
        1 MKFTNAIALA ILAATATAVA VPEPWCGRPG QPCKREAVAV AAPVAEAWCG
RPGQPCKRTP
       61 EAEAWCGRPG QPCKRDAEPW CGRPGQPCKR EALPEAWCGR PGQPCKRTPL
AEAEAEAWCG
      121 RPGQPCRKNK RAAEAVAEAF AEPWCGRPGQ PCKRDAEADV SEAAIKRCNM
VGGACFEAKR
      181 LARDLAEATA ETVEDSDLFL RSLNIETREV SEVVAREAEA WCGRPGQPCK
RDAEAWCRRP
      241 GQPCKREALA EAEAWCGRPG QPCKEKREAD PEAETWCGRP GQPCRAVKRA
AEAIAEALAE
      301 PIAEAWCGRH GQPCKREALA EAEANAEAWC GRPGQPCRKA KRDAFALAYA
ADVALAQL*
//
LOCUS       B_convoluta_ppg1       344 aa            linear   UNA
FEATURES             Location/Qualifiers
     AlphaRep        25..33
                     /label="AWAlpha"
     KR              34..35
```

```
     AlphaRep        50..58
                     /label="AWAlpha"
     KR              59..60
     AlphaRep        65..73
                     /label="AWAlpha"
     KR              74..75
     AlphaRep        82..90
                     /label="AWAlpha"
     KR              91..92
     AlphaRep        103..111
                     /label="AWAlpha"
     KR              115..116
     AlphaRep        129..137
                     /label="AWAlpha"
     KR              138..139
                     /label="RR"
     KR              151..152
     KR              164..165
     AlphaRep        206..214
                     /label="AWAlpha"
     KR              215..216
     AlphaRep        225..233
                     /label="AWAlpha"
     KR              234..235
     AlphaRep        240..248
                     /label="AWAlpha"
     KR              251..252
     AlphaRep        261..269
                     /label="AWAlpha"
     KR              273..274
     AlphaRep        291..299
                     /label="AWAlpha"
     KR              300..301
     AlphaRep        314..322
                     /label="AWAlpha"
     KR              326..327
ORIGIN
        1 MKFTNAIALA ILAATATAVA VPEPWCGRPG QPCKREAVAV AVAAPVAEAW
CGRPGQPCKR
       61 DAEPWCGRPG QPCKREALPE AWCGRPGQPC KRTPLAEAEA EAWCGRPGQP
CRKNKRAAEA
      121 VAEAFAEPWC GRPGQPCRRD AEADVSEAAI KRCNMVGGAC FEAKRLARDL
AEATAETVED
      181 ADAFLESLNI ETREVSEVVA REAEAWCGRP GQPCKREALA EAEAWCGRPG
QPCKRTAEPW
      241 CGRPGQPCKE KREADPEAEA WCGRPGQPCR AVKRAAEAIA EALAEPTAEA
WCGRPGQPCK
      301 REALAEAEAN AEAWCGRPGQ PCRKAKRDAF ALAYAADIAL AQL*
//
LOCUS       B_globosa_ppg1       393 aa            linear   UNA
FEATURES             Location/Qualifiers
     AlphaRep        25..33
                     /label="AWAlpha"
     KR              34..35
     AlphaRep        48..56
                     /label="AWAlpha"
```

```
     KR              57..58
     AlphaRep        65..73
                     /label="AWAlpha"
     KR              74..75
     AlphaRep        80..88
                     /label="AWAlpha"
     KR              89..90
     AlphaRep        97..105
                     /label="AWAlpha"
     KR              106..107
     AlphaRep        118..126
                     /label="AWAlpha"
     KR              130..131
     AlphaRep        144..152
                     /label="AWAlpha"
     KR              153..154
     KR              166..167
     KR              179..180
     AlphaRep        221..229
                     /label="AWAlpha"
     KR              230..231
     AlphaRep        236..244
                     /label="AWAlpha"
     KR              245..246
     AlphaRep        255..263
                     /label="AWAlpha"
     KR              264..265
     AlphaRep        274..282
                     /label="AWAlpha"
     KR              283..284
     AlphaRep        289..297
                     /label="AWAlpha"
     KR              300..301
     AlphaRep        310..318
                     /label="AWAlpha"
     KR              322..323
     AlphaRep        340..348
                     /label="AWAlpha"
     KR              349..350
     AlphaRep        363..371
                     /label="AWAlpha"
     KR              375..376
ORIGIN
        1 MKFTNAIALA ILAATATAVA VPEPWCGRPG QPCKREAVAV AAPVAEAWCG
RPGQPCKRTP
       61 EAEAWCGRPG QPCKRDAEPW CGRPGQPCKR EALPEAWCGR PGQPCKRTPL
AEAEAEAWCG
      121 RPGQPCRKNK RAAEAVAEAF AEPWCGRPGQ PCKRDAEADV SEAAIKRCNM
VGGACFEAKR
      181 LARDLAEATA ETVEDSDAFL ESLNIETREV SEVVAREAEA WCGRPGQPCK
RDAEAWCGRP
      241 GQPCKREALA EAEAWCGRPG QPCKREALAE AEAWCGRPGQ PCKRTAEPWC
GRPGQPCKEK
      301 READPEAEAW CGRPGQPCRA VKRAAEAIAE ALAEPTAEAW CGRPGQPCKR
EALAEAEANA
      361 EAWCGRPGQP CRKAKRDAFA LAYAADIALA QL*
```

```
//
LOCUS       B_narcissicola_ppg1       338 aa            linear   UNA
FEATURES             Location/Qualifiers
     AlphaRep        25..33
                     /label="AWAlpha"
     KR              34..35
     AlphaRep        42..50
                     /label="AWAlpha"
     KR              51..52
     AlphaRep        63..71
                     /label="AWAlpha"
     KR              75..76
     AlphaRep        89..97
                     /label="AWAlpha"
     KR              111..112
     KR              124..125
     AlphaRep        166..174
                     /label="AWAlpha"
     KR              175..176
     AlphaRep        181..189
                     /label="AWAlpha"
     KR              190..191
     AlphaRep        200..208
                     /label="AWAlpha"
     KR              209..210
     AlphaRep        219..227
                     /label="AWAlpha"
     KR              228..229
     AlphaRep        234..242
                     /label="AWAlpha"
     KR              245..246
     AlphaRep        255..263
                     /label="AWAlpha"
     KR              267..268
     AlphaRep        285..293
                     /label="AWAlpha"
     KR              294..295
     AlphaRep        308..316
                     /label="AWAlpha"
     KR              320..321
ORIGIN
        1 MKFTNAIALA ILAATATAVA VPEPWCGRPG QPCKREALPE AWCGRPGQPC
KRTPLAEAEA
       61 EAWCGRPGQP CRKNKRAAEA VAEAFAEPWC GRPGQPCKCD AEADVSEAAI
KRCNMVGGAC
      121 FEAKRLARDL AEATAETVED SDAFLESLNI ETREVSEVVA REAEAWCGRP
GQPCKRDAEA
      181 WCGRPGQPCK REALAEAEAW CGRPGQPCKR GALAEAEAWC GRPGQPCKRN
AEAWCGRPGQ
      241 PCKEKREADP EAEAWCGRPG QPCRAVKRAA EAIAEALAEP TAEAWCGRPG
QPCKREALAE
      301 AEANAEAWCG RPGQPCRKAK RDAFALAYAA DIALAQL*
//
LOCUS       B_squamosa_ppg1       391 aa            linear   UNA
FEATURES             Location/Qualifiers
     KR              32..33
```

```
     AlphaRep        46..54
                     /label="AWAlpha"
     KR              55..56
     AlphaRep        63..71
                     /label="AWAlpha"
     KR              72..73
     AlphaRep        78..86
                     /label="AWAlpha"
     KR              87..88
     AlphaRep        95..103
                     /label="AWAlpha"
     KR              104..105
     AlphaRep        116..124
                     /label="AWAlpha"
     KR              128..129
     AlphaRep        142..150
                     /label="AWAlpha"
     KR              151..152
     KR              164..165
     KR              177..178
     AlphaRep        219..227
                     /label="AWAlpha"
     KR              228..229
     AlphaRep        234..242
                     /label="AWAlpha"
     KR              243..244
     AlphaRep        253..261
                     /label="AWAlpha"
     KR              262..263
     AlphaRep        272..280
                     /label="AWAlpha"
     KR              281..282
     AlphaRep        287..295
                     /label="AWAlpha"
     KR              298..299
     AlphaRep        308..316
                     /label="AWAlpha"
     KR              320..321
     AlphaRep        338..346
                     /label="AWAlpha"
     KR              347..348
     AlphaRep        361..369
                     /label="AWAlpha"
     KR              373..374
ORIGIN
        1 MKFTNAIALA ILAATATAVA VPECGRPGQP CKREAVAVAA PVAEAWCGRP
GQPCKRTPEA
       61 EAWCGRPGQP CKRDAEPWCG RPGQPCKREA LPEAWCGRPG QPCKRTPLAE
AEAEAWCGRP
      121 GQPCRKNKRA AEAVAEAFAE PWCGRPGQPC KRDAEADISE AAIKRCNMVG
GACFEAKRLA
      181 RDLAEATAET VEDADAFLES LNIETREVSE VVAREAEAWC GRPGQPCKRD
AEAWCGRPGQ
      241 PCKREALAEA EAWCGRPGQP CKREALAEAE AWCGRPGQPC KRTAEPWCGR
PGQPCKEKRE
```

```
      301 ADPEAEAWCG RPGQPCRAVK RAAEAIAEAL AEPTAEAWCG RPGQPCKREA
LAEAEANAEA
      361 WCGRPGQPCR KAKRDAFALA YAADIALAQL *
//
LOCUS       B_aclada_ppg1       393 aa            linear   UNA
FEATURES             Location/Qualifiers
     AlphaRep        25..33
                     /label="AWAlpha"
     KR              34..35
     AlphaRep        48..56
                     /label="AWAlpha"
     KR              57..58
     AlphaRep        65..73
                     /label="AWAlpha"
     KR              74..75
     AlphaRep        80..88
                     /label="AWAlpha"
     KR              89..90
     AlphaRep        97..105
                     /label="AWAlpha"
     KR              106..107
     AlphaRep        118..126
                     /label="AWAlpha"
     KR              130..131
     AlphaRep        144..152
                     /label="AWAlpha"
     KR              153..154
     KR              166..167
     KR              179..180
     AlphaRep        221..229
                     /label="AWAlpha"
     KR              230..231
     AlphaRep        236..244
                     /label="AWAlpha"
     KR              245..246
     AlphaRep        255..263
                     /label="AWAlpha"
     KR              264..265
     AlphaRep        274..282
                     /label="AWAlpha"
     KR              283..284
     AlphaRep        289..297
                     /label="AWAlpha"
     KR              300..301
     AlphaRep        310..318
                     /label="AWAlpha"
     KR              322..323
     AlphaRep        340..348
                     /label="AWAlpha"
     KR              349..350
     AlphaRep        363..371
                     /label="AWAlpha"
     KR              375..376
ORIGIN
        1 MKFTNAIALA ILAATATAVA VPEPWCGRPG QPCKREAVAV AAPVAEAWCG
RPGQPCKRTP
```

```
       61 EAEAWCGRPG QPCKRDAEPW CGRPGQPCKR EALPEAWCGR PGQPCKRTPL
AEAEAEAWCG
      121 RPGQPCRKNK RAAEAVAEAF AEPWCGRPGQ PCKRDAEADV SEAAIKRCNM
VGGACFEAKR
      181 LARDLAEATA ETVEDADAFL ESLNIETREV SEVVAREAEA WCGRPGQPCK
RDAEAWCGRP
      241 GQPCKREALA EAEAWCGRPG QPCKREALAE AEAWCGRPGQ PCKRAAEPWC
GRPGQPCKEK
      301 READPEAEAW CGRPGQPCRA VKRAAEAIAE ALAEPTAEAW CGRPGQPCKR
EALAEAEANA
      361 EAWCGRPGQP CRKAKRDAFA LAYAADIALA QL*
//
LOCUS       B_byssoidea_ppg1       376 aa            linear   UNA
FEATURES             Location/Qualifiers
     AlphaRep        25..33
                     /label="AWAlpha"
     KR              34..35
     AlphaRep        48..56
                     /label="AWAlpha"
     KR              57..58
     AlphaRep        63..71
                     /label="AWAlpha"
     KR              72..73
     AlphaRep        80..88
                     /label="AWAlpha"
     KR              89..90
     AlphaRep        101..109
                     /label="AWAlpha"
     KR              113..114
     AlphaRep        127..135
                     /label="AWAlpha"
     KR              136..137
     KR              149..150
     KR              162..163
     AlphaRep        204..212
                     /label="AWAlpha"
     KR              213..214
     AlphaRep        219..227
                     /label="AWAlpha"
     KR              228..229
     AlphaRep        238..246
                     /label="AWAlpha"
     KR              247..248
     AlphaRep        257..265
                     /label="AWAlpha"
     KR              266..267
     AlphaRep        272..280
                     /label="AWAlpha"
     KR              283..284
     AlphaRep        293..301
                     /label="AWAlpha"
     KR              305..306
     AlphaRep        323..331
                     /label="AWAlpha"
     KR              332..333
     AlphaRep        346..354
```

```
                     /label="AWAlpha"
     KR              358..359
ORIGIN
        1 MKFTNAIALA ILAATATAVA VPEPWCGRPG QPCKREAVAV AAPVAEAWCG
RPGQPCKRDA
       61 EPWCGRPGQP CKREALPEAW CGRPGQPCKR TPLAEAEAEA WCGRPGQPCR
KNKRAAEAVA
      121 EAFAKPWCGR PGQPCKRDAE ADVSEAAIKR CNMVGGACFE AKRLARDLAE
ATAETVEDTD
      181 AFLESLNIET REVSEVVARE AEAWCGRPGQ PCKRDAEAWC GRPGQPCKRE
ALAEAEAWCG
      241 RPGQPCKREA LAEAEAWCGR PGQPCKRTAE PWCGRPGQPC KEKREADPEA
EAWCGRPGQP
      301 CRAVKRAAEA IAEALAEPTA EAWCGRPGQP CKREALAEAE ANAEAWCGRP
GQPCRKAKRD
      361 AFALAYAADI ALAQL*
//
LOCUS       B_cinerea_ppg1       392 aa            linear   UNA 28-
Dec-2021
DEFINITION
FEATURES             Location/Qualifiers
     AlphaRep        25..33
                     /label="AWAlpha"
     KR              34..35
     AlphaRep        48..56
                     /label="AWAlpha"
     KR              57..58
     AlphaRep        65..73
                     /label="AWAlpha"
     KR              74..75
     AlphaRep        80..88
                     /label="AWAlpha"
     KR              89..90
     AlphaRep        97..105
                     /label="AWAlpha"
     KR              106..107
     AlphaRep        118..126
                     /label="AWAlpha"
     KR              130..131
     AlphaRep        144..152
                     /label="AWAlpha"
     KR              153..154
     KR              166..167
     KR              179..180
     AlphaRep        221..229
                     /label="AWAlpha"
     KR              230..231
     AlphaRep        236..244
                     /label="AWAlpha"
     KR              245..246
     AlphaRep        255..263
                     /label="AWAlpha"
     KR              264..265
     AlphaRep        274..282
                     /label="AWAlpha"
     KR              283..284
```

```
     AlphaRep        289..297
                     /label="AWAlpha"
     KR              300..301
     AlphaRep        310..318
                     /label="AWAlpha"
     KR              322..323
     AlphaRep        340..348
                     /label="AWAlpha"
     KR              349..350
     AlphaRep        363..371
                     /label="AWAlpha"
     KR              375..376
ORIGIN
        1 MKFTNAIALA ILAATATAVA VPEPWCGRPG QPCKREAVAV AAPVAEPWCG
RPGQPCKRTP
       61 EAEAWCGRPG QPCKRDAEPW CGRPGQPCKR EALPEAWCGR PGQPCKRTPL
AEAEAEAWCG
      121 RPGQPCRKNK RAAEAVAEAF AEPWCGRPGQ PCKRDAEADV SEAAIKRCNM
VGGACFEAKR
      181 LARDLAEATA ETVEDSDLFL RSLNIETREV SEVVAREAEA WCGRPGQPCK
RDAEAWCGRP
      241 GQPCKREALA EAEAWCGRPG QPCKREALAE AEAWCGRPGQ PCKRTAEPWC
GRPGQPCKEK
      301 READPEAEAW CGRPGQPCRA VKRAAEAIAE ALAEPTAEAW CGRPGQPCKR
EALAEAEANA
      361 EAWCGRPGQP CRKAKRDAFA LAYAADVALA QL
//
LOCUS       B_deweyae_ppg1       391 aa            linear   UNA
FEATURES             Location/Qualifiers
     KR              32..33
     AlphaRep        46..54
                     /label="AWAlpha"
     KR              55..56
     AlphaRep2       63..71
                     /label="AWAlpha"
     KR              72..73
     AlphaRep3       78..86
                     /label="AWAlpha"
     KR              87..88
     AlphaRep        95..103
                     /label="AWAlpha"
     KR              104..105
     AlphaRep        116..124
                     /label="AWAlpha"
     KR              128..129
     AlphaRep3       142..150
                     /label="AWAlpha"
     KR              151..152
     KR              164..165
     KR              177..178
     AlphaRep2       219..227
                     /label="AWAlpha"
     KR              228..229
     AlphaRep        234..242
                     /label="AWAlpha"
     KR              243..244
```

```
     AlphaRep        253..261
                     /label="AWAlpha"
     KR              262..263
     AlphaRep        272..280
                     /label="AWAlpha"
     KR              281..282
     AlphaRep        287..295
                     /label="AWAlpha"
     KR              298..299
     AlphaRep        308..316
                     /label="AWAlpha"
     KR              320..321
     AlphaRep3       338..346
                     /label="AWAlpha"
     KR              347..348
     AlphaRep4       361..369
                     /label="AWAlpha"
     KR              373..374
ORIGIN
        1 MKFTNAIALA ILAATATAVA VPECGRPGQP CKREAVAVAA PVAEAWCGRP
GQPCKRTPEA
       61 EAWCGRPGEP CKRDAEPWCG HPGQPCKREA LPEAWCGRPG QPCKRTPLAE
AEAEAWCGRP
      121 GQPCRKNKRA AEAVAEAFAE PWCGHPGQPC KRDAEADISE AAIKRCNMVG
GACFEAKRLA
      181 RDLAEATAET VEDADAFLES LNIETREVSE VVAREAEAWC GRPGEPCKRD
AEAWCGRPGQ
      241 PCKREALAEA EAWCGRPGQP CKREALAEAE AWCGRPGQPC KRTAEPWCGR
PGQPCKEKRE
      301 ADPEAEAWCG RPGQPCRAVK RAAEAIAEAL AEPTAEAWCG HPGQPCKREA
LAEAEANAEA
      361 WCGLPGQPCR KAKRDAFALA YAADIALAQL *
//
LOCUS       B_elliptica_ppg1       391 aa            linear   UNA
FEATURES             Location/Qualifiers
     KR              32..33
     AlphaRep        46..54
                     /label="AWAlpha"
     KR              55..56
     AlphaRep2       63..71
                     /label="AWAlpha"
     KR              72..73
     AlphaRep2       78..86
                     /label="AWAlpha"
     KR              87..88
     AlphaRep2       95..103
                     /label="AWAlpha"
     KR              104..105
     AlphaRep        116..124
                     /label="AWAlpha"
     KR              128..129
     AlphaRep        142..150
                     /label="AWAlpha"
     KR              151..152
     KR              164..165
     KR              177..178
```

```
     AlphaRep        219..227
                     /label="AWAlpha"
     KR              228..229
     AlphaRep        234..242
                     /label="AWAlpha"
     KR              243..244
     AlphaRep        253..261
                     /label="AWAlpha"
     KR              262..263
     AlphaRep        272..280
                     /label="AWAlpha"
     KR              281..282
     AlphaRep        287..295
                     /label="AWAlpha"
     KR              298..299
     AlphaRep2       308..316
                     /label="AWAlpha"
     KR              320..321
     AlphaRep2       338..346
                     /label="AWAlpha"
     KR              347..348
     AlphaRep2       361..369
                     /label="AWAlpha"
     KR              373..374
ORIGIN
        1 MKFTNAIALA ILAATATAVA VPECGRPGQP CKREAVAVAA PVAEAWCGRP
GQPCKRTPEA
       61 EAWCGLPGQP CKRDAEPWCG LPGQPCKREA LPEAWCGLPG QPCKRTPLAE
AEAEAWCGRP
      121 GQPCRKNKRA AEAVAEAFAE PWCGRPGQPC KRDAEADISE AAIKRCNMVG
GACFEAKRLA
      181 RDLAEATAET VEDADAFLES LNIETREVSE VVAREAEAWC GRPGQPCKRD
AEAWCGRPGQ
      241 PCKREALAEA EAWCGRPGQP CKREALAEAE AWCGRPGQPC KRTAEPWCGR
PGQPCKEKRE
      301 ADPEAEAWCG LPGQPCRAVK RAAEAIAEAL AEPTAEAWCG LPGQPCKREA
LAEAEANAEA
      361 WCGLPGQPCR KAKRDAFALA YAADIALAQL *
//
LOCUS       B_fabae_ppg1       393 aa            linear   UNA
FEATURES             Location/Qualifiers
     AlphaRep        25..33
                     /label="AWAlpha"
     KR              34..35
     AlphaRep        48..56
                     /label="AWAlpha"
     KR              57..58
     AlphaRep        65..73
                     /label="AWAlpha"
     KR              74..75
     AlphaRep        80..88
                     /label="AWAlpha"
     KR              89..90
     AlphaRep        97..105
                     /label="AWAlpha"
     KR              106..107
```

```
     AlphaRep        118..126
                     /label="AWAlpha"
     KR              130..131
     AlphaRep        144..152
                     /label="AWAlpha"
     KR              153..154
     KR              166..167
     KR              179..180
     AlphaRep        221..229
                     /label="AWAlpha"
     KR              230..231
     AlphaRep        236..244
                     /label="AWAlpha"
     KR              245..246
     AlphaRep        255..263
                     /label="AWAlpha"
     KR              264..265
     AlphaRep        274..282
                     /label="AWAlpha"
     KR              283..284
     AlphaRep        289..297
                     /label="AWAlpha"
     KR              300..301
     AlphaRep        310..318
                     /label="AWAlpha"
     KR              322..323
     AlphaRep        340..348
                     /label="AWAlpha"
     KR              349..350
     AlphaRep        363..371
                     /label="AWAlpha"
     KR              375..376
ORIGIN
        1 MKFTNAIALA ILAATATAVA VPEPWCGRPG QPCKREAVAV AAPVAEPWCG
RPGQPCKRTP
       61 EAEAWCGRPG QPCKRDAEPW CGRPGQPCKR EALPEAWCGR PGQPCKRTPL
AEAEAEAWCG
      121 RPGQPCRKNK RAAEAVAEAF AEPWCGRPGQ PCKRDAEADV SEAAIKRCNM
VGGACFEAKR
      181 LARDLAEATA ETVEDSDLFL RSLNIETREV SEVVAREAEA WCGRPGQPCK
RDAEAWCGRP
      241 GQPCKREALA EAEAWCGRPG QPCKREALAE AEAWCGRPGQ PCKRTAEPWC
GRPGQPCKEK
      301 READPEAEAW CGRPGQPCRA VKRAAEAIAE ALAEPTAEAW CGRPGQPCKR
EALAEAEDNA
      361 EAWCGRPGQP CRKAKRDAFA LAYAADVALA QL*
//
LOCUS       B_fragariae_ppg1       446 aa            linear   UNA
FEATURES             Location/Qualifiers
     AlphaRep        25..33
                     /label="AWAlpha"
     KR              34..35
     AlphaRep        48..56
                     /label="AWAlpha"
     KR              57..58
     AlphaRep        65..73
```

```
                     /label="AWAlpha"
     KR              74..75
     AlphaRep        80..88
                     /label="AWAlpha"
     KR              89..90
     AlphaRep        97..105
                     /label="AWAlpha"
     KR              106..107
     AlphaRep        118..126
                     /label="AWAlpha"
     KR              130..131
     AlphaRep        144..152
                     /label="AWAlpha"
     KR              153..154
     KR              166..167
     KR              179..180
     AlphaRep        221..229
                     /label="AWAlpha"
     KR              230..231
     AlphaRep        236..244
                     /label="AWAlpha"
     KR              245..246
     AlphaRep        255..263
                     /label="AWAlpha"
     KR              264..265
     AlphaRep        274..282
                     /label="AWAlpha"
     KR              283..284
     AlphaRep        289..297
                     /label="AWAlpha"
     KR              298..299
     AlphaRep        308..316
                     /label="AWAlpha"
     KR              317..318
     AlphaRep        327..335
                     /label="AWAlpha"
     KR              336..337
     AlphaRep        342..350
                     /label="AWAlpha"
     KR              353..354
     AlphaRep        363..371
                     /label="AWAlpha"
     KR              375..376
     AlphaRep        393..401
                     /label="AWAlpha"
     KR              402..403
     AlphaRep        416..424
                     /label="AWAlpha"
     KR              428..429
ORIGIN
        1 MKFTNAIALA ILAATATAVA VPEPWCGRPG QPCKREAVAV AAPVAEAWCG
RPGQPCKRTP
       61 EAEAWCGRPG QPCKRDAEPW CGRPGQPCKR EALPEAWCGR PGQPCKRTPL
AEAEAEAWCG
      121 RPGQPCRKNK RAAEAVAEAF AEPWCGRPGQ PCKRDAEADV SEAAIKRCNM
VGGACFEAKR
```

```
      181 LARDLAEATA ETVEDSDAFL ESLNIETREV SEVVAREAEA WCGRPGQPCK
RDAEAWCGRP
      241 GQPCKREALA EAEAWCGRPG QPCKREALAE AEAWCGRPGQ PCKRTAEPWC
GRPGQPCKRE
      301 ALAEAEAWCG RPGQPCKREA LAEAEAWCGR PGQPCKRTAE PWCGRPGQPC
KEKREADPEA
      361 EAWCGRPGQP CRAVKRAAEA IAEALAEPTA EAWCGRPGQP CKREALAEAE
ANAEAWCGRP
      421 GQPCRKAKRD AFALAYAADI ALAQL*
//
LOCUS       B_galanthina_ppg1       393 aa            linear   UNA
FEATURES             Location/Qualifiers
     AlphaRep        25..33
                     /label="AWAlpha"
     KR              34..35
                     /label="RR"
     AlphaRep        48..56
                     /label="AWAlpha"
     KR              57..58
     AlphaRep        65..73
                     /label="AWAlpha"
     KR              74..75
     AlphaRep        80..88
                     /label="AWAlpha"
     KR              89..90
     AlphaRep        97..105
                     /label="AWAlpha"
     KR              106..107
     AlphaRep        118..126
                     /label="AWAlpha"
     KR              130..131
     AlphaRep        144..152
                     /label="AWAlpha"
     KR              153..154
     KR              166..167
     KR              179..180
     AlphaRep        221..229
                     /label="AWAlpha"
     KR              230..231
     AlphaRep        236..244
                     /label="AWAlpha"
     KR              245..246
     AlphaRep        255..263
                     /label="AWAlpha"
     KR              264..265
     AlphaRep        274..282
                     /label="AWAlpha"
     KR              283..284
     AlphaRep        289..297
                     /label="AWAlpha"
     KR              300..301
     AlphaRep        310..318
                     /label="AWAlpha"
     KR              322..323
     AlphaRep        340..348
                     /label="AWAlpha"
```

```
     KR              349..350
     AlphaRep        363..371
                     /label="AWAlpha"
     KR              375..376
ORIGIN
        1 MKFTNAIALA ILAATATAVA VPEPWCGRPG QPCRREAVAV AAPVAEAWCG
RPGQPCKRTP
       61 EAEAWCGRPG QPCKRDAEPW CGRPGQPCKR EALPEAWCGR PGQPCKRTPL
AEAEAEAWCG
      121 RPGQPCRKNK RAAEAVAEAF AEPWCGRPGQ PCKRDAEADV SEAAIKRCNM
VGGACFEAKR
      181 LARDLAEATA ETVEDSDAFL ESLNIETREV SEVVAREAEA WCGRPGQPCK
RDVEAWCGRP
      241 GQPCKREALA EAEAWCGRPG QPCKREALAE AEAWCGRPGQ PCKRTAEPWC
GRPGQPCKEK
      301 READPEAEAW CGRPGQPCRA VKRAAEAIAE ALAEPTAEAW CGRPGQPCKR
EALAEAEANA
      361 EAWCGRPGQP CRKAKRDAFA LAYAADIALA QL*
//
LOCUS       B_hyacinthi_ppg1       374 aa            linear   UNA
FEATURES             Location/Qualifiers
     AlphaRep        25..33
                     /label="AWAlpha"
     KR              34..35
     AlphaRep        48..56
                     /label="AWAlpha"
     KR              57..58
     AlphaRep        65..73
                     /label="AWAlpha"
     KR              74..75
     AlphaRep        80..88
                     /label="AWAlpha"
     KR              89..90
     AlphaRep        97..105
                     /label="AWAlpha"
     KR              106..107
     AlphaRep        118..126
                     /label="AWAlpha"
     KR              130..131
     AlphaRep        144..152
                     /label="AWAlpha"
     KR              153..154
     KR              166..167
     KR              179..180
     AlphaRep        221..229
                     /label="AWAlpha"
     KR              230..231
     AlphaRep        236..244
                     /label="AWAlpha"
     KR              245..246
     AlphaRep        255..263
                     /label="AWAlpha"
     KR              264..265
     AlphaRep        270..278
                     /label="AWAlpha"
     KR              281..282
```

```
     AlphaRep        291..299
                     /label="AWAlpha"
     KR              303..304
     AlphaRep        321..329
                     /label="AWAlpha"
     KR              330..331
     AlphaRep        344..352
                     /label="AWAlpha"
     KR              356..357
ORIGIN
        1 MKFTNAIALA ILAATATAVA VPEPWCGRPG QPCKREAVAV AAPVAEAWCG
RPGQPCKRTS
       61 EAEAWCGRPG QPCKRDAEPW CGRPGQPCKR EALPKAWCGR PGQPCKRTPL
AKAEAEAWCG
      121 RPGQPCRKNK RAAEAVAEAF AEPWCGRPGQ PCKRDAEAEV SEAAIKRCNM
VGGACFEAKR
      181 LARDLAEATA ETVEDSDAFL ESLNIETREV SEVVAREAEA WCGRPGQPCK
RDAEAWCGRP
      241 GQPCKREALA EAEAWCGRPG QPCKRTAEAW CGRPGQPCKE KREADPEAEA
WCGRPGQPCS
      301 AVKRAAEAIA EALAEPTAEA WCGRPGQPCK REALAEAEAN AEAWCGRPGQ
PCRKAKRDAF
      361 ALAYAADIAL AQL*
//
LOCUS       B_medusae_ppg1       393 aa            linear   UNA
FEATURES             Location/Qualifiers
     AlphaRep        25..33
                     /label="AWAlpha"
     KR              34..35
     AlphaRep        48..56
                     /label="AWAlpha"
     KR              57..58
     AlphaRep        65..73
                     /label="AWAlpha"
     KR              74..75
     AlphaRep        80..88
                     /label="AWAlpha"
     KR              89..90
     AlphaRep        97..105
                     /label="AWAlpha"
     KR              106..107
     AlphaRep        118..126
                     /label="AWAlpha"
     KR              130..131
     AlphaRep        144..152
                     /label="AWAlpha"
     KR              153..154
     KR              166..167
     KR              179..180
     AlphaRep        221..229
                     /label="AWAlpha"
     KR              230..231
     AlphaRep        236..244
                     /label="AWAlpha"
     KR              245..246
     AlphaRep        255..263
```

```
                     /label="AWAlpha"
     KR              264..265
     AlphaRep        274..282
                     /label="AWAlpha"
     KR              283..284
     AlphaRep        289..297
                     /label="AWAlpha"
     KR              300..301
     AlphaRep        310..318
                     /label="AWAlpha"
     KR              322..323
     AlphaRep        340..348
                     /label="AWAlpha"
     KR              349..350
     AlphaRep        363..371
                     /label="AWAlpha"
     KR              375..376
ORIGIN
        1 MKFTNAIALA ILAATATAVA VPEPWCGRPG QPCKREAVAV AAPVAEAWCG
RPGQPCKRTP
       61 EAEAWCGRPG QPCKRDAEPW CGRPGQPCKR EALPEAWCGR PGQPCKRTPL
AEAEAEAWCG
      121 RPGQPCRKNK RAAEAVAEAF AEPWCGRPGQ PCKRDAEADV SEAAIKRCNM
VGGACFEAKR
      181 LARDLAEATA ETVEDSDLFL RSLNIETREV SEVVAREAEA WCGRPGQPCK
RDAEAWCGRP
      241 GQPCKREALA EAEAWCGRPG QPCKREALAE AEAWCGRPGQ PCKRTAEPWC
GRPGQPCKEK
      301 READPEAEAW CGRPGQPCRA VKRAAEAIAE ALAEPTAEAW CGRPGQPCKR
EALAEAEANA
      361 EAWCGRPGQP CRKAKRDAFA LAYAADVALA QL*
//
LOCUS       B_paeoniae_ppg1       344 aa            linear   UNA
FEATURES             Location/Qualifiers
     AlphaRep        25..33
                     /label="AWAlpha"
     KR              34..35
     AlphaRep        48..56
                     /label="AWAlpha"
     KR              57..58
     AlphaRep        65..73
                     /label="AWAlpha"
     KR              74..75
     AlphaRep        86..94
                     /label="AWAlpha"
     KR              98..99
     AlphaRep        112..120
                     /label="AWAlpha"
     KR              121..122
     KR              134..135
     KR              147..148
     AlphaRep        189..197
                     /label="AWAlpha"
     KR              198..199
     AlphaRep1       206..214
                     /label="AWAlpha"
```

```
     KR              215..216
     AlphaRep        225..233
                     /label="AWAlpha"
     KR              234..235
     AlphaRep        240..248
                     /label="AWAlpha"
     KR              251..252
     AlphaRep        261..269
                     /label="AWAlpha"
     KR              273..274
     AlphaRep        291..299
                     /label="AWAlpha"
     KR              300..301
     AlphaRep        314..322
                     /label="AWAlpha"
     KR              326..327
ORIGIN
        1 MKFTNAIALA ILAATATAVA VPEPWCGRPG QPCKREAVAV AAPVAEAWCG
RPGQPCKREA
       61 LPEAWCGRPG QPCKRTPLAE AEAEAWCGRP GQPCRKNKRA AEAVAEAFAE
PWCGRPGQPC
      121 KRDAEADVSE AAIKRCNMVG GACFEAKRLA RDLAEATAET VEDADAFLES
LNIETREVSE
      181 VVAREAEAWC GRPGQPCKRE ALVEAWCGRS GQPCKREALA EAEAWCGRPG
QPCKRTAEPW
      241 CGRPGQPCKE KREADPEAEA WCGRPGQPCR AVKRAAEAIA EALAEPTAEA
WCGRPGQPCK
      301 REALAEAEAN AEAWCGRPGQ PCRKAKRDTF ALAYAADIAL AQL*
//
LOCUS       B_porri_ppg1       169 aa            linear   UNA
FEATURES             Location/Qualifiers
     AlphaRep        25..33
                     /label="AWAlpha"
     KR              34..35
     AlphaRep        48..56
                     /label="AWAlpha"
     KR              57..58
     AlphaRep        65..73
                     /label="AWAlpha"
     KR              74..75
     AlphaRep        86..94
                     /label="AWAlpha"
     KR              98..99
     AlphaRep        116..124
                     /label="AWAlpha"
     KR              125..126
     AlphaRep        139..147
                     /label="AWAlpha"
     KR              151..152
ORIGIN
        1 MKFTNAIALA MLAATATAVA VPEPWCGRPG QPCKREAVAV AAPVAEAWCG
RPGQPCKRTP
       61 EAEAWCGRPG QPCKRTPLAE AEAEAWCGRP GQPCRAVKRA AEAIAEALAE
PTAEAWCGRP
      121 GQPCKREALA EAEANAEAWC GRPGQPCRKA KRDAFALAYA ADIALAQL*
//
```

```
LOCUS       B_pseudocinerea_ppg1       374 aa            linear
UNA
FEATURES             Location/Qualifiers
     AlphaRep        25..33
                     /label="AWAlpha"
     KR              34..35
     AlphaRep        48..56
                     /label="AWAlpha"
     KR              57..58
     AlphaRep        65..73
                     /label="AWAlpha"
     KR              74..75
     AlphaRep        80..88
                     /label="AWAlpha"
     KR              89..90
     AlphaRep        97..105
                     /label="AWAlpha"
     KR              106..107
     AlphaRep        118..126
                     /label="AWAlpha"
     KR              130..131
     AlphaRep        144..152
                     /label="AWAlpha"
     KR              153..154
     KR              166..167
     KR              179..180
     AlphaRep        221..229
                     /label="AWAlpha"
     KR              230..231
     AlphaRep        236..244
                     /label="AWAlpha"
     KR              245..246
     AlphaRep        255..263
                     /label="AWAlpha"
     KR              264..265
     AlphaRep        270..278
                     /label="AWAlpha"
     KR              281..282
     AlphaRep        291..299
                     /label="AWAlpha"
     KR              303..304
     AlphaRep        321..329
                     /label="AWAlpha"
     KR              330..331
     AlphaRep        344..352
                     /label="AWAlpha"
     KR              356..357
ORIGIN
        1 MKFTNAIALA ILAATATAVA VPEPWCGRPG QPCKREAVAV AAPVAEPWCG
RPGQPCKRTP
       61 EAEAWCGRPG QPCKRDAEPW CGRPGQPCKR EALPEAWCGR PGQPCKRTPL
AEAEAEAWCG
      121 RPGQPCRKNK RAAEAVAEAF AEPWCGRPGQ PCKRDAEADV SEAAIKRCNM
VGGACFEAKR
      181 LARDLAEATA ETVEDSDLFL RSLNIETREV SEVVAREAEA WCGRPGQPCK
RDAEAWCGRP
```

```
      241 GQPCKREALA EAEAWCGRPG QPCKRTAEPW CGRPGQPCKE KREADPEAEA
WCGRPGQPCR
      301 AVKRAAEAIA EALAEPTAEA WCGRPGQPCK REALAEAEAN AEAWCGRPGQ
PCRKAKRDAF
      361 ALAYAADVAL AQL*
//
LOCUS       B_sinoallii_ppg1       374 aa            linear   UNA
FEATURES             Location/Qualifiers
     AlphaRep        25..33
                     /label="AWAlpha"
     KR              34..35
     AlphaRep        48..56
                     /label="AWAlpha"
     KR              57..58
     AlphaRep        65..73
                     /label="AWAlpha"
     KR              74..75
     AlphaRep        80..88
                     /label="AWAlpha"
     KR              89..90
     AlphaRep        97..105
                     /label="AWAlpha"
     KR              106..107
     AlphaRep        118..126
                     /label="AWAlpha"
     KR              130..131
     AlphaRep        144..152
                     /label="AWAlpha"
     KR              153..154
     KR              166..167
     KR              179..180
     AlphaRep        221..229
                     /label="AWAlpha"
     KR              230..231
     AlphaRep        236..244
                     /label="AWAlpha"
     KR              245..246
     AlphaRep        255..263
                     /label="AWAlpha"
     KR              264..265
     AlphaRep        270..278
                     /label="AWAlpha"
     KR              281..282
     AlphaRep        291..299
                     /label="AWAlpha"
     KR              303..304
     AlphaRep        321..329
                     /label="AWAlpha"
     KR              330..331
     AlphaRep        344..352
                     /label="AWAlpha"
     KR              356..357
ORIGIN
        1 MKFTNAIALA ILAATATAVA VPEPWCGRPG QPCKREAVAV AAPVAEAWCG
RPGQPCKRTP
```

```
       61 EAEAWCGRPG QPCKRDAEPW CGRPGQPCKR EALPEAWCGR PGQPCKRTPL
AEAEAEAWCG
      121 RPGQPCHKNK RAAEAVAEAF AEPWCGRPGQ PCKRDAEADI SEAAIKRCNM
VGGACFEAKR
      181 LARDLAEATA ETVEDADAFL ESLNIETREV SEVVAREAEA WCGRPGQPCK
RDAEAWCGRP
      241 GQPCKREALA EAEAWCGRPG QPCKRTAEPW CGRPGQPCKE KREADPEAEA
WCGRPGQPCR
      301 AVKRAAEAIA EALAEPTAEA WCGRPGQPCK REALAEAEAN AEAWCGRPGQ
PCRKAKRDAF
      361 ALAYAADIAL AQL*
//
LOCUS       B_tulipae_ppg1       378 aa            linear   UNA
FEATURES             Location/Qualifiers
     AlphaRep        25..33
                     /label="AWAlpha"
     KR              34..35
     AlphaRep        48..56
                     /label="AWAlpha"
     KR              57..58
     AlphaRep        65..73
                     /label="AWAlpha"
     KR              74..75
     AlphaRep        80..88
                     /label="AWAlpha"
     KR              89..90
     AlphaRep        97..105
                     /label="AWAlpha"
     KR              106..107
     AlphaRep        118..126
                     /label="AWAlpha"
     KR              130..131
     AlphaRep        144..152
                     /label="AWAlpha"
     KR              153..154
     KR              166..167
     KR              179..180
     AlphaRep        221..229
                     /label="AWAlpha"
     KR              230..231
     AlphaRep        240..248
                     /label="AWAlpha"
     KR              249..250
     AlphaRep        259..267
                     /label="AWAlpha"
     KR              268..269
     AlphaRep        274..282
                     /label="AWAlpha"
     KR              285..286
     AlphaRep        295..303
                     /label="AWAlpha"
     KR              307..308
     AlphaRep        325..333
                     /label="AWAlpha"
     KR              334..335
     AlphaRep        348..356
```

```
                    /label="AWAlpha"
     KR             360..361
ORIGIN
        1 MKFTNAIALA ILAATATAVA VPEPWCGRPG QPCKREAVAV AAPVAETWCG
RPGQPCKRTP
       61 EAEAWCGRPG QPCKRDAEPW CGRPGQPCKR EALPEAWCGR PGQPCKRTPL
AEAEAEAWCG
      121 RPGQPCRKNK RAAEAVAEAF AEPWCGRPGQ PCKRDAEADV SEAAIKRCNM
VGGACFEAKR
      181 LARDLAEATA ETVEDSDAFL ESLNIETREV SEVVAREAEA WCGRPGQPCK
REALAEAEAW
      241 CGRPGQPCKR EALAEAEAWC GRPGQPCKRT AEPWCGRPGQ PCKEKREADP
EAEAWCGRPG
      301 QPCRAVKRAA EAIAEALAEP TAEAWCGRPG QPCKREALAE AEANAEAWCG
RPGQPCRKAK
      361 RDAFALAYAA DIALAQL*
//
LOCUS       C_camelliae_ppg1       359 aa            linear   UNA
FEATURES             Location/Qualifiers
     AlphaRep       25..33
                    /label="AWAlpha"
     KR             34..35
     AlphaRep       48..56
                    /label="AWAlpha"
     KR             57..58
     AlphaRep       65..73
                    /label="AWAlpha"
     KR             74..75
     AlphaRep       80..88
                    /label="AWAlpha"
     KR             89..90
     AlphaRep       97..105
                    /label="AWAlpha"
     KR             106..107
     AlphaRep       118..126
                    /label="AWAlpha"
     KR             130..131
     KR             155..156
     KR             168..169
     AlphaRep       210..218
                    /label="AWAlpha"
     KR             219..220
     AlphaRep       225..233
                    /label="AWAlpha"
     KR             234..235
     AlphaRep       240..248
                    /label="AWAlpha"
     KR             249..250
     AlphaRep       255..263
                    /label="AWAlpha"
     KR             266..267
     AlphaRep       276..284
                    /label="AWAlpha"
     KR             288..289
                    /label="RR"
     AlphaRep       306..314
```

```
                     /label="AWAlpha"
     KR              315..316
     AlphaRep        329..337
                     /label="AWAlpha"
     KR              341..342
ORIGIN
        1 MKFTNAIALA ILAATATAVA LPEPWCGRPG QPCKREAVAI AAPAPEPWCG
RPGQPCKRTP
       61 EAEAWCGRPG QPCKRDAEPW CGRPGQPCKR EALPEAWCGR PGQPCKRTAL
AEAEAEAWCG
      121 RPGQPCRKNK RAAEALAEAF AEPTEETDVD EAVVKRCNMV GGACFEAKRL
ARDLAEATAE
      181 TSEDATAFLE GLNIETREAS EIIAREAEAW CGRPGQPCKR SAEPWCGRPG
QPCKRDAEPW
      241 CGRPGQPCKR DAEPWCGRPG QPCKEKREAD PEAEAWCGRP GQPCRAVRRA
AEAMADALAE
      301 PTAEAWCGRP GQPCKREALA EAEANAEAWC GRPGQPCRKA KRDAMALAYA
ADVALAQL*
//
LOCUS       C_shiraiana_ppg1       327 aa            linear   UNA
FEATURES             Location/Qualifiers
     AlphaRep        25..33
                     /label="AWAlpha"
     KR              34..35
     AlphaRep        48..56
                     /label="AWAlpha"
     KR              57..58
     AlphaRep        63..71
                     /label="AWAlpha"
     KR              72..73
     AlphaRep        80..88
                     /label="AWAlpha"
     KR              89..90
     AlphaRep        101..109
                     /label="AWAlpha"
     KR              113..114
     KR              138..139
     KR              151..152
     KR              165..166
     AlphaRep        193..201
                     /label="AWAlpha"
     KR              202..203
     AlphaRep        208..216
                     /label="AWAlpha"
     KR              217..218
     AlphaRep        223..231
                     /label="AWAlpha"
     KR              234..235
     AlphaRep        244..252
                     /label="AWAlpha"
     KR              256..257
                     /label="RR"
     AlphaRep        274..282
                     /label="AWAlpha"
     KR              283..284
     AlphaRep        297..305
```

```
                    /label="AWAlpha"
     KR             309..310
ORIGIN
        1 MKFTNAIALA ILAATATAVA IPEPWCGRPG QPCKREAVAV AAPIPEPWCG
RPGQPCKRDA
       61 EPWCGRPGQP CKREALPEAW CGRPGQPCKR TPLAEAEAEA WCGRPGQPCR
KNKRAAEALA
      121 EAFAEPAAEA DVDEAVIKRC NMVGGACFEA KRLARDLAEA TAETKRDADA
FLEGLNIESR
      181 EVSEIVAREA EAWCGRPGQP CKRNAEAWCG RPGQPCKRDA EPWCGRPGQP
CKEKREADPE
      241 AEAWCGRPGQ PCRAVRRAAE AMAEALAEPT AEAWCGRPGQ PCKREALAEA
EANAEAWCGR
      301 PGQPCRKAKR DAFALAYAAD VVLAQL*
//
LOCUS       M_curreyana_ppg1       342 aa            linear   UNA
FEATURES             Location/Qualifiers
     AlphaRep       25..33
                    /label="AWAlpha"
     KR             34..35
     AlphaRep       48..56
                    /label="AWAlpha"
     KR             57..58
     AlphaRep       63..71
                    /label="AWAlpha"
     KR             72..73
     AlphaRep       80..88
                    /label="AWAlpha"
     KR             89..90
     AlphaRep       101..109
                    /label="AWAlpha"
     KR             113..114
     AlphaRep       127..135
                    /label="AWAlpha"
     KR             136..137
     KR             149..150
     KR             162..163
     AlphaRep       204..212
                    /label="AWAlpha"
     KR             213..214
     AlphaRep       219..227
                    /label="AWAlpha"
     KR             228..229
     AlphaRep       238..246
                    /label="AWAlpha"
     KR             249..250
     AlphaRep       259..267
                    /label="AWAlpha"
     KR             271..272
     AlphaRep       289..297
                    /label="AWAlpha"
     KR             298..299
     AlphaRep       312..320
                    /label="AWAlpha"
     KR             324..325
ORIGIN
```

```
        1 MKFTNAIALA ILAATATAVA VPEPWCGRPG QPCKREAVAI ADPIAEAWCG
RPGQPCKRDA
       61 EPWCGRPGQP CKREALPEAW CGRPGQPCKR SPLAEAEAEA WCGRPGQPCR
KNKRAAEAVA
      121 EAFAEPWCGR PGQPCKRDAE ADVDEAVIKR CNMVGGACFE AKRLARDLAE
ATAETVEDPD
      181 TFLEGLNIET REVSEIVARE AEAWCGRPGQ PCKRSAEPWC GRPGQPCKRD
AQPEAEPWCG
      241 RPGQPCKEKR EADAEAEAWC GRPGQPCRAV KRAAEAIAEA LAEPTAEAWC
GRPGQPCKRE
      301 ALAEAEANAE AWCGRPGQPC RKAKRDAFAL AYAADVALAQ L*
//
LOCUS       M_duriaeana_ppg1       342 aa            linear   UNA
FEATURES             Location/Qualifiers
     AlphaRep        25..33
                     /label="AWAlpha"
     KR              34..35
     AlphaRep        48..56
                     /label="AWAlpha"
     KR              57..58
     AlphaRep        63..71
                     /label="AWAlpha"
     KR              72..73
     AlphaRep        80..88
                     /label="AWAlpha"
     KR              89..90
     AlphaRep        101..109
                     /label="AWAlpha"
     KR              113..114
     AlphaRep        127..135
                     /label="AWAlpha"
     KR              136..137
     KR              149..150
     KR              162..163
     AlphaRep        204..212
                     /label="AWAlpha"
     KR              213..214
     AlphaRep        219..227
                     /label="AWAlpha"
     KR              228..229
     AlphaRep        238..246
                     /label="AWAlpha"
     KR              249..250
     AlphaRep        259..267
                     /label="AWAlpha"
     KR              271..272
     AlphaRep        289..297
                     /label="AWAlpha"
     KR              298..299
     AlphaRep        312..320
                     /label="AWAlpha"
     KR              324..325
ORIGIN
        1 MKFTNAIALA ILAATATAVA VPEPWCGRPG QPCKREAVAV ADPVAEAWCG
RPGQPCKREA
```

```
        61 EPWCGRPGQP CKREALPEAW CGRPGQPCKR SPLAEAEAEA WCGRPGQPCR
KNKRAAEAVA
       121 EAFAEPWCGR PGQPCKRDAE ADVDEAVVKR CNMVGGACFE AKRLARDLAE
ATAETVEDPD
       181 AYLEGLNIET REVSEIVARE AEAWCGRPGQ PCKRSAEPWC GRPGQPCKRD
AQPEAEPWCG
       241 RPGQPCKEKR EADPEAEAWC GRPGQPCRAV KRAAEAIAEA LAEPTAEAWC
GRPGQPCKRE
       301 ALAEAEANAE AWCGRPGQPC RKAKRDAFAL AYAADVALAQ L*
//
LOCUS       M_scirpicola_ppg1       342 aa            linear   UNA
FEATURES             Location/Qualifiers
     AlphaRep        25..33
                     /label="AWAlpha"
     KR              34..35
     AlphaRep        48..56
                     /label="AWAlpha"
     KR              57..58
     AlphaRep        63..71
                     /label="AWAlpha"
     KR              72..73
     AlphaRep        80..88
                     /label="AWAlpha"
     KR              89..90
     AlphaRep        101..109
                     /label="AWAlpha"
     KR              113..114
     AlphaRep        127..135
                     /label="AWAlpha"
     KR              136..137
     KR              149..150
     KR              162..163
     AlphaRep        204..212
                     /label="AWAlpha"
     KR              213..214
     AlphaRep        219..227
                     /label="AWAlpha"
     KR              228..229
     AlphaRep        238..246
                     /label="AWAlpha"
     KR              249..250
     AlphaRep        259..267
                     /label="AWAlpha"
     KR              271..272
     AlphaRep        289..297
                     /label="AWAlpha"
     KR              298..299
     AlphaRep        312..320
                     /label="AWAlpha"
     KR              324..325
ORIGIN
         1 MKFTNAIALA ILAATATAVA VPEPWCGRPG QPCKREAVAV ADPVAEAWCG
RPGQPCKREA
        61 EPWCGRPGQP CKREALPEAW CGRPGQPCKR SPLAEAEAEA WCGRPGQPCR
KNKRAAEAVA
```

```
      121 EAFAEPWCGR PGQPCKRDAE ADVDEAVVKR CNMVGGACFE AKRLARDLAE
ATAETVEDPE
      181 AYLEGLNIET REVSEIVARE AEAWCGRPGQ PCKRSAEPWC GRPGQPCKRD
AQPEAEPWCG
      241 RPGQPCKEKR EADPEAEAWC GRPGQPCRAV KRAAEAMAEA LAEPTAEAWC
GRPGQPCKRE
      301 ALAEAEANAE AWCGRPGQPC RKAKRDAFAL AYAADVALAQ L*
//
LOCUS       M_aucupariae_ppg1       316 aa            linear   UNA
FEATURES             Location/Qualifiers
     AlphaRep        27..35
                     /label="AWAlpha"
     KR              36..37
     AlphaRep        50..58
                     /label="AWAlpha"
     KR              59..60
     AlphaRep        65..73
                     /label="AWAlpha"
     KR              74..75
     AlphaRep        80..88
                     /label="AWAlpha"
     KR              89..90
     AlphaRep        97..105
                     /label="AWAlpha"
     KR              106..107
     AlphaRep        118..126
                     /label="AWAlpha"
     KR              130..131
     KR              155..156
     KR              168..169
     AlphaRep        210..218
                     /label="AWAlpha"
     KR              219..220
     AlphaRep1       233..241
                     /label="AWAlpha"
     KR              245..246
                     /label="RR"
     AlphaRep        263..271
                     /label="AWAlpha"
     KR              272..273
     AlphaRep        286..294
                     /label="AWAlpha"
     KR              298..299
ORIGIN
        1 MKFTNAIALA ILAATAAAVA VPEAQPWCGR PGQGCKREAL AAPVSEAQPW
CGRPGQGCKR
       61 EAEAWCGRPG QGCKRDAEPW CGRPGQGCKR EALPEAWCGR PGQGCKRAPL
PEAEAEAWCG
      121 RPGQGCRKTK RAAEALAEAL AEPEAEADVE EEVAKRCNMV GGACFEAKRL
ARDLAETVAE
      181 TTADPAAYLE SLNLETREVS EVIAREAEAW CGRPGQGCKR NAEAEADAEA
EAWCGRPGQP
      241 CRKVRRSAEA MAEALAEATP VAWCGRPGQG CKREALAEAE ANAEAWCGRP
GQGCRKAKRD
      301 AYALAYAADM VLKQL*
//
```

```
LOCUS       M_fructicola_ppg1       291 aa            linear   UNA
FEATURES             Location/Qualifiers
     AlphaRep        27..35
                     /label="AWAlpha"
     KR              36..37
     AlphaRep        54..62
                     /label="AWAlpha"
     KR              63..64
     AlphaRep        69..77
                     /label="AWAlpha"
     KR              78..79
     AlphaRep        84..92
                     /label="AWAlpha"
     KR              93..94
     AlphaRep        101..109
                     /label="AWAlpha"
     KR              110..111
     AlphaRep        122..130
                     /label="AWAlpha"
     KR              134..135
     KR              159..160
     KR              172..173
     AlphaRep        214..222
                     /label="AWAlpha"
     KR              223..224
     AlphaRep        237..245
                     /label="AWAlpha"
     KR              247..248
     AlphaRep        261..269
                     /label="AWAlpha"
     KR              273..274
ORIGIN
        1 MKFTNAIALA ILAATATAVA VPEAQPWCGR PGQGCKREAV AAPVAAPVSE
AQPWCGRPGQ
       61 GCKREAEAWC GRPGQGCKRD AEPWCGRPGQ GCKREALPEA WCGRPGQGCK
RAPLAEAEAE
      121 AWCGRPGQGC RKTKRAAEAL AEALAEPEAE ADVEEAVIKR CNMVGGACFE
AKRLARDLAE
      181 TVAETTSDPA AYLESLNLET REVSEVVARE AEAWCGRPGQ GCKRNAEAEA
DAEAEAWCGR
      241 PGQGCRKREA LAEAEANAEA WCGRPGQGCR KAKRDAYALA YAADMALKQL *
//
LOCUS       M_fructigena_ppg1       301 aa            linear   UNA
FEATURES             Location/Qualifiers
     AlphaRep        35..43
                     /label="AWAlpha"
     KR              44..45
     AlphaRep        50..58
                     /label="AWAlpha"
     KR              59..60
     AlphaRep        65..73
                     /label="AWAlpha"
     KR              74..75
     AlphaRep        82..90
                     /label="AWAlpha"
     KR              91..92
```

```
     AlphaRep        103..111
                     /label="AWAlpha"
     KR              115..116
     KR              140..141
     KR              153..154
     AlphaRep        195..203
                     /label="AWAlpha"
     KR              204..205
     AlphaRep        218..226
                     /label="AWAlpha"
     KR              230..231
                     /label="RR"
     AlphaRep        248..256
                     /label="AWAlpha"
     KR              257..258
     AlphaRep        271..279
                     /label="AWAlpha"
     KR              283..284
ORIGIN
        1 MKFTNAIALA ILAATATAVA VAAPAAAPVS EAQPWCGRPG QGCKREAEAW
CGRPGQGCKR
       61 DAEPWCGRPG QGCKREALPE AWCGRPGQGC KRAPLPEAEA EAWCGRPGQG
CRKTKRAAEA
      121 LAEALAEPEA EADVEEAVIK RCNMVGGACF EAKRLARDLA ETVAETTSDP
AAYLESLNLE
      181 TREVSEVVAR EAEAWCGRPG QGCKRNAEAE ADAEAEAWCG RPGQGCRKVR
RAAEVMAEAL
      241 AEATPVAWCG RPGQGCKREA LAEAEANAEA WCGRPGQGCR KAKRDAYALA
YAADLALKQL
      301 *
//
LOCUS       M_laxa_ppg1       320 aa            linear   UNA
FEATURES             Location/Qualifiers
     AlphaRep        27..35
                     /label="AWAlpha"
     KR              36..37
     AlphaRep        54..62
                     /label="AWAlpha"
     KR              63..64
     AlphaRep        69..77
                     /label="AWAlpha"
     KR              78..79
     AlphaRep        84..92
                     /label="AWAlpha"
     KR              93..94
     AlphaRep        101..109
                     /label="AWAlpha"
     KR              110..111
     AlphaRep        122..130
                     /label="AWAlpha"
     KR              134..135
     KR              159..160
     KR              172..173
     AlphaRep        214..222
                     /label="AWAlpha"
     KR              223..224
```

```
     AlphaRep        237..245
                     /label="AWAlpha"
     KR              249..250
                     /label="RR"
     AlphaRep        267..275
                     /label="AWAlpha"
     KR              276..277
     AlphaRep        290..298
                     /label="AWAlpha"
     KR              302..303
ORIGIN
        1 MKFTNAIALA ILAATATAVA VPEAQPWCGR PGQGCKREAI AAPAAAPVSE
AQPWCGRPGQ
       61 GCKREAEAWC GRPGQGCKRD AEPWCGRPGQ GCKREALPEA WCGRPGQGCK
RAPLPEAEAE
      121 AWCGRPGQGC RKTKRAAEAL AEALAEPEAE ADAEEAVIKR CNMVGGACFE
AKRLARDLAE
      181 TVAETTSDPA AYLESLNLET REVSEVVARE AEAWCGRPGQ GCKRNAEAEA
DAEAEAWCGR
      241 PGQGCRKVRR AAEAMAEALA EATPVAWCGR PGQGCKREAL AEAEANAEAW
CGRPGQGCRK
      301 AKRDAYALAY AADLALKQL*
//
LOCUS       M_polystroma_ppg1       301 aa            linear   UNA
FEATURES             Location/Qualifiers
     AlphaRep        35..43
                     /label="AWAlpha"
     KR              44..45
     AlphaRep        50..58
                     /label="AWAlpha"
     KR              59..60
     AlphaRep        65..73
                     /label="AWAlpha"
     KR              74..75
     AlphaRep        82..90
                     /label="AWAlpha"
     KR              91..92
     AlphaRep        103..111
                     /label="AWAlpha"
     KR              115..116
     KR              140..141
     KR              153..154
     AlphaRep        195..203
                     /label="AWAlpha"
     KR              204..205
     AlphaRep        218..226
                     /label="AWAlpha"
     KR              230..231
                     /label="RR"
     AlphaRep        248..256
                     /label="AWAlpha"
     KR              257..258
     AlphaRep        271..279
                     /label="AWAlpha"
     KR              283..284
ORIGIN
```

```
        1 MKFTNAIALA ILAATATAVA VAAPAAAPVS EAQPWCGRPG QGCKREAEAW
CGRPGQGCKR
       61 DAEPWCGRPG QGCKREALPE AWCGRPGQGC KRAPLPEAEA EAWCGRPGQG
CRKTKRAAEA
      121 LAEALAEPEA EADVEEAVIK RCNMVGGACF EAKRLARDLA ETVAETTSDP
AAYLKSLNLE
      181 TREVSEVVAR EAEAWCGRPG QGCKRNAEAE ADAEAEAWCG RPGQGCRKVR
RAAEAMAEAL
      241 AEATPVAWCG RPGQGCKREA LAEAEANAEA WCGRPGQGCR KAKRDAYALA
YAADLALKQL
      301 *
//
LOCUS       M_vacciniicorymbosi_ppg1       288 aa            linear
UNA
FEATURES             Location/Qualifiers
     AlphaRep        27..35
                     /label="AWAlpha"
     KR              36..37
     AlphaRep1       50..58
                     /label="AWAlpha"
     KR              59..60
     AlphaRep        65..73
                     /label="AWAlpha"
     KR              74..75
     AlphaRep2       86..94
                     /label="AWAlpha"
     KR              98..99
     KR              123..124
     KR              136..137
     AlphaRep        178..186
                     /label="AWAlpha"
     KR              187..188
     AlphaRep        201..209
                     /label="AWAlpha"
     KR              213..214
                     /label="RR"
     AlphaRep        231..239
                     /label="AWAlpha"
     KR              240..241
     AlphaRep        258..266
                     /label="AWAlpha"
     KR              270..271
ORIGIN
        1 MKFTNSIALA ILAATAIAVA VPETQPWCGR PGQPCKREAV AAPVSEAQPW
CGRPGKPCKR
       61 NAEPWCGRPG QPCKRAPLPE AEAEAWCGRP GQGCRKTKRA AEALAEALAE
PEAEADVEEA
      121 VVKRCNMVGG ACFEAKRLAR DLAETVAETT ADPAAYLESL TLETREASEV
AAREAEAWCG
      181 RPGQPCKRSA EAEADAEAEA WCGRPGQPCR KVRRSAEAMA EALAEATPVA
WCGRPGQPCK
      241 RGAEAEALAE AEANAEAWCG RPGQPCRKAK RNAYALAYAA DMALKQL*
//
LOCUS       S_borealis_ppg1       299 aa            linear   UNA
FEATURES             Location/Qualifiers
     AlphaRep        27..35
```

```
                     /label="AWAlpha"
     KR              36..37
     AlphaRep1       48..56
                     /label="AWAlpha"
     KR              57..58
     AlphaRep1       69..77
                     /label="AWAlpha"
     KR              81..82
     KR              106..107
     KR              119..120
     AlphaRep1       161..169
                     /label="AWAlpha"
     KR              170..171
     AlphaRep1       180..188
                     /label="AWAlpha"
     KR              189..190
     AlphaRep1       195..203
                     /label="AWAlpha"
     KR              206..207
     AlphaRep1       216..224
                     /label="AWAlpha"
     KR              228..229
                     /label="RR"
     AlphaRep1       246..254
                     /label="AWAlpha"
     KR              255..256
     AlphaRep1       269..277
                     /label="AWAlpha"
     KR              281..282
ORIGIN
        1 MKFTNAIALA ILAAIATAAA VPEAQPWCGR PGQACKREAV AGPVAEPWCG
RPGQPCKRTP
       61 LAEAEAEAWC GRPGQPCRKN KRAAEALAEA FAEPAAEADV DEAVIKRCNM
VGGACFEAKR
      121 LARDLAEATA ETVEDSTAFL ESLNIETREI SEVVAREAEA WCGRPGQPCK
RDAQPEAEAW
      181 CGRPGQPCKR NAEPWCGRPG QPCKEKREAD AEAEAWCGRP GQPCRAVRRA
AEAVAEALAE
      241 ATAEAWCGRP GQPCKREAIA EAEANAEAWC GRPGQPCRKA KRDAYALAYA
ADVALAQL*
//
LOCUS       S_cepivorum_ppg1       359 aa            linear   UNA
FEATURES             Location/Qualifiers
     AlphaRep        25..33
                     /label="AWAlpha"
     KR              34..35
     AlphaRep        48..56
                     /label="AWAlpha"
     KR              57..58
     AlphaRep        65..73
                     /label="AWAlpha"
     KR              74..75
     AlphaRep        80..88
                     /label="AWAlpha"
     KR              89..90
     AlphaRep        97..105
```

```
                     /label="AWAlpha"
     KR              106..107
     AlphaRep        118..126
                     /label="AWAlpha"
     KR              130..131
     KR              155..156
     KR              168..169
     KR              182..183
     AlphaRep        210..218
                     /label="AWAlpha"
     KR              219..220
     AlphaRep        225..233
                     /label="AWAlpha"
     KR              234..235
     AlphaRep        240..248
                     /label="AWAlpha"
     KR              249..250
     AlphaRep        255..263
                     /label="AWAlpha"
     KR              266..267
     AlphaRep        276..284
                     /label="AWAlpha"
     KR              288..289
                     /label="RR"
     AlphaRep        306..314
                     /label="AWAlpha"
     KR              315..316
     AlphaRep        329..337
                     /label="AWAlpha"
     KR              341..342
ORIGIN
        1 MKFTNAIALA ILAATATAVA VPEPWCGRPG QPCKREAVAV AAPIAEPWCG
RPGQPCKRSP
       61 EAEAWCGRPG QPCKRDAEPW CGRPGQPCKR EALPEAWCGR PGQPCKRTPL
AEAEAEAWCG
      121 RPGQPCRKNK RAAEALAEAF AEPAAEADVD EAVIKRCNMV GGACFEAKRL
ARDLAEATAE
      181 TKRDADAFLE GLNIESREVS EIVAREAEAW CGRPGQPCKR NAEAWCGRPG
QPCKREAEAW
      241 CGRPGQPCKR DAEPWCGRPG QPCKEKREAD PEAEAWCGRP GQPCRAVRRA
AEAMADALAE
      301 PTAEAWCGRP GQPCKREALA EAEANAEAWC GRPGQPCRKA KRDAFALAYA
ADVALAQL*
//
LOCUS       S_glacialis_ppg1       380 aa            linear   UNA
FEATURES             Location/Qualifiers
     KR              31..32
     AlphaRep        42..50
                     /label="AWAlpha"
     KR              53..54
     AlphaRep1       84..92
                     /label="AWAlpha"
     KR              93..94
     AlphaRep        105..113
                     /label="AWAlpha"
     KR              117..118
```

```
     KR              142..143
     KR              155..156
     AlphaRep        197..205
                     /label="AWAlpha"
     KR              206..207
     AlphaRep        212..220
                     /label="AWAlpha"
     KR              221..222
     AlphaRep        227..235
                     /label="AWAlpha"
     KR              236..237
     AlphaRep        242..250
                     /label="AWAlpha"
     KR              253..254
     AlphaRep        263..271
                     /label="AWAlpha"
     KR              275..276
                     /label="RR"
     AlphaRep        293..301
                     /label="AWAlpha"
     KR              302..303
     AlphaRep        310..318
                     /label="AWAlpha"
     KR              319..320
     AlphaRep        327..335
                     /label="AWAlpha"
     KR              336..337
     AlphaRep        350..358
                     /label="AWAlpha"
     KR              362..363
ORIGIN
        1 MKFTSAIAIA ILAATATAAA IPAPSSDQPS KRNITATQDI AWCGRPGQPC
HIKRDALPDI
       61 QAAAEAAVIC HPGQPCSQKH NPTWCGQPGR PCKRDALPDI QNVAWCGRPG
QPCHKNKRAA
      121 QALAEALAEP ADGADVDEAV VKRCNMVGGA CFEAKRLARD LAQTTAETSD
DAQTFLDGLN
      181 VETREASEII AREAEAWCGR PGQPCKREAE AWCGRPGQPC KREAEAWCGR
PGQPCKRDAE
      241 PWCGRPGQPC KEKREADPEA EAWCGRPGQP CRAVRRAAEA IAKALAEPTA
EAWCGRPGQP
      301 CKRDALAEPW CGRPGQPCKR EADAGAWCGR PGQPCKREAL AVAEADAEAW
CGRPGQPCRK
      361 AKRDAYALAY AADVALAQL*
//
LOCUS       S_sclerotiorum_ppg1       326 aa            linear   UNA
28-Dec-2021
DEFINITION
FEATURES             Location/Qualifiers
     sig_peptide     1..20
                     /note="Cleavage site: 20^21: AVA-IP"
                     /note="D-score: 0.798"
                     /note="D-cutoff: 0.45"
                     /note="Max C-score: 0.509"
                     /note="Max S-score: 0.962"
                     /note="Max Y-score: 0.677"
```

```
                     /note="S-mean: 0.901"
                     /note="Networks: SignalP-noTM"
     AlphaRep        25..33
                     /label="AWAlpha"
     KR              34..35
     AlphaRep        48..56
                     /label="AWAlpha"
     KR              57..58
     AlphaRep        63..71
                     /label="AWAlpha"
     KR              72..73
     AlphaRep        80..88
                     /label="AWAlpha"
     KR              89..90
     AlphaRep        101..109
                     /label="AWAlpha"
     KR              113..114
     KR              138..139
     KR              151..152
     KR              165..166
     AlphaRep        193..201
                     /label="AWAlpha"
     KR              202..203
     AlphaRep        208..216
                     /label="AWAlpha"
     KR              217..218
     AlphaRep        223..231
                     /label="AWAlpha"
     KR              234..235
     AlphaRep        244..252
                     /label="AWAlpha"
     KR              256..257
                     /label="RR"
     AlphaRep        274..282
                     /label="AWAlpha"
     KR              283..284
     AlphaRep        297..305
                     /label="AWAlpha"
     KR              309..310
ORIGIN
        1 MKFTNAIALA ILAATATAVA IPEPWCGRPG QPCKREAVAV AAPIPEPWCG
RPGQPCKRDA
       61 EPWCGRPGQP CKREALPEAW CGRPGQPCKR TPLAEAEAEA WCGRPGQPCR
KNKRAAEALA
      121 EAFAEPAAEA DVDEAVIKRC NMVGGACFEA KRLARDLAEA TAETKRDADA
FLEGLNIESR
      181 EVSEIVAREA EAWCGRPGQP CKRNAEAWCG RPGQPCKRDA EPWCGRPGQP
CKEKREADPE
      241 AEAWCGRPGQ PCRAVRRAAE AMAEALAEPT AEAWCGRPGQ PCKREALAEA
EANAEAWCGR
      301 PGQPCRKAKR DAFALAYAAD VVLAQL
//
LOCUS       S_trifoliorum_ppg1       230 aa            linear   UNA
FEATURES             Location/Qualifiers
     AlphaRep        25..33
                     /label="AWAlpha"
```

```
     KR              34..35
     AlphaRep1       46..54
                     /label="AWAlpha"
     KR              58..59
     KR              83..84
     KR              96..97
     KR              110..111
     AlphaRep2       138..146
                     /label="AWAlpha"
     KR              147..148
     KR              164..165
     AlphaRep3       174..182
                     /label="AWAlpha"
     KR              186..187
                     /label="RR"
     KR              212..213
ORIGIN
        1 MKFTNAIALA ILAATAAAVA VPEPWCGRPG QLCKRTPLAE AEAEAWCGRP
GRPCRKTKRA
       61 AEALAEAFAE PAAEAYVDEA VIKRCNMVGG ACFEAKRLAR DLAEATAETK
RDADAFLEGL
      121 NIESREVSEI VAREAEAWCG RPGQPCKRNA EAECGRPGQS CKEKRKADPE
AEAWCGSPGQ
      181 PCRAVRRAAE AMAEALAEPT AEAWCGPCRK AKRDAFALAY AANVALAQL*
//
LOCUS       C_homoeocarpa_ppg1       316 aa            linear   UNA
FEATURES             Location/Qualifiers
     AlphaRep        25..33
                     /label="AWAlpha"
     KR              34..35
     AlphaRep        42..50
                     /label="AWAlpha"
     KR              51..52
     AlphaRep        59..67
                     /label="AWAlpha"
     KR              68..69
     AlphaRep        80..88
                     /label="AWAlpha"
     KR              92..93
                     /label="RR"
     KR              119..120
     KR              132..133
     KR              146..147
                     /label="RR"
     AlphaRep        174..182
                     /label="AWAlpha"
     KR              183..184
     AlphaRep        193..201
                     /label="AWAlpha"
     KR              202..203
     AlphaRep        212..220
                     /label="AWAlpha"
     KR              223..224
     AlphaRep        233..241
                     /label="AWAlpha"
     KR              245..246
```

```
                     /label="RR"
     AlphaRep        263..271
                     /label="AWAlpha"
     KR              272..273
     AlphaRep        286..294
                     /label="AWAlpha"
     KR              298..299
ORIGIN
        1 MKLTNAIALA ILAATAAAVA IPEPWCGRPG QPCKRDALPE PWCGRPGQPC
KRDALPEAWC
       61 GRPGQPCKRS ALAEPDAEAW CGRPGQPCRK VRRAAEALAK AMAEPVADVE
ADVDEAVVKR
      121 CNMIGGPCYE AKRLARDLAG VTASVRRDAD AFYEDLGIES REVSDKITRD
AEAWCGRPGQ
      181 PCKRDAAPEA EPWCGRPGQP CKRDAAPEAE PWCGRPGQPC KEKREADPEA
EAWCGRPGQP
      241 CRKVRRAAEA MAEALAEPTA EAWCGRPGQP CKREALAEAE ANAEAWCGRP
GQPCRKAKRD
      301 AFALAYAADV ALQQL*
//
LOCUS       C_jacksonii_ppg1_trunc       342 aa            linear
UNA
FEATURES             Location/Qualifiers
     AlphaRep        25..33
                     /label="AWAlpha"
     KR              34..35
     AlphaRep        42..50
                     /label="AWAlpha"
     KR              51..52
     AlphaRep        59..67
                     /label="AWAlpha"
     KR              68..69
     AlphaRep        80..88
                     /label="AWAlpha"
     KR              92..93
                     /label="RR"
     KR              119..120
     KR              132..133
     KR              146..147
                     /label="RR"
     AlphaRep        238..246
                     /label="AWAlpha"
     KR              249..250
     AlphaRep        259..267
                     /label="AWAlpha"
     KR              271..272
                     /label="RR"
     AlphaRep        289..297
                     /label="AWAlpha"
     KR              298..299
     AlphaRep        312..320
                     /label="AWAlpha"
     KR              324..325
ORIGIN
        1 MKLTNAIALA ILAATAAAVA IPEPWCGRPG QPCKRDALPE PWCGRPGQPC
KRDALPEAWC
```

```
       61 GRPGQPCKRS ALAEPDAEAW CGRPGQPCRK VRRAAEALAK AMAEPVADVE
ADVDEAVVKR
      121 CNMIGGPCYE AKRLARDLAG VTASVRRDAD AFYEDLGIES REVSDKIARD
AEAWXXXXXX
      181 XXXXXXXXXX XXXXXXXXXX XXXXXXXXXX XXXXXXXXXX XXXXXXXXXD
AAPEAEPWCG
      241 RPGQPCKEKR EADPEAEAWC GRPGQPCRKV RRAAEAMAEA LAEPTAEAWC
GRPGQPCKRE
      301 ALAEAEANAE AWCGRPGQPC RKAKRDAFAL AYAADVALRQ L*
//
LOCUS       C_monteithiana_ppg1       301 aa            linear   UNA
FEATURES             Location/Qualifiers
     AlphaRep        25..33
                     /label="AWAlpha"
     KR              34..35
     AlphaRep        42..50
                     /label="AWAlpha"
     KR              51..52
     AlphaRep        59..67
                     /label="AWAlpha"
     KR              68..69
     AlphaRep        80..88
                     /label="AWAlpha"
     KR              92..93
                     /label="RR"
     KR              119..120
     KR              132..133
     KR              146..147
                     /label="RR"
     AlphaRep        174..182
                     /label="AWAlpha"
     KR              183..184
     AlphaRep        197..205
                     /label="AWAlpha"
     KR              208..209
     AlphaRep        218..226
                     /label="AWAlpha"
     KR              230..231
                     /label="RR"
     AlphaRep        248..256
                     /label="AWAlpha"
     KR              257..258
     AlphaRep        271..279
                     /label="AWAlpha"
     KR              283..284
ORIGIN
        1 MKLTNAIALA ILAATAAAVA IPEPWCGRPG QPCKRDALPE PWCGRPGQPC
KREALPEAWC
       61 GRPGQPCKRS ALAEPDAEAW CGRPGQPCRK VRRAAEALAE AMAEPVADVE
ADVDEAVVKR
      121 CNMIGGPCYE AKRLARDLAG VTASVRRDAD AFYEDLGIES RDVSDKIARD
AEAWCGRPGQ
      181 PCKRDAAPEA APEAEPWCGR PGQPCKEKRE ADPEAEAWCG RPGQPCRKVR
RAAEAMAEAL
      241 AEPTAEAWCG RPGQPCKREA LAEAEANAEA WCGRPGQPCR KAKRDAFALA
YAADVALRQL
```

```
      301 *
//
LOCUS       C_spCPB17_ppg1       314 aa            linear   UNA
FEATURES             Location/Qualifiers
     AlphaRep        25..33
                     /label="AWAlpha"
     KR              34..35
     AlphaRep        42..50
                     /label="AWAlpha"
     KR              51..52
     AlphaRep        59..67
                     /label="AWAlpha"
     KR              68..69
     AlphaRep        80..88
                     /label="AWAlpha"
     KR              92..93
                     /label="RR"
     KR              119..120
     KR              132..133
     KR              146..147
     AlphaRep        174..182
                     /label="AWAlpha"
     KR              183..184
     AlphaRep        195..203
                     /label="AWAlpha"
     KR              204..205
     AlphaRep        210..218
                     /label="AWAlpha"
     KR              221..222
     AlphaRep        231..239
                     /label="AWAlpha"
     KR              243..244
                     /label="RR"
     AlphaRep        261..269
                     /label="AWAlpha"
     KR              270..271
     AlphaRep        284..292
                     /label="AWAlpha"
     KR              296..297
ORIGIN
        1 MKFTNAIALA ILAATATAVA IPEPWCGRPG QPCKRDALPE PWCGRPGQPC
KREALPEAWC
       61 GRPGQPCKRS ALAEPDAEAW CGRPGQPCRK VRRAAEALAE AMAEPAAEAE
ADVDEAVVKR
      121 CNIVGGACYE AKRLARDLAG VTASTKRDAD AFYEELGIES REVSDKVARD
AEAWCGRPGQ
      181 PCKRDAAPEA EAEAWCGRPG QPCKRDAEPW CGRPGQPCKE KREADPEAEA
WCGRPGQPCR
      241 QVRRAAEAMA EALAEPTAEA WCGRPGQPCK REALAEAEAN AEAWCGRPGQ
PCRKAKRDAF
      301 ALAYAADVAL AQL*
//
LOCUS       C_spSE16F4_ppg1       316 aa            linear   UNA
FEATURES             Location/Qualifiers
     AlphaRep        25..33
                     /label="AWAlpha"
```

```
     KR              34..35
     AlphaRep        42..50
                     /label="AWAlpha"
     KR              51..52
     AlphaRep        59..67
                     /label="AWAlpha"
     KR              68..69
     AlphaRep        80..88
                     /label="AWAlpha"
     KR              92..93
                     /label="RR"
     KR              119..120
     KR              132..133
     KR              146..147
                     /label="RR"
     AlphaRep        174..182
                     /label="AWAlpha"
     KR              183..184
     AlphaRep        193..201
                     /label="AWAlpha"
     KR              202..203
     AlphaRep        212..220
                     /label="AWAlpha"
     KR              223..224
     AlphaRep        233..241
                     /label="AWAlpha"
     KR              245..246
                     /label="RR"
     AlphaRep        263..271
                     /label="AWAlpha"
     KR              272..273
     AlphaRep        286..294
                     /label="AWAlpha"
     KR              298..299
ORIGIN
        1 MKLTNAIALA ILAATAAAVA IPEPWCGRPG QPCKRDALPE PWCGRPGQPC
KRDALPEAWC
       61 GRPGQPCKRS ALAEPDAEAW CGRPGQPCRK VRRAAEALAK AMAEPVADVE
ADVDEAVVKR
      121 CNMIGGPCYE AKRLARDLAG VTASVRRDAD AFYEDLGIES REVSDKITRD
AEAWCGRPGQ
      181 PCKRDAAPEA EPWCGRPGQP CKRDAAPEAE PWCGRPGQPC KEKREADPEA
EAWCGRPGQP
      241 CRKVRRAAEA MAEALAEPTA EAWCGRPGQP CKREALAEAE ANAEAWCGRP
GQPCRKAKRD
      301 AFALAYAADV ALQQL*
//
LOCUS       R_spNJR-2017aBBW_ppg1       312 aa            linear
UNA
FEATURES             Location/Qualifiers
     AlphaRep        25..33
                     /label="AWAlpha"
     KR              34..35
     AlphaRep        42..50
                     /label="AWAlpha"
     KR              51..52
```

```
     AlphaRep        59..67
                     /label="AWAlpha"
     KR              68..69
     AlphaRep        80..88
                     /label="AWAlpha"
     KR              92..93
                     /label="RR"
     KR              119..120
     KR              132..133
     KR              146..147
     AlphaRep        174..182
                     /label="AWAlpha"
     KR              183..184
     AlphaRep        191..199
                     /label="AWAlpha"
     KR              200..201
     AlphaRep        208..216
                     /label="AWAlpha"
     KR              219..220
     AlphaRep        229..237
                     /label="AWAlpha"
     KR              241..242
                     /label="RR"
     AlphaRep        259..267
                     /label="AWAlpha"
     KR              268..269
     AlphaRep        282..290
                     /label="AWAlpha"
     KR              294..295
ORIGIN
        1 MKFTSTLALA ILAATATAIA LPEPWCGRPG QPCKRDALPE PWCGRPGQPC
KRESLPEAWC
       61 GRPGQPCKRS PLAEADAEAW CGRPGQPCRK VRRAAEALAE ALAEPVADAE
ADFDEAVVKR
      121 CNMVGGACYE AKRLARDLAG VTASAKRDAD AFYEGLGIES REVSDKIARD
AEAWCGRPGQ
      181 PCKRSADAEP WCGRPGQPCK RDASPEPWCG RPGQPCKEKR EADPEAEAWC
GRPGQPCRKV
      241 RRAAEAMAEA LAEPTAEAWC GRPGQPCKRE ALAEAEANAE AWCGRPGQPC
RKAKRDAFAL
      301 AYAADVALQQ L*
//
LOCUS       R_spNJR2017aBVV2_ppg1       316 aa            linear
UNA
FEATURES             Location/Qualifiers
     AlphaRep        25..33
                     /label="AWAlpha"
     KR              34..35
     AlphaRep        42..50
                     /label="AWAlpha"
     KR              51..52
     AlphaRep        59..67
                     /label="AWAlpha"
     KR              68..69
     AlphaRep        80..88
                     /label="AWAlpha"
```

```
     KR              92..93
                     /label="RR"
     KR              119..120
     KR              132..133
     KR              146..147
                     /label="RR"
     AlphaRep        174..182
                     /label="AWAlpha"
     KR              183..184
     AlphaRep        197..205
                     /label="AWAlpha"
     KR              206..207
     AlphaRep        212..220
                     /label="AWAlpha"
     KR              223..224
     AlphaRep        233..241
                     /label="AWAlpha"
     KR              245..246
                     /label="RR"
     AlphaRep        263..271
                     /label="AWAlpha"
     KR              272..273
     AlphaRep        286..294
                     /label="AWAlpha"
     KR              298..299
ORIGIN
        1 MKFTNAIALA ILAATATAVA IPEPWCGRPG QPCKREALPE PWCGRPGQPC
KREALPEAWC
       61 GRPGQPCKRS PLAEPDAEAW CGRPGQPCRK VRRAAEALAE AMAEPAADAE
ADFDEAVVKR
      121 CNMVGGACYE AKRLARDLAG VTASVRRDAD AFYEELGIES REVSDKIARD
AEAWCGRPGQ
      181 PCKREANPEA EAEAEAWCGR PGQPCKRDAE PWCGRPGQPC KEKREADAEA
EAWCGRPGQP
      241 CRKVRRAAEA MAEALAEPTA EAWCGRPGQP CKREALAEAE ANAEAWCGRP
GQPCRKAKRD
      301 AFALAYAADV ALQQL*
//
LOCUS       R_spNJR2017aWRK4_ppg1       297 aa            linear
UNA
FEATURES             Location/Qualifiers
     AlphaRep        25..33
                     /label="AWAlpha"
     KR              34..35
     AlphaRep        42..50
                     /label="AWAlpha"
     KR              51..52
     AlphaRep        59..67
                     /label="AWAlpha"
     KR              68..69
     AlphaRep        80..88
                     /label="AWAlpha"
     KR              92..93
                     /label="RR"
     KR              119..120
     KR              132..133
```

```
     KR              146..147
     AlphaRep        174..182
                     /label="AWAlpha"
     KR              183..184
     AlphaRep        193..201
                     /label="AWAlpha"
     KR              204..205
     AlphaRep        214..222
                     /label="AWAlpha"
     KR              226..227
                     /label="RR"
     AlphaRep        244..252
                     /label="AWAlpha"
     KR              253..254
     AlphaRep        267..275
                     /label="AWAlpha"
     KR              279..280
ORIGIN
        1 MKFTSTLALA ILAATATAIA LPEPWCGRPG QPCKRDALPE PWCGRPGQPC
KREALPEAWC
       61 GRPGQPCKRS PLAEADAEAW CGRPGQPCRK VRRAAEALAE AMAEPVADAE
ADFDEAVVKR
      121 CNMVGGACYE AKRLARDLAG VTASAKRDAD AFYEGLGIES REVSDKIARD
AEAWCGRPGQ
      181 PCKRTASADA EPWCGRPGQP CKEKREADPE AEAWCGRPGQ PCRKVRRAAE
AMAEALAEPT
      241 AEAWCGRPGQ PCKREALAEA EANAEAWCGR PGQPCRKAKR DAFALAYAAD
VALQQL*
//
LOCUS       C_malorum_ppg1       203 aa            linear   UNA
FEATURES             Location/Qualifiers
     AlphaRep        29..38
                     /label="AWAlpha"
     KR              41..42
                     /label="RR"
     AlphaRep        49..58
                     /label="AWAlpha"
     KR              59..60
     AlphaRep        71..80
                     /label="AWAlpha"
     KR              81..82
     AlphaRep        93..102
                     /label="AWAlpha"
     KR              103..104
     AlphaRep        117..126
                     /label="AWAlpha"
     KR              127..128
     AlphaRep        141..150
                     /label="AWAlpha"
     KR              151..152
     AlphaRep        165..174
                     /label="AWAlpha"
     KR              175..176
     AlphaRep        187..196
                     /label="AWAlpha"
     KR              197..198
```

```
ORIGIN
        1 MKLTSAICAL ALAATVSSSA IPWAAAEARC GHPGQPCGKV RRVALPEPRC
GHPGQPCGKR
       61 DAMPEPAPEP RCGHPGQPCG KRDAMPAPEP EARCGHPGQP CGKRDAIPEP
APEPEARCGH
      121 PGQPCGKRDA IPEPAPEPEP RCGHPGQPCG KRNAIPEPAP EPEARCGHPG
QPCGKRDAMP
      181 EPEPEARCGH PGQPCGKREF VA*
//
LOCUS       L_spMPISDFRAT0119_ppg1       132 aa            linear
UNA
FEATURES             Location/Qualifiers
     AlphaRep        29..38
                     /label="AWAlpha"
     KR              41..42
     AlphaRep1       51..60
                     /label="AWAlpha"
     KR              61..62
     AlphaRep2       75..84
                     /label="AWAlpha"
     KR              85..86
     AlphaRep1       97..106
                     /label="AWAlpha"
     KR              107..108
     AlphaRep1       119..128
                     /label="AWAlpha"
ORIGIN
        1 MKLTSAICAL VLAATVSSSA IPWAAAEAKC GHPGQPCGKS KRVALPEPEA
ICGHPGQPCG
       61 KRDAIPEPAP EPEARCGHPG QPCGKRDAMP EPEPEAICGH PGQPCGKRDA
MPEPEPEAIC
      121 GHPGQPCGKK M*
//
LOCUS       N_avenae_ppg1       347 aa            linear   UNA
FEATURES             Location/Qualifiers
     AlphaRep        29..38
                     /label="AWAlpha"
     KR              41..42
     AlphaRep2       51..60
                     /label="AWAlpha"
     AlphaRep        74..83
                     /label="AWAlpha"
     KR              84..85
     AlphaRep        94..103
                     /label="AWAlpha"
     KR              104..105
     AlphaRep        114..123
                     /label="AWAlpha"
     KR              124..125
     AlphaRep        134..143
                     /label="AWAlpha"
     KR              144..145
     AlphaRep        154..163
                     /label="AWAlpha"
     KR              168..169
     KR              214..215
```

```
     KR              229..230
     AlphaRep        237..246
                     /label="AWAlpha"
     KR              247..248
     AlphaRep        259..268
                     /label="AWAlpha"
     KR              269..270
     AlphaRep1       281..290
                     /label="AWAlpha"
     KR              291..292
     KR              308..309
ORIGIN
        1 MKLTSAICAL IFAATVSSSA IPLAAAEARC GHPGQPCGKF KRAALAEPAA
RCGFPGQPCG
       61 RDAIPAPAPE PEARCGHPGQ PCGKRDAMPE PEARCGHPGQ PCGKRDAVPE
PEARCGHPGQ
      121 PCGKRDAIPE PEVRCGHPGQ PCGKRDAIPE PEARCGHPGQ PCGRDLGKRT
PIPRCGQGPY
      181 CPGSYEWEVY MVRAKNHVSW AEARAIVDKY WRKKRDANAE VIVAKTFGKR
EAIPEARCGH
      241 PGQPCGKRDA IPEPAPEARC GHPGQPCGKR DAIPEPAPEA RCGHLGQPCG
KRDAIPEPAP
      301 EARQPYGKRN ALPEPIPEPK LGVVVGLVAS ATSVTPPPNR SIDTQA*
//
LOCUS       R_spMPIPUGEAT0058_ppg1       180 aa            linear
UNA
FEATURES             Location/Qualifiers
     AlphaRep        29..38
                     /label="AWAlpha"
     KR              41..42
     AlphaRep        51..60
                     /label="AWAlpha"
     KR              61..62
     AlphaRep        73..82
                     /label="AWAlpha"
     KR              83..84
     AlphaRep        97..106
                     /label="AWAlpha"
     KR              107..108
     AlphaRep        121..130
                     /label="AWAlpha"
     KR              131..132
     AlphaRep        145..154
                     /label="AWAlpha"
     KR              155..156
     AlphaRep        167..176
                     /label="AWAlpha"
ORIGIN
        1 MKLTSAICAL IFAATVSSSA IPWAAAEARC GHPGQPCGKF KRVALPEPEA
RCGHPGQPCG
       61 KRDAIPEPAP EARCGHPGQP CGKRDAIPEP APEPEARCGH PGQPCGKRDA
IPEPAANPEA
      121 RCGHPGQPCG KRDAMPEPEP EPEARCGHPG QPCGKRDAMP EPEPEARCGH
PGQPCGKKL*
//
LOCUS       R_commune_ppg1       201 aa            linear   UNA
```

```
FEATURES             Location/Qualifiers
     AlphaRep        29..38
                     /label="AWAlpha"
     KR              41..42
     AlphaRep        51..60
                     /label="AWAlpha"
     AlphaRep        74..83
                     /label="AWAlpha"
     KR              84..85
     AlphaRep        94..103
                     /label="AWAlpha"
     KR              104..105
     AlphaRep        118..127
                     /label="AWAlpha"
     KR              128..129
     AlphaRep        138..147
                     /label="AWAlpha"
     KR              148..149
     AlphaRep        160..169
                     /label="AWAlpha"
     KR              170..171
     AlphaRep        182..191
                     /label="AWAlpha"
     KR              192..193
ORIGIN
        1 MKFTSAICAL IFAATVSSSA IPWAAAEARC GHPGQPCGKF KRVALPEPVA
RCGHPGQPCG
       61 RDAIPAPAPE PEARCGHPGQ PCGKRDAIPE PEARCGHPGQ PCGKRDAIPA
PAPEPEARCG
      121 HPGQPCGKRD AIPEPEARCG HPGQPCGKRD AIPEPAPEAR CGHPGQPCGK
RDAIPEPAPE
      181 ARCGHPGQPC GKRDAIPEAN *
//
LOCUS       B_jaapii_ppg1       207 aa            linear   UNA
FEATURES             Location/Qualifiers
     AlphaRep        29..38
                     /label="AWAlpha"
     KR              41..42
                     /label="RR"
     AlphaRep        51..60
                     /label="AWAlpha"
     KR              63..64
     AlphaRep        73..82
                     /label="AWAlpha"
     KR              85..86
     AlphaRep        99..108
                     /label="AWAlpha"
     KR              111..112
     AlphaRep        121..130
                     /label="AWAlpha"
     KR              133..134
     AlphaRep        145..154
                     /label="AWAlpha"
     KR              157..158
     AlphaRep        169..178
                     /label="AWAlpha"
```

```
     KR              181..182
     AlphaRep        191..200
                     /label="AWAlpha"
     KR              203..204
ORIGIN
        1 MKLTSAICAL ILAASVSASA LPVANAEARC GYPGQPCGKV RREAIAEPEA
RCGYPGQPCG
       61 KAKREAAPVA EARCGYPGQP CGKAKREAAP VAEAAPEARC GYPGQPCGKA
KREALPEPEA
      121 RCGYPGQPCG KAKREAVAEA EPEARCGYPG QPCGKAKREA EALPEPEARC
GYPGQPCGKA
      181 KREALPEPEA RCGYPGQPCG KAKRDE*
//
LOCUS       D_brunnea_ppg1       113 aa            linear   UNA
FEATURES             Location/Qualifiers
     AlphaRep        29..38
                     /label="AWAlpha"
     KR              41..42
     AlphaRep        49..58
                     /label="AWAlpha"
     KR              61..62
     AlphaRep        77..86
                     /label="AWAlpha"
     KR              89..90
     AlphaRep        101..110
                     /label="AWAlpha"
ORIGIN
        1 MKLTSAICAL IFAATVSASA LPVANAEARC GYRGQPCPKA KREAIAEARC
GYRGQPCPKA
       61 KREAEALPEA LPEAEARCGY RGQPCPKAKR EAEALPEPEA RCGYRGQPCP KK*
//
LOCUS       D_rosae_ppg1       280 aa            linear   UNA
FEATURES             Location/Qualifiers
     AlphaRep        29..38
                     /label="AWAlpha"
     KR              41..42
     AlphaRep        51..60
                     /label="AWAlpha"
     KR              63..64
     AlphaRep        69..78
                     /label="AWAlpha"
     KR              81..82
     KR              120..121
     KR              156..157
                     /label="RR"
     AlphaRep        162..171
                     /label="AWAlpha"
     KR              174..175
     AlphaRep        180..189
                     /label="AWAlpha"
     KR              192..193
     AlphaRep        198..207
                     /label="AWAlpha"
     KR              210..211
     AlphaRep        220..229
                     /label="AWAlpha"
```

```
     KR              232..233
                     /label="RR"
     AlphaRep        250..259
                     /label="AWAlpha"
     KR              262..263
ORIGIN
        1 MKLTSAICAL VFAASVTASA LPAAAAEARC GHPGQPCGKA KRVALPEPEA
RCGHPGQPCG
       61 KAKREAAPRC GHPGQPCGKV KRAAEAFAEA LAEPEAEASA DRTTLDARCN
LVGGACYEAK
      121 RLTRDLAAIV ASTQDDADDY YDSLEIEDED TSEKVRRDAE ARCGHPGQPC
GKAKREPEAR
      181 CGHPGQPCGK AKREAEARCG HPGQPCGKAK READPEAEAR CGHPGQPCGK
LRRAAEAIAE
      241 AVAEAEPEAR CGHPGQPCGK AKRDAHALAH ASDVALSQL*
//
LOCUS       M_coronariae_ppg1       265 aa            linear   UNA
FEATURES             Location/Qualifiers
     AlphaRep        29..38
                     /label="AWAlpha"
     KR              41..42
     AlphaRep        51..60
                     /label="AWAlpha"
     KR              63..64
     KR              123..124
     KR              159..160
                     /label="RR"
     AlphaRep        165..174
                     /label="AWAlpha"
     KR              177..178
     AlphaRep        183..192
                     /label="AWAlpha"
     KR              195..196
     AlphaRep2       205..214
                     /label="AWAlpha"
     KR              217..218
                     /label="RR"
     AlphaRep        235..244
                     /label="AWAlpha"
     KR              247..248
ORIGIN
        1 MKLTSAICAL IFAASVTASA LPVAAAEARC GKPGQPCGKA KRVALPEPEA
RCGKPGQPCG
       61 KVKRAAEAFA EALAAPDVSF LSSPASGLSW PATKNSFQAE DPAEWATLDA
RCNLVGGACY
      121 EAKRLTRDLA AIVASTQDDA DEYYDSLEIE DEDTSEKVRR NAEARCGKPG
QPCGKVKREA
      181 EARCGKPGQP CGKAKREADP EAEARCGRPG QPCGKLRRAA EAIAEAVAEP
EPEARCGKPG
      241 QPCGKAKRDA HALAHAADVA ISQL*
//
LOCUS       M_rosae_ppg1       301 aa            linear   UNA
FEATURES             Location/Qualifiers
     AlphaRep        29..38
                     /label="AWAlpha"
     KR              41..42
```

```
     AlphaRep        51..60
                     /label="AWAlpha"
     KR              63..64
     AlphaRep        69..78
                     /label="AWAlpha"
     KR              81..82
     KR              141..142
     KR              177..178
                     /label="RR"
     AlphaRep        183..192
                     /label="AWAlpha"
     KR              195..196
     AlphaRep        201..210
                     /label="AWAlpha"
     KR              213..214
     AlphaRep        219..228
                     /label="AWAlpha"
     KR              231..232
     AlphaRep        241..250
                     /label="AWAlpha"
     KR              253..254
                     /label="RR"
     AlphaRep        271..280
                     /label="AWAlpha"
     KR              283..284
ORIGIN
        1 MKLTSAICAL IFAASVTASA LPAAAAEARC GHPGQPCGKA KRVALPEPEA
RCGHPGQPCG
       61 KAKREAAPRC GHPGQPCGKV KRAAEAFAEA LAEPEVSSIS LPPVTSSCSN
THDAIQAEAS
      121 ADWTTLDARC NLVGGACYEA KRLTRDLAAI VASTQDDADD YYDSLDIEDE
DTSEKVRRDA
      181 EARCGHPGQP CGKAKREPEA RCGHPGQPCG KAKREAEARC GHPGQPCGKA
KREADPEAEA
      241 RCGHPGQPCG KLRRAAEAIA EAVAEAEPEA RCGHPGQPCG KAKRDAHALA
HASDVALSQL
      301 *
//
LOCUS       A_macrosclerotiorum_ppg1       289 aa            linear
UNA
FEATURES             Location/Qualifiers
     AlphaRep1       27..36
                     /label="AWAlpha"
     AlphaRep        51..60
                     /label="AWAlpha"
     KR              63..64
     AlphaRep        75..84
                     /label="AWAlpha"
     KR              87..88
     AlphaRep1       93..102
                     /label="AWAlpha"
     KR              105..106
     KR              141..142
     KR              177..178
     AlphaRep        183..192
                     /label="AWAlpha"
```

```
     KR              195..196
     AlphaRep3       207..216
                     /label="AWAlpha"
     KR              219..220
     AlphaRep1       229..238
                     /label="AWAlpha"
     KR              241..242
     AlphaRep1       259..268
                     /label="AWAlpha"
     KR              271..272
ORIGIN
        1 MKFTAAIAAL ILAATVTSSA LPSADARCGY PGQPCSKMIK ARLAEPEANA
RCGYPGQPCG
       61 KAKRVALPEP EADARCGYPG QPCGKAKREA DARCGYPGQP CSKVKRAAEA
FAEALAAPEA
      121 SPDNDHGLAA RCDMGSCFEA KRLTRDLASI VADTTEDPAA FYHSLGLETE
EVSENKKREA
      181 EARCGYPGQP CGKVKRDAMP EPEADVRCGY PGQPCNKAKR EADPEADARC
GYPGQPCSKL
      241 KRAAEAVAEA IAEAEPSARC GYPGQPCSKA KRDAFALAHA ADLVLGNL*
//
LOCUS       M_scopiformis_ppg1       269 aa            linear   UNA
FEATURES             Location/Qualifiers
     AlphaRep        27..36
                     /label="AWAlpha"
     AlphaRep        50..59
                     /label="AWAlpha"
     KR              62..63
     AlphaRep        72..81
                     /label="AWAlpha"
     KR              84..85
     KR              121..122
     KR              157..158
     AlphaRep        163..172
                     /label="AWAlpha"
     KR              175..176
     AlphaRep        187..196
                     /label="AWAlpha"
     KR              199..200
     AlphaRep        209..218
                     /label="AWAlpha"
     KR              221..222
     AlphaRep        239..248
                     /label="AWAlpha"
     KR              251..252
ORIGIN
        1 MKFTAAIAAL ILAATVTATA LPNADARCGY PGQPCGKVKA RLAEPEADAR
CGYPGQPCGK
       61 AKRDAMPDAD ARCGYPGQPC GKVKRAAEAF AEALVPKPGH ILPRDLHLTA
RCDMGSCYEA
      121 KRMTRDLAAI VAETTEDPAA YYVSLGLEDE ETSEKLKREA SARCGYPGQP
CGKVKRDAAP
      181 EPEADARCGY PGQPCGKAKR EADPEADARC GYPGQPCGKL KRAAEAVAAA
IAEAEPAARC
      241 GYPGQPCGKA KRDAFALAHA VDLVLGNL*
//
```

```
LOCUS       P_spD728_ppg1       267 aa            linear   UNA
FEATURES             Location/Qualifiers
     AlphaRep        27..36
                     /label="AWAlpha"
     AlphaRep        50..59
                     /label="AWAlpha"
     KR              62..63
     AlphaRep        68..77
                     /label="AWAlpha"
     KR              80..81
     KR              119..120
     KR              155..156
     AlphaRep        161..170
                     /label="AWAlpha"
     KR              173..174
     AlphaRep        185..194
                     /label="AWAlpha"
     KR              197..198
     AlphaRep        207..216
                     /label="AWAlpha"
     KR              219..220
     AlphaRep        237..246
                     /label="AWAlpha"
     KR              249..250
ORIGIN
        1 MKLTAAIAAL ILAATVSASA LPNPDARCGY PGQPCGKVKA RLAEPEADAR
CGYPGQPCGK
       61 AKREADARCG YPGQPCGKVK RAAEAFAAAL AEPSNDKLST RDNDALAARC
DMGSCYEAKR
      121 MTRDLAAIVA ETTDDPAAFY HSLNLEDEET SEKLKREAEA RCGYPGQPCG
KVKRDAAPEP
      181 EADARCGYPG QPCGKAKREA DPEADARCGY PGQPCGKLKR AAEAVAEAIA
EAEPSARCGY
      241 PGQPCGKAKR DAFAMAHAAD IVLGNL*
//
LOCUS       P_subalpina_ppg1       265 aa            linear   UNA
FEATURES             Location/Qualifiers
     AlphaRep5       27..36
                     /label="AWAlpha"
     AlphaRep        51..60
                     /label="AWAlpha"
     KR              63..64
     AlphaRep1       69..78
                     /label="AWAlpha"
     KR              81..82
     KR              117..118
     KR              153..154
     AlphaRep        159..168
                     /label="AWAlpha"
     KR              171..172
     AlphaRep3       183..192
                     /label="AWAlpha"
     KR              195..196
     AlphaRep4       205..214
                     /label="AWAlpha"
     KR              217..218
```

```
     AlphaRep1       235..244
                     /label="AWAlpha"
     KR              247..248
ORIGIN
        1 MKFTAAIAAL ILAATVTSSA LPSADARCGY PGQPCTKMVK ARLAEPEANA
RCGYPGQPCG
       61 KAKREADARC GYPGQPCSKV KRAAEAFAEA LASPEASADN DHGLAARCDM
GSCFEAKRMT
      121 RDLAAIVADT TEDPAAFYHS LGLETEEVSE NKKREAEARC GYPGQPCGKV
KRDAAPEPED
      181 DARCGYPGQP CNKAKREADP EADARCGYRG QPCSKLKRAA EAVAEAIAEA
EPTARCGYPG
      241 QPCSKAKRDA FALAHAADLV LGNL*
//
LOCUS       E_pisi_ppg1?        64 aa            linear   UNA
FEATURES             Location/Qualifiers
ORIGIN
        1 MKITIFLAAL AFFTTSLGAS IPNKIEDRAM LHDTTIKPSL IEARDPRNGR
QNCGYVGQGC
       61 PGG*
//
LOCUS       E_pulchra_potential_ppg1        63 aa            linear
UNA
FEATURES             Location/Qualifiers
ORIGIN
        1 MKLTSFLAAL AFFTTSLCAS IPIKIDGVML DDTTIQSSLI EARDPRKGRE
NCGYVGQACP
       61 GG*
//
LOCUS       G_cichoracearum_ppg1?        65 aa            linear
UNA
FEATURES             Location/Qualifiers
ORIGIN
        1 MKSTGLLFAL ALLTALLCSS LPSVAHDLLH LEASSSAKTS PKIIRDPRKG
RQTCGRPGQA
       61 CSGG*
//
LOCUS       G_magnicellulatus_ppg1?        65 aa            linear
UNA
FEATURES             Location/Qualifiers
ORIGIN
        1 MKSTSLLFAL ALLTAPLCSS LPSTAHDLFH LEASSSGKPS PIIVRDPRKG
RQTCGRPGQA
       61 CSGG*
//
LOCUS       O_heveae_ppg1?        64 aa            linear   UNA
FEATURES             Location/Qualifiers
ORIGIN
        1 MKITIFLAAV TFFTTSLSAS IPIKIEDRVV PDDTTIKSPL IEARDPRNGR
QNCGYVGQGC
       61 PGG*
//
LOCUS       O_neolycopersici_ppg1?        63 aa            linear
UNA
FEATURES             Location/Qualifiers
ORIGIN
```

```
        1 MKLTNFLAAL TFFTTSFCAS IPIQIDGVML DDTTIKSSLI ETRDPRKGRQ
NCGYVGQACP
       61 GG*
//
LOCUS       P_cerasi_ppg1?        63 aa            linear   UNA
FEATURES             Location/Qualifiers
ORIGIN
        1 MKLVVTLVAL FVSTVSSMVL PHASLAAIIY PREARPQPAP NPVPKPWRKD
HCGRPGQACV
       61 GG*
//
LOCUS       P_moricola_ppg1?        65 aa            linear   UNA
FEATURES             Location/Qualifiers
ORIGIN
        1 MKSIKLLAVL AVAVAVTALH INIPKIADNV TLEQPQTTPD RVQPRGPRST
KAHCGRPGQV
       61 CMGG*
//
LOCUS       P_xanthii_ppg1?        65 aa            linear   UNA
FEATURES             Location/Qualifiers
ORIGIN
        1 MKPILALPLF ILLITMVSSI ALPHTSSSST ITQREAKPQP VPKPEPRPWR
KDHCGRPGQP
       61 CPGG*
//
LOCUS       C_longipes_ppg1       239 aa            linear   UNA
FEATURES             Location/Qualifiers
     AlphaRep        35..46
                     /label="AWAlpha"
     KR              47..48
     AlphaRep1       55..66
                     /label="AWAlpha"
     KR              67..68
     AlphaRep1       75..86
                     /label="AWAlpha"
     KR              87..88
     KR              149..150
     AlphaRep1       155..166
                     /label="AWAlpha"
     KR              167..168
     AlphaRep1       179..190
                     /label="AWAlpha"
     KR              191..192
     AlphaRep1       209..220
                     /label="AWAlpha"
     KR              221..222
ORIGIN
        1 MQLTSALCAI ILAVTVSSAA LPIAEPVTAA IAEPLCGARG QPCGKAKREA
EAAPRCGARG
       61 QPCGKAKREA EAAPRCGARG QPCGKVKRAA EAFAEALAEP IPEPQEDSLY
ARHALQLTRD
      121 LASIVAETQD DPEAFFASLS LPEDSEVDKR DALARCGARG QPCGKAKREA
EADPEAEARC
      181 GARGQPCGKL RRAAEAIAEA VAYAEPEARC GARGQPCGKA KRNALAMAHA
ADIALAAL*
//
```

```
LOCUS       C_variabilis_ppg1        89 aa            linear   UNA
FEATURES             Location/Qualifiers
     AlphaRep        31..40
                     /label="AWAlpha"
     KR              41..42
                     /label="RR"
     AlphaRep        51..60
                     /label="AWAlpha"
     KR              63..64
     AlphaRep        75..84
                     /label="AWAlpha"
ORIGIN
        1 MKVTSTLFIV AMATAATAMA IPAAEPEPLP WCQWKGQGGC RRSAEPEPLP
WCQWKGQGGC
       61 RAKREAEAEA EPMPWCQWKG QGGCKEKL*
//
LOCUS       H_bicolor_ppg1       269 aa            linear   UNA
FEATURES             Location/Qualifiers
     AlphaRep        35..46
                     /label="AWAlpha"
     KR              47..48
     AlphaRep        57..68
                     /label="AWAlpha"
     KR              69..70
     AlphaRep        81..92
                     /label="AWAlpha"
     KR              93..94
     AlphaRep1       101..112
                     /label="AWAlpha"
     KR              113..114
     KR              177..178
     AlphaRep        183..194
                     /label="AWAlpha"
     KR              195..196
     AlphaRep2       209..220
                     /label="AWAlpha"
     KR              221..222
                     /label="RR"
     AlphaRep        239..250
                     /label="AWAlpha"
     KR              251..252
ORIGIN
        1 MQLTNALCVL ILAVTVSSTA LPNAQPAAAA IVEPRCGARG QPCGKAKREA
APEPAPRCGA
       61 RGQPCGKAKR EAAPEPEAAP RCGARGQPCG KAKREAEAAP RCGARGQPCG
KVKRAAEAFA
      121 EAIAEPTAEP QEDSVYARHA LQLTRDLASV VAETQDDAEA FWTTLSLPED
GESEVDKRDA
      181 LARCGARGQP CGKAKRDAEA EADPEAEARC GARGQPCGKL RRAAEAIAEA
VAVSEPEARC
      241 GARGQPCGKA KRDALALAHA ADIVLSAL*
//
LOCUS       H_hepaticicola_ppg1       211 aa            linear   UNA
FEATURES             Location/Qualifiers
     AlphaRep        38..52
                     /label="AWAlpha"
```

```
     KR              53..54
     KR              115..116
     AlphaRep1       123..136
                     /label="AWAlpha"
     KR              137..138
     AlphaRep2       145..159
                     /label="AWAlpha"
     KR              160..161
     AlphaRep3       178..192
                     /label="AWAlpha"
     KR              193..194
ORIGIN
        1 MHLTNTLCAL ILAVTVSSVA LPNAEPIAAA VANAQWDNDW GCGERGQPCG
KVKRAAEAFA
       61 EALAEPNAQP ELDSPYARYA RQLTRDLAAV VAETQDDPVK YFASLALRDD
SDLDKRDALA
      121 EAGWGWGERG QPCGKAKRDA DPAAWDWGCG ERGQPCGKLR RAAEAIAEAV
AVPESEAWDW
      181 GCGERGQPCG KAKRDALALA HAADIILRTI *
//
LOCUS       H_variabilis_ppg1_trunc       149 aa            linear
UNA
FEATURES             Location/Qualifiers
     AlphaRep        35..48
                     /label="AWAlpha"
     KR              49..50
     AlphaRep        61..74
                     /label="AWAlpha"
     AlphaRep1       80..93
                     /label="AWAlpha"
     KR              94..95
     AlphaRep3       106..119
                     /label="AWAlpha"
     KR              120..121
ORIGIN
        1 MQLTRALCAL ILAVTVSSAA LPVAYPIANA IAEPVALCGR RGEPCGKVKR
EAEAEPVAEP
       61 VALCGRRGEP CGKVKLAEPV ALCGRRGEPC GKAKREAEAE PVAEPVARCG
RRGEPCGKVK
      121 REAEAEPEAE PEAEPVARCG RRGEPCGK*
//
LOCUS       A_encephaloides_ppg1       243 aa            linear
UNA
FEATURES             Location/Qualifiers
     KR              54..55
     AlphaRep        64..72
                     /label="AWAlpha"
     KR              75..76
     KR              83..84
     AlphaRep        93..101
                     /label="AWAlpha"
     KR              106..107
     KR              118..119
     AlphaRep        128..136
                     /label="AWAlpha"
     KR              141..142
```

```
     KR              149..150
     KR              169..170
     AlphaRep        179..187
                     /label="AWAlpha"
     KR              192..193
     AlphaRep        206..214
                     /label="AWAlpha"
     KR              217..218
ORIGIN
        1 MKFANSLCAI ALLASANALP TAEDTAAAAT GPPSGGPPPG YFSLIQHLDS
NFEKRNAEAV
       61 AEACQWWGQG CWKAKREAEA VEKRASEAEA EPCQWWGQGC WKAKVKREAE
AEAEAIAKRD
      121 AEAEAEPCQW WGQGCWKTKV KREAEAITKR YAEAAAEALA EPNAEAIAKR
DANAEAEPCQ
      181 WWGQGCWKEK VKREAEAAAE AVAEACQWWG QGCWKAKRDA EAVTKSHAAA
EAEAAAAAEA
      241 KA*
//
LOCUS       H_albidus_ppg1       367 aa            linear   UNA
FEATURES             Location/Qualifiers
     KR              52..53
     AlphaRep        66..74
                     /label="AWAlpha"
     KR              77..78
     AlphaRep1       89..97
                     /label="AWAlpha"
     KR              100..101
     AlphaRep        118..126
                     /label="AWAlpha"
     KR              129..130
     AlphaRep2       141..149
                     /label="AWAlpha"
     KR              152..153
     AlphaRep        164..172
                     /label="AWAlpha"
     KR              175..176
     AlphaRep2       185..193
                     /label="AWAlpha"
     KR              196..197
     KR              232..233
     KR              273..274
     AlphaRep        277..285
                     /label="AWAlpha"
     KR              286..287
     KR              294..295
     AlphaRep2       310..318
                     /label="AWAlpha"
     KR              321..322
     KR              348..349
ORIGIN
        1 MKLTNTLCVL ILAFTASAAP NASPNPEANP FAAAVAEAEA CYSVGGACYN
AKRAATPEAL
       61 AEAEACRYAG QGCWKAKRTA EALANPEACK YAGQGCWKIK REAEAEPEPE
AHPAAEACRY
```

```
      121 AGQGCWKAKR EAAAAAEAEA CKYVGQGCWK AKREASALAE AEACRYAGQG
CWKAKREADA
      181 EAEACKYVGQ GCWKAKRAAE AFAEALAEPF AEPNEKISAR CDLVGGACYN
AKRDALAMAE
      241 AVAAAQPNPE EFLATLKLRN PDIDALEAEK KIKREPCRYA GQGCWKRDAE
AVEKRSPSPE
      301 ALAGADAEAC KYVGQGCWKV KRAAEALAEA AANPEPCLRV GGACFQAKRD
FHAFENVARS
      361 VIASQA*
//
LOCUS       H_fraxineus_ppg1       416 aa            linear   UNA
FEATURES             Location/Qualifiers
     KR              52..53
     AlphaRep        66..74
                     /label="AWAlpha"
     KR              77..78
     AlphaRep1       89..97
                     /label="AWAlpha"
     KR              100..101
     AlphaRep        118..126
                     /label="AWAlpha"
     KR              129..130
     AlphaRep2       141..149
                     /label="AWAlpha"
     KR              152..153
     AlphaRep1       164..172
                     /label="AWAlpha"
     KR              175..176
     AlphaRep2       187..195
                     /label="AWAlpha"
     KR              198..199
     AlphaRep3       206..214
                     /label="AWAlpha"
     KR              217..218
     KR              253..254
     KR              294..295
     AlphaRep        298..306
                     /label="AWAlpha"
     KR              307..308
     KR              321..322
                     /label="RR"
     KR              339..340
     AlphaRep2       359..367
                     /label="AWAlpha"
     KR              370..371
     KR              397..398
ORIGIN
        1 MKLTNTLCVL ILAFTASAAP NASPNPEANP FAAAVAEAEA CYSVGGACYN
AKRAATPEAL
       61 AEAEACRYAG QGCWKAKRTA EALANPEACK YAGQGCWKIK REAEAEPEPE
AHPAAEACRY
      121 AGQGCWKAKR EAAAAAEAEA CKYVGQGCWK AKREASALAE AEACKYAGQG
CWKAKRAAAA
      181 DAEAEACKYV GQGCWKAKRE ADAEACQYVG QGCWKAKRAA EAFAEALAEP
FAEPNEKISA
```

```
      241 RCDLVGGACY NAKRDALAMA EAVAAAQPNP EEFLATLKLR NPDIDALEAA
KKIKREPCRY
      301 AGQGCWKRDA EAVEVRDQIA RRCFKFVKGF RPDLCWKQKR SPSPEALAEA
DAKADAEACK
      361 YVGQGCWKVK RAAEALAEAA ANPEPCLRVG GACFQAKRDL HAFENVARSV
IASQA*
//
LOCUS       H_fructigenus_ppg1       328 aa            linear   UNA
FEATURES             Location/Qualifiers
     KR              49..50
     AlphaRep        63..71
                     /label="AWAlpha"
     KR              74..75
     AlphaRep        86..94
                     /label="AWAlpha"
     KR              97..98
     AlphaRep        111..119
                     /label="AWAlpha"
     KR              122..123
     AlphaRep        134..142
                     /label="AWAlpha"
     KR              145..146
     AlphaRep        153..161
                     /label="AWAlpha"
     KR              164..165
     KR              196..197
     KR              237..238
     AlphaRep        247..255
                     /label="AWAlpha"
     KR              258..259
     AlphaRep        274..282
                     /label="AWAlpha"
     KR              285..286
     AlphaRep        301..309
                     /label="AWAlpha"
     KR              312..313
ORIGIN
        1 MRLANTLCVL ILAATSVSAA PNANPDPFAA AVAEAEACYS VGGACYNAKR
AASPEALAEA
       61 EACRRVGQSC WKAKRTANAL ANPEACRRVG QSCWKVKREA DAEAEAEPEA
CRRVGQSCWK
      121 AKRAAEAEAE AEACRRVGQS CWKAKRDADA EACRRVGQSC WKVKRAAEAV
AEAFAEPTEQ
      181 LEARCDLVGG ACYNAKRDAL AIAEAVAAAS PDGEEFMSKL KLRNPDIDAE
EAENKIKREA
      241 EAEAEACRRV GQSCWKNKRD ANPEAEAEAD AEACRRVGQS CWKVKRAAEA
LADAAANPEP
      301 CRRVGQSCWK AKRDLHAFEN VARSILA*
//
LOCUS       H_herbarum_ppg1       328 aa            linear   UNA
FEATURES             Location/Qualifiers
     KR              49..50
     AlphaRep        63..71
                     /label="AWAlpha"
     KR              74..75
     AlphaRep        86..94
```

```
                     /label="AWAlpha"
     KR              97..98
     AlphaRep        111..119
                     /label="AWAlpha"
     KR              122..123
     AlphaRep        134..142
                     /label="AWAlpha"
     KR              145..146
     AlphaRep        153..161
                     /label="AWAlpha"
     KR              164..165
     KR              196..197
     KR              237..238
     AlphaRep        247..255
                     /label="AWAlpha"
     KR              258..259
     AlphaRep        274..282
                     /label="AWAlpha"
     KR              285..286
     AlphaRep        301..309
                     /label="AWAlpha"
     KR              312..313
ORIGIN
        1 MRLTNTLCVL ILAATSVSAA PNANPDPFAA AVAEAEACYS VGGACYNSKR
AASPEALAEA
       61 EACRRVGQSC WKAKRTADAL ANPEACRRVG QSCWKVKREA DAEAEAEPEA
CRRVGQSCWK
      121 AKRAAEAEAE AEACRRVGQS CWKAKRDADA EACRRVGQSC WKVKRAAEAV
AEAFAEPTEQ
      181 LEARCDLVGG ACYNAKRDAL AIAEAVAAAS PDGEEFISKL KLRNPDIDAE
EAEKKIKREA
      241 EAEAEACRRV GQSCWKNKRD ANPEAEAEAD AEACRRVGQS CWKVKRAAEA
LAEAAADPEP
      301 CRRVGQSCWK AKRDLHAFEN VARSILA*
//
LOCUS       H_infarciens_ppg1       328 aa            linear   UNA
FEATURES             Location/Qualifiers
     KR              49..50
     AlphaRep        63..71
                     /label="AWAlpha"
     KR              74..75
     AlphaRep        86..94
                     /label="AWAlpha"
     KR              97..98
     AlphaRep        111..119
                     /label="AWAlpha"
     KR              122..123
     AlphaRep        134..142
                     /label="AWAlpha"
     KR              145..146
     AlphaRep        153..161
                     /label="AWAlpha"
     KR              164..165
     KR              196..197
     KR              237..238
     AlphaRep        247..255
```

```
                     /label="AWAlpha"
     KR              258..259
     AlphaRep        274..282
                     /label="AWAlpha"
     KR              285..286
     AlphaRep        301..309
                     /label="AWAlpha"
     KR              312..313
ORIGIN
        1 MRLTNTLCVL ILAATSVSAA PNANPDPFAA AVAEAEACYS IGGACYNAKR
AASPEALAEA
       61 EACRRVGQSC WKAKRTADAL ANPEACRRVG QSCWKVKREA DAEAEAEPEA
CRRVGQSCWK
      121 AKRAAEAEAE AEACRRVGQS CWKAKRDADA EACRRVGQSC WKVKRAAEAV
AEAFAEPTEQ
      181 LEARCDLVGG ACYNAKRDAL AIAEAVAAAS PDGEEFMSKL KLRNPDIDAE
EAEKKIKREA
      241 EAEAEACRRV GQSCWKNKRD ANPEAEAEAD AEACRRVGQS CWKVKRAAEA
LAEAAANPEP
      301 CRRVGQSCWK AKRDLHAFEN VARSVLA*
//
LOCUS       H_koreanus_ppg1       376 aa            linear   UNA
FEATURES             Location/Qualifiers
     KR              52..53
     AlphaRep        66..74
                     /label="AWAlpha"
     KR              77..78
     AlphaRep1       89..97
                     /label="AWAlpha"
     AlphaRep        118..126
                     /label="AWAlpha"
     KR              129..130
     AlphaRep2       141..149
                     /label="AWAlpha"
     KR              152..153
     AlphaRep        164..172
                     /label="AWAlpha"
     KR              175..176
     AlphaRep        187..195
                     /label="AWAlpha"
     KR              198..199
     KR              237..238
     KR              278..279
     AlphaRep        282..290
                     /label="AWAlpha"
     KR              291..292
     KR              299..300
     AlphaRep3       319..327
                     /label="AWAlpha"
     KR              330..331
     KR              357..358
ORIGIN
        1 MKLTNTLCVL ILAFTASAAP NASPNPEANP FAAAVAEAEA CYSVGGACYN
AKRAATPEAL
       61 AEAEACRYAG QGCWKAKRTA EALANPEACK YAGQGCWKIN REAEAEPEPE
AHPAAEACRY
```

121 AGQGCWKAKR EAAAAAEAEA CKYVGQGCWK AKRETSALAE AEACRYAGQG
CWKAKRAAAA
181 DAEAEACRYA GQGCWKAKRE ADAEACKYVA LAEPFAEPNE KISARCDLVG
GACYNAKRDA
241 LAMAEAVAAA QPNPEEFLAT LKLRNPDIDA LEAEKKIKRE PCRYAGQGCW
KRDAEAVEKR
301 SPSPEALAEA DAKADAEACK YVGQGYWKVK RAAEALAEAA ANPEPCLRVG
GACFQAKRDL
361 HAFENVARSA IASQA*
//
LOCUS H_linearius_ppg1 354 aa linear UNA
FEATURES Location/Qualifiers
KR 48..49
AlphaRep 62..70
/label="AWAlpha"
KR 73..74
AlphaRep 85..93
/label="AWAlpha"
KR 96..97
AlphaRep 110..118
/label="AWAlpha"
KR 121..122
AlphaRep 133..141
/label="AWAlpha"
KR 144..145
AlphaRep 152..160
/label="AWAlpha"
KR 163..164
KR 199..200
KR 240..241
AlphaRep 246..254
/label="AWAlpha"
KR 257..258
KR 267..268
/label="RR"
KR 285..286
AlphaRep 297..305
/label="AWAlpha"
KR 308..309
KR 335..336
ORIGIN
1 MKLTNTLCVL ILAFTASAAP NAEPNPFAAA VAEAEACYSV GGQCYNAKRA
ATPEALAAAE
61 ACKYVGQGCW KAKRTAAALA NPEACKYVGQ GCWKVKREAE AHPEPEAEAC
KYVGQGCWKA
121 KRAAEAEAEA EACKYVGQGC WKAKREADAE ACKYVGQGCW KAKRAAEAFA
EALAEPFAEP
181 NEKISARCDL VGGACYNAKR DALAMAEAVA AAQPNPEEWL ANLKLRNPDI
DALEAEKKIK
241 REAEPCKYVG QGCWKEKRDA EVREQLRRRC RMVAGWHPDL CWKQKREATP
EADAEACKYV
301 GQGCWKVKRA AEALAEAAAN PEPCLRLGGA CFQAKRDLLA FETVARSVIA
NQA*
//
LOCUS H_occultus_ppg1 376 aa linear UNA
FEATURES Location/Qualifiers

```
     KR              52..53
     AlphaRep        66..74
                     /label="AWAlpha"
     KR              77..78
     AlphaRep1       89..97
                     /label="AWAlpha"
     AlphaRep        118..126
                     /label="AWAlpha"
     KR              129..130
     AlphaRep2       141..149
                     /label="AWAlpha"
     KR              152..153
     AlphaRep        164..172
                     /label="AWAlpha"
     KR              175..176
     AlphaRep        187..195
                     /label="AWAlpha"
     KR              198..199
     KR              237..238
     KR              278..279
     AlphaRep        282..290
                     /label="AWAlpha"
     KR              291..292
     KR              299..300
     AlphaRep3       319..327
                     /label="AWAlpha"
     KR              330..331
     KR              357..358
ORIGIN
        1 MKLTNTLCVL ILAFTASAAP NASPNPEANP FAAAVAEAEA CYSVGGACYN
AKRAATPEAL
       61 AEAEACRYAG QGCWKAKRTA EALANPEACK YAGQGCWKIN REAEAEPEPE
AHPAAEACRY
      121 AGQGCWKAKR EAAAAAEAEA CKYVGQGCWK AKRETSALAE AEACRYAGQG
CWKAKRAAAA
      181 DAEAEACRYA GQGCWKAKRE ADAEACKYVA LAEPFAEPNE KISARCDLVG
GACYNAKRDA
      241 LAMAEAVAAA QPNPEEFLAT LKLRNPDIDA LEAEKKIKRE PCRYAGQGCW
KRDAEAVEKR
      301 SPSPEALAEA DAKADAEACK YVGQGYWKVK RAAEALAEAA ANPEPCLRVG
GACFQAKRDL
      361 HAFENVARSA IASQA*
//
LOCUS       H_repandus_ppg1       128 aa            linear   UNA
FEATURES             Location/Qualifiers
     KR              36..37
     AlphaRep        46..56
                     /label="AWAlpha?"
     KR              57..58
     KR              65..66
     KR              82..83
     AlphaRep1       93..103
                     /label="AWAlpha?"
     KR              112..113
ORIGIN
```

```
        1 MMFTNALCAI ALLVSANALP TAVDTTTAAA DNNFEKRNAE SVASVCQWWG
QACLKAKREA
       61 EPVEKRTAEA EVEPSCWKYL VKREAEAAAA EACQWWGQAC WKAMRDAEAF
TKRHAEAEAN
      121 ASAEAKA*
//
LOCUS       H_scutula_ppg1       294 aa            linear   UNA
FEATURES             Location/Qualifiers
     KR              49..50
     AlphaRep        63..71
                     /label="AWAlpha"
     KR              74..75
     AlphaRep1       86..94
                     /label="AWAlpha"
     KR              97..98
     AlphaRep2       111..119
                     /label="AWAlpha"
     KR              122..123
     KR              142..143
     KR              174..175
     KR              215..216
     KR              224..225
     AlphaRep        240..248
                     /label="AWAlpha"
     KR              251..252
     KR              278..279
ORIGIN
        1 MKLTNTICTP ILAATSASAT PTANPNPFAA AVAEAEACYS IGGACHNAKR
AATPEALAEA
       61 EACRYVGQSC WKAKRTADAL ANPEACKYVG QGCWKVKRAA EAEAEPEPEA
CRYVGKSCWK
      121 AKRAAEAEAE AEACKYSCWK VKRAAKAVAE AFAEPTEQIS ARCDLVGGAC
YNAKRDALAM
      181 AEAVAAASPD GEEFLSKLKL RNPDIDAQEA ERKLKRETEA GKTKRDANPE
AEAEADAEAC
      241 RYVGQSCWKV KRAAEALAEA AANPEPCARP RAICWQAKRD LYAFENIARS
ILA*
//
LOCUS       H_dingleyae_ppg1       102 aa            linear   UNA
FEATURES             Location/Qualifiers
     AlphaRep        34..42
                     /label="AWAlpha"
     KR              45..46
     AlphaRep1       57..65
                     /label="AWAlpha"
     KR              68..69
     AlphaRep        80..88
                     /label="AWAlpha"
     KR              91..92
ORIGIN
        1 MQLTNILSFL AIAATAVSAM PAAEANPAAV AEACQYPGQG CWKLKRDAAA
SAEAEACLYP
       61 GQGCWKAKRE AVAVAEAEAC QYPGQGCWKA KREVRTLLIS S*
//
LOCUS       P_palmicola_ppg1       176 aa            linear   UNA
FEATURES             Location/Qualifiers
```

```
     AlphaRep        36..44
                     /label="AWAlpha"
     KR              47..48
     AlphaRep        59..67
                     /label="AWAlpha"
     KR              70..71
     AlphaRep        82..90
                     /label="AWAlpha"
     KR              93..94
     AlphaRep        105..113
                     /label="AWAlpha"
     KR              116..117
     AlphaRep        128..136
                     /label="AWAlpha"
     KR              139..140
     AlphaRep        149..157
                     /label="AWAlpha"
     KR              160..161
ORIGIN
        1 MKLTTALYVL VLAATTATAI PNPEPLALAL AEAEACKRPG QPCWKAKRAA
EAMAEAEACK
       61 RPGQPCWKAK RAAEAVAEAE ACKRPGQPCW KAKRAAEAEA EAEACKRPGQ
PCWKAKRAAE
      121 AMAEAEACKR PGQPCWKAKR AAEAEAEACK RPGQPCWKAK REAEAEAEAE
AEAEE*
//
LOCUS       C_acicularis_ppg1       103 aa            linear   UNA
FEATURES             Location/Qualifiers
     KR              28..29
     AlphaRep        38..49
                     /label="AWAlpha"
     KR              50..51
     AlphaRep1       60..71
                     /label="AWAlpha"
     KR              72..73
     AlphaRep2       82..93
                     /label="AWAlpha"
     KR              94..95
ORIGIN
        1 MKFTNAIAVL ILAATSVSAF PTADAADKRA ALPEADPQCY QPGEPCWKAK
RXAXAXAEAX
       61 CYHPGEPCWK AKREAEAAAE AQCYHPGEPC WKAKRDFLAK KK*
//
LOCUS       H_varia_ppg1       216 aa            linear   UNA
FEATURES             Location/Qualifiers
     KR              29..30
     KR              66..67
     AlphaRep        78..89
                     /label="AWAlpha"
     KR              90..91
     AlphaRep        100..111
                     /label="AWAlpha"
     KR              112..113
     AlphaRep        122..133
                     /label="AWAlpha"
     KR              134..135
```

```
     AlphaRep        146..157
                     /label="AWAlpha"
     KR              158..159
     AlphaRep        168..179
                     /label="AWAlpha"
     KR              180..181
     AlphaRep        190..201
                     /label="AWAlpha"
     KR              202..203
ORIGIN
        1 MQLANAFCVF ILAATSVSAY PNPAEAFDKR LALAEAGSLP SPCIVRPCKT
LAHVLAMRCF
       61 CGYKSKREPV ADPVAEACIG RPGQPCWKGK RTAVAEAEAC IGRPGQPCWK
GKREALAEAE
      121 ACIGRPGQPC WKIKREAEAE PEAEACIGRP GQPCWKGKRE AVAEADACIG
RPGQPCWKGK
      181 REALAEAEAC IGRPGQPCWK LKREACIARP GQPCP*
//
LOCUS       L_arida_ppg1       247 aa            linear   UNA
FEATURES             Location/Qualifiers
     AlphaRep        25..36
                     /label="AWAlpha"
     KR              37..38
     KR              111..112
     AlphaRep1       119..130
                     /label="AWAlpha"
     KR              131..132
     AlphaRep        139..150
                     /label="AWAlpha"
     KR              151..152
     AlphaRep1       167..178
                     /label="AWAlpha"
     KR              179..180
     AlphaRep        189..200
                     /label="AWAlpha"
     KR              201..202
     AlphaRep        217..228
                     /label="AWAlpha"
     KR              229..230
ORIGIN
        1 MQLTNALCVV ILAATTAVTA LPEPGCGARG QGCWKIKRAA NALAEALAEP
AVDVIEARCN
       61 IAGGACQKAR MLTRDLAETV AAVQDDPEAY YASLNFEDDS AVETAAIKQV
KREAEADARC
      121 GARGQGCWKE KRDAAPAPGC GARGQGCWKE KRVAISAAEA EANPEARCGA
RGQGCWKIKR
      181 EAVSEADAGC GARGQGCWKV KRAAEAIADA LAEPLPGCGA RGQGCWKEKR
DIQALQNAAR
      241 DALEKL*
//
LOCUS       L_cervina_ppg1_trunc       142 aa            linear
UNA
FEATURES             Location/Qualifiers
     AlphaRep        25..36
                     /label="AWAlpha"
     KR              37..38
```

```
     KR              111..112
     AlphaRep1       119..130
                     /label="AWAlpha"
     KR              131..132
ORIGIN
        1 MQLTNTLCVI ILAATTAVTA LPEPHCGARG QGCWKIKRAA NALAEALAEP
AVDVINARCN
       61 IAGGACQKAR MLTRDLAETV AAVQDDPEAY FASLSFEDDS AVETAATEQV
KREADADARC
      121 GARGQGCWKE KRDAAPAPGC GA
//
LOCUS       L_hyalina_ppg1       247 aa            linear   UNA
FEATURES             Location/Qualifiers
     AlphaRep        25..36
                     /label="AWAlpha"
     KR              37..38
     KR              111..112
     AlphaRep1       119..130
                     /label="AWAlpha"
     KR              131..132
     AlphaRep        139..150
                     /label="AWAlpha"
     KR              151..152
     AlphaRep1       167..178
                     /label="AWAlpha"
     KR              179..180
     AlphaRep1       189..200
                     /label="AWAlpha"
     KR              201..202
     AlphaRep1       217..228
                     /label="AWAlpha"
     KR              229..230
ORIGIN
        1 MQLTNTLCVI ILAATTAVTA LPEPGCGARG QGCWKIKRAA NALAEALAEP
AVDVIEARCN
       61 IAGGACQKAR MLTRDLAETV AAVQDDPEAY YASLSFEDDS AVETAAIKQI
KREADADARC
      121 GARGQGCWKE KRDAAPAPGC GARGQGCWKE KREAISAAEA EANPEARCGA
RGQGCWKIKR
      181 EAVSEADARC GARGQGCWKV KRAAEAIADA LAEPLPRCGA RGQGCWKQKR
DIQALQNVAR
      241 DVLEKL*
//
LOCUS       L_occidentalis_ppg1_trunc       161 aa            linear
UNA
FEATURES             Location/Qualifiers
     KR              24..25
     AlphaRep        30..42
                     /label="AWAlpha"
     KR              43..44
     AlphaRep1       53..64
                     /label="AWAlpha"
     KR              65..66
     AlphaRep1       81..92
                     /label="AWAlpha"
     KR              93..94
```

```
     AlphaRep1       103..114
                     /label="AWAlpha"
     KR              115..116
     AlphaRep3       131..142
                     /label="AWAlpha"
     KR              143..144
ORIGIN
        1 EAYYASLSFE DDSAVEAAAT EQVKREAEAH NGCGARGQGC PKKRDADADA
APRCGARGQG
       61 CWKEKRVAIS AAEAEANPEA RCGARGQGCW KVKREAVSEA EARCGARGQG
CWKVKRAAEA
      121 IADALAEPLP GCGARGQGCW KEKRDIQALQ NAARDALEKL *
//
LOCUS       L_subtilissima_ppg1_trunc       215 aa            linear
UNA
FEATURES             Location/Qualifiers
     KR              5..6
     KR              79..80
     AlphaRep        87..98
                     /label="AWAlpha"
     KR              99..100
     AlphaRep1       107..118
                     /label="AWAlpha"
     KR              119..120
     AlphaRep        135..146
                     /label="AWAlpha"
     KR              147..148
     AlphaRep1       157..168
                     /label="AWAlpha"
     KR              169..170
     AlphaRep        185..196
                     /label="AWAlpha"
     KR              197..198
ORIGIN
        1 CWKIKRAANA LAEALAEPAV DVIEARCNIA GGACQKARML TRDLAETVAA
VQDDPEAYYA
       61 SLSFEDDSAV ETAAIKQIKR EADADARCGA RGQGCWKEKR DAAPAPGCGA
RGQGCWKEKR
      121 EAISAAEAEA NPEARCGARG QGCWKIKREA VSEADAGCGA RGQGCWKVKR
AAEAIADALA
      181 EPLPRCGARG QGCWKEKRDI QALQNVARDV LEKL*
//
LOCUS       L_suecica_ppg1       233 aa            linear   UNA
FEATURES             Location/Qualifiers
     AlphaRep3       25..36
                     /label="AWAlpha"
     KR              37..38
     AlphaRep2       51..62
                     /label="AWAlpha"
     KR              65..66
     KR              139..140
     AlphaRep3       145..156
                     /label="AWAlpha"
     KR              159..160
     AlphaRep        177..186
                     /label="AWAlpha"
```

```
     KR              189..190
     AlphaRep3       201..212
                     /label="AWAlpha"
     KR              215..216
ORIGIN
        1 MQLTNALCVL ILAATSVVTA LPDAGRGCGA RGQGCYKRAA VALAEPVAYA
GHGCGARGQG
       61 CYKIKRAGEA LAEALAEEAT SEIDVRCDLA GGACHKARML ARDLADVVAA
TQDDSEAYFN
      121 SLNIEDADTF AEDAAEHIKR EAEAGRGCGA RGQGCYKNKR TAEAEAKADA
EAEAVAGCGA
      181 RGQGCYKAKR EADAEAEAEA GRGCGARGQG CYKEKRDLAA LRNVAREALA QL*
//
LOCUS       L_willkommii_ppg1       247 aa            linear   UNA
FEATURES             Location/Qualifiers
     AlphaRep        25..36
                     /label="AWAlpha"
     KR              37..38
     KR              111..112
     AlphaRep1       119..130
                     /label="AWAlpha"
     KR              131..132
     AlphaRep        139..150
                     /label="AWAlpha"
     KR              151..152
     AlphaRep1       167..178
                     /label="AWAlpha"
     KR              179..180
     AlphaRep        189..200
                     /label="AWAlpha"
     KR              201..202
     AlphaRep        217..228
                     /label="AWAlpha"
     KR              229..230
ORIGIN
        1 MQLTNALCVV ILAATTAVTA LPEPGCGARG QGCWKIKRAA NALAEALAEP
AVDVIEARCN
       61 IAGGACQKAR MLTRDLAETV AAVQDDPEAY YASLNFEDDS AVETAAIKQV
KREAEADARC
      121 GARGQGCWKE KRDAAPAPGC GARGQGCWKE KRVAISAAEA EANPEARCGA
RGQGCWKIKR
      181 EAVSEADAGC GARGQGCWKV KRAAEAIADA LAEPLPGCGA RGQGCWKEKR
DIQALQNAAR
      241 DALEKL*
//
LOCUS       P_dingleyae_ppg1       271 aa            linear   UNA
FEATURES             Location/Qualifiers
     AlphaRep        29..40
                     /label="AWAlpha"
     KR              41..42
     AlphaRep1       53..64
                     /label="AWAlpha"
     KR              65..66
     KR              139..140
     AlphaRep2       147..158
                     /label="AWAlpha"
```

```
     KR              159..160
     AlphaRep3       167..178
                     /label="AWAlpha"
     KR              179..180
     AlphaRep4       191..202
                     /label="AWAlpha"
     KR              203..204
     AlphaRep5       213..224
                     /label="AWAlpha"
     KR              225..226
     AlphaRep3       241..252
                     /label="AWAlpha"
     KR              253..254
ORIGIN
        1 MHIIRTLSIA ILAATAAATA LPNPNAEPWC GGRGQGCWKT KRVALPEPEA
FPGCGARGQG
       61 CWKIKRAGVA LRDALAEPAT GEIDARCNLA GGACFYSRQL ARDLATVVAS
TQDDPEAYFA
      121 SLDLEDEDAA ADEAAEKVKR EAEAEANCGG RGQGCWKEKR EPEPLPGCGA
RGQGCWKEKR
      181 MAVPEAFAEP GCGARGQGCW KVKREPIPEA DARCGARGQG CWKVKRAAEA
IAEAVAEAEP
      241 GCGARGQGCW KEKRDFAALQ NAARDALEKL *
//
LOCUS       C_crateriformis_ppg1       138 aa            linear
UNA
FEATURES             Location/Qualifiers
     AlphaRep        27..35
                     /label="AWAlpha"
     KR              36..37
     KR              50..51
     AlphaRep1       62..70
                     /label="AWAlpha"
     KR              71..72
     AlphaRep1       85..93
                     /label="AWAlpha"
     KR              94..95
     AlphaRep1       106..114
                     /label="AWAlpha"
     KR              115..116
     AlphaRep1       129..137
                     /label="AWAlpha"
ORIGIN
        1 MKVTQVFSVL LVAATVSAVA IPAPEAHCGR LGQPCKREAV AEVTADEIVK
REPGPIPEPE
       61 PRCGRMGQPC KREAAPEPVP EPEPRCGRMG QPCKREPGPI PEPEPRCGRM
GQPCKREAAP
      121 EPVPEPEPRC GRMGQPC*
//
LOCUS       C_cylindrospora_ppg1       163 aa            linear
UNA
FEATURES             Location/Qualifiers
     AlphaRep        27..35
                     /label="AWAlpha"
     KR              36..37
     KR              50..51
```

```
     AlphaRep1       62..70
                     /label="AWAlpha"
     KR              71..72
     AlphaRep1       85..93
                     /label="AWAlpha"
     KR              94..95
     AlphaRep1       108..116
                     /label="AWAlpha"
     KR              117..118
     AlphaRep1       131..139
                     /label="AWAlpha"
     KR              140..141
     AlphaRep1       154..162
                     /label="AWAlpha"
ORIGIN
        1 MKFTQVFSVL LVAATVSAVA IPAPEAHCGR LGQPCKREAL AEVTVDEIAK
REPEPVPEPE
       61 PRCGRMGQPC KREAAPEPAP EPEPRCGRMG QPCKREAAPE PAPEPEPRCG
RMGQPCKREA
      121 APEPVPEPEP RCGRMGQPCK REAAPEPAPE PKPRCGRMGQ PC*
//
LOCUS       P_neosporulosa_ppg1       140 aa            linear   UNA
FEATURES             Location/Qualifiers
     AlphaRep        25..33
                     /label="AWAlpha"
     KR              34..35
     AlphaRep        44..52
                     /label="AWAlpha"
     KR              53..54
     AlphaRep        67..75
                     /label="AWAlpha"
     KR              76..77
     KR              103..104
     KR              111..112
     AlphaRep        131..139
                     /label="AWAlpha"
ORIGIN
        1 MKFTQAFSVI ILAASVSAVA LPEARCGRVG QPCKREAAPA PEPRCGRVGQ
PCKRAAEAVA
       61 DALADPRCGR VGQPCKRDVA EADLDDIVAR LVTSDDDFFE KVKREVIAIS
KREAAPEPEP
      121 EPEPEPEPEA RCGRVGQPC*
//
LOCUS       P_radicicola_ppg1       136 aa            linear   UNA
FEATURES             Location/Qualifiers
     AlphaRep        25..33
                     /label="AWAlpha"
     KR              34..35
     AlphaRep        44..52
                     /label="AWAlpha"
     KR              53..54
     AlphaRep        67..75
                     /label="AWAlpha"
     KR              76..77
     KR              103..104
     KR              111..112
```

```
     AlphaRep1       127..135
                     /label="AWAlpha"
ORIGIN
        1 MKFTQAFSVI ILAASVSAAA LPEARCGRIG QPCKREAAPA PEPRCGRIGQ
PCKRAAEAVA
       61 DALADPRCGR IGQPCKRDVA EADLDDIVAR LVTVDNDVFE KVKREVIAIS
KREAAPEPEP
      121 EAEPEAKCGR IGQPC*
//
```
