# Supplementary material for: Needles in fungal haystacks: Discovery of a putative a-factor pheromone and a unique mating strategy in the Leotiomycetes
Source: PLoS One. 2023 Oct 12;18(10):e0292619. doi: 10.1371/journal.pone.0292619 (PMC10569646; doi:10.1371/journal.pone.0292619)
Supplement: S9 File — (PDF) [file pone.0292619.s019.pdf]

#Additional File S9: Alpha pheromone sequences for all Leotiomycetes (FASTA)

```
>A_resinae_ppg1
MRLTNALCVIILAATASAVAIPEPLALPLPEAYAEAKCGEFGECGRVKRAAEAFAEAFAE
PSAEPEDSIVMARCNLVGGACYEAKRLARDLASVLAHTRDEGSAFVARLALEDEETSDLK
AREAEAKCGKVGQPCGKIKRVAEPEAKCGKVGQPCGKAKRIADPEAEAKCGKVGQPCGKL
RRAAEAIAEAIAEPECGKVGQPCGKAKREADALAEAEAVCGKVGQPCGKAKRDALAAAIA
EADAKCGKVGQPCGKAKRDAHAIASAVSTVLEQL*

>A_pellizariae_ppg1
MQFATAIVVAVLAYSVNSAATPDAAAWCRYVGQPCGKVKRDGTPESEPWCRYVGQPCGKS
KREAEAACFLPGGPCAKAKRDANALAHAVADALPAAEAEAYYKALALRDAQPEPAPLAGA
EAEAWCRYFGQPCGKAKRSAVAFEDISEQAKRDAEAWCRYVGQPCGKAKRAAEAVAEAEA
WCRYVGQPCGKVKRGATPEAEPWCRYVGKPCGKSKRGAEAACDASGLCGKAKREAHAIAE
AVANIIEKY*

>A_tetracladia_ppg1
MKFASTFIAVAIAVSVSATAIPEACSVEGQSCMAIKRHAEAIATPDPWCWHPGQVCVKSK
RTATAYAEAWCWHPGQVCVKVKRAAEAFAEAIASPDEILDARCESSECFDARLKIRELAG
LVALTVRDATPEAYIESLNLRDETKLRRDASEHIEKREAEADAEATPWCWHPGQVCVKTK
RSCGDAGSPCDKLKRAAAALANAVAEPAAEPQCLNPGQICVKERREAEATAKAWCMTVPG
CNMAKRDAHALAAAANEVLAMYN*

>A_sarcoides_ppg1
MKLSHALSIFLLAATATAVAIPCGAPGQPCKREALPAPVPEPRCGAVGQPCKREAVPEPA
PGPRCGAVGQPCKREAEPEPAPEPVPCGAPGQPCKREVEPEPAPEPRCGAVGQPC*

>C_torta_ppg1
MKFTSAVCVIILAASASAIAIPEANAQHCGRPGQPCGKRDASPFPEPDAEALAQHCGRPG
QPCGKVRRAAEAMAEAFAEPEPQHCGRPGQPCGKREVEIAERCNLVGGACYEAKRLARDL
AGVVASTIEGDADAFYDNLNLEDRETSDKIARDAEAQHCGRPGQPCGKRDAEAEAQHCGR
PGQPCGKLRRAAEAIAEAVAEPQHCGRPGQPCGRDALPEPQHCGRPGQPCGKREAEAFAQ
HCGRPGQPCGKAKRNAHALAHAADVAVSQL*

>C_aeruginascens_ppg1
MKFATAIFVIVLAATATATALPEPTCGRPGQGCKREPLALPEAEADAIAIAICGSEGQPC
ARVKRAAEAFASAVSEPEAEPVDEMLLARCNLVGGACYEAKRMTRDLATEVAATQEDPEA
FYSSLDIEDEETSEKIRRDAEAKCGRPGQGCRRDADAEPEAICGRPGQGCRQLRRAAEAM
ANALADAKCGRPGQGCKDKAKREASALAEAEADARCGRPGQGCKDKLKREAVAVSIAEAV
AKCGRPGQGCRKAKRDAAALASAAEEALRSL*

>G_debralockiae_ppg1
MKYLSIIVLAAAVSAIAIPAPEPTCGKPGQPCRRNPEPVAVALPEPTCGKPGQPCKREAE
PVAVAAPEPTCGKSGQPCKREAAPAPEPTCGKPGQPCKREAEPIAVAAPQPTCGKPGQPC
KRKAAPAPEPTCGKPGQPCKREAEPVAVAAPEPTCGKPGQPCK*

>H_hymeniophilus_ppg1
MKYLSLVLLAAAVSAAPWCGKPGQPCPRSAEAIGEPWCYLPGKPGNPCPKKREAIPEPEP
WCGKPGQPCPKKREAIPEPEPVAEPWCGKPGQPCPKKREAIPEPEPVAEPWCGKPGQPCP
KKREAIPEPEPWCGKPGQPCPRSAIAEPEPIAEPWCGKPGQPCPKKREAIPEAEPVAEPW
CGKPGQPCPRSAIAEPEPIAEPWCGKPGQPCPKKREAYHKKVQWCGKPGQPCPREAEAEA
SA*

>M_aquatica_ppg1
MKFTTTLFAVALATSVSAIALPASEPQNCWRQGQICNKEKRDAEAQNCWRQGQICNKVKR
```

AADAFAEAVASPDDILDARCESAECFSARLKLRELAGLVAVTVRDAKPEAYLASLNLRDE
FEIRRDALAFQEKRAADPQNCWRQGQICNKEKREAEALAQNCWRQGQICNKERREADPEA
QNCWRQGQICNKLKRAAAAVAEALAEPQNCWRQGQICNKEKRDAEAEAVASCGEACTMAK
RDASALAAAANEILYMAN*

>N_alba_ppg1
MKFTSSLCIGIAAFAINTTALPVANPVPAAAPEPCNGWHSPGQPCKREADPEPIPEPEPE
PEADPCLYWHSPGQPCKREAXPEPIPEPEPEPEPEACNGWHSPGQPCKREAAPEPIPEPE
PEPEAEPCNGWYSPGQPCRRRRRSFKV*

>O_maius_ppg1
MKIASSVFLAVLVIGASTAPVPEAKRNPDPVLSPIMVARYGYPTGCGAPGEPCPKREAAP
EPVAAAIAEPRYGYPKGCGAPGEPCP*

>P_destructans_ppg1
MKYLATLCVAALVAGVNSAAIAAAEPFCWRLGQPCDKVKRAAEAFAEAFDEPIAEAEAFD
EPIAEAEASAFCWRPGQICEKAKRAALALAHTVADANPEAEAFFDKLAIDEAFPEPEAVA
DAEIADKVKREAEAEAFCWRPGQPCGKVKRAADAIASALAEPAPEPFCQRPGQLCGKVKR
DAEAVAEAFCWRPGQPCGKAKREANALAEAAAEALEFGGLEKEQNSKRIFRPPHYTTTAI
FPTDPRLFHHFHEEQPYDCRKVDPNCVTVEA*

>P_pannorum_ppg1
MKYLATICVAALVAGVNSAAIAVAEPEPICWRPDQPCGKVKRAAEAFAEAMAEPIAEPIA
EPICWRPGQQCGKVKREAEAAAICWRPGQPCGKAKRAALALAHAVANAQPEAEAFFDKLA
VREAFPEPEPEPEAIANAAPIDDKLKREAEAICWRPGQPCGKVKRAAEAIASALAKSEPE
AICWRPGQPCGKAKRDAEAEAEAICWRPGQPCGKAKREANALAEAAAETLASL*

>P_verrucosus_ppg1
MKYLANLCLAILVVGANSAAIAVAEPEPICWRPGQPCGKVKRAAEAFAEAIAEPIAEPIC
WRPGQPCGKVKREAEAEAEAAAICWRPGQPCGKAKRAALALAHAVANANPEAGAFFDKLA
IREAFPEPEAVAEAEPVDEKLKREAEAICWRPGQPCGKVKRAAEAIASALAEAAPEPICW
RPGQPCGKAKRDAEAAAEAICWRPGQPCGKAKREANALAEAAAEALASL*

>S_lignicola_ppg1
MKLTSVLAFAILAVARAPIPEPEPEPEPAPVAEPEPHPVAVAEPRCTRMGQPCGKKAKRE
AVPEPVPVPKAAPNPVAEPNPVCTRMGQACGKKAKREPIPEPVPEPEPAAKAEPNPVAAP
EPKCTRMGQPCGKKSKRVAVAEPEPEPEPMPVPLPEPQPEPEPLPEPAPAAKAVPNPVAE
PEPKCTRMGQPCGRKEKREPVPEPQPAPVAKAAPNPVAEPDPECTRMGQPCGRKFRA*

>V_echinocandica_ppg1
MKFTNAIAILFLTATTINALPSAEPCTHVGQGCWKDKREAVAEPDPCTHVGQGCWKHKLK
RLAEAEAEPCTHVGQGCWKEKQKRVAEAEAEADAAAEACTHVGQGCWKREADPCTHVGQG
CWKHKMKRVAEAYAEPCTHVGQGCWKEKQKRVAEAEAEADAAAEACTHVGQGCWKREADP
CTHVGQGCWKHKMKRVAEAYAEPCTHVGQGCWKEKMKRVAEAEAEAEAAAEACTHVGQGC
WKQ*

>B_calthae_ppg1
MKFTNAIALAILAATATAVAVPEPWCGRPGQPCKREAVAVAAPVAEAWCGRPGQPCKRTP
EAEAWCGRPGQPCKRDAEPWCGRPGQPCKREALPEAWCGRPGQPCKRTPLAEAEAEAWCG
RPGQPCRKNKRAAEAVAEAFAEPWCGRPGQPCKRDAEADVSEAAIKRCNMVGGACFEAKR
LARDLAEATAETVEDSDLFLRSLNIETREVSEVVAREAEAWCGRPGQPCKRDAEAWCRRP
GQPCKREALAEAEAWCGRPGQPCKEKREADPEAETWCGRPGQPCRAVKRAAEAIAEALAE
PIAEAWCGRHGQPCKREALAEAEANAEAWCGRPGQPCRKAKRDAFALAYAADVALAQL*

>B_convoluta_ppg1
MKFTNAIALAILAATATAVAVPEPWCGRPGQPCKREAVAVAVAAPVAEAWCGRPGQPCKR

DAEPWCGRPGQPCKREALPEAWCGRPGQPCKRTPLAEAEAEAWCGRPGQPCRKNKRAAEA
VAEAFAEPWCGRPGQPCRRDAEADVSEAAIKRCNMVGGACFEAKRLARDLAEATAETVED
ADAFLESLNIETREVSEVVAREAEAWCGRPGQPCKREALAEAEAWCGRPGQPCKRTAEPW
CGRPGQPCKEKREADPEAEAWCGRPGQPCRAVKRAAEAIAEALAEPTAEAWCGRPGQPCK
REALAEAEANAEAWCGRPGQPCRKAKRDAFALAYAADIALAQL*

>B_globosa_ppg1
MKFTNAIALAILAATATAVAVPEPWCGRPGQPCKREAVAVAAPVAEAWCGRPGQPCKRTP
EAEAWCGRPGQPCKRDAEPWCGRPGQPCKREALPEAWCGRPGQPCKRTPLAEAEAEAWCG
RPGQPCRKNKRAAEAVAEAFAEPWCGRPGQPCKRDAEADVSEAAIKRCNMVGGACFEAKR
LARDLAEATAETVEDSDAFLESLNIETREVSEVVAREAEAWCGRPGQPCKRDAEAWCGRP
GQPCKREALAEAEAWCGRPGQPCKREALAEAEAWCGRPGQPCKRTAEPWCGRPGQPCKEK
READPEAEAWCGRPGQPCRAVKRAAEAIAEALAEPTAEAWCGRPGQPCKREALAEAEANA
EAWCGRPGQPCRKAKRDAFALAYAADIALAQL*

>B_narcissicola_ppg1
MKFTNAIALAILAATATAVAVPEPWCGRPGQPCKREALPEAWCGRPGQPCKRTPLAEAEA
EAWCGRPGQPCRKNKRAAEAVAEAFAEPWCGRPGQPCKCDAEADVSEAAIKRCNMVGGAC
FEAKRLARDLAEATAETVEDSDAFLESLNIETREVSEVVAREAEAWCGRPGQPCKRDAEA
WCGRPGQPCKREALAEAEAWCGRPGQPCKRGALAEAEAWCGRPGQPCKRNAEAWCGRPGQ
PCKEKREADPEAEAWCGRPGQPCRAVKRAAEAIAEALAEPTAEAWCGRPGQPCKREALAE
AEANAEAWCGRPGQPCRKAKRDAFALAYAADIALAQL*

>B_squamosa_ppg1
MKFTNAIALAILAATATAVAVPECGRPGQPCKREAVAVAAPVAEAWCGRPGQPCKRTPEA
EAWCGRPGQPCKRDAEPWCGRPGQPCKREALPEAWCGRPGQPCKRTPLAEAEAEAWCGRP
GQPCRKNKRAAEAVAEAFAEPWCGRPGQPCKRDAEADISEAAIKRCNMVGGACFEAKRLA
RDLAEATAETVEDADAFLESLNIETREVSEVVAREAEAWCGRPGQPCKRDAEAWCGRPGQ
PCKREALAEAEAWCGRPGQPCKREALAEAEAWCGRPGQPCKRTAEPWCGRPGQPCKEKRE
ADPEAEAWCGRPGQPCRAVKRAAEAIAEALAEPTAEAWCGRPGQPCKREALAEAEANAEA
WCGRPGQPCRKAKRDAFALAYAADIALAQL*

>B_aclada_ppg1
MKFTNAIALAILAATATAVAVPEPWCGRPGQPCKREAVAVAAPVAEAWCGRPGQPCKRTP
EAEAWCGRPGQPCKRDAEPWCGRPGQPCKREALPEAWCGRPGQPCKRTPLAEAEAEAWCG
RPGQPCRKNKRAAEAVAEAFAEPWCGRPGQPCKRDAEADVSEAAIKRCNMVGGACFEAKR
LARDLAEATAETVEDADAFLESLNIETREVSEVVAREAEAWCGRPGQPCKRDAEAWCGRP
GQPCKREALAEAEAWCGRPGQPCKREALAEAEAWCGRPGQPCKRAAEPWCGRPGQPCKEK
READPEAEAWCGRPGQPCRAVKRAAEAIAEALAEPTAEAWCGRPGQPCKREALAEAEANA
EAWCGRPGQPCRKAKRDAFALAYAADIALAQL*

>B_byssoidea_ppg1
MKFTNAIALAILAATATAVAVPEPWCGRPGQPCKREAVAVAAPVAEAWCGRPGQPCKRDA
EPWCGRPGQPCKREALPEAWCGRPGQPCKRTPLAEAEAEAWCGRPGQPCRKNKRAAEAVA
EAFAKPWCGRPGQPCKRDAEADVSEAAIKRCNMVGGACFEAKRLARDLAEATAETVEDTD
AFLESLNIETREVSEVVAREAEAWCGRPGQPCKRDAEAWCGRPGQPCKREALAEAEAWCG
RPGQPCKREALAEAEAWCGRPGQPCKRTAEPWCGRPGQPCKEKREADPEAEAWCGRPGQP
CRAVKRAAEAIAEALAEPTAEAWCGRPGQPCKREALAEAEANAEAWCGRPGQPCRKAKRD
AFALAYAADIALAQL*

>B_cinerea_ppg1
MKFTNAIALAILAATATAVAVPEPWCGRPGQPCKREAVAVAAPVAEPWCGRPGQPCKRTP
EAEAWCGRPGQPCKRDAEPWCGRPGQPCKREALPEAWCGRPGQPCKRTPLAEAEAEAWCG
RPGQPCRKNKRAAEAVAEAFAEPWCGRPGQPCKRDAEADVSEAAIKRCNMVGGACFEAKR
LARDLAEATAETVEDSDLFLRSLNIETREVSEVVAREAEAWCGRPGQPCKRDAEAWCGRP
GQPCKREALAEAEAWCGRPGQPCKREALAEAEAWCGRPGQPCKRTAEPWCGRPGQPCKEK

READPEAEAWCGRPGQPCRAVKRAAEAIAEALAEPTAEAWCGRPGQPCKREALAEAEANA
EAWCGRPGQPCRKAKRDAFALAYAADVALAQL

>B_deweyae_ppg1
MKFTNAIALAILAATATAVAVPECGRPGQPCKREAVAVAAPVAEAWCGRPGQPCKRTPEA
EAWCGRPGEPCKRDAEPWCGHPGQPCKREALPEAWCGRPGQPCKRTPLAEAEAEAWCGRP
GQPCRKNKRAAEAVAEAFAEPWCGHPGQPCKRDAEADISEAAIKRCNMVGGACFEAKRLA
RDLAEATAETVEDADAFLESLNIETREVSEVVAREAEAWCGRPGEPCKRDAEAWCGRPGQ
PCKREALAEAEAWCGRPGQPCKREALAEAEAWCGRPGQPCKRTAEPWCGRPGQPCKEKRE
ADPEAEAWCGRPGQPCRAVKRAAEAIAEALAEPTAEAWCGHPGQPCKREALAEAEANAEA
WCGLPGQPCRKAKRDAFALAYAADIALAQL*

>B_elliptica_ppg1
MKFTNAIALAILAATATAVAVPECGRPGQPCKREAVAVAAPVAEAWCGRPGQPCKRTPEA
EAWCGLPGQPCKRDAEPWCGLPGQPCKREALPEAWCGLPGQPCKRTPLAEAEAEAWCGRP
GQPCRKNKRAAEAVAEAFAEPWCGRPGQPCKRDAEADISEAAIKRCNMVGGACFEAKRLA
RDLAEATAETVEDADAFLESLNIETREVSEVVAREAEAWCGRPGQPCKRDAEAWCGRPGQ
PCKREALAEAEAWCGRPGQPCKREALAEAEAWCGRPGQPCKRTAEPWCGRPGQPCKEKRE
ADPEAEAWCGLPGQPCRAVKRAAEAIAEALAEPTAEAWCGLPGQPCKREALAEAEANAEA
WCGLPGQPCRKAKRDAFALAYAADIALAQL*

>B_fabae_ppg1
MKFTNAIALAILAATATAVAVPEPWCGRPGQPCKREAVAVAAPVAEPWCGRPGQPCKRTP
EAEAWCGRPGQPCKRDAEPWCGRPGQPCKREALPEAWCGRPGQPCKRTPLAEAEAEAWCG
RPGQPCRKNKRAAEAVAEAFAEPWCGRPGQPCKRDAEADVSEAAIKRCNMVGGACFEAKR
LARDLAEATAETVEDSDLFLRSLNIETREVSEVVAREAEAWCGRPGQPCKRDAEAWCGRP
GQPCKREALAEAEAWCGRPGQPCKREALAEAEAWCGRPGQPCKRTAEPWCGRPGQPCKEK
READPEAEAWCGRPGQPCRAVKRAAEAIAEALAEPTAEAWCGRPGQPCKREALAEAEDNA
EAWCGRPGQPCRKAKRDAFALAYAADVALAQL*

>B_fragariae_ppg1
MKFTNAIALAILAATATAVAVPEPWCGRPGQPCKREAVAVAAPVAEAWCGRPGQPCKRTP
EAEAWCGRPGQPCKRDAEPWCGRPGQPCKREALPEAWCGRPGQPCKRTPLAEAEAEAWCG
RPGQPCRKNKRAAEAVAEAFAEPWCGRPGQPCKRDAEADVSEAAIKRCNMVGGACFEAKR
LARDLAEATAETVEDSDAFLESLNIETREVSEVVAREAEAWCGRPGQPCKRDAEAWCGRP
GQPCKREALAEAEAWCGRPGQPCKREALAEAEAWCGRPGQPCKRTAEPWCGRPGQPCKRE
ALAEAEAWCGRPGQPCKREALAEAEAWCGRPGQPCKRTAEPWCGRPGQPCKEKREADPEA
EAWCGRPGQPCRAVKRAAEAIAEALAEPTAEAWCGRPGQPCKREALAEAEANAEAWCGRP
GQPCRKAKRDAFALAYAADIALAQL*

>B_galanthina_ppg1
MKFTNAIALAILAATATAVAVPEPWCGRPGQPCRREAVAVAAPVAEAWCGRPGQPCKRTP
EAEAWCGRPGQPCKRDAEPWCGRPGQPCKREALPEAWCGRPGQPCKRTPLAEAEAEAWCG
RPGQPCRKNKRAAEAVAEAFAEPWCGRPGQPCKRDAEADVSEAAIKRCNMVGGACFEAKR
LARDLAEATAETVEDSDAFLESLNIETREVSEVVAREAEAWCGRPGQPCKRDVEAWCGRP
GQPCKREALAEAEAWCGRPGQPCKREALAEAEAWCGRPGQPCKRTAEPWCGRPGQPCKEK
READPEAEAWCGRPGQPCRAVKRAAEAIAEALAEPTAEAWCGRPGQPCKREALAEAEANA
EAWCGRPGQPCRKAKRDAFALAYAADIALAQL*

>B_hyacinthi_ppg1
MKFTNAIALAILAATATAVAVPEPWCGRPGQPCKREAVAVAAPVAEAWCGRPGQPCKRTS
EAEAWCGRPGQPCKRDAEPWCGRPGQPCKREALPKAWCGRPGQPCKRTPLAKAEAEAWCG
RPGQPCRKNKRAAEAVAEAFAEPWCGRPGQPCKRDAEAEVSEAAIKRCNMVGGACFEAKR
LARDLAEATAETVEDSDAFLESLNIETREVSEVVAREAEAWCGRPGQPCKRDAEAWCGRP
GQPCKREALAEAEAWCGRPGQPCKRTAEAWCGRPGQPCKEKREADPEAEAWCGRPGQPCS
AVKRAAEAIAEALAEPTAEAWCGRPGQPCKREALAEAEANAEAWCGRPGQPCRKAKRDAF

ALAYAADIALAQL*

>B_medusae_ppg1
MKFTNAIALAILAATATAVAVPEPWCGRPGQPCKREAVAVAAPVAEAWCGRPGQPCKRTP
EAEAWCGRPGQPCKRDAEPWCGRPGQPCKREALPEAWCGRPGQPCKRTPLAEAEAEAWCG
RPGQPCRKNKRAAEAVAEAFAEPWCGRPGQPCKRDAEADVSEAAIKRCNMVGGACFEAKR
LARDLAEATAETVEDSDLFLRSLNIETREVSEVVAREAEAWCGRPGQPCKRDAEAWCGRP
GQPCKREALAEAEAWCGRPGQPCKREALAEAEAWCGRPGQPCKRTAEPWCGRPGQPCKEK
READPEAEAWCGRPGQPCRAVKRAAEAIAEALAEPTAEAWCGRPGQPCKREALAEAEANA
EAWCGRPGQPCRKAKRDAFALAYAADVALAQL*

>B_paeoniae_ppg1
MKFTNAIALAILAATATAVAVPEPWCGRPGQPCKREAVAVAAPVAEAWCGRPGQPCKREA
LPEAWCGRPGQPCKRTPLAEAEAEAWCGRPGQPCRKNKRAAEAVAEAFAEPWCGRPGQPC
KRDAEADVSEAAIKRCNMVGGACFEAKRLARDLAEATAETVEDADAFLESLNIETREVSE
VVAREAEAWCGRPGQPCKREALVEAWCGRSGQPCKREALAEAEAWCGRPGQPCKRTAEPW
CGRPGQPCKEKREADPEAEAWCGRPGQPCRAVKRAAEAIAEALAEPTAEAWCGRPGQPCK
REALAEAEANAEAWCGRPGQPCRKAKRDTFALAYAADIALAQL*

>B_porri_ppg1
MKFTNAIALAMLAATATAVAVPEPWCGRPGQPCKREAVAVAAPVAEAWCGRPGQPCKRTP
EAEAWCGRPGQPCKRTPLAEAEAEAWCGRPGQPCRAVKRAAEAIAEALAEPTAEAWCGRP
GQPCKREALAEAEANAEAWCGRPGQPCRKAKRDAFALAYAADIALAQL*

>B_pseudocinerea_ppg1
MKFTNAIALAILAATATAVAVPEPWCGRPGQPCKREAVAVAAPVAEPWCGRPGQPCKRTP
EAEAWCGRPGQPCKRDAEPWCGRPGQPCKREALPEAWCGRPGQPCKRTPLAEAEAEAWCG
RPGQPCRKNKRAAEAVAEAFAEPWCGRPGQPCKRDAEADVSEAAIKRCNMVGGACFEAKR
LARDLAEATAETVEDSDLFLRSLNIETREVSEVVAREAEAWCGRPGQPCKRDAEAWCGRP
GQPCKREALAEAEAWCGRPGQPCKRTAEPWCGRPGQPCKEKREADPEAEAWCGRPGQPCR
AVKRAAEAIAEALAEPTAEAWCGRPGQPCKREALAEAEANAEAWCGRPGQPCRKAKRDAF
ALAYAADVALAQL*

>B_sinoallii_ppg1
MKFTNAIALAILAATATAVAVPEPWCGRPGQPCKREAVAVAAPVAEAWCGRPGQPCKRTP
EAEAWCGRPGQPCKRDAEPWCGRPGQPCKREALPEAWCGRPGQPCKRTPLAEAEAEAWCG
RPGQPCHKNKRAAEAVAEAFAEPWCGRPGQPCKRDAEADISEAAIKRCNMVGGACFEAKR
LARDLAEATAETVEDADAFLESLNIETREVSEVVAREAEAWCGRPGQPCKRDAEAWCGRP
GQPCKREALAEAEAWCGRPGQPCKRTAEPWCGRPGQPCKEKREADPEAEAWCGRPGQPCR
AVKRAAEAIAEALAEPTAEAWCGRPGQPCKREALAEAEANAEAWCGRPGQPCRKAKRDAF
ALAYAADIALAQL*

>B_tulipae_ppg1
MKFTNAIALAILAATATAVAVPEPWCGRPGQPCKREAVAVAAPVAETWCGRPGQPCKRTP
EAEAWCGRPGQPCKRDAEPWCGRPGQPCKREALPEAWCGRPGQPCKRTPLAEAEAEAWCG
RPGQPCRKNKRAAEAVAEAFAEPWCGRPGQPCKRDAEADVSEAAIKRCNMVGGACFEAKR
LARDLAEATAETVEDSDAFLESLNIETREVSEVVAREAEAWCGRPGQPCKREALAEAEAW
CGRPGQPCKREALAEAEAWCGRPGQPCKRTAEPWCGRPGQPCKEKREADPEAEAWCGRPG
QPCRAVKRAAEAIAEALAEPTAEAWCGRPGQPCKREALAEAEANAEAWCGRPGQPCRKAK
RDAFALAYAADIALAQL*

>C_camelliae_ppg1
MKFTNAIALAILAATATAVALPEPWCGRPGQPCKREAVAIAAPAPEPWCGRPGQPCKRTP
EAEAWCGRPGQPCKRDAEPWCGRPGQPCKREALPEAWCGRPGQPCKRTALAEAEAEAWCG
RPGQPCRKNKRAAEALAEAFAEPTEETDVDEAVVKRCNMVGGACFEAKRLARDLAEATAE
TSEDATAFLEGLNIETREASEIIAREAEAWCGRPGQPCKRSAEPWCGRPGQPCKRDAEPW

CGRPGQPCKRDAEPWCGRPGQPCKEKREADPEAEAWCGRPGQPCRAVRRAAEAMADALAE
PTAEAWCGRPGQPCKREALAEAEANAEAWCGRPGQPCRKAKRDAMALAYAADVALAQL*

>C_shiraiana_ppg1
MKFTNAIALAILAATATAVAIPEPWCGRPGQPCKREAVAVAAPIPEPWCGRPGQPCKRDA
EPWCGRPGQPCKREALPEAWCGRPGQPCKRTPLAEAEAEAWCGRPGQPCRKNKRAAEALA
EAFAEPAAEADVDEAVIKRCNMVGGACFEAKRLARDLAEATAETKRDADAFLEGLNIESR
EVSEIVAREAEAWCGRPGQPCKRNAEAWCGRPGQPCKRDAEPWCGRPGQPCKEKREADPE
AEAWCGRPGQPCRAVRRAAEAMAEALAEPTAEAWCGRPGQPCKREALAEAEANAEAWCGR
PGQPCRKAKRDAFALAYAADVVLAQL*

>M_curreyana_ppg1
MKFTNAIALAILAATATAVAVPEPWCGRPGQPCKREAVAIADPIAEAWCGRPGQPCKRDA
EPWCGRPGQPCKREALPEAWCGRPGQPCKRSPLAEAEAEAWCGRPGQPCRKNKRAAEAVA
EAFAEPWCGRPGQPCKRDAEADVDEAVIKRCNMVGGACFEAKRLARDLAEATAETVEDPD
TFLEGLNIETREVSEIVAREAEAWCGRPGQPCKRSAEPWCGRPGQPCKRDAQPEAEPWCG
RPGQPCKEKREADAEAEAWCGRPGQPCRAVKRAAEAIAEALAEPTAEAWCGRPGQPCKRE
ALAEAEANAEAWCGRPGQPCRKAKRDAFALAYAADVALAQL*

>M_duriaeana_ppg1
MKFTNAIALAILAATATAVAVPEPWCGRPGQPCKREAVAVADPVAEAWCGRPGQPCKREA
EPWCGRPGQPCKREALPEAWCGRPGQPCKRSPLAEAEAEAWCGRPGQPCRKNKRAAEAVA
EAFAEPWCGRPGQPCKRDAEADVDEAVVKRCNMVGGACFEAKRLARDLAEATAETVEDPD
AYLEGLNIETREVSEIVAREAEAWCGRPGQPCKRSAEPWCGRPGQPCKRDAQPEAEPWCG
RPGQPCKEKREADPEAEAWCGRPGQPCRAVKRAAEAIAEALAEPTAEAWCGRPGQPCKRE
ALAEAEANAEAWCGRPGQPCRKAKRDAFALAYAADVALAQL*

>M_scirpicola_ppg1
MKFTNAIALAILAATATAVAVPEPWCGRPGQPCKREAVAVADPVAEAWCGRPGQPCKREA
EPWCGRPGQPCKREALPEAWCGRPGQPCKRSPLAEAEAEAWCGRPGQPCRKNKRAAEAVA
EAFAEPWCGRPGQPCKRDAEADVDEAVVKRCNMVGGACFEAKRLARDLAEATAETVEDPE
AYLEGLNIETREVSEIVAREAEAWCGRPGQPCKRSAEPWCGRPGQPCKRDAQPEAEPWCG
RPGQPCKEKREADPEAEAWCGRPGQPCRAVKRAAEAMAEALAEPTAEAWCGRPGQPCKRE
ALAEAEANAEAWCGRPGQPCRKAKRDAFALAYAADVALAQL*

>M_aucupariae_ppg1
MKFTNAIALAILAATAAAVAVPEAQPWCGRPGQGCKREALAAPVSEAQPWCGRPGQGCKR
EAEAWCGRPGQGCKRDAEPWCGRPGQGCKREALPEAWCGRPGQGCKRAPLPEAEAEAWCG
RPGQGCRKTKRAAEALAEALAEPEAEADVEEEVAKRCNMVGGACFEAKRLARDLAETVAE
TTADPAAYLESLNLETREVSEVIAREAEAWCGRPGQGCKRNAEAEADAEAEAWCGRPGQP
CRKVRRSAEAMAEALAEATPVAWCGRPGQGCKREALAEAEANAEAWCGRPGQGCRKAKRD
AYALAYAADMVLKQL*

>M_fructicola_ppg1
MKFTNAIALAILAATATAVAVPEAQPWCGRPGQGCKREAVAAPVAAPVSEAQPWCGRPGQ
GCKREAEAWCGRPGQGCKRDAEPWCGRPGQGCKREALPEAWCGRPGQGCKRAPLAEAEAE
AWCGRPGQGCRKTKRAAEALAEALAEPEAEADVEEAVIKRCNMVGGACFEAKRLARDLAE
TVAETTSDPAAYLESLNLETREVSEVVAREAEAWCGRPGQGCKRNAEAEADAEAEAWCGR
PGQGCRKREALAEAEANAEAWCGRPGQGCRKAKRDAYALAYAADMALKQL*

>M_fructigena_ppg1
MKFTNAIALAILAATATAVAVAAPAAAPVSEAQPWCGRPGQGCKREAEAWCGRPGQGCKR
DAEPWCGRPGQGCKREALPEAWCGRPGQGCKRAPLPEAEAEAWCGRPGQGCRKTKRAAEA
LAEALAEPEAEADVEEAVIKRCNMVGGACFEAKRLARDLAETVAETTSDPAAYLESLNLE
TREVSEVVAREAEAWCGRPGQGCKRNAEAEADAEAEAWCGRPGQGCRKVRRAAEVMAEAL
AEATPVAWCGRPGQGCKREALAEAEANAEAWCGRPGQGCRKAKRDAYALAYAADLALKQL

*

>M_laxa_ppg1
MKFTNAIALAILAATATAVAVPEAQPWCGRPGQGCKREAIAAPAAAPVSEAQPWCGRPGQ
GCKREAEAWCGRPGQGCKRDAEPWCGRPGQGCKREALPEAWCGRPGQGCKRAPLPEAEAE
AWCGRPGQGCRKTKRAAEALAEALAEPEAEADAEEAVIKRCNMVGGACFEAKRLARDLAE
TVAETTSDPAAYLESLNLETREVSEVVAREAEAWCGRPGQGCKRNAEAEADAEAEAWCGR
PGQGCRKVRRAAEAMAEALAEATPVAWCGRPGQGCKREALAEAEANAEAWCGRPGQGCRK
AKRDAYALAYAADLALKQL*

>M_polystroma_ppg1
MKFTNAIALAILAATATAVAVAAPAAAPVSEAQPWCGRPGQGCKREAEAWCGRPGQGCKR
DAEPWCGRPGQGCKREALPEAWCGRPGQGCKRAPLPEAEAEAWCGRPGQGCRKTKRAAEA
LAEALAEPEAEADVEEAVIKRCNMVGGACFEAKRLARDLAETVAETTSDPAAYLKSLNLE
TREVSEVVAREAEAWCGRPGQGCKRNAEAEADAEAEAWCGRPGQGCRKVRRAAEAMAEAL
AEATPVAWCGRPGQGCKREALAEAEANAEAWCGRPGQGCRKAKRDAYALAYAADLALKQL
*

>M_vacciniicorymbosi_ppg1
MKFTNSIALAILAATAIAVAVPETQPWCGRPGQPCKREAVAAPVSEAQPWCGRPGKPCKR
NAEPWCGRPGQPCKRAPLPEAEAEAWCGRPGQGCRKTKRAAEALAEALAEPEAEADVEEA
VVKRCNMVGGACFEAKRLARDLAETVAETTADPAAYLESLTLETREASEVAAREAEAWCG
RPGQPCKRSAEAEADAEAEAWCGRPGQPCRKVRRSAEAMAEALAEATPVAWCGRPGQPCK
RGAEAEALAEAEANAEAWCGRPGQPCRKAKRNAYALAYAADMALKQL*

>S_borealis_ppg1
MKFTNAIALAILAAIATAAAVPEAQPWCGRPGQACKREAVAGPVAEPWCGRPGQPCKRTP
LAEAEAEAWCGRPGQPCRKNKRAAEALAEAFAEPAAEADVDEAVIKRCNMVGGACFEAKR
LARDLAEATAETVEDSTAFLESLNIETREISEVVAREAEAWCGRPGQPCKRDAQPEAEAW
CGRPGQPCKRNAEPWCGRPGQPCKEKREADAEAEAWCGRPGQPCRAVRRAAEAVAEALAE
ATAEAWCGRPGQPCKREAIAEAEANAEAWCGRPGQPCRKAKRDAYALAYAADVALAQL*

>S_cepivorum_ppg1
MKFTNAIALAILAATATAVAVPEPWCGRPGQPCKREAVAVAAPIAEPWCGRPGQPCKRSP
EAEAWCGRPGQPCKRDAEPWCGRPGQPCKREALPEAWCGRPGQPCKRTPLAEAEAEAWCG
RPGQPCRKNKRAAEALAEAFAEPAAEADVDEAVIKRCNMVGGACFEAKRLARDLAEATAE
TKRDADAFLEGLNIESREVSEIVAREAEAWCGRPGQPCKRNAEAWCGRPGQPCKREAEAW
CGRPGQPCKRDAEPWCGRPGQPCKEKREADPEAEAWCGRPGQPCRAVRRAAEAMADALAE
PTAEAWCGRPGQPCKREALAEAEANAEAWCGRPGQPCRKAKRDAFALAYAADVALAQL*

>S_glacialis_ppg1
MKFTSAIAIAILAATATAAAIPAPSSDQPSKRNITATQDIAWCGRPGQPCHIKRDALPDI
QAAAEAAVICHPGQPCSQKHNPTWCGQPGRPCKRDALPDIQNVAWCGRPGQPCHKNKRAA
QALAEALAEPADGADVDEAVVKRCNMVGGACFEAKRLARDLAQTTAETSDDAQTFLDGLN
VETREASEIIAREAEAWCGRPGQPCKREAEAWCGRPGQPCKREAEAWCGRPGQPCKRDAE
PWCGRPGQPCKEKREADPEAEAWCGRPGQPCRAVRRAAEAIAKALAEPTAEAWCGRPGQP
CKRDALAEPWCGRPGQPCKREADAGAWCGRPGQPCKREALAVAEADAEAWCGRPGQPCRK
AKRDAYALAYAADVALAQL*

>S_sclerotiorum_ppg1
MKFTNAIALAILAATATAVAIPEPWCGRPGQPCKREAVAVAAPIPEPWCGRPGQPCKRDA
EPWCGRPGQPCKREALPEAWCGRPGQPCKRTPLAEAEAEAWCGRPGQPCRKNKRAAEALA
EAFAEPAAEADVDEAVIKRCNMVGGACFEAKRLARDLAEATAETKRDADAFLEGLNIESR
EVSEIVAREAEAWCGRPGQPCKRNAEAWCGRPGQPCKRDAEPWCGRPGQPCKEKREADPE
AEAWCGRPGQPCRAVRRAAEAMAEALAEPTAEAWCGRPGQPCKREALAEAEANAEAWCGR
PGQPCRKAKRDAFALAYAADVVLAQL

```
>S_trifoliorum_ppg1
MKFTNAIALAILAATAAAVAVPEPWCGRPGQLCKRTPLAEAEAEAWCGRPGRPCRKTKRA
AEALAEAFAEPAAEAYVDEAVIKRCNMVGGACFEAKRLARDLAEATAETKRDADAFLEGL
NIESREVSEIVAREAEAWCGRPGQPCKRNAEAECGRPGQSCKEKRKADPEAEAWCGSPGQ
PCRAVRRAAEAMAEALAEPTAEAWCGPCRKAKRDAFALAYAANVALAQL*

>C_homoeocarpa_ppg1
MKLTNAIALAILAATAAAVAIPEPWCGRPGQPCKRDALPEPWCGRPGQPCKRDALPEAWC
GRPGQPCKRSALAEPDAEAWCGRPGQPCRKVRRAAEALAKAMAEPVADVEADVDEAVVKR
CNMIGGPCYEAKRLARDLAGVTASVRRDADAFYEDLGIESREVSDKITRDAEAWCGRPGQ
PCKRDAAPEAEPWCGRPGQPCKRDAAPEAEPWCGRPGQPCKEKREADPEAEAWCGRPGQP
CRKVRRAAEAMAEALAEPTAEAWCGRPGQPCKREALAEAEANAEAWCGRPGQPCRKAKRD
AFALAYAADVALQQL*

>C_jacksonii_ppg1_trunc
MKLTNAIALAILAATAAAVAIPEPWCGRPGQPCKRDALPEPWCGRPGQPCKRDALPEAWC
GRPGQPCKRSALAEPDAEAWCGRPGQPCRKVRRAAEALAKAMAEPVADVEADVDEAVVKR
CNMIGGPCYEAKRLARDLAGVTASVRRDADAFYEDLGIESREVSDKIARDAEAWXXXXXX
XXXXXXXXXXXXXXXXXXXXXXXXXXXXXXXXXXXXXXXXXXXXXXXXXDAAPEAEPWCG
RPGQPCKEKREADPEAEAWCGRPGQPCRKVRRAAEAMAEALAEPTAEAWCGRPGQPCKRE
ALAEAEANAEAWCGRPGQPCRKAKRDAFALAYAADVALRQL*

>C_monteithiana_ppg1
MKLTNAIALAILAATAAAVAIPEPWCGRPGQPCKRDALPEPWCGRPGQPCKREALPEAWC
GRPGQPCKRSALAEPDAEAWCGRPGQPCRKVRRAAEALAEAMAEPVADVEADVDEAVVKR
CNMIGGPCYEAKRLARDLAGVTASVRRDADAFYEDLGIESRDVSDKIARDAEAWCGRPGQ
PCKRDAAPEAAPEAEPWCGRPGQPCKEKREADPEAEAWCGRPGQPCRKVRRAAEAMAEAL
AEPTAEAWCGRPGQPCKREALAEAEANAEAWCGRPGQPCRKAKRDAFALAYAADVALRQL
*

>C_spCPB17_ppg1
MKFTNAIALAILAATATAVAIPEPWCGRPGQPCKRDALPEPWCGRPGQPCKREALPEAWC
GRPGQPCKRSALAEPDAEAWCGRPGQPCRKVRRAAEALAEAMAEPAAEAEADVDEAVVKR
CNIVGGACYEAKRLARDLAGVTASTKRDADAFYEELGIESREVSDKVARDAEAWCGRPGQ
PCKRDAAPEAEAEAWCGRPGQPCKRDAEPWCGRPGQPCKEKREADPEAEAWCGRPGQPCR
QVRRAAEAMAEALAEPTAEAWCGRPGQPCKREALAEAEANAEAWCGRPGQPCRKAKRDAF
ALAYAADVALAQL*

>C_spSE16F4_ppg1
MKLTNAIALAILAATAAAVAIPEPWCGRPGQPCKRDALPEPWCGRPGQPCKRDALPEAWC
GRPGQPCKRSALAEPDAEAWCGRPGQPCRKVRRAAEALAKAMAEPVADVEADVDEAVVKR
CNMIGGPCYEAKRLARDLAGVTASVRRDADAFYEDLGIESREVSDKITRDAEAWCGRPGQ
PCKRDAAPEAEPWCGRPGQPCKRDAAPEAEPWCGRPGQPCKEKREADPEAEAWCGRPGQP
CRKVRRAAEAMAEALAEPTAEAWCGRPGQPCKREALAEAEANAEAWCGRPGQPCRKAKRD
AFALAYAADVALQQL*

>R_spNJR-2017aBBW_ppg1
MKFTSTLALAILAATATAIALPEPWCGRPGQPCKRDALPEPWCGRPGQPCKRESLPEAWC
GRPGQPCKRSPLAEADAEAWCGRPGQPCRKVRRAAEALAEALAEPVADAEADFDEAVVKR
CNMVGGACYEAKRLARDLAGVTASAKRDADAFYEGLGIESREVSDKIARDAEAWCGRPGQ
PCKRSADAEPWCGRPGQPCKRDASPEPWCGRPGQPCKEKREADPEAEAWCGRPGQPCRKV
RRAAEAMAEALAEPTAEAWCGRPGQPCKREALAEAEANAEAWCGRPGQPCRKAKRDAFAL
AYAADVALQQL*

>R_spNJR2017aBVV2_ppg1
```

```
MKFTNAIALAILAATATAVAIPEPWCGRPGQPCKREALPEPWCGRPGQPCKREALPEAWC
GRPGQPCKRSPLAEPDAEAWCGRPGQPCRKVRRAAEALAEAMAEPAADAEADFDEAVVKR
CNMVGGACYEAKRLARDLAGVTASVRRDADAFYEELGIESREVSDKIARDAEAWCGRPGQ
PCKREANPEAEAEAEAWCGRPGQPCKRDAEPWCGRPGQPCKEKREADAEAEAWCGRPGQP
CRKVRRAAEAMAEALAEPTAEAWCGRPGQPCKREALAEAEANAEAWCGRPGQPCRKAKRD
AFALAYAADVALQQL*

>R_spNJR2017aWRK4_ppg1
MKFTSTLALAILAATATAIALPEPWCGRPGQPCKRDALPEPWCGRPGQPCKREALPEAWC
GRPGQPCKRSPLAEADAEAWCGRPGQPCRKVRRAAEALAEAMAEPVADAEADFDEAVVKR
CNMVGGACYEAKRLARDLAGVTASAKRDADAFYEGLGIESREVSDKIARDAEAWCGRPGQ
PCKRTASADAEPWCGRPGQPCKEKREADPEAEAWCGRPGQPCRKVRRAAEAMAEALAEPT
AEAWCGRPGQPCKREALAEAEANAEAWCGRPGQPCRKAKRDAFALAYAADVALQQL*

>C_malorum_ppg1
MKLTSAICALALAATVSSSAIPWAAAEARCGHPGQPCGKVRRVALPEPRCGHPGQPCGKR
DAMPEPAPEPRCGHPGQPCGKRDAMPAPEPEARCGHPGQPCGKRDAIPEPAPEPEARCGH
PGQPCGKRDAIPEPAPEPEPRCGHPGQPCGKRNAIPEPAPEPEARCGHPGQPCGKRDAMP
EPEPEARCGHPGQPCGKREFVA*

>L_spMPISDFRAT0119_ppg1
MKLTSAICALVLAATVSSSAIPWAAAEAKCGHPGQPCGKSKRVALPEPEAICGHPGQPCG
KRDAIPEPAPEPEARCGHPGQPCGKRDAMPEPEPEAICGHPGQPCGKRDAMPEPEPEAIC
GHPGQPCGKKM*

>N_avenae_ppg1
MKLTSAICALIFAATVSSSAIPLAAAEARCGHPGQPCGKFKRAALAEPAARCGFPGQPCG
RDAIPAPAPEPEARCGHPGQPCGKRDAMPEPEARCGHPGQPCGKRDAVPEPEARCGHPGQ
PCGKRDAIPEPEVRCGHPGQPCGKRDAIPEPEARCGHPGQPCGRDLGKRTPIPRCGQGPY
CPGSYEWEVYMVRAKNHVSWAEARAIVDKYWRKKRDANAEVIVAKTFGKREAIPEARCGH
PGQPCGKRDAIPEPAPEARCGHPGQPCGKRDAIPEPAPEARCGHLGQPCGKRDAIPEPAP
EARQPYGKRNALPEPIPEPKLGVVVGLVASATSVTPPPNRSIDTQA*

>R_spMPIPUGEAT0058_ppg1
MKLTSAICALIFAATVSSSAIPWAAAEARCGHPGQPCGKFKRVALPEPEARCGHPGQPCG
KRDAIPEPAPEARCGHPGQPCGKRDAIPEPAPEPEARCGHPGQPCGKRDAIPEPAANPEA
RCGHPGQPCGKRDAMPEPEPEPEARCGHPGQPCGKRDAMPEPEPEARCGHPGQPCGKKL*

>R_commune_ppg1
MKFTSAICALIFAATVSSSAIPWAAAEARCGHPGQPCGKFKRVALPEPVARCGHPGQPCG
RDAIPAPAPEPEARCGHPGQPCGKRDAIPEPEARCGHPGQPCGKRDAIPAPAPEPEARCG
HPGQPCGKRDAIPEPEARCGHPGQPCGKRDAIPEPAPEARCGHPGQPCGKRDAIPEPAPE
ARCGHPGQPCGKRDAIPEAN*

>B_jaapii_ppg1
MKLTSAICALILAASVSASALPVANAEARCGYPGQPCGKVRREAIAEPEARCGYPGQPCG
KAKREAAPVAEARCGYPGQPCGKAKREAAPVAEAAPEARCGYPGQPCGKAKREALPEPEA
RCGYPGQPCGKAKREAVAEAEPEARCGYPGQPCGKAKREAEALPEPEARCGYPGQPCGKA
KREALPEPEARCGYPGQPCGKAKRDE*

>D_brunnea_ppg1
MKLTSAICALIFAATVSASALPVANAEARCGYRGQPCPKAKREAIAEARCGYRGQPCPKA
KREAEALPEALPEAEARCGYRGQPCPKAKREAEALPEPEARCGYRGQPCPKK*

>D_rosae_ppg1
MKLTSAICALVFAASVTASALPAAAAEARCGHPGQPCGKAKRVALPEPEARCGHPGQPCG
```

KAKREAAPRCGHPGQPCGKVKRAAEAFAEALAEPEAEASADRTTLDARCNLVGGACYEAK
RLTRDLAAIVASTQDDADDYYDSLEIEDEDTSEKVRRDAEARCGHPGQPCGKAKREPEAR
CGHPGQPCGKAKREAEARCGHPGQPCGKAKREADPEAEARCGHPGQPCGKLRRAAEAIAE
AVAEAEPEARCGHPGQPCGKAKRDAHALAHASDVALSQL*

>M_coronariae_ppg1
MKLTSAICALIFAASVTASALPVAAAEARCGKPGQPCGKAKRVALPEPEARCGKPGQPCG
KVKRAAEAFAEALAAPDVSFLSSPASGLSWPATKNSFQAEDPAEWATLDARCNLVGGACY
EAKRLTRDLAAIVASTQDDADEYYDSLEIEDEDTSEKVRRNAEARCGKPGQPCGKVKREA
EARCGKPGQPCGKAKREADPEAEARCGRPGQPCGKLRRAAEAIAEAVAEPEPEARCGKPG
QPCGKAKRDAHALAHAADVAISQL*

>M_rosae_ppg1
MKLTSAICALIFAASVTASALPAAAAEARCGHPGQPCGKAKRVALPEPEARCGHPGQPCG
KAKREAAPRCGHPGQPCGKVKRAAEAFAEALAEPEVSSISLPPVTSSCSNTHDAIQAEAS
ADWTTLDARCNLVGGACYEAKRLTRDLAAIVASTQDDADDYYDSLDIEDEDTSEKVRRDA
EARCGHPGQPCGKAKREPEARCGHPGQPCGKAKREAEARCGHPGQPCGKAKREADPEAEA
RCGHPGQPCGKLRRAAEAIAEAVAEAEPEARCGHPGQPCGKAKRDAHALAHASDVALSQL
*

>A_macrosclerotiorum_ppg1
MKFTAAIAALILAATVTSSALPSADARCGYPGQPCSKMIKARLAEPEANARCGYPGQPCG
KAKRVALPEPEADARCGYPGQPCGKAKREADARCGYPGQPCSKVKRAAEAFAEALAAPEA
SPDNDHGLAARCDMGSCFEAKRLTRDLASIVADTTEDPAAFYHSLGLETEEVSENKKREA
EARCGYPGQPCGKVKRDAMPEPEADVRCGYPGQPCNKAKREADPEADARCGYPGQPCSKL
KRAAEAVAEAIAEAEPSARCGYPGQPCSKAKRDAFALAHAADLVLGNL*

>M_scopiformis_ppg1
MKFTAAIAALILAATVTATALPNADARCGYPGQPCGKVKARLAEPEADARCGYPGQPCGK
AKRDAMPDADARCGYPGQPCGKVKRAAEAFAEALVPKPGHILPRDLHLTARCDMGSCYEA
KRMTRDLAAIVAETTEDPAAYYVSLGLEDEETSEKLKREASARCGYPGQPCGKVKRDAAP
EPEADARCGYPGQPCGKAKREADPEADARCGYPGQPCGKLKRAAEAVAAAIAEAEPAARC
GYPGQPCGKAKRDAFALAHAVDLVLGNL*

>P_spD728_ppg1
MKLTAAIAALILAATVSASALPNPDARCGYPGQPCGKVKARLAEPEADARCGYPGQPCGK
AKREADARCGYPGQPCGKVKRAAEAFAAALAEPSNDKLSTRDNDALAARCDMGSCYEAKR
MTRDLAAIVAETTDDPAAFYHSLNLEDEETSEKLKREAEARCGYPGQPCGKVKRDAAPEP
EADARCGYPGQPCGKAKREADPEADARCGYPGQPCGKLKRAAEAVAEAIAEAEPSARCGY
PGQPCGKAKRDAFAMAHAADIVLGNL*

>P_subalpina_ppg1
MKFTAAIAALILAATVTSSALPSADARCGYPGQPCTKMVKARLAEPEANARCGYPGQPCG
KAKREADARCGYPGQPCSKVKRAAEAFAEALASPEASADNDHGLAARCDMGSCFEAKRMT
RDLAAIVADTTEDPAAFYHSLGLETEEVSENKKREAEARCGYPGQPCGKVKRDAAPEPED
DARCGYPGQPCNKAKREADPEADARCGYRGQPCSKLKRAAEAVAEAIAEAEPTARCGYPG
QPCSKAKRDAFALAHAADLVLGNL*

>E_pisi_ppg1
MKITIFLAALAFFTTSLGASIPNKIEDRAMLHDTTIKPSLIEARDPRNGRQNCGYVGQGC
PGG*

>E_pulchra_potential_ppg1
MKLTSFLAALAFFTTSLCASIPIKIDGVMLDDTTIQSSLIEARDPRKGRENCGYVGQACP
GG*

```
>G_cichoracearum_ppg1
MKSTGLLFALALLTALLCSSLPSVAHDLLHLEASSSAKTSPKIIRDPRKGRQTCGRPGQA
CSGG*

>G_magnicellulatus_ppg1
MKSTSLLFALALLTAPLCSSLPSTAHDLFHLEASSSGKPSPIIVRDPRKGRQTCGRPGQA
CSGG*

>O_heveae_ppg1
MKITIFLAAVTFFTTSLSASIPIKIEDRVVPDDTTIKSPLIEARDPRNGRQNCGYVGQGC
PGG*

>O_neolycopersici_ppg1
MKLTNFLAALTFFTTSFCASIPIQIDGVMLDDTTIKSSLIETRDPRKGRQNCGYVGQACP
GG*

>P_cerasi_ppg1
MKLVVTLVALFVSTVSSMVLPHASLAAIIYPREARPQPAPNPVPKPWRKDHCGRPGQACV
GG*

>P_moricola_ppg1
MKSIKLLAVLAVAVAVTALHINIPKIADNVTLEQPQTTPDRVQPRGPRSTKAHCGRPGQV
CMGG*

>P_xanthii_ppg1
MKPILALPLFILLITMVSSIALPHTSSSSTITQREAKPQPVPKPEPRPWRKDHCGRPGQP
CPGG*

>C_longipes_ppg1
MQLTSALCAIILAVTVSSAALPIAEPVTAAIAEPLCGARGQPCGKAKREAEAAPRCGARG
QPCGKAKREAEAAPRCGARGQPCGKVKRAAEAFAEALAEPIPEPQEDSLYARHALQLTRD
LASIVAETQDDPEAFFASLSLPEDSEVDKRDALARCGARGQPCGKAKREAEADPEAEARC
GARGQPCGKLRRAAEAIAEAVAYAEPEARCGARGQPCGKAKRNALAMAHAADIALAAL*

>C_variabilis_ppg1
MKVTSTLFIVAMATAATAMAIPAAEPEPLPWCQWKGQGGCRRSAEPEPLPWCQWKGQGGC
RAKREAEAEAEPMPWCQWKGQGGCKEKL*

>H_bicolor_ppg1
MQLTNALCVLILAVTVSSTALPNAQPAAAAIVEPRCGARGQPCGKAKREAAPEPAPRCGA
RGQPCGKAKREAAPEPEAAPRCGARGQPCGKAKREAEAAPRCGARGQPCGKVKRAAEAFA
EAIAEPTAEPQEDSVYARHALQLTRDLASVVAETQDDAEAFWTTLSLPEDGESEVDKRDA
LARCGARGQPCGKAKRDAEAEADPEAEARCGARGQPCGKLRRAAEAIAEAVAVSEPEARC
GARGQPCGKAKRDALALAHAADIVLSAL*

>H_hepaticicola_ppg1
MHLTNTLCALILAVTVSSVALPNAEPIAAAVANAQWDNDWGCGERGQPCGKVKRAAEAFA
EALAEPNAQPELDSPYARYARQLTRDLAAVVAETQDDPVKYFASLALRDDSDLDKRDALA
EAGWGWGERGQPCGKAKRDADPAAWDWGCGERGQPCGKLRRAAEAIAEAVAVPESEAWDW
GCGERGQPCGKAKRDALALAHAADIILRTI*

>H_variabilis_ppg1_trunc
MQLTRALCALILAVTVSSAALPVAYPIANAIAEPVALCGRRGEPCGKVKREAEAEPVAEP
VALCGRRGEPCGKVKLAEPVALCGRRGEPCGKAKREAEAEPVAEPVARCGRRGEPCGKVK
REAEAEPEAEPEAEPVARCGRRGEPCGK*
```

>A_encephaloides_ppg1
MKFANSLCAIALLASANALPTAEDTAAAATGPPSGGPPPGYFSLIQHLDSNFEKRNAEAV
AEACQWWGQGCWKAKREAEAVEKRASEAEAEPCQWWGQGCWKAKVKREAEAEAEAIAKRD
AEAEAEPCQWWGQGCWKTKVKREAEAITKRYAEAAAEALAEPNAEAIAKRDANAEAEPCQ
WWGQGCWKEKVKREAEAAAEAVAEACQWWGQGCWKAKRDAEAVTKSHAAAEAEAAAAAEA
KA*

>H_albidus_ppg1
MKLTNTLCVLILAFTASAAPNASPNPEANPFAAAVAEAEACYSVGGACYNAKRAATPEAL
AEAEACRYAGQGCWKAKRTAEALANPEACKYAGQGCWKIKREAEAEPEPEAHPAAEACRY
AGQGCWKAKREAAAAAEAEACKYVGQGCWKAKREASALAEAEACRYAGQGCWKAKREADA
EAEACKYVGQGCWKAKRAAEAFAEALAEPFAEPNEKISARCDLVGGACYNAKRDALAMAE
AVAAAQPNPEEFLATLKLRNPDIDALEAEKKIKREPCRYAGQGCWKRDAEAVEKRSPSPE
ALAGADAEACKYVGQGCWKVKRAAEALAEAAANPEPCLRVGGACFQAKRDFHAFENVARS
VIASQA*

>H_fraxineus_ppg1
MKLTNTLCVLILAFTASAAPNASPNPEANPFAAAVAEAEACYSVGGACYNAKRAATPEAL
AEAEACRYAGQGCWKAKRTAEALANPEACKYAGQGCWKIKREAEAEPEPEAHPAAEACRY
AGQGCWKAKREAAAAAEAEACKYVGQGCWKAKREASALAEAEACKYAGQGCWKAKRAAAA
DAEAEACKYVGQGCWKAKREADAEACQYVGQGCWKAKRAAEAFAEALAEPFAEPNEKISA
RCDLVGGACYNAKRDALAMAEAVAAAQPNPEEFLATLKLRNPDIDALEAAKKIKREPCRY
AGQGCWKRDAEAVEVRDQIARRCFKFVKGFRPDLCWKQKRSPSPEALAEADAKADAEACK
YVGQGCWKVKRAAEALAEAAANPEPCLRVGGACFQAKRDLHAFENVARSVIASQA*

>H_fructigenus_ppg1
MRLANTLCVLILAATSVSAAPNANPDPFAAAVAEAEACYSVGGACYNAKRAASPEALAEA
EACRRVGQSCWKAKRTANALANPEACRRVGQSCWKVKREADAEAEAEPEACRRVGQSCWK
AKRAAEAEAEAEACRRVGQSCWKAKRDADAEACRRVGQSCWKVKRAAEAVAEAFAEPTEQ
LEARCDLVGGACYNAKRDALAIAEAVAAASPDGEEFMSKLKLRNPDIDAEEAENKIKREA
EAEAEACRRVGQSCWKNKRDANPEAEAEADAEACRRVGQSCWKVKRAAEALADAAANPEP
CRRVGQSCWKAKRDLHAFENVARSILA*

>H_herbarum_ppg1
MRLTNTLCVLILAATSVSAAPNANPDPFAAAVAEAEACYSVGGACYNSKRAASPEALAEA
EACRRVGQSCWKAKRTADALANPEACRRVGQSCWKVKREADAEAEAEPEACRRVGQSCWK
AKRAAEAEAEAEACRRVGQSCWKAKRDADAEACRRVGQSCWKVKRAAEAVAEAFAEPTEQ
LEARCDLVGGACYNAKRDALAIAEAVAAASPDGEEFISKLKLRNPDIDAEEAEKKIKREA
EAEAEACRRVGQSCWKNKRDANPEAEAEADAEACRRVGQSCWKVKRAAEALAEAAADPEP
CRRVGQSCWKAKRDLHAFENVARSILA*

>H_infarciens_ppg1
MRLTNTLCVLILAATSVSAAPNANPDPFAAAVAEAEACYSIGGACYNAKRAASPEALAEA
EACRRVGQSCWKAKRTADALANPEACRRVGQSCWKVKREADAEAEAEPEACRRVGQSCWK
AKRAAEAEAEAEACRRVGQSCWKAKRDADAEACRRVGQSCWKVKRAAEAVAEAFAEPTEQ
LEARCDLVGGACYNAKRDALAIAEAVAAASPDGEEFMSKLKLRNPDIDAEEAEKKIKREA
EAEAEACRRVGQSCWKNKRDANPEAEAEADAEACRRVGQSCWKVKRAAEALAEAAANPEP
CRRVGQSCWKAKRDLHAFENVARSVLA*

>H_koreanus_ppg1
MKLTNTLCVLILAFTASAAPNASPNPEANPFAAAVAEAEACYSVGGACYNAKRAATPEAL
AEAEACRYAGQGCWKAKRTAEALANPEACKYAGQGCWKINREAEAEPEPEAHPAAEACRY
AGQGCWKAKREAAAAAEAEACKYVGQGCWKAKRETSALAEAEACRYAGQGCWKAKRAAAA
DAEAEACRYAGQGCWKAKREADAEACKYVALAEPFAEPNEKISARCDLVGGACYNAKRDA
LAMAEAVAAAQPNPEEFLATLKLRNPDIDALEAEKKIKREPCRYAGQGCWKRDAEAVEKR
SPSPEALAEADAKADAEACKYVGQGYWKVKRAAEALAEAAANPEPCLRVGGACFQAKRDL

HAFENVARSAIASQA*

>H_linearius_ppg1
MKLTNTLCVLILAFTASAAPNAEPNPFAAAVAEAEACYSVGGQCYNAKRAATPEALAAAE
ACKYVGQGCWKAKRTAAALANPEACKYVGQGCWKVKREAEAHPEPEAEACKYVGQGCWKA
KRAAEAEAEAEACKYVGQGCWKAKREADAEACKYVGQGCWKAKRAAEAFAEALAEPFAEP
NEKISARCDLVGGACYNAKRDALAMAEAVAAAQPNPEEWLANLKLRNPDIDALEAEKKIK
REAEPCKYVGQGCWKEKRDAEVREQLRRRCRMVAGWHPDLCWKQKREATPEADAEACKYV
GQGCWKVKRAAEALAEAAANPEPCLRLGGACFQAKRDLLAFETVARSVIANQA*

>H_occultus_ppg1
MKLTNTLCVLILAFTASAAPNASPNPEANPFAAAVAEAEACYSVGGACYNAKRAATPEAL
AEAEACRYAGQGCWKAKRTAEALANPEACKYAGQGCWKINREAEAEPEPEAHPAAEACRY
AGQGCWKAKREAAAAAEAEACKYVGQGCWKAKRETSALAEAEACRYAGQGCWKAKRAAAA
DAEAEACRYAGQGCWKAKREADAEACKYVALAEPFAEPNEKISARCDLVGGACYNAKRDA
LAMAEAVAAAQPNPEEFLATLKLRNPDIDALEAEKKIKREPCRYAGQGCWKRDAEAVEKR
SPSPEALAEADAKADAEACKYVGQGYWKVKRAAEALAEAAANPEPCLRVGGACFQAKRDL
HAFENVARSAIASQA*

>H_repandus_ppg1
MMFTNALCAIALLVSANALPTAVDTTTAAADNNFEKRNAESVASVCQWWGQACLKAKREA
EPVEKRTAEAEVEPSCWKYLVKREAEAAAAEACQWWGQACWKAMRDAEAFTKRHAEAEAN
ASAEAKA*

>H_scutula_ppg1
MKLTNTICTPILAATSASATPTANPNPFAAAVAEAEACYSIGGACHNAKRAATPEALAEA
EACRYVGQSCWKAKRTADALANPEACKYVGQGCWKVKRAAEAEAEPEPEACRYVGKSCWK
AKRAAEAEAEAEACKYSCWKVKRAAKAVAEAFAEPTEQISARCDLVGGACYNAKRDALAM
AEAVAAASPDGEEFLSKLKLRNPDIDAQEAERKLKRETEAGKTKRDANPEAEAEADAEAC
RYVGQSCWKVKRAAEALAEAAANPEPCARPRAICWQAKRDLYAFENIARSILA*

>H_dingleyae_ppg1
MQLTNILSFLAIAATAVSAMPAAEANPAAVAEACQYPGQGCWKLKRDAAASAEAEACLYP
GQGCWKAKREAVAVAEAEACQYPGQGCWKAKREVRTLLISS*

>P_palmicola_ppg1
MKLTTALYVLVLAATTATAIPNPEPLALALAEAEACKRPGQPCWKAKRAAEAMAEAEACK
RPGQPCWKAKRAAEAVAEAEACKRPGQPCWKAKRAAEAEAEAEACKRPGQPCWKAKRAAE
AMAEAEACKRPGQPCWKAKRAAEAEAEACKRPGQPCWKAKREAEAEAEAEAEAEE*

>C_acicularis_ppg1
MKFTNAIAVLILAATSVSAFPTADAADKRAALPEADPQCYQPGEPCWKAKRXAXAXAEAX
CYHPGEPCWKAKREAEAAAEAQCYHPGEPCWKAKRDFLAKKK*

>H_varia_ppg1
MQLANAFCVFILAATSVSAYPNPAEAFDKRLALAEAGSLPSPCIVRPCKTLAHVLAMRCF
CGYKSKREPVADPVAEACIGRPGQPCWKGKRTAVAEAEACIGRPGQPCWKGKREALAEAE
ACIGRPGQPCWKIKREAEAEPEAEACIGRPGQPCWKGKREAVAEADACIGRPGQPCWKGK
REALAEAEACIGRPGQPCWKLKREACIARPGQPCP*

>L_arida_ppg1
MQLTNALCVVILAATTAVTALPEPGCGARGQGCWKIKRAANALAEALAEPAVDVIEARCN
IAGGACQKARMLTRDLAETVAAVQDDPEAYYASLNFEDDSAVETAAIKQVKREAEADARC
GARGQGCWKEKRDAAPAPGCGARGQGCWKEKRVAISAAEAEANPEARCGARGQGCWKIKR
EAVSEADAGCGARGQGCWKVKRAAEAIADALAEPLPGCGARGQGCWKEKRDIQALQNAAR
DALEKL*

>L_cervina_ppg1_trunc
MQLTNTLCVIILAATTAVTALPEPHCGARGQGCWKIKRAANALAEALAEPAVDVINARCN
IAGGACQKARMLTRDLAETVAAVQDDPEAYFASLSFEDDSAVETAATEQVKREADADARC
GARGQGCWKEKRDAAPAPGCGA

>L_hyalina_ppg1
MQLTNTLCVIILAATTAVTALPEPGCGARGQGCWKIKRAANALAEALAEPAVDVIEARCN
IAGGACQKARMLTRDLAETVAAVQDDPEAYYASLSFEDDSAVETAAIKQIKREADADARC
GARGQGCWKEKRDAAPAPGCGARGQGCWKEKREAISAAEAEANPEARCGARGQGCWKIKR
EAVSEADARCGARGQGCWKVKRAAEAIADALAEPLPRCGARGQGCWKQKRDIQALQNVAR
DVLEKL*

>L_occidentalis_ppg1_trunc
EAYYASLSFEDDSAVEAAATEQVKREAEAHNGCGARGQGCPKKRDADADAAPRCGARGQG
CWKEKRVAISAAEAEANPEARCGARGQGCWKVKREAVSEAEARCGARGQGCWKVKRAAEA
IADALAEPLPGCGARGQGCWKEKRDIQALQNAARDALEKL*

>L_subtilissima_ppg1_trunc
CWKIKRAANALAEALAEPAVDVIEARCNIAGGACQKARMLTRDLAETVAAVQDDPEAYYA
SLSFEDDSAVETAAIKQIKREADADARCGARGQGCWKEKRDAAPAPGCGARGQGCWKEKR
EAISAAEAEANPEARCGARGQGCWKIKREAVSEADAGCGARGQGCWKVKRAAEAIADALA
EPLPRCGARGQGCWKEKRDIQALQNVARDVLEKL*

>L_suecica_ppg1
MQLTNALCVLILAATSVVTALPDAGRGCGARGQGCYKRAAVALAEPVAYAGHGCGARGQG
CYKIKRAGEALAEALAEEATSEIDVRCDLAGGACHKARMLARDLADVVAATQDDSEAYFN
SLNIEDADTFAEDAAEHIKREAEAGRGCGARGQGCYKNKRTAEAEAKADAEAEAVAGCGA
RGQGCYKAKREADAEAEAEAGRGCGARGQGCYKEKRDLAALRNVAREALAQL*

>L_willkommii_ppg1
MQLTNALCVVILAATTAVTALPEPGCGARGQGCWKIKRAANALAEALAEPAVDVIEARCN
IAGGACQKARMLTRDLAETVAAVQDDPEAYYASLNFEDDSAVETAAIKQVKREAEADARC
GARGQGCWKEKRDAAPAPGCGARGQGCWKEKRVAISAAEAEANPEARCGARGQGCWKIKR
EAVSEADAGCGARGQGCWKVKRAAEAIADALAEPLPGCGARGQGCWKEKRDIQALQNAAR
DALEKL*

>P_dingleyae_ppg1
MHIIRTLSIAILAATAAATALPNPNAEPWCGGRGQGCWKTKRVALPEPEAFPGCGARGQG
CWKIKRAGVALRDALAEPATGEIDARCNLAGGACFYSRQLARDLATVVASTQDDPEAYFA
SLDLEDEDAAADEAAEKVKREAEAEANCGGRGQGCWKEKREPEPLPGCGARGQGCWKEKR
MAVPEAFAEPGCGARGQGCWKVKREPIPEADARCGARGQGCWKVKRAAEAIAEAVAEAEP
GCGARGQGCWKEKRDFAALQNAARDALEKL*

>C_crateriformis_ppg1
MKVTQVFSVLLVAATVSAVAIPAPEAHCGRLGQPCKREAVAEVTADEIVKREPGPIPEPE
PRCGRMGQPCKREAAPEPVPEPEPRCGRMGQPCKREPGPIPEPEPRCGRMGQPCKREAAP
EPVPEPEPRCGRMGQPC*

>C_cylindrospora_ppg1
MKFTQVFSVLLVAATVSAVAIPAPEAHCGRLGQPCKREALAEVTVDEIAKREPEPVPEPE
PRCGRMGQPCKREAAPEPAPEPEPRCGRMGQPCKREAAPEPAPEPEPRCGRMGQPCKREA
APEPVPEPEPRCGRMGQPCKREAAPEPAPEPKPRCGRMGQPC*

>P_neosporulosa_ppg1
MKFTQAFSVIILAASVSAVALPEARCGRVGQPCKREAAPAPEPRCGRVGQPCKRAAEAVA

DALADPRCGRVGQPCKRDVAEADLDDIVARLVTSDDDFFEKVKREVIAISKREAAPEPEP
EPEPEPEPEARCGRVGQPC*

>P_radicicola_ppg1
MKFTQAFSVIILAASVSAAALPEARCGRIGQPCKREAAPAPEPRCGRIGQPCKRAAEAVA
DALADPRCGRIGQPCKRDVAEADLDDIVARLVTVDNDVFEKVKREVIAISKREAAPEPEP
EAEPEAKCGRIGQPC*
