# Supplementary material for: Needles in fungal haystacks: Discovery of a putative a-factor pheromone and a unique mating strategy in the Leotiomycetes
Source: PLoS One. 2023 Oct 12;18(10):e0292619. doi: 10.1371/journal.pone.0292619 (PMC10569646; doi:10.1371/journal.pone.0292619)
Supplement: S10 File — (PDF) [file pone.0292619.s020.pdf]

#Additional File S10: A pheromone sequences for all Leotiomycetes

>B_globosa_Aprot
MCTSTSTGLVNRNGPGSAASSTISDIVKGTSTTGKTKCLECDGYECCCIPIPCTVM*

>H_repandus_Aprot
MCTSSSNGLVNRNGPGSAAVSTISSIVKPEGSAAGKTKCLECDGVECCCIPIPCTVM*

>B_aclada_Aprot
MCTSTSTGLVNRNGPGSAASSTISDIVKGTSTTGKTKCLECDGYECCCIPIPCTVM*

>B_byssoidea_Aprot
MCTSTSTGLVNRNGPGSAASSTISDIVKGTSTTGKTKCLECDGYECCCIPIPCTVM*

>B_calthae_Aprot
MCTSTSTGLVNRNGPGSAASSTISDIVKGTSTTGKTKCLECDGYECCCIPIPCTVM*

>B_cinerea_Aprot
MCTSTSTGLVNRNGPGSAASSTISDIVKGTSTTGKTKCLECDGYECCCIPIPCTVM*

>B_convoluta_Aprot
MCTSTSTGLVNRNGPGSAASSTISDIVKGTSTTGKTKCLECDGYECCCIPIPCTVM*

>B_deweyae_Aprot
MCTSTSTGLVNRNGPGSAASSTISDIVKGTSTTGKTKCLECDGYECCCIPIPCTVM*

>B_elliiptica_Aprot
MCTSTSTGLVNRNGPGSAASSTISDIVKGTSTTGKTKCLECDGYECCCIPIPCTVM*

>B_fabae_Aprot
MCTSTSTGLVNRNGPGSAASSTISDIVKGTSTTGKTKCLECDGYECCCIPIPCTVM*

>B_fragariae_Aprot
MCTSTSTGLVNRNGPGSAASSTISDIVKGTSTTGKTKCLECDGYECCCIPIPCTVM*

>B_galanthina_Aprot
MCTSTSTGLVNRNGPGSAASSTISDIVKGTSTTGKTKCLECDGYECCCIPIPCTVM*

>B_hyacinthi_Aprot
MCTSTSTGLVNRNGPGSAASSTISDIVKGTSTTGKTKCLECDGYECCCIPIPCTVM*

>B_medusae_Aprot
MCTSTSTGLVNRNGPGSAASSTISDIVKGTSTTGKTKCLECDGYECCCIPIPCTVM*

>B_narcissicola_Aprot
MCTSTSTGLVNRNGPGSAASSTISDIVKGTSTTGKTKCLECDGYECCCIPIPCTVM*

>B_paeoniae_Aprot
MCTSTSTGLVNRNGPGSAASSTISDIVKGTSTTGKTKCLECDGYECCCIPIPCTVM*

>B_porri_Aprot
MCTSTSTGLVNRNGPGSAASSTISDIVKGTSTTGKTKCLECDGYECCCIPIPCTVM*

>B_pseudocinerea_Aprot
MCTSTSTGLVNRNGPGSAASSTISDIVKGTSTTGKTKCLECDGYECCCIPIPCTVM*

```
>B_sinoallii_Aprot
MCTSTSTGLVNRNGPGSAASSTISDIVKGTSTTGKTKCLECDGYECCCIPIPCTVM*

>B_squamosa_Aprot
MCTSTSTGLVNRNGPGSAASSTISDIVKGTSTTGKTKCLECDGYECCCIPIPCTVM*

>B_tulipae_Aprot
MCTSTSTGFVNRNGPGSAASSTISDIVKGTSTTGKTKCLECDGYECCCIPIPCTVM*

>C_camelliae_Aprot
MCTSTSSTGLVNRNGPGSAASSTISDIVKATNTTGKTKCLECDGYECCCIPIPCTVM*

>C_shiraiana_Aprot
MCTSTSTGLVNRNGPGSAASSTISNIVKGTNTTGKTKCIECDGYECCCIPIPCTVM*

>M_aucupariae_Aprot
MCTSTSTGLVNRNGPGSAASSTISDIVKGTSTTGKTKCLECDGYECCCIPIPCTVM*

>M_curreyana_Aprot
MCTSTSTGLVNRNGPGSAASSTISDIVKGTNSTGKTKCLECDGYECCCIPIPCTVM*

>M_duriaeana_Aprot
MCTSTSTGLVNRNGPGSAASSTISDIVKGTNTTGKTKCLECDGYECCCIPIPCTVM*

>M_fructicola_Aprot
MCTSNSTGLVNRNGPGSAASSTISDIVKGTNTTGKTKCLECDGYECCCIPIPCTVM*

>M_fructigena_Aprot
MCTSNSTGLVNRNGPGSAASSTISDIVKGTNTTGKTKCLECDGYECCCIPIPCTVM*

>M_laxa_Aprot
MCTSNSTGLVNRNGPGSAASSTISDIVKGTNTTGKTKCLECDGYECCCIPIPCTVM*

>M_polystroma_Aprot
MCTSNSTGLVNRNGPGSAASSTISDIVKGTNTTGKTKCLECDGYECCCIPIPCTVM*

>M_scirpicola_Aprot
MCTSTSTGLVNRNGPGSAASSTISDIVKGTNTTGKTKCLECDGYECCCIPIPCTVM*

>M_vacciniicorymbosi_Aprot
MCTSNSTGLVNRNGPGSAASSTISDIVKGTSATGKTKCLECDGYECCCIPIPCTVM*

>S_borealis_Aprot
MCTSTSTTSLVNRNGPGSAASSTISDIVKGGSATGKTKCLECDGYECCCIPIPCTVM*

>S_cepivorum_Aprot
MCTSTSTGLVNRNGPGSAASSTISDIVKGTNTTGKTKCIECDGYECCCIPIPCTVM*

>S_glacialis_Aprot
MCTSTSAGLVNRNGPGSAASSTISDIVKGTNTTGKTKCIECDGYECCCIPIPCTVM*

>S_sclerotiorum_Aprot
MCTSTSTGLVNRNGPGSAASSTISDIVKGTNTTGKTKCIECDGYECCCIPIPCTVM*

>S_trifoliorum_Aprot
MCTSTSTGLVNRNGPGSAASSTISDIVKGTNTTGKTKCIECDGYECCCIPIPCTVM*
```

>C_jacksonii_Aprot
MCTSSSTLVNRNGPGSAASSTVSSIVKGTNSTGKTKCIECDGYECCCIPIPCTVM*

>C_homoeocarpa_Aprot
MCTSSSTLVNRNGPGSAASSTVSSIVKGTNSTGKTKCIECDGYECCCIPIPCTVM*

>C_spSE16F4_Aprot
MCTSSSTLVNRNGPGSAASSTVSSIVKGTNSTGKTKCIECDGYECCCIPIPCTVM*

>C_monteithiana_Aprot
MCTSSSTLVNRNGPGSAASSTVSNIVKGTNSTGKTKCIECDGYECCCIPIPCTVM*

>C_spCPB17_Aprot
MCTSSSSTLVNRNGPGSAASSTISNIVKGTNSTGKTKCIECDGYECCCIPIPCTVM*

>R_spNJR2017aWRK4_Aprot
MCTSSSSTLVNRNGPGSAASSTISNIVKGTNSTGKTKCIECDGYECCCIPIPCTVM*

>R_spNRJ2017aBBW_Aprot
MCTSSSSTLVNRNGPGSAASSTISNIVKGTNSTGKTKCIECDGYECCCIPIPCTVM*

>R_spNRJ2017aBVV2_Aprot
MCTSSSSTLVNRNGPGSAASSTISNIVKGTNSTGKTKCIECDGYECCCIPIPCTVM*

>R_sydowiana_Aprot
MCNSTSSTLVNRNGPGSAASSTISGLVKSDTNAPKCFSCDGVDCCCIPIPCTVM*

>R_agropyri_Aprot
MCTSSSSSSSSSSSPSLVNNNLPGSSASSTISSIVKGDAAGRTKCIECDGYECCCIPIPC
TVM*

>R_commune_Aprot
MCISSSSSSSSPSLVNNNLPGSFASSTISSIVKGDAAGKTKCIECDGYECCCIPIPCTVM
*

>R_secalis_Aprot
MCTSSSSSSSPSLVNNNLPGSSASSTISSIVKGDAAGKTKCIECDGYECCCIPIPCTVM*

>RspMPIPUGEAT0058_Aprot
MCTSTSTLVNRNGPGSAASSTISSIVKGEGGAAGKTKCIECDGYECCCIPIPCTVM*

>C_malorum_Aprot
MCTSTSSTLVNRNGPGSAASSTISSIVKGEGGAAGKTKCIECDGYECCCIPIPCTVM*

>N_avenae_Aprot
MCTSNSPLVNNNLPGSAASSTIGSIVKGETAGKTKCIECDGYECCCIPIPCTVM*

>L_spMPISDFRAT0119_Aprot
MCTSTSQSSLVNRNGPGSAASSTISSIVKGEGGAAGKTKCIECDGYECCCIPIPCTVM*

>B_jaapii_Aprot
MCTSTASSLVNRNGPGSAASSTISSIVKGGAATGKTKCIECDGYECCCIPIPCTVM*

>D_rosae_Aprot
MYASTPAPRGNCHGPGSAASATVSGIVKGEGSAGGKTKCIECDGYECCCIPLPCVVM*

>D_brunnea_Aprot
MCSSTSAPTLVNRNGPGSAASSTIESIVKGGPATGKTKCIECDGYECCCIPIPCTVM*

>M_coronariae_Aprot
MCISAPAARVNRNGPGSAAASTVGSLVKGEGAAAGKTRCIECDGYECCCIPIPCVVM*

>A_macrosclerotiorum_Aprot
MYTSSSNGLVNRNGPGSSAASTISSIVKPETGASGKTKCIECDGYECCCIPIPCTVM*

>P_spD728_Aprot
MCTSSSSILVNRNGPGSAAASTISSIVKPESGATGKTKCIECDGYECCCIPIPCTVM*

>P_subalpina_Aprot
MCTSSSSSLVNRNGPGSSAASTISSIVKPETGASGKTKCIECDGYECCCIPCTVM*

>M_scopiformis_Aprot
MCTSSTSVLVNRNGPGSAAASTISSIVKPESGGAGKTKCIECDGYECCCIPIPCTVM*

>P_shiraiana_Aprot
MALGSIQLINHNGIETLGSSISSTTSTSPSFVKSQDIGKIKCFECDGISCCCIPIPCVVM
*

>P_moricola_Aprot
MASRILQFVNNNGSSITTSSTCSNNKPNPANAPSKIKCLECDGFQCCCIPIPCVVM*

>B_hordei_Aprot
MGISAFPPTLTANKPGSAAVSTVSSIVKPCDGASAPRKCIECDGFECCCIPIPCVVM*

>B_graminis_Aprot
MGISASPPTLTANKPGSAAISTVSSIVKPCEGASAPRKCIECDGFECCCIPIPCIVM*

>P_leucotricha_Aprot
MLPLVNANKPGSAAASTISSLVKPDTGAAGQTKCLECDGYQCCCIPIPCTVM*

>P_cerasi_Aprot
MLPTGNQPAAAASTIASLVKPDAAGPGQTKCLECDGYQCCCIPIPCTVM*

>P_xanthii_Aprot
MFPIVNENKLGSAAASTVSSMVKPDAAVSGKTKCLECDGYTCCCIPIPCTVM*

>H_variabilis_Aprot
MCTSISHSLVNRNGPGSAASSTISSIVKPEGGAAGKTKCIECDGYECCCIPIPCTVM*

>H_bicolor_Aprot
MCTSSTSQLVNRNSPGSAASSTISSIVNPEGGAAGKTKCIECDGYECCCIPIPCTIL*

>H_hepaticicola_Aprot
MCSSTSHSLVNQNGPGSAASSTISAIVKPEGGAAGKTKCIECDGYECCCIPIPCTVM*

>C_longipes_Aprot
MCTSGSHSLVNHNGPGSAASSTISSIVKPEGGAAGKTKCIECDGYECCCIPCVVM*

>C_variabilis_Aprot
MCISSSNSLVNRNGPGSAASSTISTIVKHQSGATGKTRCIECDGYECCCIPIPCTVM*

```
>A_encephaloides_Aprot
MCSNGLVNRNGPGSAASSTISSIVKPEGSAAGKIKCLECDGVDCCCIPIPCTVM*

>H_koreanus_Aprot
MCTTTSSGLVNRNGPGSAAASTISSIVKPETGATGKTKCFECDGVECCCIPIPCVVM*

>H_occultus_Aprot
MCTTTSSGLVNRNGPGSAAASTISSIVKPETGATGKTKCFECDGVECCCIPIPCVVM*

>H_linearis_Aprot
MCTSSSGLVNRNGLGSAAASTISSIVKPDSGATGKTKCFECDGVECCCIPIPCVVM*

>H_infarciens_Aprot
MCTSSSSTLVNRNGPGSAAASTISSIVKPESGATGKTKCFECDGIDCCCIPIPCTVM*

>H_herbarum_Aprot
MCSSSSSSLVNRNGPGSAAASTISSIVKPESGATGKTKCFECDGVECCCIPIPCTVM*

>H_fructigenus_Aprot
MCTSSSSTLVNRNGPGSAAASTISSIVKPESGATGKTKCFECDGVECCCIPIPCTVM*

>H_scutula_Aprot
MCTTSSSSTLVNRNGPGSSASSTISSIVKPSTGATGKTKCFECDGVECCCIPIPCTVM*

>H_dingleyae_Aprot
MCTTSHTLVNRNGPGSAASSNVSSIVKPDSGAAGKTKCFTCDGIDCCCIPCTVM*

>P_palmicola_Aprot
MCTSSSNGLVNRNGPGSAAASTISSIVKPETGAAGKTKCLECDGYECCCIPIPCTVM*

>G_lozoyensis_Aprot
MATSVHQTPVNANKPGSSAASTISSIVKPETGKTKCLECDGYECCCIPIPCVVM*

>L_hyalina_Aprot
MCTSTSSSTLVNLNGPGSAASSTISSIVKPDGGAGGTKSFCIECDGFECCCIPIPCTVM*

>L_subtilissima_Aprot
MCTSTSSALVNRNGPGSAASSTISSIVKPDGGAGGTQNFCIECDGYNCCCIPIPCTVM*

>L_occidentalis_Aprot
MCASISTSTLVNRNGPGSAASSTISSIVKPDGGAAGMPKCIECDGISCCCLPIPCTVM*

>L_arida_Aprot
MCASTSTSLVNRNGPGSAASSTISSIVKPEAGAAGKTKCIECDGYECCCIPIPCTVM*

>L_willkommii_Aprot
MCASTSNSLVNRNGPGSAASSTISSIVKPEGGAAGKSKCLECDGVECCCIPIPCTVM*

>L_suecica_Aprot
MCTSSSTGLVNRNGPGSAASSTISSIVKAEGGAAGKTKCIECDGYECCCIPIPCTVM*

>P_dingleyae_Aprot
MCTSSSTLVNRNGPGSAASSTISSIVKPEGGSSGKTKCIECDGFECCCIPIPCTVM*

>P_radicicola_Aprot
MCTSSSSQLVNRNGPGSAAASTISSIIKPESGAAGKTKCIECDGYECCCIPIPCTVM*
```

>C_crateriformis_Aprot
MCTSTTLTLVNRNGPGSAAASTISSIVKPEGAAAGKTKCIECDGYECCCIPIPCTVM*

>C_cylindrospora_Aprot
MCTSTTFTLVNRNGPGSAAASTISSIVKPEGAAAGKTKCIECDGYECCCIPIPCTVM*

>P_destructans_Aprot
MCQSTAPSFVNRNGPGSAASNTISSIVKPEAPKKSCISCDGYDCCCIPLPCSIM*

>P_pannorum_Aprot
MCQSSAPSFVNRNGPGSAASSTISSIVKPEAEKKKCFSCDGYDCCCIPIPCSVM*

>P_verrucosus_Aprot
MCQSSAPSFVNRNGPGSAAASTISSIVKPEAPKKSCISCDGYDCCCIPIPCSVM*

>A_pellizariae_Aprot
MPPSNTTGSSAASIISSIVKPEVSPCLDCGEYSCCCIPIPCTVM*

>A_resinae_Aprot
MCTATPSAPINHNGPGSAASSTISSIVKPEAGAAGKTKCLECDGFECCCIPIPCTVM*

>A_sarcoides_Aprot
MCTSTHILVDKNGPGSAAASTISTIVKPETGKTKCLECDGYDCCCIPIPCVVM*

>A_tetracladia_Aprot
MCTSTPNTLVNRNGPGSAAASTISSIVKSEGAGKTKCIECDGYECCCIPCTVM*

>C_acicularis_Aprot
MCQSSTSGLVNRNKGPGSAASSIISGIVKGEGTGGAGAGKTKCLECDGYECCCIPIPCVV
M*

>C_aeruginascenes_Aprot
MALITFTLANRNGPGSSAASTVSSIVKPEAGSAGRTKCLECDGYECCCIPIPCTVM*

>C_marina_Aprot
MCTTSPATLADRNGPGSVATSIVSGIVKPESGAAGKTRCIECDGYECCCIPIPCTVM*

>G_debralockiae_Aprot
MCTSSSTLVNLNGPGSSAASTISSIVKPETGAAGKTKCLECDGYECCCIPIPCTVM*

>H_hymeniophilus_Aprot
MCTSSTVSLVNRNGPGSSAASTISSIVKPDSGAAGKTKCLECDGYECCCIPIPCTVM*

>H_varia_Aprot
MCSSASSTLVNRNGPGSAAASTISSIVKPDSGAAGKTKCIECDGYECCCIPIPCTVM*

>M_aquatica_Aprot
MCTSSPTTLVNRNGPGSAAASTISSIVKGDGGAAGKTKCIECDGYECCCIPIPCTVM*

>O_maius_Aprot
MCFSTTHGLVSLNGPGSSASSTISSLVKPESGTAGKTKCIECDGFECCCIPIPCTVM*

>S_lignicola_Aprot
MISSSNSLVNHNGGPGSSAASTISSIVKPETSTTGKTKCIECDGFECCCIPIPCTVM*

>V_echinocandica_Aprot
MRTSNSSLVNRNGPGSAAASTISSIVKPETGAAGKTKCLECDGYECCCIPIPCTVM*
